# Posicionamento sobre o Consumo de Gorduras e Saúde Cardiovascular – 2021

**DOI:** 10.36660/abc.20201340

**Published:** 2021-01-27

**Authors:** Maria Cristina de Oliveira Izar, Ana Maria Lottenberg, Viviane Zorzanelli Rocha Giraldez, Raul Dias dos Santos, Roberta Marcondes Machado, Adriana Bertolami, Marcelo Heitor Vieira Assad, José Francisco Kerr Saraiva, André Arpad Faludi, Annie Seixas Bello Moreira, Bruno Geloneze, Carlos Daniel Magnoni, Carlos Scherr, Cristiane Kovacs Amaral, Daniel Branco de Araújo, Dennys Esper Corrêa Cintra, Edna Regina Nakandakare, Francisco Antonio Helfenstein Fonseca, Isabela Cardoso Pimentel Mota, José Ernesto dos Santos, Juliana Tieko Kato, Lis Mie Misuzawa Beda, Lis Proença Vieira, Marcelo Chiara Bertolami, Marcelo Macedo Rogero, Maria Silvia Ferrari Lavrador, Miyoko Nakasato, Nagila Raquel Teixeira Damasceno, Renato Jorge Alves, Lara Roberta Soares, Rosana Perim Costa, Valéria Arruda Machado

**Affiliations:** 1 Universidade Federal de São Paulo São PauloS Brasil Universidade Federal de São Paulo (UNIFESP), São Paulo, SP – Brasil; 2 Hospital Israelita Albert Einstein Faculdade Israelita de Ciências da Saúde Albert Einstein São PauloSP Brasil Hospital Israelita Albert Einstein (HIAE) - Faculdade Israelita de Ciências da Saúde Albert Einstein (FICSAE), São Paulo, SP – Brasil; 3 Faculdade de Medicina Universidade de São Paulo Laboratório de Lípides São PauloSP Brasil Faculdade de Medicina da Universidade de São Paulo, Laboratório de Lípides (LIM10),São Paulo, São Paulo, SP – Brasil; 4 Hospital das Clínicas Faculdade de Medicina Universidade de São Paulo São PauloSP Brasil Instituto do Coração do Hospital das Clínicas da Faculdade de Medicina da Universidade de São Paulo (FMUSP),São Paulo, São Paulo, SP – Brasil; 5 Instituto Dante Pazzanese de Cardiologia São PauloSP Brasil Instituto Dante Pazzanese de Cardiologia, São Paulo, São Paulo, SP – Brasil; 6 Instituto Nacional de Cardiologia Rio de JaneiroRJ Brasil Instituto Nacional de Cardiologia, Rio de Janeiro, RJ – Brasil; 7 Sociedade Campineira de Educação e Instrução CampinasSP Brasil Sociedade Campineira de Educação e Instrução, Campinas, SP – Brasil; 8 Universidade do Estado do Rio de Janeiro Rio de JaneiroRJ Brasil Universidade do Estado do Rio de Janeiro (UERJ), Rio de Janeiro, RJ – Brasil; 9 Universidade Estadual de Campinas CampinasSP Brasilil Universidade Estadual de Campinas (UNICAMP), Campinas, SP – Brasil; 10 Ministério da Saúde BrasíliaDF Brasil Ministério da Saúde, Brasília, DF – Brasil; 11 Universidade de São Paulo São PauloSP Brasilil Universidade de São Paulo (USP), São Paulo, São Paulo, SP – Brasil; 12 Centro Universitário Senac São PauloSP Brasil Centro Universitário Senac, São Paulo, São Paulo, SP – Brasil; 13 Faculdade de Medicina Universidade de São Paulo Liga de Diabetes São PauloSP Brasil Faculdade de Medicina da Universidade de São Paulo, Liga de Diabetes, São Paulo, São Paulo, SP – Brasil; 14 Santa Casa de Misericórdia de São Paulo São PauloSP Brasil Santa Casa de Misericórdia de São Paulo, São Paulo, São Paulo, SP – Brasil; 15 Núcleo de Alimentação e Nutrição Sociedade Brasileira de Cardiologia Rio de JaneiroRJ Brasil Núcleo de Alimentação e Nutrição da Sociedade Brasileira de Cardiologia, Rio de Janeiro, RJ – Brasil; 16 Hospital do Coração São PauloSP Brasil Hospital do Coração (HCor), São Paulo, São Paulo, SP – Brasil

**Table:** 

Posicionamento sobre o Consumo de Gorduras e Saúde Cardiovascular – 2021	
O relatório abaixo lista as declarações de interesse conforme relatadas à SBC pelos especialistas durante o período de desenvolvimento desta diretriz, 2020.	
Especialista	Tipo de relacionamento com a indústria
Adriana Bertolami	Nada a ser declarado
Ana Maria Lottenberg	DECLARAÇÃO FINANCEIRA A - PAGAMENTO DE QUALQUER ESPÉCIE E DESDE QUE ECONOMICAMENTE APRECIÁVEIS, FEITOS A (i) VOCÊ, (ii) AO SEU CÔNJUGE/COMPANHEIRO OU A QUALQUER OUTRO MEMBRO QUE RESIDA COM VOCÊ, (iii) A QUALQUER PESSOA JURÍDICA EM QUE QUALQUER DESTES SEJA CONTROLADOR, SÓCIO, ACIONISTA OU PARTICIPANTE, DE FORMA DIRETA OU INDIRETA, RECEBIMENTO POR PALESTRAS, AULAS, ATUAÇÃO COMO PROCTOR DE TREINAMENTOS, REMUNERAÇÕES, HONORÁRIOS PAGOS POR PARTICIPAÇÕES EM CONSELHOS CONSULTIVOS, DE INVESTIGADORES, OU OUTROS COMITÊS, ETC. PROVENIENTES DA INDÚSTRIA FARMACÊUTICA, DE ÓRTESES, PRÓTESES, EQUIPAMENTOS E IMPLANTES, BRASILEIRAS OU ESTRANGEIRAS: - PTC: medicamentos
Andre Arpad Faludi	Nada a ser declarado
Annie Seixas Bello Moreira	Nada a ser declarado
Bruno Geloneze	DECLARAÇÃO FINANCEIRA A - PAGAMENTO DE QUALQUER ESPÉCIE E DESDE QUE ECONOMICAMENTE APRECIÁVEIS, FEITOS A (i) VOCÊ, (ii) AO SEU CÔNJUGE/COMPANHEIRO OU A QUALQUER OUTRO MEMBRO QUE RESIDA COM VOCÊ, (iii) A QUALQUER PESSOA JURÍDICA EM QUE QUALQUER DESTES SEJA CONTROLADOR, SÓCIO, ACIONISTA OU PARTICIPANTE, DE FORMA DIRETA OU INDIRETA, RECEBIMENTO POR PALESTRAS, AULAS, ATUAÇÃO COMO PROCTOR DE TREINAMENTOS, REMUNERAÇÕES, HONORÁRIOS PAGOS POR PARTICIPAÇÕES EM CONSELHOS CONSULTIVOS, DE INVESTIGADORES, OU OUTROS COMITÊS, ETC. PROVENIENTES DA INDÚSTRIA FARMACÊUTICA, DE ÓRTESES, PRÓTESES, EQUIPAMENTOS E IMPLANTES, BRASILEIRAS OU ESTRANGEIRAS: - Novo Nordisk: diabetes - AstraZeneca: diabetes - MSD: diabetes OUTROS RELACIONAMENTOS FINANCIAMENTO DE ATIVIDADES DE EDUCAÇÃO MÉDICA CONTINUADA, INCLUINDO VIAGENS, HOSPEDAGENS E INSCRIÇÕES PARA CONGRESSOS E CURSOS, PROVENIENTES DA INDÚSTRIA FARMACÊUTICA, DE ÓRTESES, PRÓTESES, EQUIPAMENTOS E IMPLANTES, BRASILEIRAS OU ESTRANGEIRAS: - Novo Nordisk: diabetes
Carlos Scherr	OUTROS RELACIONAMENTOS FINANCIAMENTO DE ATIVIDADES DE EDUCAÇÃO MÉDICA CONTINUADA, INCLUINDO VIAGENS, HOSPEDAGENS E INSCRIÇÕES PARA CONGRESSOS E CURSOS, PROVENIENTES DA INDÚSTRIA FARMACÊUTICA, DE ÓRTESES, PRÓTESES, EQUIPAMENTOS E IMPLANTES, BRASILEIRAS OU ESTRANGEIRAS: - Bayer: trombose
Cristiane Kovacs Amaral	Nada a ser declarado
Daniel Branco de Araújo	DECLARAÇÃO FINANCEIRA A - PAGAMENTO DE QUALQUER ESPÉCIE E DESDE QUE ECONOMICAMENTE APRECIÁVEIS, FEITOS A (i) VOCÊ, (ii) AO SEU CÔNJUGE/COMPANHEIRO OU A QUALQUER OUTRO MEMBRO QUE RESIDA COM VOCÊ, (iii) A QUALQUER PESSOA JURÍDICA EM QUE QUALQUER DESTES SEJA CONTROLADOR, SÓCIO, ACIONISTA OU PARTICIPANTE, DE FORMA DIRETA OU INDIRETA, RECEBIMENTO POR PALESTRAS, AULAS, ATUAÇÃO COMO PROCTOR DE TREINAMENTOS, REMUNERAÇÕES, HONORÁRIOS PAGOS POR PARTICIPAÇÕES EM CONSELHOS CONSULTIVOS, DE INVESTIGADORES, OU OUTROS COMITÊS, ETC. PROVENIENTES DA INDÚSTRIA FARMACÊUTICA, DE ÓRTESES, PRÓTESES, EQUIPAMENTOS E IMPLANTES, BRASILEIRAS OU ESTRANGEIRAS: - Novo Nordisk: diabetes - Sanofi: dislipidemia OUTROS RELACIONAMENTOS FINANCIAMENTO DE ATIVIDADES DE EDUCAÇÃO MÉDICA CONTINUADA, INCLUINDO VIAGENS, HOSPEDAGENS E INSCRIÇÕES PARA CONGRESSOS E CURSOS, PROVENIENTES DA INDÚSTRIA FARMACÊUTICA, DE ÓRTESES, PRÓTESES, EQUIPAMENTOS E IMPLANTES, BRASILEIRAS OU ESTRANGEIRAS: - Sanofi: dislipidemia
Carlos Daniel Magnoni	Nada a ser declarado
Dennys Esper Corrêa Cintra	Nada a ser declarado
Edna Regina Nakandakare	Nada a ser declarado
Francisco Antonio Helfenstein Fonseca	DECLARAÇÃO FINANCEIRA A - PAGAMENTO DE QUALQUER ESPÉCIE E DESDE QUE ECONOMICAMENTE APRECIÁVEIS, FEITOS A (i) VOCÊ, (ii) AO SEU CÔNJUGE/COMPANHEIRO OU A QUALQUER OUTRO MEMBRO QUE RESIDA COM VOCÊ, (iii) A QUALQUER PESSOA JURÍDICA EM QUE QUALQUER DESTES SEJA CONTROLADOR, SÓCIO, ACIONISTA OU PARTICIPANTE, DE FORMA DIRETA OU INDIRETA, RECEBIMENTO POR PALESTRAS, AULAS, ATUAÇÃO COMO PROCTOR DE TREINAMENTOS, REMUNERAÇÕES, HONORÁRIOS PAGOS POR PARTICIPAÇÕES EM CONSELHOS CONSULTIVOS, DE INVESTIGADORES, OU OUTROS COMITÊS, ETC. PROVENIENTES DA INDÚSTRIA FARMACÊUTICA, DE ÓRTESES, PRÓTESES, EQUIPAMENTOS E IMPLANTES, BRASILEIRAS OU ESTRANGEIRAS: - Novo Nordisk: antidiabéticos - Libbs: hipolipemiantes - Ache: hipolipemiantes C - FINANCIAMENTO DE PESQUISA (PESSOAL), CUJAS RECEITAS TENHAM SIDO PROVENIENTES DA INDÚSTRIA FARMACÊUTICA, DE ÓRTESES, PRÓTESES, EQUIPAMENTOS E IMPLANTES, BRASILEIRAS OU ESTRANGEIRAS: - AstraZeneca: projeto de iniciativa do investigador, já concluído - Amgen: hipolipemiantes - Biolab: vitaminas OUTROS RELACIONAMENTOS FINANCIAMENTO DE ATIVIDADES DE EDUCAÇÃO MÉDICA CONTINUADA, INCLUINDO VIAGENS, HOSPEDAGENS E INSCRIÇÕES PARA CONGRESSOS E CURSOS, PROVENIENTES DA INDÚSTRIA FARMACÊUTICA, DE ÓRTESES, PRÓTESES, EQUIPAMENTOS E IMPLANTES, BRASILEIRAS OU ESTRANGEIRAS: - Bayer: anticoagulante - Sanofi: hipolipemiantes - Amgen: hipolipemiantes
Isabela Cardoso Pimentel Mota	Nada a ser declarado
Jose Ernesto dos Santos	Nada a ser declarado
José Francisco Kerr Saraiva	Nada a ser declarado
Juliana Tieko Kato	Nada a ser declarado
Lis Mie Masuzawa Beda	Nada a ser declarado
Lis Proença Vieira	Nada a ser declarado
Marcelo Chiara Bertolami	DECLARAÇÃO FINANCEIRA A - PAGAMENTO DE QUALQUER ESPÉCIE E DESDE QUE ECONOMICAMENTE APRECIÁVEIS, FEITOS A (i) VOCÊ, (ii) AO SEU CÔNJUGE/COMPANHEIRO OU A QUALQUER OUTRO MEMBRO QUE RESIDA COM VOCÊ, (iii) A QUALQUER PESSOA JURÍDICA EM QUE QUALQUER DESTES SEJA CONTROLADOR, SÓCIO, ACIONISTA OU PARTICIPANTE, DE FORMA DIRETA OU INDIRETA, RECEBIMENTO POR PALESTRAS, AULAS, ATUAÇÃO COMO PROCTOR DE TREINAMENTOS, REMUNERAÇÕES, HONORÁRIOS PAGOS POR PARTICIPAÇÕES EM CONSELHOS CONSULTIVOS, DE INVESTIGADORES, OU OUTROS COMITÊS, ETC. PROVENIENTES DA INDÚSTRIA FARMACÊUTICA, DE ÓRTESES, PRÓTESES, EQUIPAMENTOS E IMPLANTES, BRASILEIRAS OU ESTRANGEIRAS: - Abbott: dislipidemias- fenofibrato - Ache: dislipidemias fenofibrato - Libbs: dislipidemias - fenofibrato - Merck: dislipidemias - fenofibrato - Novo Nordisk: dislipidemias - fenofibrato - Sanofi: dislipidemias - fenofibrato OUTROS RELACIONAMENTOS FINANCIAMENTO DE ATIVIDADES DE EDUCAÇÃO MÉDICA CONTINUADA, INCLUINDO VIAGENS, HOSPEDAGENS E INSCRIÇÕES PARA CONGRESSOS E CURSOS, PROVENIENTES DA INDÚSTRIA FARMACÊUTICA, DE ÓRTESES, PRÓTESES, EQUIPAMENTOS E IMPLANTES, BRASILEIRAS OU ESTRANGEIRAS : - Merck: dislipidemia - estatinas - Novo Nordisk: dislipidemia - estatinas - Sanofi: dislipidemia - estatinas
Marcelo Heitor Vieira Assad	DECLARAÇÃO FINANCEIRA A - PAGAMENTO DE QUALQUER ESPÉCIE E DESDE QUE ECONOMICAMENTE APRECIÁVEIS, FEITOS A (i) VOCÊ, (ii) AO SEU CÔNJUGE/COMPANHEIRO OU A QUALQUER OUTRO MEMBRO QUE RESIDA COM VOCÊ, (iii) A QUALQUER PESSOA JURÍDICA EM QUE QUALQUER DESTES SEJA CONTROLADOR, SÓCIO, ACIONISTA OU PARTICIPANTE, DE FORMA DIRETA OU INDIRETA, RECEBIMENTO POR PALESTRAS, AULAS, ATUAÇÃO COMO PROCTOR DE TREINAMENTOS, REMUNERAÇÕES, HONORÁRIOS PAGOS POR PARTICIPAÇÕES EM CONSELHOS CONSULTIVOS, DE INVESTIGADORES, OU OUTROS COMITÊS, ETC. PROVENIENTES DA INDÚSTRIA FARMACÊUTICA, DE ÓRTESES, PRÓTESES, EQUIPAMENTOS E IMPLANTES, BRASILEIRAS OU ESTRANGEIRAS: - AstraZeneca: prevenção cardiovascular - Boeringher: diabetes/anticoagulação - Novo Nordisk: diabetes OUTROS RELACIONAMENTOS FINANCIAMENTO DE ATIVIDADES DE EDUCAÇÃO MÉDICA CONTINUADA, INCLUINDO VIAGENS, HOSPEDAGENS E INSCRIÇÕES PARA CONGRESSOS E CURSOS, PROVENIENTES DA INDÚSTRIA FARMACÊUTICA, DE ÓRTESES, PRÓTESES, EQUIPAMENTOS E IMPLANTES, BRASILEIRAS OU ESTRANGEIRAS: - Boeringher: diabetes - Novo Nordisk: diabetes
Marcelo Macedo Rogero	Nada a ser declarado
Maria Cristina de Oliveira Izar	DECLARAÇÃO FINANCEIRA A - PAGAMENTO DE QUALQUER ESPÉCIE E DESDE QUE ECONOMICAMENTE APRECIÁVEIS, FEITOS A (i) VOCÊ, (ii) AO SEU CÔNJUGE/COMPANHEIRO OU A QUALQUER OUTRO MEMBRO QUE RESIDA COM VOCÊ, (iii) A QUALQUER PESSOA JURÍDICA EM QUE QUALQUER DESTES SEJA CONTROLADOR, SÓCIO, ACIONISTA OU PARTICIPANTE, DE FORMA DIRETA OU INDIRETA, RECEBIMENTO POR PALESTRAS, AULAS, ATUAÇÃO COMO PROCTOR DE TREINAMENTOS, REMUNERAÇÕES, HONORÁRIOS PAGOS POR PARTICIPAÇÕES EM CONSELHOS CONSULTIVOS, DE INVESTIGADORES, OU OUTROS COMITÊS, ETC. PROVENIENTES DA INDÚSTRIA FARMACÊUTICA, DE ÓRTESES, PRÓTESES, EQUIPAMENTOS E IMPLANTES, BRASILEIRAS OU ESTRANGEIRAS: - Amgen: dislipidemia - Ache: dislipidemia - Novartis: dislipidemia - PTCbio: dislipidemia B - FINANCIAMENTO DE PESQUISAS SOB SUA RESPONSABILIDADE DIRETA/PESSOAL (DIRECIONADO AO DEPARTAMENTO OU INSTITUIÇÃO) PROVENIENTES DA INDÚSTRIA FARMACÊUTICA, DE ÓRTESES, PRÓTESES, EQUIPAMENTOS E IMPLANTES, BRASILEIRAS OU ESTRANGEIRAS: - Amgen: dislipidemia - PTCbio: dislipidemia - Novartis: dislipidemia OUTROS RELACIONAMENTOS FINANCIAMENTO DE ATIVIDADES DE EDUCAÇÃO MÉDICA CONTINUADA, INCLUINDO VIAGENS, HOSPEDAGENS E INSCRIÇÕES PARA CONGRESSOS E CURSOS, PROVENIENTES DA INDÚSTRIA FARMACÊUTICA, DE ÓRTESES, PRÓTESES, EQUIPAMENTOS E IMPLANTES, BRASILEIRAS OU ESTRANGEIRAS: - Amgen: dislipidemia - Novo Nordisk: GLP-1RA - PTCbio: dislipidemia
Maria Silvia Ferrari Lavrador	Nada a ser declarado
Miyoko Nakasato	Nada a ser declarado
Nagila Raquel Teixeira Damasceno	Nada a ser declarado
Raul Dias dos Santos Filho	DECLARAÇÃO FINANCEIRA A - PAGAMENTO DE QUALQUER ESPÉCIE E DESDE QUE ECONOMICAMENTE APRECIÁVEIS, FEITOS A (i) VOCÊ, (ii) AO SEU CÔNJUGE/COMPANHEIRO OU A QUALQUER OUTRO MEMBRO QUE RESIDA COM VOCÊ, (iii) A QUALQUER PESSOA JURÍDICA EM QUE QUALQUER DESTES SEJA CONTROLADOR, SÓCIO, ACIONISTA OU PARTICIPANTE, DE FORMA DIRETA OU INDIRETA, RECEBIMENTO POR PALESTRAS, AULAS, ATUAÇÃO COMO PROCTOR DE TREINAMENTOS, REMUNERAÇÕES, HONORÁRIOS PAGOS POR PARTICIPAÇÕES EM CONSELHOS CONSULTIVOS, DE INVESTIGADORES, OU OUTROS COMITÊS, ETC. PROVENIENTES DA INDÚSTRIA FARMACÊUTICA, DE ÓRTESES, PRÓTESES, EQUIPAMENTOS E IMPLANTES, BRASILEIRAS OU ESTRANGEIRAS: Abbott: cardiologia Ache: cardiologia AstraZeneca: cardiologia Amgen: cardiologia Novo Nordisk: cardiologia Novartis: cardiologia PTC Therapeutics: cardiologia Sanofi/Regeneron: cardiologia B - FINANCIAMENTO DE PESQUISAS SOB SUA RESPONSABILIDADE DIRETA/PESSOAL (DIRECIONADO AO DEPARTAMENTO OU INSTITUIÇÃO) PROVENIENTES DA INDÚSTRIA FARMACÊUTICA, DE ÓRTESES, PRÓTESES, EQUIPAMENTOS E IMPLANTES, BRASILEIRAS OU ESTRANGEIRAS: Amgen: cardiologia Esperion: cardiologia Kowa: cardiologia Sanofi/Regeneron: cardiologia
Renato Jorge Alves	DECLARAÇÃO FINANCEIRA A - PAGAMENTO DE QUALQUER ESPÉCIE E DESDE QUE ECONOMICAMENTE APRECIÁVEIS, FEITOS A (i) VOCÊ, (ii) AO SEU CÔNJUGE/COMPANHEIRO OU A QUALQUER OUTRO MEMBRO QUE RESIDA COM VOCÊ, (iii) A QUALQUER PESSOA JURÍDICA EM QUE QUALQUER DESTES SEJA CONTROLADOR, SÓCIO, ACIONISTA OU PARTICIPANTE, DE FORMA DIRETA OU INDIRETA, RECEBIMENTO POR PALESTRAS, AULAS, ATUAÇÃO COMO PROCTOR DE TREINAMENTOS, REMUNERAÇÕES, HONORÁRIOS PAGOS POR PARTICIPAÇÕES EM CONSELHOS CONSULTIVOS, DE INVESTIGADORES, OU OUTROS COMITÊS, ETC. PROVENIENTES DA INDÚSTRIA FARMACÊUTICA, DE ÓRTESES, PRÓTESES, EQUIPAMENTOS E IMPLANTES, BRASILEIRAS OU ESTRANGEIRAS: - MSD: diabetes - PTC Brasil: dislipidemias - Bayer: anticoagulação - Pfizer: cardiologia - Sandoz: cardiologia - Servier: cardiologia OUTROS RELACIONAMENTOS FINANCIAMENTO DE ATIVIDADES DE EDUCAÇÃO MÉDICA CONTINUADA, INCLUINDO VIAGENS, HOSPEDAGENS E INSCRIÇÕES PARA CONGRESSOS E CURSOS, PROVENIENTES DA INDÚSTRIA FARMACÊUTICA, DE ÓRTESES, PRÓTESES, EQUIPAMENTOS E IMPLANTES, BRASILEIRAS OU ESTRANGEIRAS: - Boehringer: diabetes
Roberta Marcondes Machado	Nada a ser declarado
Roberta Soares Lara	Nada a ser declarado
Rosana Perim Costa	Nada a ser declarado
Valéria Arruda Machado	Nada a ser declarado
Viviane Zorzanelli Rocha	DECLARAÇÃO FINANCEIRA A - PAGAMENTO DE QUALQUER ESPÉCIE E DESDE QUE ECONOMICAMENTE APRECIÁVEIS, FEITOS A (i) VOCÊ, (ii) AO SEU CÔNJUGE/COMPANHEIRO OU A QUALQUER OUTRO MEMBRO QUE RESIDA COM VOCÊ, (iii) A QUALQUER PESSOA JURÍDICA EM QUE QUALQUER DESTES SEJA CONTROLADOR, SÓCIO, ACIONISTA OU PARTICIPANTE, DE FORMA DIRETA OU INDIRETA, RECEBIMENTO POR PALESTRAS, AULAS, ATUAÇÃO COMO PROCTOR DE TREINAMENTOS, REMUNERAÇÕES, HONORÁRIOS PAGOS POR PARTICIPAÇÕES EM CONSELHOS CONSULTIVOS, DE INVESTIGADORES, OU OUTROS COMITÊS, ETC. PROVENIENTES DA INDÚSTRIA FARMACÊUTICA, DE ÓRTESES, PRÓTESES, EQUIPAMENTOS E IMPLANTES, BRASILEIRAS OU ESTRANGEIRAS: - Amgen: evolocumabe - Novo Nordisk: diabetes - AstraZeneca: dapagliflozina OUTROS RELACIONAMENTOS FINANCIAMENTO DE ATIVIDADES DE EDUCAÇÃO MÉDICA CONTINUADA, INCLUINDO VIAGENS, HOSPEDAGENS E INSCRIÇÕES PARA CONGRESSOS E CURSOS, PROVENIENTES DA INDÚSTRIA FARMACÊUTICA, DE ÓRTESES, PRÓTESES, EQUIPAMENTOS E IMPLANTES, BRASILEIRAS OU ESTRANGEIRAS: - Amgen: evolocumabe

## Lista de Siglas

ACC - American College of Cardiology

Acetil-CoA - acetilcoenzima A

AGCC - ácidos graxos de cadeia curta

AHA - American Heart Association

ALA - ácido alfalinolênico

ALP - fosfatase alcalina

ALT - alanina aminotransferase

AMPK - proteína adenosina monofosfato quinase

ApoA-I - apolipoproteína A-I

ApoB - apolipoproteína B

AST - aspartato aminotransferase

AVC - acidente vascular cerebral

CE - colesterol éster

CETP – do inglês cholesterol estertransfer protein (proteína de transferência de colesterol esterificado)

COX 2 - ciclo-oxigenase 2

CPT1- carnitina palmitoil transferase 1

DAC - doença arterial coronária

 DASH - *Dietary Approach to Stop Hypertension*

DCNT - doenças crônicas não transmissíveis

DCV - doença cardiovascular

DHA - ácido docosaexaenoico

DM2 - diabetes melito tipo 2

DPA - ácido docosapentaenoico

DRI - Dietary Reference Intakes

EPA - ácidos eicosapentaenoicos

ERE - estresse de retículo endoplasmático

FMO3 - flavina monoxigenase 3

GGT - gama glutamil transferase

GLUT4 - proteína de translocação de glicose-4

HbA1c - hemoglobina glicada

HMG-CoA redutase - hidroximetilglutaril coenzima A redutase

IAM - infarto agudo do miocárdio

IC - insuficiência cardíaca

iNOS - óxido nítrico sintase induzida

INSAT - ácidos graxos insaturados

IRS1 - substrato 1 do receptor de insulina

JNK - quinase c-Jun N terminal

LPL - lipoproteína lipase

LPS - lipopolissacarídeo

MCP - proteína quimioática de monócitos

MONO - monoinsaturados

 NAFLD - do inglês *nonalcoholic fatty liver disease* (doença hepática gordurosa não alcoólica) 

 NASH - do inglês *nonalcoholic steatohepatitis* (esteato-hepatite não alcoólica) 

NHANES - National Health and Nutrition Examination Survey

Ômega-9 - ácido oleico

PAMPs - padrões moleculares associados a patógenos

PCR - proteína C-reativa

PGE2 - prostaglandina E2

POLI - poli-insaturados

PPARγ-2 - proliferadores do peroxissomo gama-2

 PURE - *Prospective Urban Rural Epidemiology*

RM - ressonância magnética

SAT - ácidos graxos saturados

SBC - Sociedade Brasileira de Cardiologia

SCD1 - estearoil-CoA dessaturase 1

SNC - sistema nervoso central

SQF – síndrome da quilomicronemia familiar

SREBP2 - proteína de ligação ao elemento responsivo a esteroides 2

TLRs - receptores Toll-like

TMA - trimetilamina

TMAO - N-óxido de trimetilamina

TXB2 - tromboxano B2

UCP1 - proteína desacopladora mitocondrial 1

VCT - valor calórico total

 WHI - *Woman’s Health Initiative*

 WHO - *World Health Organization*

γGT - gama-glutamil transpeptidase

ω3 - ômega-3

ω6 - ômega-6

## Definição de Graus de Recomendação e Níveis de Evidência

### Classes (graus) de recomendação:

Classe I: condições para as quais há evidências conclusivas ou, na sua falta, consenso geral de que o procedimento é seguro e útil/eficaz.

Classe II: condições para as quais há evidências conflitantes e/ou divergência de opinião sobre segurança e utilidade/eficácia do procedimento.

Classe IIA: peso ou evidência/opinião a favor do procedimento; a maioria aprova.

Classe IIB: segurança e utilidade/eficácia menos bem estabelecida, não havendo predomínio de opiniões a favor.

Classe III: condições para as quais há evidências e/ou consenso de que o procedimento não é útil/eficaz e, em alguns casos, pode ser prejudicial.

### Níveis de evidência:

Nível A: dados obtidos a partir de múltiplos estudos randomizados de bom porte, concordantes e/ou de metanálise robusta de estudos clínicos randomizados.

Nível B: dados obtidos a partir de metanálise menos robusta, a partir de um único estudo randomizado ou de estudos não randomizados (observacionais).

Nível C: dados obtidos de opiniões consensuais de especialistas.

## Carta de Apresentação

 A Nutrição tem importante papel na gênese das doenças crônicas não transmissíveis (DCNT), consideradas um dos mais importantes problemas de saúde pública da atualidade no mundo e em nosso país. Além da quantidade, a qualidade dos alimentos que consumimos (em particular aqueles que são fonte de gorduras) participa tanto na patogênese das doenças cardiovasculares (DCV) quanto na sua prevenção. Especialistas em todo o mundo têm elaborado, com base em evidências científicas, guias sobre o consumo de gorduras e proposto adequação das quantidades de gorduras, além de limitar o consumo de gorduras saturadas e *trans* . Tem-se priorizado avaliar e propor padrões alimentares mais saudáveis e não valorizar alimentos individualmente, com uma abordagem muito mais racional na prevenção cardiovascular, adequando-se o consumo calórico, a inclusão de grãos, frutas e hortaliças e a restrição de carboidratos refinados, alimentos ultraprocessados, priorizando-se gorduras mais saudáveis, em detrimento das saturadas e *trans* . 

Tal posicionamento tem por objetivo orientar os profissionais de saúde no entendimento sobre as ações dos diferentes ácidos graxos e propor medidas dietéticas adequadas visando à prevenção e ao controle da DCV.

O Departamento de Aterosclerose da Sociedade Brasileira de Cardiologia (SBC-DA) reuniu os maiores especialistas do país para a elaboração deste documento, com o objetivo de transmitir as melhores informações disponíveis para aprimorar a prática clínica em nosso país, de forma clara e objetiva, para a prevenção e o tratamento da DCV.

Sinceramente,

Prof. Dra. Maria Cristina de Oliveira Izar

## Sumário

1. Introdução 169

2. Classificação e Fontes de Ácidos Graxos 171

2.1. Monoinsaturados 171

2.2. Poli-insaturados 171

2.3. Saturados 171

2.4. Trans 171

3. Concentração Plasmática de Colesterol Total e Lipoproteínas 171

4. Concentração Plasmática de Triglicérides 173

5. Doença Cardiovascular e Coronariana 173

5.1. Saturados 173

5.2. Substituição de Saturados por Insaturados 174

5.3. Substituição de Saturados por Carboidratos 174

5.4. Ácidos Graxos Poli-insaturados (Ômega-6) 175

5.5. Ácidos Graxos Poli-insaturados (Ômega-3 Marinho) 175

**5.5.1. Efeitos sobre Doença Vascular Periférica** 176 

**5.5.2. Efeitos sobre Arritmias Cardíacas e Morte Súbita** 176 

**5.5.3. Efeitos na Insuficiência Cardíaca** 177 

5.6. Graxos Poli-insaturados (Ômega-3 Vegetal) 177

5.7. Trans 177

6. Disfunção Endotelial 178

6.1. Pressão Arterial 179

6.2. Acidente Vascular Cerebral 179

7. Inflamação 180

8. Resistência à Insulina e Diabetes 180

9. Doença Hepática Gordurosa 182

9.1. Esteatose Hepática 182

9.2. Ácidos Graxos Saturados e Esteato-hepatite Não Alcoólica 183

9.3. Ácidos Graxos Insaturados e Esteato-hepatite Não Alcoólica 183

9.4. Ácidos Graxos Trans e Esteato-hepatite Não Alcoólica 184

10. Metabolismo Lipídico do Tecido Adiposo 184

10.1. Ácidos Graxos Saturados e Metabolismo do Tecido Adiposo 185

10.2. Ácidos Graxos Insaturados e Metabolismo do Tecido Adiposo 185

11. Alimentos 186

11.1. Óleo de Coco 186

11.2. Óleo de Palma 187

11.3. Chocolate 187

11.4. Manteiga 187

11.5. Lácteos 188

11.6. Carnes 188

12. Microbiota Intestinal 188

12.1. Alimentares e Microbiota Intestinal 189

12.2. Importância do Padrão Alimentar na Síntese de Ácidos Graxos de Cadeia Curta 189

13. Colesterol Alimentar 189

13.1. Concentração Plasmática de Lípides e Lipoproteínas 189

13.2. Risco de Desenvolvimento de Diabetes Melito Tipo 2 190

13.3. Risco de Doenças Cardiovasculares em Diabetes Melito Tipo 2 190

13.4. Impacto nas Doenças Cardiovasculares 190

14. Ovo 190

14.1. N-óxido de Trimetilamina nas Doenças Cardiovasculares 191

14.2. Esteatose Hepática 192

15. Gorduras Interesterificadas 192

15.1. Estudos com Animais 192

15.2. Estudos em Humanos 192

16. Triglicérides de Cadeia Média 193

17. Síndrome da Quilomicronemia Familiar 193

18. Aspectos Práticos Da Intervenção Nutricional 193

19. Rotulagem e Ácidos Graxos Trans 194

20. Considerações Finais 194

21. Quantidade de Ácidos Graxos e Colesterol em Alimentos 195

22. Grau de Recomendação e Nível de Evidência: Ácidos Graxos e Risco Cardiovascular 198

Referências 198

## 1. Introdução

 A relevância da dieta na gênese das doenças crônicas não transmissíveis (DCNT) encontra-se bem documentada na literatura. ^1^ O conjunto dessas doenças é considerado um dos mais importantes problemas de saúde pública da atualidade e responde por aproximadamente 71% da mortalidade mundial. ^2^ No Brasil, em 2016, as DCNT foram associadas a 74% do total de mortes, com destaque para doenças cardiovasculares (DCV). ^3^ A qualidade e a quantidade dos alimentos, em particular as fontes de gorduras, influenciam tanto a patogênese quanto a prevenção das DCV. 

 Diretrizes e posicionamentos sobre o consumo de gorduras vêm sendo elaboradas há mais de 50 anos, tendo sido a American Heart Association (AHA), pioneira na elaboração deste documento. ^4^ Nas últimas décadas, órgãos governamentais e sociedades médicas internacionais, tais como World Health Organization (WHO), United States Government, Institute of Medicine of USA, European Food Safety Authority, entre outros, têm se empenhado na elaboração de documentos fundamentados em estudos de alto rigor científico. ^5^ No Brasil, a primeira Diretriz sobre o consumo de gorduras foi publicada em 2013 pela Sociedade Brasileira de Cardiologia (SBC). ^6^

 Os primeiros trabalhos, publicados nos anos 1950, mostraram que o aumento do consumo de gorduras se associava significativamente a maior prevalência da aterosclerose. ^4^ Os estudos preliminares baseavam-se na análise de dados populacionais a partir da utilização de inquérito alimentar, em que se avaliava o efeito da quantidade e dos tipos de ácidos graxos saturados (SAT) e insaturados (INSAT) sobre a mortalidade e a DCV. Assim, a primeira recomendação quanto ao consumo de gorduras estabeleceu limite máximo de 30% do valor calórico total da dieta na forma de gorduras, e recomendou redução no consumo de SAT. ^4^ As diretrizes subsequentes publicadas pela AHA ^5^ e as orientações do Dietary Guidelines for Americans 2015-2020 ^7^ mantiveram a mesma linha para a prevenção cardiovascular, recomendando limite máximo de 35% do valor calórico total (VCT) da dieta, podendo variar conforme o perfil lipídico de cada indivíduo. Além disso, recomendam-se ingestão de no máximo 10% do VCT para SAT, estímulo ao consumo de INSAT e ausência de ácidos graxos *trans* da dieta. 

 Desse modo, ao recomendar dietas com menor conteúdo de gordura *(low-fat)* , a American Heart Association, na verdade, tem como objetivo propor a adequação de seu consumo. Esta indicação se baseou no fato de que o consumo de gordura pela população americana, ao longo das últimas décadas, era muito elevado (36% a 46% do VCT), e foi associado ao aumento de risco cardiovascular. Adicionalmente, apenas para indivíduos hipercolesterolêmicos, o American College of Cardiology (ACC) e a AHA ^8 , 9^ recomendam limite de 5% a 6% das calorias na forma de SAT. Neste mesmo sentido, a diretriz europeia para o controle das dislipidemias, 2019 ESC/EAS Guidelines for the management of dyslipidaemias: lipid modification to reduce cardiovascular risk, ^10^ recomenda limite do consumo de SAT (< 7% do VCT) e de gorduras totais (< 35% do VCT). 

 As investigações na área da Nutrição apresentam muitos resultados controversos, em virtude da inconsistência observada nos protocolos quanto ao tempo de estudo, população estudada, tamanho da amostra e tipo de nutriente utilizado como comparação. ^10^ A substituição das calorias provenientes de SAT por poli-insaturados (POLI) e monoinsaturados (MONO) diminui o risco cardiovascular, ^11^ enquanto resultado oposto é observado quando SAT são substituídos por carboidratos refinados como o açúcar. ^12^ Além disso, os SAT podem ser encontrados em ampla diversidade de alimentos, que variam em estrutura e composição, tais como carnes, leite, óleos e alimentos industrializados. O fato de os SAT se apresentarem em emulsões de gordura como no leite, ou em matriz sólida de óleo de palma com açúcar, como observado em biscoitos industrializados, deve induzir efeitos distintos sobre os lípides plasmáticos. ^13^

 Outro ponto importante que pode interferir na análise do papel dos ácidos graxos é o padrão alimentar no qual se inserem. Ácidos graxos SAT podem se associar a efeitos deletérios do ponto de vista cardiovascular, quando inseridos em um contexto de dieta rica em açúcares e pobre em fibras, ao passo que, no contexto de padrão alimentar saudável, seu impacto negativo pode ser menor. ^14^

 Embora, de forma genérica, SAT se associe a maior risco cardiovascular, é importante salientar que nem todos os ácidos graxos SAT elevam a concentração plasmática de colesterol e do risco cardiovascular. ^11^ Além disso, diversos estudos mostram que o aumento do seu consumo pode induzir aumento do HDLc. ^11^ No entanto, essa informação deve ser vista com cautela, uma vez que essas partículas de HDL se enriquecem com proteínas inflamatórias, podendo diminuir a sua funcionalidade e prejudicar alguma etapa do transporte reverso de colesterol. ^15^

 As diretrizes internacionais apontam para importância do seguimento de padrões alimentares saudáveis, como o Padrão Mediterrâneo ^16 - 18^ e a dieta DASH ( *Dietary Approach to Stop Hypertension* ). ^19^ O Guia Alimentar para a Populacão Brasileira ^20^ também evidencia a importância do seguimento de padrões alimentares saudáveis e enfatiza que simplesmente estudar os nutrientes de forma isolada não esclarece por completo a influência da alimentação na saúde. Explica ainda que os benefícios devem ser atribuídos menos a um alimento individualmente, e mais ao conjunto que integra o padrão alimentar. 

 Como ponto comum, todos esses documentos enfatizam a importância da adequação calórica, inclusão de grãos, frutas, hortaliças e redução de carboidratos refinados, especialmente os açúcares. Com relação às gorduras, recomendam que se priorize o consumo de MONO e POLI, acrescido de um limite de ingestão de saturados, o que converge para as orientações da AHA ^9^ quanto ao perfil lipídico recomendado para uma dieta saudável. 

 De acordo com dados recentes do estudo National Health and Nutrition Examination Survey (NHANES), houve redução do consumo de carboidratos refinados e ácidos graxos SAT pela população americana. Apesar disso, essa população ainda extrapola a quantidade recomendada desses nutrientes. ^21^

 Com relação ao Brasil, não existem dados suficientes para análise detalhada quanto à evolução do consumo percentual de gorduras. No entanto, resultados importantes da última POF/IBGE, 2019 ^22^ que comparou o período de 2017-2018 a 2002-2003, mostraram diminuição significativa nas despesas domiciliares com óleos e gorduras. Além disso, essa pesquisa mostrou redução do consumo de leguminosas (grãos). Esse levantamento também mostrou que quase um terço da população se alimenta fora do domicílio, o que aumenta a chance do consumo de alimentos em lanchonetes, os quais apresentam baixa qualidade nutricional, por seu baixo teor de fibras, vitaminas e alta concentração de gorduras e carboidratos refinados. Apesar de ter havido pequeno aumento nas despesas domiciliares com frutas, os dados da Pesquisa de Vigilância de Fatores de Risco e Proteção de Doenças Crônicas por Inquérito Telefônico (VIGITEL) mostram que apenas 24,4% da população consome frutas e hortaliças em quantidades recomendadas pelo Ministério da Saúde ^3^ e que 32% da população consome diariamente carnes com alto teor de gorduras. Além disso, alimentos ultraprocessados que têm baixo valor nutricional, como biscoitos recheados, são aqueles que mais contribuem com o consumo de SAT e açúcar (IBGE). 

 O Brasil fez parte dos 195 países que foram incluídos no estudo Global Burden of Disease Study 2017, ^1^ cujo principal objetivo foi avaliar o impacto da alimentação sobre morbidade e mortalidade associadas às doenças crônicas não transmissíveis. Entre as principais causas de mortalidade cardiovascular atribuídas à dieta, destacam-se o alto consumo de sódio e gorduras *trans* e o baixo consumo de frutas, hortaliças, grãos e alimentos fontes de ácidos graxos POLI. Nesse estudo, o principal fator de risco alimentar associado à mortalidade e morbidade cardiovascular no Brasil foi o baixo consumo de grãos, que, em nossa população, é representado principalmente pelo feijão. De fato, dados levantados por ambos os estudos, VIGITEL e POF, enfatizam que houve redução no consumo de feijão, que, além de fazer parte da cultura alimentar brasileira, integra padrão alimentar saudável, por apresentar reduzido conteúdo lipídico e significante quantidade de fibras. 

 Apesar do impacto deletério de *trans* sobre o risco cardiovascular, estudo recente conduzido no Brasil revela que um quinto dos alimentos empacotados ainda são preparados com este ácido graxo. ^23^ Além disso, outros alimentos consumidos em lanchonetes em substituição às refeições, tais como salgados fritos ou assados, folhados, tortas, entre outros, são frequentemente preparados com *trans.* Nesse sentido, a dieta praticada atualmente por parte dos brasileiros contraria as atuais recomendações internacionais sobre o seguimento de padrões alimentares saudáveis. 

Este posicionamento elaborado pela SBC tem por objetivo descrever os recentes avanços sobre as ações dos diferentes ácidos graxos, desde a sua influência na microbiota intestinal, metabolismo lipídico hepático e tecido adiposo até os principais aspectos relacionados com o risco e o controle da DCV.

## 2. Classificação E Fontes De Ácidos Graxos

### 2.1. Monoinsaturados

 Os ácidos graxos monoinsaturados (MONO) são caracterizados pela presença de uma única dupla ligação em sua cadeia carbônica. Destes, o ácido oleico (ômega-9) se apresenta como o mais abundante encontrado na natureza, e representa cerca de 90% de todos os ácidos graxos MONO, ^24^ sendo os principais óleos-fonte os de oliva e canola. Os MONO ocupam papel de destaque também na composição dos ácidos graxos de diversos frutos secos, tais como macadâmia (59%), avelã (46%), amendoim (41%), amêndoas (31%), castanha-de-caju (27%) e pistache (24%) ^25^ . Outro óleo rico em MONO é o *high oleic acid* , o qual vem sendo utilizado em alguns países e pode ser preparado a partir de óleos de girassol, canola ou soja. ^26 , 27^ Com devida atenção ao teor elevado de ácidos graxos SAT, produtos cárneos também são considerados fontes importantes de MONO, respondendo, em alguns casos, entre 40% e 50% da composição de alimentos como carne de boi, frango ^28^ e porco. ^29^

### 2.2. Poli-insaturados

 Os ácidos graxos poli-insaturados (POLI) fazem parte de um amplo grupo de gorduras com duas ou mais duplas ligações em sua cadeia carbônica. Tal característica implica em funções biológicas amplamente distintas e, portanto, seu impacto na saúde cardiovascular também apresenta especificidades diretamente relacionadas com o tipo de POLI consumido. Fazem parte da série ômega-6 (ω6) ou ômega-3 (ω3), em função da localização da primeira dupla ligação na cadeia carbônica a partir do terminal metila. Os ácidos graxos da série ω6 classificam-se em linoleico (18:2), cujas principais fontes são óleos (girassol, milho e soja), nozes e castanha-do-pará, e ácido araquidônico (20:4), obtido a partir da conversão endógena do ácido linoleico. Os principais ácidos graxos da série ω3 são o ácido alfalinolênico (ALA [C18:3]) de origem vegetal, cujas fontes principais são a soja, a canola, a linhaça e a chia, ^30 , 31^ e os ácidos eicosapentaenoicos (EPA [C20:5]) e docosaexaenoico (DHA [C22:6]), provenientes de peixes e crustáceos de águas muito frias dos oceanos Pacífico e Ártico. Os ácidos graxos linoleico e linolênico são considerados essenciais aos seres humanos, sendo necessária sua aquisição por meio da alimentação. No entanto, de acordo com Dietary Reference Intakes (DRI), sua suplementação não é necessária, uma vez que a ingestão moderada de óleo de soja ou canola (cerca de 15 mL/dia) garante o consumo adequado. ^32^ Já os ácidos EPA e DHA podem ser produzidos endogenamente por ação enzimática de dessaturases e elongases sobre o ALA, porém essa conversão é limitada e sofre interferência de fatores fisiológicos e externos. ^33 - 35^ Outra fonte de EPA e DHA é o óleo de krill, crustáceo semelhante ao camarão, encontrado nos mares do Sul. O óleo de krill é fonte singular de EPA e DHA, pois a maior parte dos ácidos graxos ω3 encontra-se em fosfolípides, apresentando maior biodisponibilidade de ω3 do krill em comparação ao ω3 marinho. ^36^

### 2.3. Saturados

 Os ácidos graxos SAT apresentam estrutura molecular simples e caracterizam-se pela ausência de duplas ligações em suas cadeias carbônicas retilíneas. São classificados em cadeia curta (ácido acético [C2:0]; ácido propiônico [C3:0] e butirato [C4:0]), média (caproico [C6:0]; caprílico [C8:0]; cáprico [C10:0]) e longa (láurico [C12:0]; mirístico [C14:0]; palmítico [C16:0]; esteárico [C18:0]). ^37^ Além disso, classificam-se também em função do seu ponto de fusão, característica fundamental para a determinação de sua forma de absorção. Os ácidos graxos de cadeia curta e média (C2-C10), que apresentam baixo ponto de fusão são absorvidos via sistema porta, enquanto os de cadeia longa (C14-C18) são absorvidos via sistema linfático, por meio dos quilomícrons. Já o láurico é absorvido em grande parte pelos quilomícrons, mas também via sistema porta. ^38^

 Essa diferença estrutural confere aos saturados diferentes ações biológicas e metabólicas, ^39^ atuando como agentes sinalizadores modulando a interação proteína-proteína e proteína-membrana plasmática por meio de processos conhecidos por miristoilação e palmitoilação de proteínas. ^40^

 Os SAT podem ser sintetizados endogenamente na maioria das células a partir de acetilcoenzima A (acetil-CoA) proveniente do metabolismo de carboidratos, aminoácidos e gorduras. ^41^ Dentre eles, o mais abundante é o ácido palmítico (carnes e óleo de palma), seguido do esteárico (cacau), mirístico (leite e coco) e, em pequena quantidade, ácido láurico (coco). As principais fontes dietéticas de ácido palmítico são as carnes e o óleo de palma. ^42 , 43^

### 2.4. Trans

 A principal fonte de *trans* na dieta é o ácido elaídico (18:1, n-9 *t* ), presente nas gorduras vegetais preparadas a partir da hidrogenação parcial de óleos vegetais, as quais são amplamente utilizadas pela indústria alimentícia. ^44^ Encontram-se também em pequenas quantidades em carnes e leite na forma de vacênico (18:1, n-11 *t* ), o qual é sintetizado por meio da bio-hidrogenação de gorduras sob ação microbiana em animais ruminantes. ^44^

## 3. Concentração Plasmática de Colesterol Total e Lipoproteínas

 A recomendação de redução do consumo de saturados se deve ao fato de estes elevarem as concentrações plasmáticas de LDLc. ^45^ Já se demonstrou que o consumo de SAT tem correlação linear com as concentrações de lípides plasmáticos, e eleva as concentrações de colesterol total (CT), LDLc e HDLc, conforme demonstrado no documento da WHO. ^11^ Uma das publicações do estudo PURE (Prospective Urban Rural Epidemiology), que avaliou a associação entre a dieta com os lípides plasmáticos em mais de 100 mil pessoas, também revelou aumento da concentração plasmática de CT, LDLc e HDLc. ^46^ Neste estudo, os autores mostraram que houve linearidade entre o consumo de SAT e a elevação dos lípides plasmáticos quando se comparou o maior quintil de consumo (> 11,2% do VCT) ao menor (< 4,03% VCT). 

 Importante salientar que os SAT elevam todas as classes de lipoproteínas, mas a elevação observada no HDL pode não ser suficiente para suplantar os efeitos deletérios do LDL sobre o risco cardiovascular. ^47^ Os diferentes SAT exercem efeitos diversos no perfil lipídico e, portanto, no risco cardiovascular. Quando comparado ao carboidrato, o mirístico (C14:0) é o que mais aumenta as concentrações de CT e LDLc, seguido de palmítico (C16:0) e ácido láurico (C12:0), efeito não observado com o ácido esteárico. ^11^ Isso se deve ao fato de o esteárico ser rapidamente convertido a oleico no fígado, pela estearoil-CoA dessaturase 1 (SCD1). ^48^ Já em relação ao HDLc, os ácidos graxos mirístico, láurico e palmítico elevam suas concentrações quando se avalia a substituição isocalórica de carboidratos. ^11^

 A ação dos SAT sobre o colesterol plasmáticos ocorre por diferentes mecanismos. Em 1969, Spritz e Mishkel ^49^ demonstraram que, em virtude de sua cadeia carbônica retilínea, os ácidos graxos SAT podem ser empacotados no centro das lipoproteínas, permitindo que estas carreiem maior quantidade de colesterol. ^49^ Posteriormente, demonstrou-se que o SAT, em associação com colesterol, é capaz de reduzir atividade, proteína e RNAm do receptor de LDL, ^50 , 51^ prejudicando assim o *clearance* das partículas de LDL. ^52 , 53^ Além disso, o consumo de SAT eleva o RNAm da hidroximetilglutaril coenzima A redutase (HMG-CoA redutase), fosfomevalonatoquinase e lanosterolsintase – enzimas importantes na via de síntese do colesterol. ^54^

 Estudo realizado nas coortes do Nurses’ Health Study (NHS) (1984-2012) e Health Professionals Follow-up Study (HPFS) (1986-2010) demonstrou que a substituição isocalórica de 1% das energias provenientes de láurico, palmítico ou esteárico por POLI ou MONO reduziu o risco de doença coronariana. ^55^ Esse efeito está associado ao impacto dos INSAT nos lípides plasmáticos, que reduz as concentrações de LDLc, podendo reduzir também as concentrações de HDLc. ^56^ Estudo de metanálise demonstrou que, para cada 1% de substituição das calorias proveniente de SAT por POLI, há redução das concentrações plasmáticas de CT, LDLc, HDLc, apolipoproteína A-I (ApoA-I) e apolipoproteína B (ApoB). Quando a substituição isocalórica é feita por MONO, observam-se reduções nos lípides plasmáticos mais modestas, porém significativas, nas concentrações de CT, LDLc, HDLc e ApoB. ^11^

 Revisão de estudos observacionais e de intervenção concluiu que a substituição de SAT por POLI reduz LDLc e, assim, o risco de DCV. ^57^ Estudo de coorte prospectivo envolvendo 84.628 mulheres e 42.908 homens evidenciou que a substituição isocalórica de saturados (5% VCT) por carboidratos complexos foi associada à redução de 11% no risco de doença coronariana. ^58^ Por outro lado, o estudo de intervenção Woman’s Health Initiative (WHI), que avaliou o efeito da redução do consumo de gorduras e aumento no consumo de vegetais, frutas e grãos no desfecho cardiovascular, não observou efeito da dieta na redução no risco cardiovascular (CV). ^59^ No entanto, a redução do LDLc alcançada com a intervenção foi pequena, assim como a diminuição no consumo de saturados (apenas 2,9% comparado ao controle). A redução no consumo total de gordura reduziu também o consumo de MONO E POLI. ^59^ Observou-se ainda que a substituição isocalórica de SAT por carboidratos, reduz CT, LDLc, HDLc, ApoA-I e ApoB. ^11^

 Duas importantes metanálises que avaliaram ensaios clínicos apontaram efeito neutro dos MONO sobre a concentração plasmática de lípides. ^60 , 61^ Mais recentemente, outra revisão sistemática, a qual realizou análise de regressão em estudos de intervenção, demonstrou que a substituição de 1% das calorias provenientes de SAT pelo equivalente calórico em MONO reduziu significativamente as concentrações plasmáticas de CT, LDLc e HDLc. ^11^ Por outro lado, a substituição isocalórica de carboidratos por MONO eleva as concentrações de HDLc, efeito que diminui com o aumento da insaturação do ácido graxo. ^62^ Demonstrou-se ainda que dieta rica em MONO (20% do VCT) reduziu a concentração plasmática de CT, LDLc, LDL pequenas, LDLox e HDLc. ^63^ Em outro estudo, conduzido em indivíduos com sobrepeso, o aumento no consumo de MONO (de 7% para 13% do VCT) também contribuiu para redução de CT e LDLc, mas sem alteração no HDLc. ^64^ Em linhas gerais, o consumo adequado de MONO tem mostrado efeito positivo sobre o metabolismo lipídico com efeitos contrários aos observados SAT. 

 Já se demonstrou que a substituição de 1% das calorias provenientes de SAT por ω6 reduz o CT em 2 mg/dL, enquanto o impacto no HDLc é mínimo. ^56^ Importante metanálise de estudos epidemiológicos observacionais apontam para o efeito redutor de colesterol pelo ω6 quando consumidos em substituição a ácidos graxos SAT e *trans* em humanos. ^56^ Foi demonstrado que a substituição de 10% das calorias provenientes dos ácidos graxos SAT pelo ω6 se associa à redução de 18 mg/dL no LDLc, impacto maior que o observado com reposição isocalórica de carboidratos. Além disso, a concentração plasmática elevada de ω6 se associou à redução da razão CT/HDLc. ^56^

 O aumento no consumo de ω6 foi associado à pequena redução na concentração plasmática de CT, e pouco ou nenhum efeito foi observado nas concentrações de HDLc ou LDLc. Portanto, evidências não são suficientes para se propor suplementação de ω6 na prevenção cardiovascular primária e secundária. ^65^

 Com relação aos ácidos graxos ω3, os resultados de uma revisão sistemática mostraram dados inconsistentes do efeito de ALA sobre o colesterol plasmático. ^66^ Metanálise de estudos randomizados não observou influência significativa da suplementação com ALA sobre CT e LDLc, tendo efeito mínimo sobre o HDLc (redução de 0,4 mg/dL). ^67^ Já o DHA se associou com elevação do LDLc, ^66^ e o mesmo resultado foi observado com a suplementação com óleo de peixe. ^68^ Essa elevação do colesterol é provavelmente atribuída à diminuição da expressão do SREBP-2, que regula a síntese do receptor de LDL, ^69 , 70^ induzida de forma dose-dependente pelo DHA. 

 Outro estudo demonstrou que dietas enriquecidas com ALA ou com EPA/DHA não promoveram alteração no perfil lipídico quando se comparou à dieta rica em MONO. ^71^ Resultado semelhante foi observado quando se avaliou o efeito de óleo enriquecido com EPA, DHA e ALA. ^72^ Nesse estudo, observou-se efeito benéfico sobre os lípides plasmáticos apenas no período inicial ( *wash in* ), no qual os indivíduos que consumiam dieta rica em saturados foram submetidos à dieta rica em MONO. ^72^ É importante enfatizar que, ao analisar o efeito dos ácidos graxos ω3 sobre a colesterolemia, deve-se considerar o tipo de comparação feita no estudo, uma vez que os INSAT, quando utilizados em substituição a dietas ricas em SAT, promovem efeitos benéficos; por outro lado, a suplementação mostra resultados distintos. 

 Entre os ácidos graxos, o *trans* apresenta maior efeito aterogênico, por sua robusta ação sobre a colesterolemia. ^73^ Importante metanálise ^56^ de estudos randomizados evidenciou as ações deletérias desses ácidos graxos sobre as concentrações plasmáticas de CT, LDLc e VLDLc. Além disso, o *trans* apresenta efeito adverso adicional por reduzir as concentrações plasmáticas de HDLc em comparação aos saturados. ^74 - 77^ A redução do HDLc se deve ao aumento do catabolismo de ApoA-I. ^74 , 75^ Adicionalmente, os ácidos graxos *trans* elevam a atividade da proteína de transferência de colesterol esterificado (CETP; *cholesterol ester transfer protein* ), proteína envolvida na transferência de colesterol éster (CE) e triglicérides (TG) entre as lipoproteínas do plasma, enriquecendo de CE as partículas ricas em ApoB. Por outro lado, as partículas de HDL tornam-se mais ricas em TG, favorecendo o seu catabolismo. ^78^ Outra ação deletéria de *trans* é a redução do *clearance* das partículas contendo ApoB100, elevando a sua concentração no plasma ^75^ o que contribui com a formação de partículas de LDLs pequenas e densas que são mais aterogênicas. ^79^ Metanálise de estudos randomizados e controlados mostrou que a cada 1% do consumo de energia na forma de trans em substituição isocalórica a SAT, MONO e POLI, aumentou tanto a relação CT/HDL quanto a razão ApoB/ApoA-I. ^80^ Dessa forma, reconhecendo o seu impacto negativo no perfil lipídico, as diretrizes nacionais e internacionais recomendam sua exclusão da dieta. ^7 , 8 , 20^

## 4. Concentração Plasmática de Triglicérides

Os ácidos graxos exercem ações distintas sobre a trigliceridemia, por modularem os fatores de transcrição envolvidos na síntese de enzimas lipogênicas envolvidas na produção de ácidos graxos.

 Os SAT são capazes de modular genes envolvidos na síntese de lípides. Já se demonstrou que os SAT induzem a expressão hepática do PGC-1β *(peroxisome proliferator-activated receptor gamma coactivator 1β* ), que, por sua vez, ativa o SREBP ( *regulatory element-binding proteins* ), fator de transcrição envolvido na transcrição de genes das enzimas lipogênicas como a Acetil-CoA carboxilase-1 (ACC) e da ácido graxo sintase (FAS), ^81^ relacionados com a síntese de ácidos graxos, favorecendo maior produção de TG. ^54^ Além disso, SAT aumenta o processamento do SREBP e sua translocação para o núcleo da célula, induzindo a transcrição de genes-alvo. ^82^

 Revisão sistemática publicada pela WHO ^11^ revelou que, a cada 1% de substituição das calorias proveniente de SAT por POLI ou MONO, observa-se redução na concentração plasmática de TG (0,88 mg/dL e 0,35 mg/dL, respectivamente). Já a substituição de SAT por carboidratos aumentou a concentração plasmática de TG em 0,97 mg/dL. ^11^

 Por outro lado, sabe-se que os ácidos graxos POLI estão envolvidos na redução da concentração plasmática de triglicérides por bloquear o SREBP – efeito mais pronunciado pelos ácidos graxos da série ω3. ^83^

 Com relação à ação do ALA sobre a trigliceridemia, estudo em animais experimentais observou efeito nulo a discreto com o uso da linhaça. ^84^ Em humanos, resultados de revisão sistemática demonstraram que o efeito redutor no TG se deve ao consumo de grandes quantidades de óleo de linhaça. ^66^ Metanálise de 14 ensaios randomizados e controlados não observou influência significativa da suplementação com ALA sobre as concentrações plasmáticas de TG. ^67^ De forma semelhante, o aumento no consumo de ω6 não foi associado à redução das concentrações plasmáticas de TG. ^65^

 Estudos clínicos mostram que a suplementação com 2 a 4 g/dia de EPA e DHA pode reduzir a concentração plasmática de TG entre 25% e 30%. ^66 , 85 , 86^ A suplementação com EPA ou DHA por 4 semanas em indivíduos saudáveis reduziu a as concentrações pós-prandiais de TG, ApoB48 e ApoB100 (16%, 28% e 24%, respectivamente), possivelmente em razão do aumento da atividade da lipoproteína lipase (LPL). ^87^

 Uma das razões pelas quais os ácidos graxos POLI reduzem a trigliceridemia está relacionada com sua capacidade de reduzir a expressão e a atividade de SREBP1. ^81^ Em modelos animais e *in vitro* , tanto EPA quanto DHA diminuem SREBP1, reduzindo a expressão de enzimas lipogênicas. ^88 , 89 , 90^

 A capacidade dos ácidos graxos ω3 em reduzir TG parece ser dose-dependente, com reduções de cerca de 5% a 10% para cada 1 g de EPA/DHA consumido ao dia, sendo maior nos indivíduos com concentrações basais mais elevadas de TG. ^91 - 93^ Estudo realizado com indivíduos com valores limítrofes ou elevados de TG que receberam óleo de krill de 1 a 4 g/dia, por 6 semanas, mostrou redução das concentrações plasmáticas de TG (18,6 a 19,9 mg/dL). Já na suplementação com 0,5 g/dia, a redução de TG foi de 13,3 mg/dL. ^36^

## 5. Doença Cardiovascular e Coronariana

### 5.1. Saturados

 Apesar das importantes ações biológicas que desempenham, o alto consumo de SAT tem efeito deletério no metabolismo de lípides e risco cardiovascular, ^94 , 95^ pois elevam as concentrações plasmáticas de LDLc, um dos principais fatores de risco para o desenvolvimento de aterosclerose e, consequentemente, DCV. ^11^ Extensa revisão sistemática conduzida pela Biblioteca Cochrane, em 2015, mostrou que a diminuição do consumo de saturados reduziu em 17% os eventos cardiovasculares, comparado com a dieta habitual. ^96^ Além disso, análise de um subgrupo de estudos da mesma metanálise que avaliou a substituição de SAT por POLI mostrou redução de 27% nos eventos cardiovasculares. Por essa razão, as recomendações nutricionais para redução do risco cardiovascular contemplam redução do consumo de SAT. 

 Entretanto, nos últimos anos, metanálises e estudos observacionais obtiveram conclusões discordantes sobre a relação entre o consumo de gordura saturada e risco cardiovascular. ^12 , 94 , 96 , 97 - 99^ Essa discrepância se deve, em parte, ao macronutriente utilizado na substituição da gordura saturada, uma vez que, a redução de um dos macronutrientes da dieta acarreta aumento de outro. ^100^ Metanálises de estudos observacionais prospectivos que avaliaram o efeito da gordura saturada sobre a ocorrência de eventos cardiovasculares, sem considerar o tipo de macronutriente utilizado para substituir as calorias provenientes de SAT, não observaram efeito do consumo de saturados sobre o risco cardiovascular. ^98 , 101^ Por outro lado, a substituição do consumo de saturados por POLI ou carboidratos complexos provenientes de grãos integrais mostrou-se benéfica e foi associada a menor risco de doença coronariana. Já a substituição de saturados por carboidratos simples não apresentou impacto sobre o risco de eventos cardiovasculares, ^97 , 99^ em virtude do alto consumo de açúcar ser deletério do ponto de vista CV. 

 O estudo PURE, realizado em 18 países, avaliou a associação entre componentes da dieta e mortalidade total e eventos cardiovasculares, e mostrou que o risco de mortalidade total e por causas não cardiovasculares se correlacionou positivamente ao maior consumo de carboidratos e negativamente ao maior consumo de gorduras (POLI, MONO e SAT) e proteínas (do VCT). Vale ressaltar que o maior consumo de gorduras e de SAT foi de 35% e 13% do VCT, respectivamente, e a mediana do maior consumo de carboidrato chegou a 77% do VCT. Além disso, o maior consumo de gorduras saturadas foi associado a menor risco de acidente vascular cerebral (AVC). O consumo total de gordura, assim como de gordura SAT e INSAT, não se associou ao risco de infarto e morte cardiovascular. ^102^ Além disso, o tipo de carboidrato consumido não foi analisado separadamente, porém foi observado que, nos países de baixa e média renda *per capita* , esse consumo era principalmente de refinados. Análise posterior mostrou que o consumo total de gordura e SAT se correlacionou com aumento das concentrações plasmáticas de CT e LDLc. ^46^ Em 2018, ainda nessa mesma coorte, foi mostrado que o consumo de lácteos se associou negativamente com mortalidade total e cardiovascular, DCV e AVC. ^103^

 Estudos randomizados avaliaram os efeitos de intervenções dietéticas sobre a ocorrência de eventos cardiovasculares; entretanto, as diferenças no consumo total de gorduras entre grupo intervenção *versus* controle são pouco substanciais na maioria dos estudos. ^59 , 104 , 105^ O Women’s Health Initiative acompanhou, por cerca de 8 anos, 48.835 mulheres que foram randomizadas para mudança de estilo de vida (redução do consumo de gorduras para 20% do VCT e aumento de vegetais e grãos) ou grupo controle (orientações por materiais educacionais). Após 6 anos de seguimento, a intervenção não reduziu a ocorrência de doença arterial coronária ou AVC, apesar da diminuição significativa na ingestão total de gorduras. ^59^

 Estudo de coorte prospectivo evidenciou que o maior consumo de gordura saturada foi associado a menor risco de doença cardíaca isquêmica, porém, não ao risco de doença coronária. ^106^ Em outra coorte, a ingestão de ácido palmítico, mas não a ingestão de gordura saturada total, foi positivamente associada ao risco de doença arterial coronária (DAC). ^107^

 Estudos recentes mostraram que os diferentes tipos de saturados apresentam efeito cardiometabólico heterogêneo e se correlacionam de forma distinta com o risco CV, doença coronariana e incidência de diabetes melito tipo 2 (DM2). Nesse contexto, os ácidos láurico, mirístico, palmítico e esteárico estão associados com aumento do risco de doença coronariana ^55 , 108^ e DM2, ^14 , 109^ enquanto ácidos graxos pentadecílico (15:0) ^110^ e magárico (17:0) estão associados ao consumo de lácteos, bem como os saturados de cadeia longa (20:0 a 24:0) se correlacionam inversamente com incidência de DCV e DM2. ^14 , 110^

### 5.2. Substituição de Saturados por Insaturados

 Estudo de coorte prospectivo que investigou 83.349 mulheres e 42.884 homens, de 1986 a 2012, mostrou que a substituição isocalórica de 5% das energias provenientes de saturados por MONO ou POLI foi associada à redução na mortalidade total estimada em 13% e 27%, respectivamente. Além disso, a substituição de SAT por POLI reduziu o risco de mortalidade por DCV, câncer e doenças neurodegenerativas ^93^ . Estudos de intervenção mostraram que a substituição isocalórica de 10% dos SAT por POLI reduz o risco de eventos cardiovasculares em 27% ^111^ e substituição de 5% reduz o risco de DAC em 10%. ^94^ A substituição isocalórica (1% do VCT) de saturados (12:0 a 18:0) por carboidratos complexos reduziu o risco de doença coronariana, conforme demonstrado na análise dos estudos HPS (Health Professional Study) e o NHS. ^55^

### 5.3. Substituição de Saturados por Carboidratos

 Estudo de coorte prospectivo envolvendo 84.628 mulheres e 42.908 homens evidenciou que a substituição isocalórica de saturados (5% VCT) por carboidratos complexos foi associada à redução de 11% no risco de doença coronariana. ^58^ Da mesma forma, a substituição isocalórica de apenas 1% do VCT sob a forma de SAT (12:0 a 18:0) por carboidratos complexos reduziu o risco de doença coronariana. ^55^

 Por outro lado, estudo de intervenção, que avaliou o efeito da redução do consumo de gorduras e aumento no consumo de vegetais, frutas e grãos no desfecho cardiovascular, não observou efeito da dieta na redução no risco CV. ^59^ No entanto, a redução do LDLc (2,7 mg/dL) alcançada com a intervenção foi pequena, assim como a diminuição no consumo de saturados (apenas 2,9% comparado ao controle). Vale ressaltar que redução no consumo total de gordura reduziu também o consumo de MONO e POLI, associados a perfil lipídico favorável do ponto de vista cardiovascular. ^59^

 Em relação aos lípides plasmáticos, demonstrou-se que a substituição isocalórica de SAT por carboidratos, reduz o CT (1,58 mg/dL), LDLc (1,27 mg/dL), HDLc (0,38 mg/dL), ApoA-I (7,0 mg/dL) e ApoB (3,6 mg/dL); por outro lado, aumenta as concentrações de triglicérides (0,97 mg/dL). ^11^

 Com relação aos ácidos graxos MONO, diversos estudos baseados na dieta do Mediterrâneo têm mostrado efeitos positivos na prevenção dos fatores de risco e desfechos cardiovasculares. Na dieta do Mediterrâneo, o azeite de oliva é a principal fonte de MONO, seguida por nozes e castanhas, as quais também fornecem POLI. Cabe ressaltar que esse padrão alimentar inclui hortaliças, frutas e grãos que também podem conferir benefícios para a saúde cardiovascular. ^112^

 O estudo PREDIMED acompanhou mais de 5.000 indivíduos com alto risco cardiovascular por 5 anos, submetidos à dieta Mediterrânea suplementada com 50 g/d de azeite de oliva extravirgem ou 30 g/d de frutas oleaginosas, ambos comparados com aqueles que consumiam dieta com menor conteúdo de gorduras (30% do VCT). Os resultados mostraram que ambos os grupos apresentaram menos eventos cardiovasculares (RR = 0,83). ^17^ Resultados semelhantes também foram observados com o consumo de azeite de oliva nos estudos NHS (1980-2010, n = 84.628 indivíduos, HR = 0,85), Health Professional Follow-up Study (1986-2010, n = 42.908, HR = 0,85), Alpha-Tocopherol, Beta-Carotene Cancer Prevention Study (ATBC) (RR = 0,92) e EPIC Study (HR = 0,87). ^113^ Um dos braços do estudo EPIC, conduzido na população holandesa, mostrou risco aumentado para doença isquemica cardíaca associado ao consumo de MONO (HR = 1,30). ^106^ Entretanto, cabe ressaltar que, nesse estudo, os autores identificaram importantes fatores de confusão capazes de interferir na compreensão final do desfecho, pois não diferenciaram ácidos graxos MONO na forma *cis* e *trans.* ^106^

 A revisão dos estudos publicados pelo grupo da Cochrane mostrou que a eficácia da substituição de SAT por MONO sobre eventos cardiovasculares é incerta em virtude do pequeno número de estudos incluídos. ^96^ Já o Guia Alimentar para Americanos ^7^ afirma que a substituição de SAT por MONO foi associada com redução de risco cardiovascular, embora as evidências não tenham sido tão fortes. Estudo de coorte publicado posteriormente mostrou que a substituição de 5% das calorias na forma de SAT por MONO reduziu em 15% o risco CV. ^58^

### 5.4. Ácidos Graxos Poli-insaturados (Ômega-6)

 Com relação aos efeitos dos INSAT da série ω6 sobre o risco cardiovascular, estudos clínicos randomizados e observacionais fornecem evidências de que a substituição ao redor de 5% a 10% VCT sob a forma de saturados e carboidratos refinados (como açúcar, pão branco, arroz branco) por ω6 reduz o risco de DCV, sem evidências clínicas de eventos adversos. ^114 - 117^ Já a substituição de 1% do VCT oriundo de SAT por ω6 tem sido associada à redução de 2% a 3% na incidência de doença coronariana. ^94 , 118^ Esse benefício ainda pode estar subestimado em razão da grande quantidade de ácidos graxos SAT em alguns alimentos que também são fontes ω6. 

 Importante revisão sistemática, que avaliou estudos de coorte prospectivos e ensaios clínicos randomizados envolvendo indivíduos em prevenção primária e secundária, mostrou que o consumo de ω6 não se associou a menor risco de doença arterial coronariana, ao contrário do que foi observado para a ingestão de peixes ou ω3 marinho. ^93^ De fato, vários estudos mostram que a redução nos desfechos cardiovasculares é menor com a substituição dos ácidos graxos SAT por ω6 do que quando comparada à substituição pelo conjunto ω6 e ω3. ^119^

 A biblioteca Cochrane publicou revisão com ensaios clínicos que avaliaram o efeito do consumo de ω6 na prevenção primária de DCV e concluiu que o consumo de ácidos graxos ω6 (linoleico, gama-linolênico, di-homo-gamalinolênico e araquidônico) não interferiu nos marcadores lipídicos e de pressão arterial; entretanto, nenhum dos trabalhos avaliou desfechos clínicos. ^65 , 120^ Em revisão mais recente, também conduzida pela biblioteca Cochrane, na qual se avaliou o efeito da suplementação com ω6 em fatores de risco (pressão arterial, perfil lipídico e adiposidade) e desfecho cardiovascular (mortalidade geral, mortalidade cardiovascular e eventos cardiovasculares). Foi observado pouco ou nenhum benefício das intervenções com ω6 sobre a mortalidade total (RR = 1,00; IC = 0,88 a 1,12), mortalidade cardiovascular (RR = 1,09; IC = 0,76 a 1,55) e eventos cardiovasculares (RR = 0,97; IC = 0,81 a 1,15). ^65^ De modo semelhante, o consumo de ω6 não foi associado ao menor risco de eventos cardíacos e cerebrovasculares (RR = 0,84; IC = 0,59 a 1,20) e AVC (RR = 1,36; IC = 0,45 a 4,11). Entretanto, foi observada discreta redução no risco para infarto agudo do miocárdio (IAM) com o aumento no consumo de ω6 (RR = 0,88; IC = 0,76 a 1,02). ^65^

 A maior concentração plasmática de ω6 associou-se a menor risco de eventos cardiovasculares, AVC isquêmico e mortalidade cardiovascular, conforme os resultados de recente estudo que analisou os dados de 30 estudos prospectivos, totalizando 68.659 participantes. ^121^ Nessa publicação, os autores reiteram os benefícios cardiovasculares do consumo do ω6. 

### 5.5. Ácidos Graxos Poli-insaturados (Ômega-3 Marinho)

 Os ácidos EPA e DHA têm sido investigados quanto ao seu potencial na redução do risco cardiovascular. Os mecanismos propostos para os benefícios cardiovasculares incluem redução de marcadores inflamatórios e da agregação plaquetária, melhora da função endotelial, redução da pressão arterial e redução da trigliceridemia. ^122 - 124^ Os ácidos graxos ω3 de origem marinha, DHA e EPA exercem inúmeros efeitos sobre diferentes aspectos fisiológicos e do metabolismo, o que pode influenciar a chance de desenvolvimento de DCV. 

 Apesar de evidências antigas sugerirem efeito protetor de peixes e dos ácidos graxos ω3 de origem marinha sobre eventos cardiovasculares, sobretudo em indivíduos que já apresentavam DCV, ^125 - 127^ os estudos mais recentes não mostraram benefícios da suplementação com ω3 em sujeitos que já haviam apresentado manifestações de doença aterosclerótica. ^128 - 130^ Uma das possíveis razões relaciona-se com o perfil da população estudada, especialmente no que se refere ao uso mais frequente de medicamentos sabidamente protetores (p. ex., estatinas, betabloqueadores, inibidores da enzima de conversão da angiotensina - ECA), ao controle mais agressivo dos fatores de risco tradicionais e ao maior número de procedimentos de revascularização nos estudos mais contemporâneos. Dessa forma, questiona-se se os ácidos graxos ω3 podem trazer reais benefícios adicionais quando o paciente é manejado de acordo com as recomendações atuais. Questões envolvendo forma, dose e tempo de suplementação também podem ser levantadas. Nos estudos Alpha Omega ^128^ e SU.FOL.OM3, ^130^ a dose de EPA+DHA (400 a 600 mg/dia) pode ter sido insuficiente para se observar benefício clínico. 

 Recente metanálise de ensaios clínicos randomizados e controlados avaliou a associação entre consumo de EPA+DHA e risco de DAC. Nesse estudo, foi conduzida metanálise de estudos prospectivos de coorte para verificar associação entre consumo de EPA+DHA e risco de DAC. Entre esses estudos, houve benefício significante apenas nas populações de maior risco, incluindo aqueles com hipertrigliceridemia. Os resultados dos estudos prospectivos de coorte mostraram significativa redução de risco de qualquer evento coronário quanto maior o consumo de EPA+DHA. Assim, o consumo de EPA+DHA parece associado à redução de risco de eventos coronários com maior benefício em populações de maior risco nos estudos controlados randomizados. ^131^

 No entanto, os dados para diferentes formulações de ω3 e as populações estudadas parecem contribuir para os resultados. Dois recentes estudos controlados mostraram dados contraditórios, porém houve diferenças na dose e na formulação de ω3 utilizada. O estudo ASCEND (A Study of Cardiovascular Events in Diabetes), ^132^ que avaliou 15.840 pacientes com DM, sem evidências de DCV, não demonstrou diferenças significantes entre aqueles que consumiram 1,0 g de EPA+DHA e os que receberam placebo. Revisão realizada pela Cochrane incluiu 79 ensaios clínicos, abrangendo 1.120.059 indivíduos com seguimento de 12 a 72 meses, e mostrou que EPA, DPA e DHA tiveram pouco ou nenhum efeito na mortalidade total (RR = 0,98; IC = 0,90 a 1,03), mortalidade cardiovascular (RR = 0,95; IC = 0,87 a 1,03) e eventos cardiovasculares (RR = 0,99; IC = 0,94 a 1,04). ^133^

 Já no estudo Reduction of Cardiovascular Events with Icosapent Ethyl – InterventionTrial (REDUCE-IT) ^134^ em pacientes de alto risco com triglicérides elevados, em uso de estatinas, o risco de eventos isquêmicos, incluindo morte cardiovascular, foi significantemente menor naqueles que receberam 2 g de icosapenta-etil éster 2 vezes/dia (dose total de 4 g/dia), em comparação aos que receberam placebo. De um total de 8.179 pacientes (70,7% em prevenção secundária) seguidos por 4,9 anos (mediana), houve redução de 25% no risco do desfecho primário composto (HR, 0,75; IC 95%: 0,68 a 0,83; P < 0,001) dos desfechos secundários-chave (HR, 0,74; IC 95%: 0,65 a 0,83; P < 0,001) e de eventos pré-especificados, incluindo mortalidade cardiovascular (HR, 0,80; IC 95%: 0,66 a 0,98; P = 0,03). Houve, no entanto, maior taxa de hospitalizações por fibrilação atrial ou *flutter* no grupo recebendo EPA, sem diferenças no risco de sangramento. Vale ressaltar, contudo, que o *Icosapent Ethyl* não é um ácido graxo de origem alimentar, e sua indicação, em doses farmacológicas, é feita a critério médico. 

Desta forma, embora exista consenso de que o consumo regular de peixes ricos em ácidos graxos ω3 deva fazer parte de uma dieta saudável, ainda não há recomendação segura para a suplementação de cápsulas de óleo de peixe. Isso se deve ao fato de o assunto ainda estar cercado por controvérsias, fomentadas por resultados conflitantes de estudos clínicos.

 Em modelos experimentais de aterosclerose em camundongos, vários estudos relatam que óleo de peixe e EPA atenuam o processo aterosclerótico, embora o mesmo não tenha sido demonstrado em outras condições experimentais. ^135 - 140^ Alguns estudos populacionais sugerem associação inversa entre consumo de peixe ou ácido graxo ω3 marinho e marcadores de aterosclerose subclínica, como espessura médio-intimal de carótida e calcificação coronariana, embora tal relação pareça ser sutil. ^141 - 143^ Em um ensaio randomizado com pacientes com DAC, a suplementação com aproximadamente 1,5 g/dia de ácido graxo ω3 por 2 anos provocou menos progressão e mais regressão da aterosclerose coronariana, medida por angiografia invasiva quantitativa, em relação ao uso de placebo, embora as diferenças tenham sido pequenas. ^144^ No entanto, em outro estudo, a suplementação não modificou a evolução da aterosclerose carotídea avaliada por ultrassonografia, ^145^ o que contrasta com os resultados do estudo randomizado proposto por Mita et al., ^146^ no qual EPA altamente purificado (1,8 g/dia) atenuou a progressão do espessamento médio-intimal de carótida em diabéticos. ^146^

 É possível também que o ácido graxo ω3 exerça papel protetor de eventos cardiovasculares através da modulação das características da placa aterosclerótica, tornando-a mais estável. Um estudo randomizado com pacientes aguardando endarterectomia de carótida, a suplementação com óleo de peixe mostrou que os ω3 rapidamente se incorporam à placa aterosclerótica e podem induzir modificações compatíveis com um perfil menos vulnerável a fenômenos de ruptura e instabilização, ^147^ observação consistente com achados experimentais. ^139^

#### 5.5.1. Efeitos sobre Doença Vascular Periférica

 A despeito da extensa investigação sobre os efeitos dos ácidos graxos ω3 na melhora da função vascular, seus efeitos nos desfechos cardiovasculares em indivíduos com doença arterial periférica são menos descritos. Metanálise que incluiu 5 estudos com o total de 396 indivíduos, publicados entre 1990 e 2010, foi conduzida para avaliar essa questão. ^148 - 152^ Em indivíduos com doença vascular periférica, não existem evidências suficientes para a recomendação de ácidos graxos ω3 na redução de eventos cardiovasculares maiores, necessidade de revascularização ou amputação, melhora da dor às caminhadas ou melhora da qualidade de vida. ^153^

#### 5.5.2. Efeitos sobre Arritmias Cardíacas e Morte Súbita

 Estudos experimentais mostraram efeitos antiarrítmicos do ω3, atribuídos especialmente a um efeito direto sobre canais iônicos. ^154^ Outros mecanismos relatados incluem modulação do tônus autonômico (melhora da variabilidade da frequência cardíaca), redução da frequência cardíaca basal e limitação da arritmia de reperfusão. ^154^ Esses efeitos podem explicar os resultados benéficos dos ácidos graxos ω3 sobre a prevenção de morte súbita observada em alguns estudos. 

 Vários estudos observacionais sugeriram que os ácidos graxos ω3 podem exercer proteção particular contra morte súbita, sobretudo nos pacientes com IAM. Esse efeito benéfico foi também verificado na subanálise do ensaio randomizado GISSI-Prevenzione, ^155^ mas não no mais recente, OMEGA. ^129^ Essa hipótese também foi verificada em pacientes portadores de cardiodesfibrilador implantável. Os resultados foram inconsistentes, sugerindo desde discreto efeito benéfico do ω3 na redução de arritmias ventriculares graves nesse subgrupo de pacientes ^156^ até um efeito pró-arrítmico em alguns pacientes. ^157^

 Devido aos resultados conflitantes, foram avaliados dados de metanálise, com total de 32.919 participantes incluídos em 9 estudos. Desses, 16.465 pacientes receberam ω3 e 16.454 receberam placebo. Houve redução não significativa do risco de morte súbita cardíaca ou arritmias ventriculares com os ácidos graxos ω3 (OR = 0,82 [IC 95%: 0,60 a 1,21], p = 0,21). ^158^

 Outra revisão avalia os resultados de estudos com ω3 nas arritmias cardíacas ventriculares e na morte súbita cardíaca, questionando se tais lípides produzem efeitos anti-arrítmicos, pró-arrítmicos ou se teriam efeitos neutros, o que, por sua vez, requer estudos randomizados controlados com desenho específico para essas populações. ^159^

#### 5.5.3. Efeitos na Insuficiência Cardíaca

 O grande ensaio randomizado GISSI-HF mostrou redução discreta de mortalidade quando ω3 (1 g/dia) foi suplementado a pacientes em classe funcional II-IV, ^160^ em consistência com outros estudos epidemiológicos e observacionais que sugeriram relação inversa entre consumo de peixe ou ω3 e eventos relacionados com insuficiência cardíaca (IC). ^161 , 162^

 Recomendações de diretrizes nacionais e internacionais consideram indicação IIb (nível de evidência B), a suplementação de ω3 na IC com base nos dados do GISSI-HF, ^160^ mas não de outros estudos em que ácidos graxos ω3 tenham sido suplementados. 

 Nesse estudo, que incluiu 6.975 pacientes com IC classe II-IV pela NYHA, e com índice de massa corporal (IMC) < 40%, ou ainda hospitalizados no último ano por IC, 1 g de ω3 foi adicionado à terapia padrão. Esta terapia incluiu inibidores da ECA/bloqueadores do receptor de angiotensina em 94%, betabloqueadores em 65%, e espironolactona em 39% dos pacientes. Esses indivíduos foram observados durante seguimento de 3,9 anos (mediana). Os ácidos graxos ω3 foram capazes de reduzir em 8% o desfecho coprimário de morte ou hospitalização cardiovascular, 10% no risco relativo de morte cardiovascular e 7% nas hospitalizações cardiovasculares. ^160^

## 5.6. Ácidos Graxos Poli-insaturados (Ômega-3 Vegetal)

 Embora ainda esteja em discussão a real influência dos ácidos graxos ω3 de origem vegetal sobre a DCV, a maior parte dos estudos observacionais prospectivos sugere que o consumo de ALA pode proteger contra eventos cardiovasculares. ^163^ Na análise prospectiva de mais de 45 mil homens do HPFS, o consumo de ω3, tanto de origem marinha quanto vegetal, associou-se à redução do risco cardiovascular, com pouca influência da ingestão de ω6. ^164^ Nos dados do NHS, que avaliou desfecho cardiovascular e acompanhou mais de 76 mil mulheres, o consumo de ALA associou-se inversamente ao risco de morte súbita cardíaca, mas não a outros tipos de desfechos coronarianos fatais ou IAM não fatal. ^165^

 Metanálises e revisões sistemáticas têm mostrado resultados contraditórios. ^93 , 166 , 167^ No estudo randomizado e controlado Alpha Omega, margarina suplementada com ALA por 40 meses não reduziu a taxa de eventos cardiovasculares em pacientes que já haviam sofrido IAM. ^128^

 Quanto à eficácia do ALA, observou-se discreta redução no risco de eventos cardiovasculares (RR = 0,95; IC = 0,83 a 1,07), mortalidade cardiovascular (RR = 0,95; IC = 0,72 a 1,26) e arritmias (RR = 0,79; IC = 0,57 a 1,10). ^133^

 O papel da relação ω6/ω3 na dieta sobre a patogênese de doenças cardiovasculares, inflamatórias e autoimunes também tem sido objeto de controvérsia nos últimos anos. A espécie humana experimentou mudanças drásticas na sua alimentação em relação à ingestão de ácidos graxos nos últimos milênios. Com a revolução agrícola e industrial, houve aumento do consumo de cereais, óleos e grãos ricos em ω6, e diminuição paralela da ingestão de ω3. A relação ω6/ω3, originalmente entre 1:1 a 3:1, atualmente varia de 15:1 a 40:1 na dieta ocidental. ^168 , 169^

 A maior parte dos estudos conclui que, para a promoção de saúde geral, a relação ω6/ω3 deveria ser mais baixa que a atualmente encontrada na população geral ocidental. ^170^ Alguns especialistas advogam diminuir essa relação por meio tanto do aumento do consumo de ω3 como também pela redução de ω6. De forma condizente, em um estudo clínico prospectivo de prevenção secundária em indíviduos pós-IAM, uma dieta experimental mediterrânea caracterizada, entre outros fatores, por ser mais rica em ácido alfalinolênico (C18:3 – ω3) e oleico (C18:1 – ω9) e mais pobre em linoleico (C18:2 – ω6), associou-se à redução de até 70% na mortalidade total. ^117^ Tal dieta incluía substituição de óleo de milho por azeite de oliva, com consequente diminuição da relação ω6/ω3 para até 4:1. ^171^

Enquanto houver evidências de que o aumento da ingestão de ω3, particularmente de DHA e EPA, há proteção às doenças cardiovasculares.

 Além disso, a validade de se utilizar apenas a relação ω6:ω3 na prática clínica e sua relação com o risco cardiovascular tem sido questionada por diversos especialistas. ^172 , 173^ Ambos os ácidos, ω6 e ω3, têm sido associados a efeitos benéficos para a saúde cardiovascular. Entretanto, a importância da relação ω6:ω3 fundamenta-se na competição enzimática existente entre o ω6 e o ω3 pela ação da delta-6 dessaturase, que converte ambos em diferentes subespécies. Por um lado, o consumo elevado de ω6 pode diminuir o metabolismo do ω3 (alfa-linolênico – C18:3) a EPA (C20:5) e DHA (C22:6), ^174^ limitando os benefícios do ácido ω3. Por outro, a afinidade maior da enzima delta-6-dessaturase pelos ácidos graxos ω3 pode fazer com que os metabólitos essenciais derivados da bioconversão do ω6 não sejam produzidos de forma satisfatória, o que justificaria recomendação para pequeno aumento de seu consumo quando comparado ao ω3. ^172^

Diante dessas questões, e até que surjam novas informações científicas que permitam modificações de conduta, as recomendações dietéticas atualmente devem ser feitas com base no consumo total de cada tipo de ácido graxo (ω6 e ω3), e não somente com base na relação ω6:ω3.

## 5.7. Trans

 Vários estudos observacionais têm vinculado o consumo de *trans* , ou alimentos que os contêm, com resultados cardiovasculares adversos. ^76 , 175 - 180^ Análise dos dados do NHS mostrou que a cada 2% do aumento no consumo de *trans,* houve aumento em 1,93 vez o risco relativo para doença coronariana. ^175^ Da mesma forma, a substituição de 2% de *trans* por INSAT reduziu o risco cardiovascular em 53%, conforme demonstrado na população do Seven Countries Study. ^181^

 O estudo Cardiovascular Health Study (CHS) ^182^ avaliou a concentração plasmática de *trans* (ácido elaídico) em 2.742 indivíduos e revelou que esses ácidos graxos se associaram a aumento de mortalidade total, principalmente pelo aumento de risco cardiovascular. O estudo que avaliou o banco de dados do NHS e HPFS também mostrou que o consumo de *trans* aumentou a 13% a mortalidade total, quando se comparou o maior ao menor quintil de consumo. ^180^

 Essa ação deletéria do *trans* sobre o risco cardiovascular pode ser atribuída a sua ação sobre o aumento do LDLc e diminuição dos transportadores ABCA1 e ABCG1, responsáveis pelo efluxo de colesterol dos macrófagos para as ApoA-I e HDL, respectivamente. ^183^

## 6. Disfunção Endotelial

 A disfunção endotelial é um dos eventos iniciais na gênese da DCV e decorre principalmente da redução da produção e/ou disponibilidade de óxido nítrico (NO) e um desequilíbrio entre fatores vasodilatadores e vasoconstritores derivados do endotélio. ^184 , 185^ Fatores de risco cardiovascular como LDL oxidada, dislipidemia, hipertensão, hiperglicemia, hiperinsulinemia e tabagismo podem induzir ativação endotelial, a qual induz aumento da produção de citocinas, quimiocinas e espécies reativas de oxigênio (ROS), reduzindo a capacidade de vasodilatação dependente de NO. Além disso, há aumento da permeabilidade endotelial, facilitando a passagem de LDLs para a camada subendotelial, em que estas podem sofrer modificação, por oxidação ou glicação, e deflagrar resposta inflamatória, levando as células endoteliais a expressarem moléculas de adesão e produzirem mediadores que promoverão quimiotaxia de células inflamatórias, ativação de plaquetas e proliferação e migração de célula muscular lisa (CML), contribuindo para a gênese da aterosclerose. ^186 , 187^ Por outro lado, o NO é capaz de reduzir a expressão de mediadores inflamatórios e moléculas de adesão por células endoteliais, e de diminuir a reatividade vascular, prevenindo a vasoconstrição no local da lesão. ^188 , 189^

 Já se demonstrou que dieta rica em gordura reduz a ativação da via de AMPK-Akt-PI3K-eNOS em células endoteliais levando à disfunção endotelial. ^185 , 190 , 191^ Em animais experimentais, o consumo de dieta hiperlipídica, por 6 semanas, aumentou a concentração plasmática de citocinas pró-inflamatórias e reduziu as concentrações de adiponectina, além disso, reduziu a produção de NO e promoveu disfunção endotelial. ^192^

 Os ácidos graxos SAT, sobretudo o palmítico, ativam resposta inflamatória e estresse oxidativo, que prejudicam a integridade do endotélio e causam disfunção endotelial. Os ácidos graxos SAT são capazes de ativar o fator de transcrição NF-κB que controla vias de sinalização inflamatória e do estresse oxidativo ^193^ e, com isso, induzir disfunção endotelial, por meio do aumento de ROS e da secreção de citocinas pró-inflamatórias, como IL-6 e TNF-α. ^194 , 195^

 Estudo em células endoteliais demonstrou que o ácido palmítico é capaz de inibir a ativação dependente de insulina da óxido nítrico sintase endotelial (eNOS), reduzindo assim a produção de NO, efeito que foi mediado pela ativação de PTEN (homólogo da fosfatase e tensina deletado no cromossomo 10); tal fosfatase, quando ativada, reduz a fosforilação de proteína quinase B (AKT). ^196^ Em outro estudo, o tratamento de células endoteliais com ácido palmítico diminuiu a produção de NO, por reduzir a fosforilação de IRS-1, AKT e eNOS mediada pela insulina. Esse efeito foi dependente do aumento da ativação de IKK-β mediada pelo palmítico. ^197^

 O ácido graxo saturado é capaz de promover inflamação e estresse do retículo em diferentes tipos celulares. ^69 , 193 , 194 , 198 , 199^ Em fibroblastos cardíacos, o ácido palmítico ativou vias inflamatórias e induziu disfunção mitocondrial e estresse do reticulo endoplasmático, levando ao aumento da produção de ROS e ativação da via do inflamassoma, efeito que foi mitigado pela presença de EPA. ^198^ Em célula muscular lisa, o ácido palmítico é capaz de induzir apoptose por meio da ativação de TLR4, aumento da produção de ROS e aumento da expressão de caspase 3 e caspase 9. ^199^ Em macrófagos, os ácidos graxos SAT são capazes de aumentar conteúdo de receptores LOX1 e, com isso, aumentar a captação de LDL modificadas, levando ao aumento da produção de ROS e estresse do retículo, efeitos que foram corrigidos pela adição de INSAT ao meio. ^193^ Em células endoteliais, o tratamento com ácido palmítico induziu disfunção endotelial, reduziu fosforilação em eNOS e AMPK e, com isso, a produção de NO. Ainda, palmítico induziu aumento de ROS, óxido nítrico sintase induzida (iNOS) e apoptose, ações que foram atenuadas com a incubação concomitante com EPA. ^194^ . 

 O consumo habitual de dieta rica em ácidos graxos SAT foi associado com alteração da função endotelial em indivíduos jovens com sobrepeso. ^200^ Por outro lado, estudos de intervenção, em que se avaliou o efeito da ingestão aguda de gordura saturada sobre a função endotelial, são ainda controversos. O estudo DIVAS, realizado em indivíduos com risco cardiovascular moderado, não observou efeito da substituição isocalórica, por 16 semanas, de ácidos graxos SAT por MONO ou ácido linoleico na função endotelial, marcadores inflamatórios e de resistência insulínica. Entretanto, observou-se redução nas concentrações plasmáticas de CT e LDLc e E-selectina. ^201^ No estudo DIVA-2, que avaliou, em mulheres pós-menopausa, o efeito agudo de refeições hiperlipídicas na função endotelial e em marcadores de risco CV, não foi observada diferença no impacto dos diferentes ácidos graxos sobre marcadores de função endotelial. ^202^

Os ácidos graxos SAT, sobretudo o palmítico, ativam resposta inflamatória e estresse oxidativo, que prejudicam a integridade do endotélio e causam disfunção endotelial.

 A suplementação de óleo de peixe melhora significantemente a função endotelial em vasos de resistência, quando medida no antebraço. ^123^ Em estudo comparado a placebo, a complacência vascular sistêmica melhorou após 3 g de DHA ou de EPA. ^203^ Os mecanismos propostos relacionam-se com a incorporação dos ω3 em fosfolípides de membrana, modificando a complacência vascular. ^68^ Atenuação do aumento da rigidez vascular relacionada à idade em pacientes com dislipidemia e da distensibilidade das artérias carótidas também é um mecanismo proposto. ^204^ A disfunção do endotélio está intimamente associada à inflamação da parede vascular. Os efeitos da suplementação com ácido graxo ω3 marinho sobre a função endotelial *in vivo* , em humanos, são controversos. Uma análise de 33 ensaios de intervenção sugere que os ácidos graxos ω3 de origem marinha podem melhorar a função endotelial em sujeitos dislipidêmicos com sobrepeso e em diabéticos, embora os resultados sejam conflitantes em pacientes com DCV e inconsistentes em indivíduos saudáveis. ^63^

 Estudo em células endoteliais mostrou que ácido elaídico pode causar morte celular através da ativação da via das caspases, ^205^ bem como ativação do NF-κB por meio do aumento da produção de ROS, o que culminou na elevação da expressão de VCAM-1 e ICAM-1 e maior adesão de leucócitos. ^206^ Corroborando esses resultados, estudo em humanos relatou aumento das concentrações plasmáticas de E-selectina e proteína C-reativa (PCR) com o consumo de *trans.* ^207^ Observou-se também que o aumento do consumo de *trans* elevou as concentrações plasmáticas de E-selectina, VCAM e ICAM, em 730 mulheres que faziam parte do NHS. ^208^ Estudo em células endoteliais que investigou o efeito de ácidos graxos *trans* na ativação do NF-kB mostrou que o ácido elaídico induziu fosforilação de IkB medido pelo aumento das concentrações de IL-6. ^209^ Além disso, promoveu diminuição tanto da produção de NO como da sinalização da insulina e, ainda, promove sinalização pró-inflamatória e morte celular. ^210^

### 6.1. Pressão Arterial

 Em estudos de intervenção dietética em pacientes com sobrepeso, indivíduos que consumiram uma porção de peixe por dia apresentaram diminuição da pressão arterial sistólica e diastólica, sendo esta redução ainda maior quando associada ao programa de perda de peso, mesmo ajustada para outras covariáveis. ^211^

 Uma metanálise realizada nos anos 1990 concluiu que o efeito da suplementação com ácidos graxos ω3 sobre a pressão arterial é dose-dependente, sendo eficaz a partir de 3,0 g/dia, com redução da pressão arterial sistólica (0,66 mmHg) e diastólica (0,35 mmHg)/g de ω3 consumido. ^212^

 Em outra metanálise com 36 ensaios clínicos randomizados, a suplementação com óleo de peixe (dose mediana 3,7 g/dia) mostrou reduzir a pressão arterial sistólica em 2,1 mmHg e a diastólica em 1,6 mmHg. ^213^ Esses resultados modestos podem ser explicados pelo baixo grau de pureza e baixas concentrações nas formulações utilizadas. Já outros estudos com doses baixas (1,6 g de DHA e 0,6 g de EPA) não demonstraram benefícios na pressão arterial, possivelmente relacionados às baixas doses utilizadas. Em pacientes de alto risco, como aqueles em hemodiálise, 4 meses de suplementação com 2 g de ω3 se associaram a menor pressão arterial sistólica (-9 mmHg) e diastólica (-11 mmHg), comparado ao óleo de oliva. ^214^

 Metanálise com pacientes com DM demonstrou que suplementação com ω3 reduziu em 1,8 mmHg a pressão diastólica. ^215^ Theobald et al. ^216^ também evidenciaram redução da pressão arterial com o consumo de baixas doses de ω3. ^216^ Entretanto, quando se avalia os índices de função endotelial ou rigidez arterial, os dados são conflitantes entre os estudos. ^216 , 217^

 Revisão sistemática e metanálise conduzida por Schwingshackl et al. ^218^ investigou o impacto dos MONO no metabolismo lipídico, pressão arterial e eventos cardiovasculares. Os resultados demonstraram que dietas com conteúdo de MONO acima de 12% em relação ao valor calórico total da dieta apresentaram efeito benéfico apenas sobre a pressão arterial diastólica e sistólica. 

 Além dos benefícios observados no perfil lipídico, ^219^ o padrão Mediterrâneo também melhora a pressão arterial ^220^ e confere proteção adicional sobre o estresse oxidativo, ^221^ marcadores de inflamação ^222^ e disfunção endotelial. ^112^ Nesse sentido, nota-se que benefícios adicionais à saúde foram conferidos por outras substâncias, de forma independente ao ácido graxo MONO. Para tais substâncias, não há por enquanto recomendação de consumo específico determinado. 

Portanto, há poucas evidências sobre o papel protetor dos MONO na proteção à hipertensão arterial sistólica e função endotelial para que se possam propor recomendações específicas.

### 6.2. Acidente Vascular Cerebral

 A elevação da pressão arterial é o principal fator de risco de AVC. Em relação ao consumo saturados, alguns estudos observaram pouco ou nenhum efeito sobre o risco de AVC. ^12 , 96 , 223 , 224^ No estudo Women Health Initiative, que acompanhou mulheres por cerca de 8 anos, a redução no consumo de saturados não reduziu o risco de AVC. ^59^ Por outro lado, outros estudos de coorte, como Japan Collaborative Cohort Study for Evaluation of Cancer Risk (JACC Study), que acompanhou por 14 anos 58.453 japoneses, ^225^ e também o estudo PURE ^102^ observaram associação inversa entre o consumo de SAT e risco de AVC. 

 Estudo de metanálise encontrou associação inversa entre consumo de SAT e risco de AVC apenas para homens asiáticos e com índice de massa corporal (IMC) < 24, mostrando que fatores como etnia, gênero e peso corporal influenciam na associação entre ácidos graxos SAT e risco de AVC. ^226^

 Assim, até o momento, não há evidências robustas para recomendar a redução de ácidos graxos SAT na prevenção do risco de AVC. ^96^

 Estudos randomizados utilizando ácidos graxos ω3 como EPA, DHA e o docosapentaenoico (C20:5 [DPA]) reduziram fatores de risco e mecanismos para eventos cardiovasculares incluindo hipertensão arterial, hiperlipidemia, disfunção endotelial, ^213 , 227 , 228^ sugerindo seu papel protetor na DCV. Entretanto, o impacto desses ácidos graxos no AVC isquêmico ainda é controverso. Estudos observacionais demonstraram associações inversas entre o relato de consumo de ácidos graxos ω3 na dieta e AVC isquêmico, ^229^ o que não se confirmou em metanálise de ensaios clínicos randomizados com a suplementação de ácidos graxos ω3. ^227^ No entanto, os dados de metanálise foram provenientes de estudos de suplementação de curta duração, com pacientes de alto risco e, em geral, com AVC prévio, em que o AVC não era um desfecho predeterminado. Com isso, não se pode generalizar tais resultados a populações em prevenção primária. ^230^ Além disso, o AVC isquêmico pode estar relacionado com doença aterotrombótica ou cardioembólica, cujos mecanismos fisiopatológicos são distintos. ^231^ O DHA pode reduzir o risco de AVC aterotrombótico, por reduzir disfunção endotelial e aterosclerose, enquanto EPA e DPA podem ter maior impacto no AVC cardioembólico, por seus efeitos na coagulação e na fibrilação atrial. ^232^ Além disso, quase todos os estudos com ω3 e risco de AVC avaliaram o consumo autorreferido desses ácidos graxos na dieta, não sendo possível diferenciar o tipo de ácido graxo consumido. 

 Em uma revisão sistemática de três grandes coortes norte-americanas, CHS, NHS e HPFS, a concentração circulante de ácidos graxos foi mensurada no período basal para avaliar sua relação com a incidência de AVC isquêmico. Os AVC isquêmicos foram adjudicados prospectivamente e classificados em aterotrombóticos ou cardioembólicos, e o risco foi calculado de acordo com a concentração de ácidos graxos circulantes. Maior concentração de DHA circulante foi inversamente associada à incidência de AVC, e os de DPA com AVC cardioembólico. Não houve associação entre EPA e AVC. Tais achados sugerem benefícios diferenciados de acordo com o ácido graxo ω3 envolvido. ^233^

## 7. Inflamação

 Os ácidos graxos SAT são componentes essenciais do lípide A, presente na parede celular de bactérias gram-negativas – é a porção endotóxica do lipopolissacarídeo (LPS). ^234^ Já é claro na literatura científica que os ácidos graxos SAT disparam a sinalização inflamatória, pois modulam tanto a via do NF-kB, por meio da estrutura dos receptores TLR4, ^235^ como a via do TLR2. ^236^ Outra estratégia de intensificação do processo inflamatório induzido pelo consumo de SAT é a ativação intracelular da proteína inflamassomal NLRP3. Quando o inflamassomo encontra-se ativo, torna madura as proteínas IL1β e IL18, induzidas pelo NF-kB. Além de ter sido demonstrado que a gordura saturada oriunda da dieta foi capaz de ativar esse mecanismo através do receptor TLR4, ^237^ as prostaglandinas E2 (PGE2) derivadas do ácido araquidônico ^238^ também o fazem, trazendo implicações importantes à doença coronariana ^239^ e a comorbidades associadas ao DM2, como a retinopatia diabética. ^238^

 Em macrófagos, o ácido láurico ^240^ apresentou maior capacidade inflamatória, avaliada pela ativação da via do TLR4, quando comparado ao mirístico, palmítico e esteárico, enquanto, MONO e POLI não ativaram essa via. Foi demonstrado que o pré-tratamento das células, com diferentes ácidos graxos INSAT, reduziu significativamente o efeito pró-inflamatório induzido pelo ácido láurico. ^241 , 242^ Além disso, a inibição da expressão de TLR2 melhorou a ação da insulina tanto em células musculares tratadas com ácido palmítico como em músculo esquelético e tecido adiposo de animais alimentados com dieta rica em saturados. ^243^

 Estudo conduzido em 965 indivíduos jovens saudáveis mostrou associação positiva entre a concentração plasmática de mirístico e palmítico com a concentração de PCR, enquanto esteárico e linoleico relacionaram-se inversamente. ^244^

 Como precursores de eicosanoides e outros mediadores anti-inflamatórios, os ácidos graxos ω3 podem proporcionar efeitos anti-inflamatórios, com benefícios para inúmeras condições patológicas, incluindo as cardiovasculares. Vários estudos experimentais têm mostrado gama ampla de efeitos anti-inflamatórios do ω3, embora as investigações *in vivo* em humanos tenham mostrado resultados conflitantes. ^154 , 245^

 Os ácidos graxos POLI da série ω3, como EPA e DHA, são precursores de eicosanoides anti-inflamatórios com benefícios cardiovasculares. Se, por um lado, estudos experimentais demonstram efeitos anti-inflamatórios do ω3, alguns estudos em humanos apresentam resultados conflitantes com relação ao desfecho cardiovascular. ^133 , 154 , 245^

 Em estudos transversais e de coorte, o consumo alimentar de ω3 marinho associou-se a menores concentrações plasmáticas de marcadores inflamatórios, incluindo moléculas de adesão e PCR. ^246 , 247^ Concentrações de ω3 marinho no plasma e em membranas de eritrócito ou granulócito associaram-se inversamente às concentrações de PCR em indivíduos saudáveis ou com DAC estável. ^248 - 250^ Estudo de intervenção mostrou que alimentação contendo ω3 marinho ou suplementação com óleo de peixe ou DHA mostraram resultados compatíveis com a atenuação da resposta inflamatória em portadores de DM2 e hipertrigliceridêmicos. ^251 - 253^ Em outros ensaios, a dieta suplementada com ω3 não provocou alterações significativas em parâmetros inflamatórios em portadores de risco cardiometabólico (1,24 g/dia) ^254^ e em pacientes com IAM prévio (5,2 g/dia), ^255 , 256^ o mesmo ocorrendo com a suplementação com ácidos graxos POLI sobre concentrações plasmáticas de PCR em indivíduos saudáveis (2,0 ou 6,6 g/dia). ^256^ Diferenças no perfil da população, forma de administração, dose de suplementação, uso concomitante de estatinas e parâmetros analisados podem ter contribuído para essas discrepâncias de resultados. Portanto, a real relevância clínica dos efeitos anti-inflamatórios dos ácidos graxos ω3 de origem marinha é ainda incerta. 

 Estudos conduzidos com ALA atestam relação inversa entre seu consumo e parâmetros inflamatórios, incluindo PCR sérica. ^246 , 257 , 258^ A suplementação com ALA reduziu a concentração de marcadores inflamatórios em indivíduos dislipidêmicos, o que ocorre especialmente quando a dieta de base é rica em gordura SAT e pobre em MONO. ^259^

 A ingestão de *trans* foi positivamente associada à inflamação sistêmica, caracterizada pelo aumento de IL-1β, IL-6, TNF-α e proteína quimiotática de monócitos (MCP) em portadores de DCV. ^260^ Estudo caso-controle conduzido em 111 indivíduos com DAC mostrou que a incorporação de ácidos graxos *trans* em eritrócitos associou-se a maior concentração plasmática de PCR e IL-6. ^77^

## 8. Resistência à Insulina e Diabetes

 A sinalização inflamatória induzida pelo consumo de ácidos graxos SAT é capaz de ativar proteínas com atividade de serina-quinase, como a JNK e o IKK. Essas proteínas interferem negativamente na transdução do sinal da insulina, por reduzirem a fosforilação em tirosina do substrato 1 do receptor de insulina (IRS1). ^261 , 262^

 O consumo por 3 meses de dieta hiperlipídica, rica em SAT, reduziu a sensibilidade à ação da insulina em indivíduos não diabéticos. ^263^ Já no estudo de coorte LIPGENE, que avaliou 417 indivíduos com síndrome metabólica, a redução no consumo de SAT não teve impacto sobre a concentração plasmática de glicose e insulina de jejum, HOMA-IR, sensibilidade à insulina e marcadores inflamatórios. ^264^ Vale ressaltar que, neste estudo, a substituição das calorias provenientes de saturados foi compensada por INSAT ou carboidratos complexos. No estudo Reading, Imperial, Surrey, Cambridge, and Kings (RISCK), conduzido em 548 indivíduos com excesso de peso e risco cardiometabólico elevado, comparou-se a substituição isocalórica de dieta rica em saturados, com alto índice glicêmico, por dieta rica em MONO com alto ou baixo índice glicêmico, por 6 meses, e não se observou alteração na sensibilidade à insulina. ^265^ Por outro lado, foi demonstrado que dietas enriquecidas com saturado, especialmente palmítico, induziram resistência à insulina de forma aguda, em indivíduos com ou sem intolerância à glicose. ^266^

 Estudos prospectivos verificaram associação positiva entre ingestão de ácidos graxos SAT e intolerância à glicose. ^267 , 268^ O HPFS, que incluiu 42.504 homens, observou associação entre ingestão total de gordura e ácidos graxos SAT com aumento do risco de DM2, mas de forma dependente dos valores de IMC. ^269^

 Já no Iowa Women’s Health Study, ^270^ realizado com 35.988 mulheres sem diagnóstico prévio de DM2, o consumo de saturados não se associou ao risco de DM2; no entanto, o risco de diabetes foi inversamente relacionado com a substituição de ácidos graxos SAT por POLI. Além disso, o consumo de gordura de origem animal foi associado ao aumento de 20% no risco de incidência de DM2. ^270^ Em outro estudo prospectivo, NHS, com 84.204 mulheres, foi avaliada a relação entre a ingestão de gorduras e o risco de DM2, e concluiu-se que a ingestão total de gordura e de saturados não se associou ao aumento do risco de DM2. ^271^

 O Women’s Health Initiative Trial, estudo de intervenção dietética em mulheres pós-menopausa, acompanhadas por cerca de 8 anos, constatou que a redução do consumo de gordura total (9,1% do VCT) e de saturados (3,2% do VCT) não alterou o risco de desenvolvimento de DM2. Vale ressaltar que redução do consumo de gorduras foi compensada pelo aumento de 10% na ingestão de carboidratos. ^272^

 Sabe-se que o desenvolvimento de DM2 resulta da interação de fatores genéticos e estilo de vida, como a dieta. O estudo EPIC (EPIC-InterAct) ^273^ avaliou a influência da suscetibilidade genética sobre o efeito do consumo de diferentes macronutrientes no risco de desenvolvimento de DM2 e observou que os SAT não foram associados ao risco de DM2. Ainda, observou-se que a predisposição genética a DM2 não influenciou a relação entre os nutrientes e risco de DM2. ^273^ Em outra coorte deste estudo, avaliou-se a associação entre o risco de DM2 e a concentração de diferentes ácidos graxos presentes em fosfolípides plasmáticos. ^14^ Observou-se que mirístico, esteárico e palmítico se correlacionaram positivamente com risco de DM2. Cabe ressaltar que a maior concentração plasmática desses ácidos graxos se associou positivamente ao consumo de álcool, margarina e refrigerantes, e negativamente ao consumo de frutas e vegetais, azeite de oliva e óleos vegetais. Já o ácido pentadecanoico (15:0) e o heptadecanoico (17:0), associados positivamente ao consumo de leite e produtos lácteos, castanhas, bolos, frutas e vegetais, apresentaram-se inversamente associados com risco de DM2 ^14^ . Portanto, os efeitos deletérios observados não podem ser atribuídos exclusivamente à ação isolada desses ácidos graxos SAT, mas sim a um contexto de dieta inadequada. 

 Metanálise de estudos observacionais não encontrou associação entre o consumo de saturados e o risco de DM2. ^223^ Em outra metanálise realizada com estudos de coorte que avaliaram a associação entre padrões alimentares e risco de DM2, a redução do risco de DM2 foi associada a padrões alimentares saudáveis, e não a um dos macronutrientes especificamente. ^274^ Já em metanálise realizada com estudos controlados de intervenção alimentar que avaliaram o efeito da substituição isocalórica de macronutrientes na concentração plasmática de glicose e insulina e parâmetros relacionados com a resistência à insulina, mostrou-se que a substituição de SAT por POLI reduziu glicemia, hemoglobina glicada (HbA1c), peptídio C e HOMA. ^109^

Até o momento, as evidências com relação ao impacto dos saturados no risco de DM2 não são conclusivas. Os resultados dos estudos sinalizam que a influência dos demais nutrientes e constituintes da dieta não pode ser descartada e apontam na direção das orientações dos guias alimentares internacionais e brasileiro. Assim, preconiza-se que a adoção de padrões alimentares saudáveis, que priorizem o consumo de frutas, verduras e legumes, além de lácteos, carnes magras e carboidratos complexos e com baixo consumo de carboidrato simples, carnes processadas e alimentos ultraprocessados é mais eficiente na redução do risco de doenças cardiometabólicas.

 Estudos de coorte prospectivos envolvendo grande número de participantes sugeriram que o maior consumo de ácido graxo ω3 associa-se à maior incidência de DM2. ^270 , 275^ Por outro lado, metanálise que avaliou a relação entre os ácidos graxos POLI ω3 de origem marinha e o risco de DM2 ^276^ mostrou que tanto o consumo de peixes e crustáceos (13 estudos, RR por 100 g de peixe/dia = 1,12, IC 95%: 0,94-1,34) como a suplementação com EPA+DHA (16 coortes, RR por consumo de 250 mg/dia= 1,04, IC 95% = 0,97-1,10) não se associaram ao risco de diabetes, As concentrações plasmáticas de EPA+DHA (5 coortes, RR para 3% do total de ácidos graxos = 0,94, IC95%: 0,75-1,17) também não se associaram ao risco para DM2. ^276^ Em virtude da heterogeneidade observada entre os estudos e efeitos inconsistentes relacionados à sua duração, não há evidências de ações benéficas ou deletérias do consumo de peixes/frutos do mar ou da suplementação de EPA+DHA no risco de desenvolvimento de DM. 

 Por outro lado, existem evidências de que concentrações plasmáticas mais elevadas de EPA/DHA possam se associar a menor chance de novos casos de diabetes. ^277^ Apesar da exposição de benefícios descritos sobre o consumo de ω3 a portadores do DM2, uma metanálise envolvendo 23 estudos clínicos randomizados não evidenciou alterações significativas de HbA1c, glicemia de jejum ou insulina de jejum quando o ω3 foi suplementado na dose média de 3,5 g/dia. ^86^ . Da mesma forma, outra metanálise com 26 ensaios clínicos constatou que a suplementação com óleo de peixe, variando entre 2 a 22 g/dia, não alterou a concentração plasmática de HbA1c em pacientes diabéticos; ^278^ entretanto, é necessário considerar as altas doses utilizadas nos estudos. Além disso, o estudo ORIGIN mostrou que a suplementação com ω3 não reduziu a taxa de eventos cardiovasculares em indivíduos com intolerância à glicose, glicemia de jejum alterada e portadores de DM2. ^279^

 Os efeitos do ALA sobre o perfil glicêmico também não têm sido consistentes. ^217^ Contudo, há sugestões de que o seu consumo possa beneficiar o metabolismo da glicose. Dados prospectivos do CHS mostraram que concentrações plasmáticas mais elevadas de ALA se associam à menor chance de novos casos de DM2. ^277^ De forma concordante, em uma grande análise prospectiva com mais de 43 mil chineses, o consumo de ALA associou-se inversamente ao risco de aparecimento de DM2. ^280^ Em revisão sistemática e metanálise de ensaios randomizados e controlados, a suplementação com ALA reduziu a glicemia em 3,6 mg/dL. ^67^ Com relação à linhaça, um estudo randomizado revelou melhora da sensibilidade à insulina. ^281^

 Revisão sistemática da literatura identificou 16 estudos prospectivos, incluindo coortes, em que o consumo e as concentrações plasmáticas de ω3 foram avaliados com relação à incidência de DM2. De um total de 540.184 indivíduos, houve 25.670 novos casos de DM2. ^276^ Tanto o consumo de ALA (n = 7 estudos) quanto sua concentração plasmática (n = 6 estudos) não se associaram a risco para DM2. Houve moderada heterogeneidade (< 55 %) para a concentração circulante de ALA e diabetes, podendo sugerir risco discretamente menor de DM2. ^276^

 Estudo de revisão sobre ácidos graxos ω3, risco cardiometabólico e DM2 concluiu que, para o ALA, não há dados demonstrando proteção quanto à conversão do risco cardiometabólico em DM2, ou de redução de mortalidade em pessoas com DM2 ou risco cardiometabólico. O ALA parece reduzir a agregação plaquetária em diabéticos. ^282^

 Estudos observacionais, utilizando marcadores biológicos da ingestão de gorduras ou de questionários alimentares, sugerem associação inversa entre a ingestão de ω6 e risco de DM2, embora os dados nem sempre sejam consistentes. ^271 , 283^

 No NHS, envolvendo 84.204 mulheres com idade entre 34 e 59 anos, sem DM, DCV ou câncer, seguidas prospectivamente por 6 anos, a ingestão de ω6, avaliada por questionários devidamente validados, associou-se ao menor risco de DM2. ^271^ Em homens, outro grande estudo prospectivo, o HPFS, também mostrou que a ingestão de ω6 como ácido linoleico estava associada ao menor risco de DM2, naqueles com idade < 65 anos e IMC < 25 kg/m ^2^ . ^269^ Também, no estudo Singapore Chinese Health Study, no qual foram avaliados prospectivamente mais de 43.000 chineses, o consumo de ω6 não se associou ao aparecimento de novos casos de DM, nem a relação ω3:6. ^280^

 Dados provenientes de pequenos estudos de intervenção também são controversos no que diz respeito ao efeito do ω6 sobre a sensibilidade à insulina. ^284^ São necessários mais estudos controlados, a longo prazo, para identificar a melhor composição de ácidos graxos da dieta, a fim de reduzir o risco de DM2. Poucos são os dados disponíveis, permanecendo incerto, ainda, os efeitos do tipo de ácidos graxos (POLI e SAT) da dieta sobre o controle glicêmico de indivíduos diabéticos. ^285^

 Com relação aos ácidos graxos *trans* , estudos experimentais demonstraram efeitos adversos na homeostase de glicose e no desenvolvimento de diabetes. ^286 - 288^ Além disso, os *trans* aumentam as concentrações plasmáticas de TG, insulina e glicemia pós-prandial ^289^ e reduzem a captação de glicose pelo músculo esquelético, alterações que são acompanhadas pelo aumento de gordura visceral e hepática. ^287^ Estudo que avaliou associação entre *trans* e síndrome metabólica usando dados do NHANES verificou que a concentração plasmática de *trans* se associou positivamente com risco de síndrome metabólica e seus componentes. ^290^ Mesmo em pequenas quantidades, exercem ações deletérias sobre a homeostase glicêmica, estímulo a glicogênese e aumento de gordura visceral; ^286 , 289^ demonstrou-se que o consumo de dieta rica em *trans* induziu maior ganho de peso, esteatose hepática e RI por meio da supressão da via de sinalização do IRS1, com consequente redução da fosforilação da AKT e da PKC. ^286^ Já o consumo de *trans* em indivíduos portadores de DM2 ou excesso de peso ^291^ se correlacionou consistentemente com redução da sensibilidade à insulina e com aumento de glicemia e insulinemia pós-prandial. 

 No CHS foi avaliada associação da incidência de DM2 tanto com a concentração de *trans* em fosfolípides plasmáticos como com o seu consumo. ^292^ As concentrações plasmáticas de *trans* se associaram positivamente à incidência de DM2 após correção para lipogênese *de novo* . Por outro lado, o consumo de *trans* , após ajuste para o consumo de outros alimentos, não se associou a incidência de DM2. ^292^

 Importante revisão sistemática evidenciou que o consumo de *trans* se associou com aumento de 28% de risco de DM2, quando analisados estudos com baixo risco de viés. ^223^ Além disso, mostrou associação com aumento de mortalidade total (34%), mortalidade por doença coronariana (28%) e aumento de risco CV (21%). 

## 9. Doença Hepática Gordurosa

### 9.1. Esteatose Hepática

 O fígado apresenta elevada capacidade metabólica relativa a todos os nutrientes, em especial às gorduras. Contudo, quando há acúmulo intracelular de triglicérides em mais de 5% dos hepatócitos, está caracterizada a doença hepática gordurosa não alcoólica ( *nonalcoholic fatty liver disease* [NAFLD]), ^293^ uma condição clínica de amplo espectro que compreende desde a esteatose hepática, evoluindo para esteato-hepatite não alcoólica ( *nonalcoholic steatohepatitis* [NASH]) marcada pela presença de gordura e infiltrado inflamatório. Essa condição predispõe o aparecimento de complicações hepáticas, como fibrose, cirrose e carcinoma hepatocelular, ^294 , 295^ e extra-hepáticas, como DCV e DM2. ^296^ O diagnóstico deve excluir causas secundárias de esteatose hepática, tais como consumo abusivo de álcool, hepatites virais ou autoimunes, ou, ainda, esteatose decorrente do uso de drogas esteatogênicas. ^296 , 297^

 A sua ocorrência está fortemente associada aos fatores que compõem o risco cardiometabólico, tais como obesidade, resistência à insulina e DM2, dislipidemias. ^296 , 297^ Cerca de 90% dos pacientes com NAFLD apresentam um ou mais fatores que compõem o risco cardiometabólico, e 30% apresentam todos os fatores. Já foi descrito que o risco de incidência de NAFLD aumenta proporcionalmente ao somatório dos fatores relacionados ao risco cardiometabólico. Por essa razão, a NAFLD é apontada como a manifestação hepática do risco cardiometabólico. ^298^ Em indivíduos com DM2, o risco de progressão para esteato-hepatite junto ao desenvolvimento de complicações da doença hepática gordurosa está de 2 a 4 vezes aumentado. ^294^

 O desenvolvimento de NAFLD está relacionado ao aumento do influxo de AGL no fígado, principalmente decorrente do aumento da lipólise do tecido adiposo, associado à resistência à insulina e ao excesso de calorias da dieta. ^299^ Em portadores de NAFLD, cerca de 60% do triglicérides hepático é proveniente da lipólise do tecido adiposo, 26% originados na lipogênese *de novo* e 14% provenientes da dieta. ^300^ Além disso, ocorre aumento da lipogênese hepática, juntamente à diminuição da β-oxidação mitocondrial ou da secreção de VLDL pelo fígado, contribuindo para o acúmulo hepático de lípides. ^301 , 302^ O acúmulo hepático de lípides pode acarretar lesão inflamatória, desenvolvimento de fibrose e perda da função. A presença de fibrose é o preditor mais importante de mortalidade relacionada com NAFLD, e sua presença eleva o risco de morte por DCV e causas hepáticas. ^296^

 Outros fatores podem estar relacionados com a progressão da doença, tais como: 1) aumento da geração de ROS, com promoção do estresse oxidativo decorrente de disfunção mitocondrial ou estresse do retículo endoplasmático; ^303^ 2) peroxidação lipídica; 3) ativação de vias inflamatórias com consequente aumento da secreção hepática de citocinas e mediadores inflamatórios, como TNF-α e IL6, que podem agravar o quadro. ^304^ Além disso, a falta de atividade física, associada à dieta inadequada, rica em gorduras e com excedente calórico, predispõe o desenvolvimento de NAFLD. ^305^

 Indivíduos com NAFLD apresentam aumento na expressão hepática de genes relacionados ao transporte de ácidos graxos (proteína ligadora de ácido graxo 4 e 5 [FABP 4 e 5]), hidrólise de triglicérides (LPL), recrutamento de monócitos (MCP1) e receptor ativado por proliferadores do peroxissomo gama-2 (PPARγ-2) ^306^ Já se demonstrou que PPARγ induz expressão de SREBP-1c, com reforço na expressão de genes controladores de proteínas relacionadas à síntese hepática de triglicérides. ^307^

 Estudos em modelos animais ^308 , 309^ ou ensaios clínicos com humanos ^306 , 310^ evidenciam de forma robusta a participação da dieta hiperlipídica na indução da esteatose hepática. A resistência à ação da insulina tem papel central no acúmulo hepático de lípides ^311^ e, neste contexto, a quantidade de gordura (especialmente o tipo de ácido graxo) influencia a lipogênese hepática e a ação da insulina. ^301 - 303^

### 9.2. Ácidos Graxos Saturados e Esteato-hepatite Não Alcoólica

 Em hepatócitos, o ácido esteárico e o ácido palmítico são capazes de induzir apoptose via ativação excessiva da quinase c-Jun N terminal (JNK). ^312^ Verificou-se ainda que o tratamento com palmitato é capaz de ativar a via de sinalização do IRE1α por meio dos receptores TLR4. A IRE1 é uma proteína transmembrânica do retículo endoplasmático que governa a resposta a proteínas malformadas no retículo, e induz apoptose. ^313^

 Estudo recente demonstrou que o ácido palmítico promove estresse oxidativo, estresse de retículo endoplasmático (ERE), disfunção mitocondrial e inflamação em células HepG2. Animais alimentados com dieta hiperlipídica, rica em SAT, desenvolveram esteatose hepática, NASH e fibrose, condições associadas ao ERE, e desenvolvimento de resistência à insulina. Por outro lado, a adição de ácido oleico à dieta protegeu contra lipotoxicidade hepática induzida por SAT. ^314^ O consumo de ácidos graxos SAT ou sacarose, por animais experimentais, induziu o acúmulo de SAT no fígado, ERE e apoptose, comparado a dieta rica em POLI. ^315^

 Estudo em humanos mostrou que o consumo total de gorduras, bem como o consumo de SAT, foi associado positivamente ao conteúdo hepático de lípides. ^316^ Estudo duplo-cego randomizado, conduzido por 7 semanas em indivíduos saudáveis, revelou que dietas enriquecidas com palmítico ou linoleico promoveram ganho de peso similar. Entretanto, o excesso de calorias proveniente de ácidos graxos SAT aumentou o depósito de lípides no fígado, tecido adiposo visceral e gordura total e reduziu o percentual de massa magra, quando comparado à dieta rica em POLI. Além disso, o aumento de gordura corporal e hepática se correlacionou positivamente com a elevação das concentrações plasmáticas de palmítico e inversamente com linoleico. ^317^

 Estudo recente mostrou que o consumo adicional de 1.000 Kcal sob a forma de ácidos graxos SAT por 3 semanas acarretou maior elevação do conteúdo intra-hepático de lípides (+55%) quando comparado ao mesmo incremento calórico, provenientes de INSAT ou açúcar, os quais elevaram o conteúdo hepático de lípides em +15% e +33%, respectivamente. Além disso, consumo de SAT induziu resistência à insulina e elevou em 49% as concentrações plasmáticas de ceramidas. ^318^

### 9.3. Ácidos Graxos Insaturados e Esteato-hepatite Não Alcoólica

 No fígado, a enzima SCD1 é a principal responsável pela inserção de duplas ligações em cadeias saturadas de ácidos graxos como o palmítico (C16:0), convertendo-o a palmitoleico (C16:1) e o esteárico (C18:0) a oleico (C18:1). Isso ocorre no intuito de controlar o conteúdo de saturados quando em excesso no organismo, seja pela alimentação ou pela conversão endógena excessiva de palmítico oriundo da lipogênese *de novo* . Na NAFLD, vias lipogênicas estão ativadas e as de dessaturação (SCD1) e oxidação, reduzidas. Isso se deve, em parte, à resistência à insulina e, principalmente, ao processo inflamatório local. ^319^ Errazuriz et al. ^320^ verificaram que pacientes portadores de NAFLD tiveram a gordura hepática reduzida (avaliado por ressonância magnética [RM] espectroscópica) quando consumiram por 12 semanas dieta enriquecida com MONO (22% VCT) em comparação ao grupo controle (8% VCT). Tais alterações ocorreram mesmo sendo as dietas isocalóricas e sem perda ponderal entre os participantes ao final do estudo. ^320^

 No estudo randomizado de Bozzetto et al., ^321^ pacientes com DM2 receberam as seguintes intervenções: (1) dieta rica em MONO; ou (2) dieta rica em fibras e carboidratos de baixo índice glicêmico; ou (3) dieta rica em fibras e carboidratos de baixo índice glicêmico e exercício associado; ou (4) dieta rica em MONO associada ao exercício. Houve redução de até 30% do conteúdo lipídico hepático nos pacientes que receberam MONO, de forma independente do exercício. ^321^ O mesmo grupo de pesquisadores demonstrou, em estudo posterior, que a redução hepática de gordura se deveu à ativação de vias oxidativas hepáticas, a partir da mensuração de β-hidroxibutirato. Apesar de terem identificado aumento da β-oxidação, não verificaram aumento na razão palmitoleico:palmítico, o que infere que não houve diferença na ação da enzima SCD1. ^322^

 Adjacente às ações lipolíticas promovidas pelos MONO, a ação anti-inflamatória coordenada pelo ácido oleico pode estar envolvida na potência do restabelecimento das funções hepáticas, como demonstrado por Morari et al. ^323^ Em seu trabalho, células HepG2 tratadas com ácido oleico apresentaram aumento na expressão gênica e conteúdo proteico da IL10, proteína com potente ação anti-inflamatória. O oleico ativa a proteína PGC1α (coativador 1 alfa do receptor ativado por proliferadores de peroxissomos gama), que se liga à proteína cMAF. Em formato de dímero, PGC1α-cMAF migram ao núcleo e induzem transcrição exclusiva do gene da IL10. ^323^

 Da mesma forma, ácidos graxos POLI apresentam respostas metabólicas hepáticas distintas. Ácidos graxos ω6 (linoleico e araquidônico) e ω3 (ALA, EPA e DHA) participam do metabolismo hepático, mas são destinados majoritariamente à constituição de membranas celulares, sinalização intracelular como segundo mensageiros e outras funções, portanto, desviados de sua utilização como substrato energético. ^324^ Em 2007, Yamaguchi et al. ^325^ inibiram experimentalmente a síntese hepática de triglicérides. Apesar de terem observado melhora no quadro de esteatose, houve intensificação do dano hepático, que progrediu à fibrose e cirrose. Nesse estudo, foi observado que, com o aumento dos ácidos graxos livres no citoplasma, houve maior oxidação por espécies reativas de oxigênio, induzindo inflamação importante. ^325^

 Apesar de alguns estudos apontarem para a melhora no quadro da NAFLD, quando suplementos com ω3 são utilizados, ^326^ parece ainda haver inconsistência na literatura quanto aos seus benefícios. ^327 , 328^ Em um estudo randomizado com crianças portadoras de NAFLD, a ingestão diária de 1.300 mg de ω3, durante 6 meses, reduziu aspartato aminotransferase (AST) e gama-glutamil transpeptidase (γGT), além de aumentar adiponectina sérica, mas essas alterações não foram suficientes para reduzir o grau de esteatose evidenciado por ultrassom. ^329^ Parte das incongruências encontradas nos estudos deve-se, em geral, ao desenho experimental, à certificação do conteúdo da cápsula escolhida, à escolha do placebo, entre outros fatores. Tobin et al. ^330^ realizaram estudo randomizado, duplo-cego, em que trataram 291 pacientes com cápsula concentrada de ω3 (460 mg de EPA + 380 de DHA) ou placebo (óleo de oliva), por 24 semanas. Análise por ressonância magnética (RM-PDFF) mostrou redução significativa no conteúdo hepático de lípides, de forma semelhante, em ambos os grupos, resultado atribuído à aderência de padrão alimentar saudável. ^330^

 Apesar de ω3 reduzir a síntese de TG por meio do bloqueio do SREBP, ^83 , 331 , 332^ os resultados dos estudos clínicos ^329 , 330^ não suportam a recomendação de sua suplementação no tratamento da NAFLD e NASH, conforme discutido no posicionamento da American Association for The Study of Liver Disease. ^297^

### 9.4. Ácidos Graxos Trans e Esteato-hepatite Não Alcoólica

 Dieta hiperlipídica enriquecida com ácidos graxos *trans* induziu aumento da expressão de fatores de transcrição envolvidos na lipogênese hepática (SREBP-1c e PPAR-γ) e redução da MTP, sugerindo menor capacidade de exportar triglicérides, o que acarretou desenvolvimento de NASH. ^308^ Estudo que avaliou 4.242 participantes da coorte do NHANES mostrou associação positiva entre a concentração plasmática de ácidos graxos *trans* com NAFLD, a qual foi estimada por biomarcadores plasmáticos de função hepática, como fosfatase alcalina (ALP), alanina aminotransferase (ALT), aspartato aminotransferase (AST) e gama glutamil transferase (GGT). ^333^

 A composição da dieta pode influenciar o desenvolvimento de NAFLD, ^334^ e, neste contexto, o excesso de ácidos graxos SAT pode contribuir para o acúmulo intra-hepático de lípides. ^318^ Por outro lado, padrões alimentares saudáveis ricos em INSAT, como a dieta do Mediterrâneo, parecem ter efeitos benéficos, melhorando a esteatose mesmo na ausência da perda de peso. ^335 , 336^ Todavia, são necessários mais estudos prospectivos comparando o efeito de macronutrientes na NAFLD e que avaliem os componentes histopatológicos pré- e pós-tratamento. 

 O tratamento da NAFLD consiste, principalmente, em perda de peso, alcançada por meio de redução de cerca de 30% da ingestão calórica. A perda de 3% a 5% do peso corporal reduz a esteatose, e a redução de 7% a 10% do peso inicial contribui para melhora dos componentes histopatológicos de esteato-hepatite e fibrose. ^337^ A prática de atividade física, associada à restrição calórica, auxilia na perda e na manutenção do peso. ^297^

 Dessa forma, indivíduos portadores de NAFLD devem ser orientados à dieta de restrição calórica para perda de peso e prática de atividade física. Deve ser incentivada a adoção de padrões alimentares saudáveis, com conteúdo amplo e principalmente diversificado de frutas, verduras e legumes, além de incremento em carboidratos integrais em detrimento aos refinados, com priorização do consumo de gorduras INSAT e adequação do consumo saturados. ^297^

## 10. Metabolismo Lipídico do Tecido Adiposo

 O tecido adiposo é composto por adipócitos, pré-adipócitos, células imunes, fibroblastos, nódulos linfáticos e tecido nervoso. O adipócito é a única célula capaz de armazenar gordura sem comprometer sua funcionalidade, e sua função primária é promover lipogênese e lipólise. ^338^ Além disso, o tecido adiposo é capaz de secretar diversas substâncias bioativas como leptina, citocinas (TNF, IL6, MCP1, IL1β) e outras adipocinas, desempenhando função autócrina, parácrina e endócrina. ^339^ Tais ações podem ser moduladas por diferentes ácidos graxos provenientes da alimentação. 

 Em resposta ao excesso de energia e na tentativa de restabelecer a homeostase do tecido, o adiposo passa por processo de remodelação, caracterizado pela hipertrofia e hiperplasia dos adipócitos e secreção de citocinas em concentrações elevadas, que as caracterizam como pró-inflamatórias. ^340^ Todavia, cronicamente, a secreção de TNF-α, IL6, iNOS e MCP1, e o recrutamento de células inflamatórias como neutrófilos, células T e macrófagos, promovem inflamação, fibrose ^339 - 341^ e resistência à insulina no tecido adiposo, ^342^ que exerce papel fundamental no desarranjo metabólico característico da obesidade. ^343^

 A sinalização celular mediada pelos receptores de TNF-α culmina com ativação do NF-kB, que incrementa a secreção de citocinas e caracteriza a inflamação local. Nessa condição, o adipócito apresenta intensificação da lipólise com aumento da liberação de ácidos graxos livres (AGL). Os ácidos graxos SAT provenientes da lipólise dos adipócitos ativam os receptores TLR4 de macrófagos residentes no tecido, agravando a resposta inflamatória local, estabelecendo círculo vicioso. ^344^ Concomitante a essas ações, ocorre gradativamente a polarização de macrófago da subpopulação M2 (anti-inflamatório, ligado à resolução da lesão) para M1 (via clássica de ativação, associada a resposta Th1). Com isso, ocorre intensificação do estado inflamatório e indução de resistência à insulina no tecido adiposo. ^339^ Além disso, na obesidade, outros fatores como hipóxia do tecido adiposo, ERE e endotoxemia contribuem para manutenção da inflamação no tecido adiposo. 

 A insulina exerce importante efeito no tecido adiposo, pois inibe a lipólise e estimula a lipogênese e a captação de glicose e AGL. A ativação de vias inflamatórias antagoniza a ação da insulina ao induzir resistência ao hormônio, e favorece o surgimento de doenças associadas ao risco cardiometabólico. ^343^

 Os TG presentes nos quilomicrons (QMs), provenientes da dieta, são hidrolisados pela ação da LPL ^343^ liberando AGL que são direcionados para o tecido adiposo e em menor quantidade para o músculo. ^345^ Desta forma, o tipo de ácido graxo no tecido adiposo tem forte correlação com o ácido graxo da dieta. 

### 10.1. Ácidos Graxos Saturados e Metabolismo do Tecido Adiposo

 Estudo *in vitro* mostrou que a pré-incubação de adipócitos com ácido palmítico induziu hipertrofia das células, com consequente aumento da secreção de MCP1 e da concentração de hidroperóxido, marcador de estresse oxidativo. ^345^ Esses efeitos não foram observados na presença de ácido oleico. ^345 , 346^ Em outro estudo, o ácido palmítico ativou NF-kB e aumentou expressão de citocinas pró-inflamatórias em adipócitos 3T3-L1. ^347^ Em animais experimentais, dieta hiperlipídica rica em ácido láurico induziu ativação de citocinas pró-inflamatórias (TNF-α, IL6, MCP1, IL1β, IFNγ) e ativou serina-quinases como IKKβ e JNK no tecido adiposo, com redução na fosforilação da proteína adenosina monofosfato quinase (AMPK). ^348^ Por outro lado, aumentou a produção de citocinas com ação anti-inflamatória na tentativa de resgate da homeostase tecidual. ^348^

 Em animais, o consumo de dieta hiperlipídica rica em palmítico acarretou aumento do infiltrado de células dendríticas no tecido adiposo, simultaneamente ao desenvolvimento de resistência à insulina. Nas células dendríticas, o ácido palmítico induziu aumento da expressão de marcadores de maturação, como CD40, CD80, MHCII e TLR4. O aumento da expressão dos genes da caspase1 e IL1β sugere ativação paralela da via do inflamassomo, outra estrutura intracelular envolvida com o controle do tônus inflamatório. ^237^

 Estudo posterior do mesmo grupo mostrou que a dieta rica em saturados induziu resistência à insulina, reduziu a captação de glicose e aumentou as concentrações plasmáticas de insulina. Além disso, houve redução no tecido adiposo da expressão do IRS1 e da proteína de translocação de glicose-4 (GLUT4), bem como fosforilação em tirosina do IRS1 e da AKT. Esses efeitos não foram observados nos grupos submetidos à dieta rica em MONO. ^349^

 Kolak et al. ^350^ verificaram que, no tecido adiposo subcutâneo, o aumento do infiltrado de macrófagos, da expressão de MCP1 e PAI1 e também do acúmulo de ceramidas ocorreu de forma independente do IMC. Além disso, essas alterações se correlacionaram positivamente com o acúmulo hepático de lípides. 

 Estudo transversal realizado no Japão, em 484 indivíduos, mostrou que o consumo de SAT, avaliado pela concentração dos ácidos graxos em fosfolípides plasmáticos, se correlacionou com redução de adiponectina e aumento de resistina e visfatina, adipocinas relacionadas à resistência à insulina e adipogênese. ^351^

 Já estudo em indivíduos com sobrepeso que avaliou o consumo adicional de 1.000 Kcal/dia, provenientes de ácidos graxos SAT (óleo de coco e manteiga), INSAT (oliva e nozes) ou açúcar, mostrou que os ácidos graxos SAT induziram resistência à insulina e aumento da expressão de genes relacionados às vias inflamatórias no tecido adiposo. ^318^

### 10.2. Ácidos Graxos Insaturados e Metabolismo do Tecido Adiposo

 O tecido adiposo armazena com mais eficiência ácidos graxos SAT; entretanto, caso haja proporção elevada de INSAT no consumo alimentar, esse perfil poderá ser refletido no tecido adiposo. ^352^ Em virtude da dificuldade em se investigar o perfil de dispersão tecidual de ácidos graxos oriundos da alimentação em humanos, grande parte dos estudos é conduzida em animais. ^353^ Observou-se que a oferta por apenas 3 dias de dieta hiperlipídica a camundongos foi capaz de elevar as quantidades de palmítico e oleico no tecido adiposo, com preferência do depósito de oleico no tecido adiposo mesentérico. 

 O mesmo estudo mostrou que apenas o oleico foi capaz de alterar o perfil inflamatório de macrófagos M1 para o perfil anti-inflamatório M2, tanto no tecido dos animais quanto em cultura de adipócitos. ^353^ Como parte do estudo LIPIGENE, 39 pacientes portadores de risco cardiometabólico alimentados com dieta rica em oleico apresentaram aumento na expressão de genes controladores da autofagia (Beclin 1 e ATG7) e apoptose (CASP3 e CASP7) em comparação tanto ao grupo pobre em gordura e rico em carboidratos complexos como ao grupo rico em carboidratos complexos e ω3. ^354^

 Vários estudos vêm demonstrando a correlação entre o conteúdo de ácido araquidônico no tecido adiposo com casos de IAM. ^355 - 358^ Estudo caso-coorte demonstrou forte correlação (39% dos casos) com o conteúdo de ácido araquidônico do tecido adiposo e IAM. ^359^ Isso pode ser explicado pela rápida liberação de AA pelo adipócito, o qual é substrato para a síntese de eicosanoides pró-inflamatórios e pró-trombóticos, facilitando a inflamação e a desestabilização da placa aterosclerótica. Além disso, esse ácido graxo tem sido associado com a resistência à insulina, podendo aumentar o risco cardiovascular. ^359^

 O potencial anti-inflamatório reconhecido dos ácidos graxos ω3 parece interferir positivamente no controle da inflamação tecidual em pacientes, mas ainda são necessárias evidências mais robustas. Spencer et al. ^360^ trataram pacientes resistentes à insulina, mas não diabéticos, com 4 g de ω3 (etil-éster) por 12 semanas, e observaram redução significante da MCP1 e, consequentemente, macrófagos, no tecido adiposo, mas não no músculo. Esses fenômenos não foram acompanhados por redução na concentração plasmática de citocinas, sensibilidade à insulina ou adiponectina. No experimento de cocultura de adipócitos e macrófagos oriundos desses mesmos indivíduos, mesmo na presença de macrófagos, os adipócitos dos pacientes que consumiram ω3 apresentaram conteúdo reduzido de MCP1. ^360^ Potencialmente diferente, em um estudo controlado, randomizado e duplo-cego, gestantes com sobrepeso e obesidade foram suplementadas com 2 g de ω3 (EPA+DHA), 2 vezes ao dia, da 10ª semana de gestação ao nascimento. A concentração plasmática de PCR reduziu de modo significativo, seguida por redução do TLR4 no tecido adiposo e da diminuída expressão gênica de TNF, IL6, IL8, no tecido placentário. ^361^

As dificuldades na elaboração de recomendações gerais para consumo de ácidos graxos em portadores de enfermidades se devem à grande variação de protocolos experimentais, incluindo diferentes tipos de alimentos, tempo de aplicação das dietas, conflito de interesses dos autores dos estudos, qualidade crítica da informação científica entre outras.

 Em um estudo duplo-cego, placebo-controlado, indivíduos resistentes à insulina que receberam por 6 meses suplementação diária de 3,9 g de ω3 (EPA+DHA), submetidos a biópsia de tecido adiposo antes e depois da intervenção, não apresentaram qualquer benefício associado ao metabolismo nesse tecido. ^362^ Entretanto, quando investigado em células de adipócitos humanos, o EPA induziu aumento na expressão de genes relacionados ao *begeamento* do adipócito. Proteínas envolvidas com a biogênese mitocondrial foram incentivadas, como a proteína desacopladora mitocondrial 1 (UCP1) e a carnitina palmitoil transferase 1 (CPT1). Por outro lado, nesse mesmo estudo, o ácido araquidônico reduziu a respiração mitocondrial e, consecutivamente o gasto energético. ^363^ Por fim, dentro das evidências encontradas e consideradas a solidificarem as tomadas de decisão quanto à participação do ω3 e sua relação com o funcionamento do tecido adiposo, Iturari et al. ^364 , 363^ trataram 55 pacientes obesos, não diabéticos, elegíveis à cirurgia bariátrica, com 3,3 g de ω3 (EPA+DHA) durante 8 semanas. Houve redução significativa no tecido adiposo subcutâneo, do conteúdo das quimiocinas CCL2 e CCL3, da IL6, do HIF1-α (fator induzido por hipóxia) e do TGF-β (fator de crescimento transformador-beta) e CD40, além do aumento da adiponectina. O tecido adiposo visceral não apresentou alteração induzida pelo consumo de ω3 comparado ao grupo placebo. 

Apesar dos potenciais benefícios metabólicos desencadeados pelo consumo de ácidos graxos ω3, até o momento, a grande divergência na literatura não dá suporte como evidência relevante para o tratamento do dismetabolismo quanto ao funcionamento do tecido adiposo. Por outro lado, ácidos graxos MONO apresentam número maior de evidências que sustentam benefícios metabólicos incrementais às condições associadas ao dismetabolismo.

## 11. Alimentos

### 11.1. Óleo de Coco

 O óleo de coco é composto, quase em sua totalidade (92%), por ácidos graxos SAT, dos quais o láurico representa cerca de 50%, seguido de mirístico (16%), palmítico (8%) e o restante composto por caprílico, cáprico e esteárico. Com relação às concentrações de ácidos graxos essenciais, o óleo de coco contém baixa concentração de ácido linoleico (18:2) e não contém ácido linolênico (18:3). ^43 , 365^

 Os maiores produtores de óleo de coco são Filipinas, Indonésia e Índia, que extraem do coco duas versões: o óleo refinado, clareado e desodorizado, e o óleo de coco virgem, extraído a frio, sem processos de refinamento. ^366^ O consumo de óleo de coco cresceu significativamente nos últimos anos, e parte disso se deve ao fato de suas propriedades terem sido erroneamente associadas às dos triglicérides de cadeia média (TCM), formados principalmente por ácidos caprílico-8:0 e cáprico-10:0, ^367^ os quais são absorvidos ligados à albumina e atingem o fígado via sistema portal, e por isso não elevam a trigliceridemia. Já o láurico, principal ácido graxo do óleo de coco, é, em grande parte, transportado por via linfática após sua absorção, ^38^ e a sua presença nos quilomícrons é dose-dependente. ^38^

 É possível que associações benéficas a respeito do consumo de óleo de coco decorram de estudo realizado em habitantes das ilhas Pukapuka e Tokelau, na Polinésia, que apresentam baixa incidência de DCV. A dieta típica dessa população é rica em gordura saturada, sendo o coco a principal fonte de gordura e energia; o consumo de proteína provém principalmente de peixes e o carboidrato, de frutas nativas como a fruta-pão. Além disso, a dieta é rica em fibras, pobre em sacarose e produtos industrializados, sendo estes de difícil acesso para os habitantes da ilha. ^368^ Esse cenário se modificou nas últimas décadas, possivelmente em razão da migração para hábitos alimentares Ocidentais, mesmo tendo sido mantido o consumo de coco. Em 2010, cerca de 40% da população da Polinésia foi diagnosticada com doenças crônicas (DCV, DM2 e HAS), e estas foram responsáveis por três quartos das mortes no arquipélago. ^369^

 O óleo de coco é capaz de aumentar as concentrações plasmáticas de CT e LDLc comparado ao consumo de outras gorduras como óleo de oliva ^370^ e cártamo. ^371^ Estudo em humanos mostrou que láurico eleva CT e LDLc comparado à dieta rica em MONO, no entanto, de forma menos pronunciada que palmítico. ^372 , 373^ Mendis et al. ^373^ verificaram que a substituição isocalórica do óleo de coco, tipicamente presente na dieta dos habitantes do Sri Lanka, por óleo de soja, rico em POLI, reduziu as concentrações plasmáticas de CT, LDLc e TG em indivíduos normolipidêmicos. O mesmo resultado foi observado com óleo de milho em indivíduos dislipidêmicos. ^219^

 Além disso, estudos mostrando elevação das concentrações de HDLc com consumo de coco mostraram aumento concomitante de LDLc, o qual sabidamente eleva o risco CV. ^374^

 Sabe-se que os ácidos graxos SAT ativam vias de sinalização inflamatória, bem como ERE, autofagia e apoptose, por meio da ativação dos receptores *Toll-like* (TLRs), ligados à resposta imune inata. ^375^ Os TLRs fazem o reconhecimento de padrões moleculares associados a patógenos (PAMPs), como o LPS, presente na parede celular de bactérias gram-negativas, e alertam o sistema imunológico. Quando ativados, os TLRs deflagram sinalização que culmina com a transcrição e secreção de citocinas pró-inflamatórias. ^375^

 O ácido láurico, dentre os ácidos graxos SAT, é o que possui maior potencial inflamatório. ^241^ Estudo *in vitro* em macrófagos mostrou que o ácido láurico induziu ativação de NF-κB, acarretando aumento da expressão de ciclo-oxigenase 2 (COX 2) por meio da ativação de TLR 2 e 4. ^376^ A capacidade do ácido láurico em ativar vias inflamatórias, por meio da ativação de TLR4 levando a secreção de citocinas inflamatórias e ativação de células T, já foi descrita em diferentes tipos celulares. ^241 , 377^

 Estudo que comparou o efeito do consumo de óleo de coco, palma ou oliva por 5 semanas em parâmetros inflamatórios de indivíduos normocolesterolêmicos não observou diferença nas concentrações plasmáticas de homocisteína e marcadores inflamatórios como TNF-α, IL-1β, IL-6, INF-γ e IL-8. Entretanto, nesse estudo, o desvio padrão foi excessivamente grande, podendo ter mascarado diferenças no perfil inflamatório. ^370^

 Valente et al. ^378^ avaliaram o efeito agudo de dieta rica em óleo de coco comparado ao azeite de oliva em 15 mulheres com sobrepeso, e não observaram diferença com relação ao metabolismo energético e oxidação lipídica. 

Com relação às propriedades antioxidantes atribuídas aos polifenóis presentes no óleo de coco virgem, os estudos são ainda preliminares, realizados, em sua maioria, em animais experimentais, não podendo ser traduzidos para humanos.

Até o momento não há na literatura estudos clínicos randomizados e controlados e estudos epidemiológicos que avaliem o efeito do óleo de coco no perfil lipídico, inflamatório e desfecho cardiovascular. Desta forma, não há evidência para sua indicação em substituição a outros óleos vegetais ricos em INSAT.

### 11.2. Óleo de Palma

 O óleo de palma, juntamente com as gorduras interesterificadas, vem sendo amplamente utilizado pela indústria em substituição ao uso de gordura *trans* nos alimentos. Apesar de se tratar de óleo vegetal, o óleo de palma é composto por ácidos graxos SAT (45% de ácido palmítico e 5% de ácido esteárico) e INSAT (40% ácido oleico e 10% ácido linoleico). Dessa forma, o aumento no consumo direto de óleo de palma, ou indireto, por meio da ingestão de produtos industrializados, contribuirá para maior consumo de SAT que elevam o risco CV. 

 Em humanos, o consumo de dieta enriquecida com óleo de palma elevou as concentrações plasmáticas de CT e LDLc, comparado ao consumo de óleo vegetal rico em INSAT. ^379^ Metanálise de estudos de intervenção verificou que, em comparação ao consumo de óleos vegetais com baixas concentrações de saturados, como canola, soja e oliva, o óleo de palma elevou as concentrações de CT, LDLc, e, de forma modesta, HDLc, resultados condizentes com o efeito de ácidos graxos SAT no perfil de lipoproteínas. Comparado ao consumo de gordura *trans* , a elevação do HDLc foi mais proeminente, uma vez que o consumo de *trans* reduz suas concentrações. ^380^ Por outro lado, o consumo de óleo de palma parece ter efeitos semelhantes ao consumo de gordura animal em relação aos lípides plasmáticos. ^380 , 381^

O consumo de óleo de palma deve ser mantido dentro do percentual de recomendação de consumo de SAT. Apesar de vegetal, o óleo de palma é bastante rico em palmítico e, dessa forma, parece se comportar de modo semelhante às gorduras de origem animal.

### 11.3. Chocolate

 O chocolate é obtido da semente do cacaueiro, proveniente principalmente de países da América do Sul e costa oeste da África. Além de cacau, manteiga de cacau, açúcar, leite e lecitina, outros ingredientes como castanhas, cereais e frutas podem ser incorporados na fabricação do chocolate, caracterizando-o como produto de alta densidade energética, rico em carboidratos e gorduras. O chocolate também contém polifenóis e minerais, tais como potássio, magnésio, ferro e zinco. Aproximadamente 63% da gordura do cacau é constituída pelos ácidos graxos SAT esteárico (34%) e palmítico (27%). Os 37% restantes estão sob a forma de MONO (33,5%) e POLI (3,5%). ^382^

 Por ser rica em esteárico, a gordura do cacau exerce efeito neutro sobre a colesterolemia. Estudo em humanos que investigaram o consumo alimentar mostram que, em comparação a palmítico, o consumo de esteárico reduziu as concentrações plasmáticas de CT e LDLc de forma similar ao ácido oleico. Além disso, o consumo de esteárico elevou as concentrações de oleico em CE e TG plasmáticos, ^383^ isso porque o esteárico é rapidamente convertido a oleico no fígado pela ação da SCD1. ^48^ Dados mais recentes do estudo EPIC mostraram associação positiva entre as concentrações de esteárico em fosfolípides plasmáticos tanto para risco de doença coronariana ^108^ como de DM2. ^14^ Entretanto, deve-se considerar que o esteárico é também produzido endogenamente pela lipogênese *de novo* (DNL). 

O consumo de esteárico parece ter efeito neutro sobre a colesterolemia; todavia, deve-se levar em conta que o chocolate é também fonte de calorias e de açúcar simples, podendo contribuir para o ganho de peso e aumento do risco CV.

### 11.4. Manteiga

 A manteiga é formada a partir do creme obtido do desnatamento do leite; desta forma, sua gordura advém exclusivamente de gordura láctea. Em uma porção de manteiga, cerca de 51,5% dos ácidos graxos são saturados, entre eles destacam-se palmítico (24%), esteárico (10%), mirístico (8%) e láurico (2%), sendo o restante composto por MONO (22%) e POLI (1,5%). ^25^

 Estudo randomizado que avaliou impacto do consumo de SAT proveniente de manteiga em comparação a dietas isocalórica ricas em INSAT, no risco cardiometabólico, mostrou que o consumo de manteiga elevou as concentrações de CT, LDLc e ApoB. ^384^ Em estudo de coorte prospectivo, com mais de 26.000 indivíduos, o consumo de manteiga, juntamente com leite e derivados, foi inversamente associado à incidência de DM2. ^385^ Em duas outras coortes, acompanhadas por 10 e 20 anos, não foi encontrada associação entre o consumo de manteiga e DCV. ^386 , 387^ Entretanto, deve-se ressaltar que na coorte do estudo MESA, ^387^ mesmo no maior quintil, a mediana de consumo de manteiga foi inferior a 5 g/dia por pessoa. 

 Revisão sistemática de estudos de coorte de alto grau de evidência não observou associação entre o consumo de manteiga e risco de DCV, DAC e AVC. Por outro lado, verificou associação inversa com risco de DM2. ^388^

Os resultados dos estudos devem ser interpretados com cautela, uma vez que se tem bastante consolidada as ações dos ácidos graxos SAT em lípides plasmáticos e saúde CV. O uso de manteiga deve estar inserido em padrão alimentar saudável e individualizado, considerando o valor calórico agregado, e buscando adequação do peso quando necessário.

### 11.5. Lácteos

 O leite e seus derivados são importante fonte de cálcio e proteína de alto valor biológico. Por outro lado, o consumo de lácteos integrais pode elevar o consumo de ácidos graxos SAT, especialmente mirístico, que possui forte correlação com aumento do risco CV. O consumo de lácteos desnatados é parte das recomendações da dieta DASH, padrão alimentar originalmente desenvolvido para o tratamento de hipertensão, que, por apresentar benefícios cardiometabólicos, ^389^ é recomendado como plano saudável para todos os adultos. ^390^

 Mais recentemente, estudos têm demonstrado que o consumo de lácteos se associa inversamente ao risco de DM2 ^14 , 391^ AVC ^392^ e DCV. ^110^ Nesses estudos, as concentrações plasmáticas dos saturados pentadecanoico (15:0) e heptadecanoico (17:0) foram utilizadas como marcadores de consumo de lácteos, uma vez que, por não serem sintetizados endogenamente, estes devem ser obtidos da dieta, sendo os lácteos sua principal fonte. 

 É importante ressaltar que a matriz alimentar é fator determinante no risco CV, uma vez que, além de macronutrientes, os alimentos fornecem micronutrientes e fibras que, quando inseridos no contexto do padrão alimentar saudável, contribuem para desfecho CV favorável. Por outro lado, a inclusão de alimentos industrializados, ricos em açúcar simples, refinados e aditivos, como corantes, conservantes e espessantes, podem impactar negativamente no risco CV. Além disso, o uso de fármacos hipolipemiantes, como as estatinas, pode atenuar ou até mesmo mascarar os efeitos do consumo de SAT sobre o risco CV. ^106^

### 11.6. Carnes

 As principais carnes consumidas são de boi, frango e suínos, e, do ponto de vista nutricional, são importantes fontes de proteínas de alto valor biológico, fornecendo todos os aminoácidos essenciais, vitaminas e minerais. A quantidade de gordura, bem como a distribuição de ácidos graxos, varia de acordo com o animal e o tipo de corte. De maneira geral, as carnes contêm principalmente MONO e SAT (especialmente palmítico e esteárico) e uma pequena quantidade de POLI. ^25 , 28^

 Associação positiva entre consumo de carnes e risco CV foi observada em alguns estudos, ^110^ mas não em outros. ^393^ Estudo conduzido em mais de 780 indivíduos verificou que o consumo de carnes vermelhas e processadas se correlacionou a padrão alimentar menos saudável, porém não se associou a marcadores de risco de DCV e DM2. ^394^ Já estudo de coorte prospectivo, com mais de 74 mil indivíduos, mostrou associação entre maior consumo de carnes (processadas e não processadas) e aumento do risco de mortalidade CV (tal associação ocorreu mesmo nos indivíduos com maior consumo de frutas e vegetais). ^395^

 O aumento do risco de mortalidade total e por DCV foi associado ao maior consumo de carnes vermelhas e processadas, mas não ao consumo isolado de carnes não processadas em duas metanálises. ^396 , 397^ Carnes processadas são também ricas em sódio e compostos nitrogenados, como nitratos, que podem contribuir para seu efeito deletério no risco CV, em razão de seus efeitos em pressão arterial e função endotelial. 

Já está bem estabelecido que o alto consumo de carnes vermelhas e processadas se associa ao aumento de risco CV, razão pela qual sua ingestão deve ser moderada e estar de acordo com o total de ácidos graxos SAT recomendados na dieta.

### 12. Microbiota Intestinal

 As dietas hiperlipídicas, especialmente aquelas ricas em ácidos graxos SAT, são capazes de alterar a composição da microbiota intestinal, ^398 - 400^ induzir diminuição de diversidade bacteriana, aumento da permeabilidade intestinal, endotoxemia metabólica e inflamação sistêmica de baixo grau, ^401 - 407^ influenciando no desenvolvimento de diversas doenças crônicas como obesidade, diabetes e aterosclerose. ^408^ A perda da integridade do epitélio intestinal permite que o LPS, proveniente da membrana celular de bactérias gram-negativas, transloque para o plasma, culminando em endotoxemia metabólica. ^401 , 403^ . 

 Já se observou que o maior consumo de dietas ricas em ácidos graxos SAT aumenta a permeabilidade paracelular intestinal, por interferir em proteínas do complexo das *tight-junctions* (TJ), e com isso elevam as concentrações plasmáticas de LPS. ^409 , 410^ Alterações na permeabilidade intestinal estão relacionadas com a regulação das TJ, complexo proteico que mantém as junções célula-célula no epitélio intestinal, formando uma barreira contra a passagem de macromoléculas. ^411^

 Estudo em camundongos observou que dieta rica em SAT induziu maior formação de ácido taurocólico, o que permitiu a expansão de bactérias redutoras de sulfato, como a *Bilophila wadsworthia* , efeito que não foi observado com dieta rica em POLI. Este estudo mostra que a alteração na composição de ácidos biliares, pelo tipo de gordura da dieta, pode acarretar disbiose, comprometendo a homeostase do hospedeiro. ^400^

 O aumento da permeabilidade intestinal induzido pela dieta rica em gordura, principalmente de ácidos graxos SAT, induz alteração da microbiota intestinal e aumento da resposta inflamatória, deflagrada pela ativação do TLR4 pelo LPS. ^412^ Outro mecanismo pode estar associado à diminuição da secreção da enzima IAP ( *Intestinal alkaline phosphatase)* pela borda em escova do duodeno, a qual é responsável por detoxificar o LPS protegendo contra endotoxemia. ^413^

 Estudo experimental demonstrou que a dieta hiperlipídica, especialmente quando associada à dieta rica em açúcar, induz disbiose, inflamação no epitélio intestinal e altera a ativação da via vagal aferente, ações que podem impedir a regulação adequada da ingestão de alimentos, contribuindo para hiperfagia e desenvolvimento de obesidade. ^414^

### 12.1. Padrões Alimentares e Microbiota Intestinal

Os componentes da dieta exercem importante impacto sobre o perfil da microbiota. Desta forma, os diferentes padrões alimentares são capazes de modular a microbiota intestinal de forma distinta.

 Estudo que investigou a associação de variáveis dietéticas com a microbiota intestinal identificou 97 nutrientes associados aos dados de abundância relativa ou com a presença/ausência de microbiomas. Os nutrientes foram ordenados em quatro grupos: aminoácidos e colina; carboidratos; gorduras; e fibras e vegetais. Observou-se que os grupos gorduras *versus* fibras associaram-se de forma antagônica com a abundância de bactérias, ^415^ ou seja, aqueles que se associaram positivamente com gordura tendem a se associar negativamente com fibras. O mesmo padrão de associação foi visto para o grupo aminoácidos e proteínas *versus* carboidratos e grupo gordura *versus* carboidratos. Além disso, as taxas microbianas correlacionadas ao IMC também se correlacionaram com maior consumo de gordura e ácidos graxos SAT. ^415^

 Recente estudo randomizado, conduzido em 217 indivíduos saudáveis, comparou o efeito de dietas isocalóricas, contendo concentrações crescentes de gordura (20%, 30% e 40%) e a mesma quantidade de fibras (14 g/dia). ^416^ A dieta rica em gordura aumentou as concentrações fecais de ácido palmítico, esteárico e araquidônico. Este último foi positivamente associado com aumento das concentrações plasmáticas de mediadores inflamatórios como PCR e prostaglandina E2 (PGE2) e tromboxano B2 (TXB2), ambos derivados do ácido araquidônico. Um resultado importante deste estudo é o fato de que, mesmo com quantidades adequadas de fibras na dieta, o alto consumo de gorduras impediu a formação de ácidos graxos de cadeia curta (AGCC) pelas bactérias. ^416^ Além disso, neste estudo, o maior consumo de gordura reduziu a diversidade bacteriana. 

### 12.2. Importância do Padrão Alimentar na Síntese de Ácidos Graxos de Cadeia Curta

 A produção de hidrolases glicosídicas, responsáveis pela quebra de determinados sacarídeos, é muito limitada no organismo humano. Por outro lado, determinadas bactérias intestinais codificam enzimas capazes de digerir uma ampla gama de polissacarídeos, como as fibras. ^417^ A fermentação das fibras solúveis promove a formação de AGCC, principalmente propionato (C3), acetato (C4), butirato (C5), que, além de servirem como substrato energético para os colonócitos, exercem ações sistêmicas, como o favorecimento da homeostase da glicose. ^418 , 419^

 A presença de AGCC induz secreção de incretinas intestinais, como GLP-1 e PYY, que atuam no sistema nervoso central (SNC) promovendo saciedade e diminuição do consumo alimentar, diminuição do tempo de esvaziamento gástrico, aumento do trânsito intestinal, além de estimular a síntese e a secreção de insulina pelo pâncreas. ^418^

 A redução no consumo de fibras pode impactar a composição da microbiota intestinal e a produção de AGCC. Estudo prospectivo, realizado em 17 indivíduos obesos, avaliou o impacto de duas dietas hiperproteicas e hiperlipídicas e pobres em fibras. Os dados mostram que ambas as dietas diminuíram a produção fecal de AGCC, e aumentaram a concentração de ácidos graxos de cadeia ramificada, ácido fenilacético e compostos nitrosos, metabólitos deletérios para a saúde do cólon. ^420^

## 13. Colesterol Alimentar

### 13.1. Concentração Plasmática de Lípides e Lipoproteínas

 A relação entre o colesterol da dieta e o CT plasmático tem sido evidenciada como sendo linear em estudos observacionais de coortes. ^421 , 422^ Entretanto, estudos observacionais apresentam limitações, tais como a presença de variáveis de confusão que podem ampliar correlações positiva ou negativa e a existência de vieses de seleção. ^423^ Além disso, o consumo de colesterol na dieta geralmente está associado a aumento do consumo de ácidos graxos SAT, que sabidamente aumentam o LDLc e o risco de DCV. ^424^

 Nos últimos anos, tem havido intenso questionamento sobre o papel do colesterol alimentar na incidência de complicações ateroscleróticas. Em resposta a isso, a AHA deixou de limitar o consumo de ovos como forma de proteção contra as DCV. Assim, as Diretrizes Dietéticas para Americanos retiraram a recomendação de que se restringisse a ingestão de colesterol a não mais do que 300 mg por dia. ^7^ Entretanto, sugerem que o colesterol da dieta permanece importante e deve ser considerado para elaboração de padrões alimentares saudáveis. Enfatizam ainda que o consumo de colesterol proveniente da dieta deve ser o menor possível, como recomendado pelo Instituto de Medicina. ^425^ Como referido, em geral, as fontes alimentares que contêm altas quantidades de colesterol são também ricas em ácidos graxos SAT, tais como carnes gordurosas e laticínios ricos em gorduras. Assim, a recomendação foca na limitação de gorduras saturadas em menos de 10% ao dia, que deve ser suficiente para limitar também o colesterol alimentar. ^7^

 Vale ressaltar que nem todas as pessoas reagem da mesma forma ao consumo do colesterol alimentar, pois a resposta é altamente variável, dependendo de fatores genéticos e metabólicos ^426 , 427^ As respostas do perfil lipídico ao colesterol alimentar foram examinadas em 19 estudos de intervenção. O consumo de colesterol, oriundo principalmente de ovos, levou a aumento tanto do LDLc como do HDLc, resultando em aumento discreto da relação LDLc/HDLc. Entretanto, a análise dessa relação pode ser muito simplista, uma vez que, enquanto o LDLc é excelente marcador do risco cardiovascular e modificações de suas taxas mostram importante relação com o risco cardiovascular, as alterações do HDLc não expressam possíveis mudanças da funcionalidade das partículas de HDL, que sabidamente vão muito além do transporte reverso do colesterol. ^428^

 O consumo de colesterol até 400 mg/dia, proveniente do ovo, não está associado ao aumento das concentrações plasmáticas de triglicérides em indivíduos acima do peso portadores de diabetes ou pré-diabetes. ^429^

### 13.2. Risco de Desenvolvimento de Diabetes Melito Tipo 2

 Estudos observacionais e randomizados apresentam resultados conflitantes a respeito da associação entre o consumo alimentar de colesterol e risco de DM2. Estudo caso-controle demonstrou aumento de 2 vezes no risco de DM2 nos indivíduos que consumiram 3 a 4,9 ovos/sem e de 3 vezes nos que consumiram mais de 5 ovos/sem, após ajustes de fatores de confusão como IMC, história familiar de diabetes, tabagismo, atividade física e concentração plasmática de triglicérides. ^430^ Investigação que utilizou dados de dois estudos prospectivos e randomizados Physicians’ Health Study I (1982-2007) e Women’s Health Study (1992-2007) demonstrou que, durante o período de seguimento (20 anos em homens e 11,7 anos em mulheres), o desenvolvimento de diabetes foi maior nos que consumiram mais de 5 ovos por semana nos homens e acima de 7 ovos por semana nas mulheres, após ajustes multivariados. ^431^ No entanto, outros estudos com populações de diferentes regiões não evidenciaram a mesma associação. Estudo prospectivo na população japonesa (Japan Public Health Center-based Prospective Study), com seguimento de 5 anos, concluiu que a alta ingestão alimentar de colesterol ou de ovos não foi associada ao maior risco de DM2. ^432^ Resultados opostos foram observados na população masculina do Kuopio Ischemic Heart Disease Risk Factor Study, com seguimento de 19,3 anos, na qual o maior consumo de ovos foi associado ao menor risco de DM2. ^433^

 No estudo Jackson Heart Study, que avaliou a população afro-americana, constatou-se maior prevalência de DM2 nos que consumiram maior quantidade de ovos (> 5 ovos/sem *vs.* < 1ovo/mês), porém, análise prospectiva não mostrou associação entre o consumo de ovos e incidência de DM. ^434^

 Entre as revisões sistemáticas e metanálises que avaliaram indivíduos saudáveis também não houve consenso da associação do consumo de ovos e maior risco de DCV e DM2. ^435 , 436^ Em parte, esses resultados podem ser explicados por fatores de confusão como a ingestão de gordura saturada e quantidade calórica, que favorecem o ganho de peso e o desenvolvimento de síndrome metabólica. ^437^

### 13.3. Risco de Doenças Cardiovasculares em Diabetes Melito Tipo 2

Outra questão também em discussão é o papel do colesterol alimentar no risco CV em indivíduos portadores de DM2 ou síndrome metabólica.

 Estudos observacionais e prospectivos associam o consumo de ovos com maior risco de DCV na população geral, enquanto outros demonstram a associação somente nos indivíduos portadores de DM2. ^438^ Estudo de metanálise concluiu que o consumo de > 1 ovo/dia aumentou em 1,69 vez o risco de desenvolver DCV comparado aos que não consumiam ovo ou consumiam < 1 ovo/sem. No entanto, o consumo de ovo não foi associado com mortalidade. ^439^

 Estudo randomizado em indivíduos pré-diabéticos ou DM2 (DIADEGG Study), submetidos à dieta com alto (2 ovos/dia por 6 dias/sem) ou baixo consumo de ovos (< 2 ovos/sem) durante 3 meses, concluiu que o maior consumo de colesterol alimentar não alterou as concentrações plasmáticas de HDLc, LDLc e CT, evidenciando também que não houve aumento nos fatores de risco para DCV em portadores de DM2. ^429^

 Na população do NHS, demonstrou-se que, em portadoras de DM2, o menor consumo de colesterol alimentar, avaliado pela ingestão de ovos e carnes, foi associado com qualidade de vida mais saudável e, portanto, menor risco de DCV. Quando foram controlados os fatores de qualidade de vida, a associação entre consumo de colesterol e risco de DCV foi atenuada, sugerindo que a melhora na qualidade de vida também está associada ao risco cardiovascular, e não apenas a colesterol alimentar. ^440^

 Resultados baseados na população do Framingham Offspring Study, seguida por 20 anos, demonstraram falta de associação entre o consumo de colesterol alimentar e perfil lipídico em jejum e também no risco de DCV de indivíduos portadores de glicemia de jejum alterada ou DM2. ^441^

 Análise do estudo prospectivo da população do estudo PREDIMED, que incluiu participantes sem eventos cardiovasculares prévios e acompanhados em média por 5,8 anos, concluiu que o consumo baixo ou moderado de ovos não aumentou o risco de DCV tanto nos indivíduos portadores quanto nos não portadores de DM2. ^442^

Resultados de estudos prospectivos randomizados e observacionais, assim como as revisões sistemáticas e metanálises, são inconclusivos para a associação entre o maior consumo alimentar de colesterol e maior risco de DCV nos casos de DM2, em virtude da grande heterogeneidade das populações avaliadas e metodologias aplicadas.

### 13.4. Impacto nas Doenças Cardiovasculares

 As evidências científicas disponíveis são conflitantes em relação ao impacto do consumo de colesterol no risco CV. Vários estudos apontam ausência de associação entre colesterol dietético e DAC e AVC, ainda que existam limitações para avaliação dos resultados. ^427 , 443 , 444^ Em asiáticos, o maior quartil de consumo de colesterol alimentar não apresentou correlação com aumento de aterosclerose subclínica identificada pelo escore de cálcio. ^445^ Em finlandeses, o consumo de mais de 400 mg de colesterol por dia não se associou ao aumento da espessura da camada íntima-média da carótida ou a incidência de doença arterial coronária. ^446^ Entretanto, em americanos, o incremento de 300 mg de colesterol na dieta-base contendo em média 300 mg de colesterol/dia foi associado a aumento de 17% do risco de incidência de DCV. ^447^

 O alto consumo de colesterol pode apresentar associação com o aumento do risco de desenvolvimento de DCV, sendo recomendado monitoramento do seu consumo, pois o aumento do risco pode ser dose dependente. ^447^

## 14. Ovo

 O ovo é fonte alimentar de colesterol, pobre em ácidos graxos SAT, com alta densidade nutricional e baixo custo. O ovo de galinha (50 g) contém proteína de alto valor biológico (7,5 g), gordura saturada (1,6 g), gordura MONO (1,8 g), gordura POLI (0,9 g) e cerca de 200 mg de colesterol. A gema do ovo é, ainda, rica em colina (147 mg), nutriente essencial para o fígado e funções musculares. ^25 , 448^

 O impacto do consumo de ovos sobre perfil lipídico é bastante variável. ^449^ Em adolescentes saudáveis, o consumo de mais de 3 ovos por semana não está associado à alteração no perfil lipídico. ^450^ Da mesma maneira, em adultos normolipidêmicos e fisicamente ativos, o consumo de 2 ovos por dia não alterou as concentrações plasmáticas de lipoproteínas após 12 semanas de estudo. ^451^ Por outro lado, metanálise realizada com 28 estudos, avaliando o consumo entre 5 ovos por semana até 3 ovos por dia, indicou que o consumo de ovos em indivíduos hiper-responsivos aumenta a concentração de CT em 5,60 mg/dL (95% CI: 3,11, 8,09; P < 0,0001), LDLc em 5,55 mg/dL (95% CI: 3,14-7,69; P < 0,0001), HDLc em 2,13 mg/dL (95% CI: 1,10-3,16; P < 0,0001), tendo efeito neutro na concentração de triglicérides comparado aos indivíduos que não consumiam ovos. ^452^ Ainda assim, existem evidências de que o consumo de ovos está associado a LDLc de maior diâmetro, a qual apresenta menor suscetibilidade à oxidação e penetração no endotélio. ^449^

 No âmbito do impacto do consumo de ovos e risco de DCV, os achados permanecem conflitantes. Metanálise que avaliou o impacto do consumo de 1 ovo ao dia *versus* < 2 ovos por semana no risco de DAC e AVC, em 7 estudos de baixa heterogeneidade, não observou associação entre consumo de ovos e risco coronariano. ^453^ Por outro lado, foi verificada redução de 12% no risco de AVC com o maior consumo de ovo, sem relação dose-resposta na tendência de risco para AVC com o aumento do consumo de ovos. ^453^

 Em estudo de coorte na população chinesa, o alto consumo de ovos (7 ou mais ovos por semana) comparado ao baixo consumo de ovos (< 1 ovo por semana) não foi associado a mortalidade cardiovascular, DAC ou AVC. ^454^ Em outro estudo analisando coortes de população americana, considerando o consumo médio de 0,5 ovo ao dia (3 a 4 ovos/semana), concluiu-se que, com o aumento de cada 0,5 ovo ao dia, há elevação de 6% no risco de DCV (95% IC, 1,03-1,10) e aumento de 8% na mortalidade total (95% IC, 1,04-1,11). No entanto, após ajuste da análise estatística para o consumo de colesterol, ambas as associações perderam significância, apresentando RR ajustado de 0,99 (95% IC, 0,93-1,05) para incidência de DCV e RR ajustado de 1,03 (95% IC, 0,97-1,09) para mortalidade por todas as causas. ^447^ A análise recente dos resultados de três estudos de corte prospectivos que incluiu 177.000 individuos mostrou que o consumo moderado de ovo (1/dia) não foi associado ao aumento de risco de mortalidade ou de doenças cardiovasculares. ^455^

 Entre indivíduos com alto risco cardiovascular, o grau de aterosclerose, avaliado pela cineangiocoronariografia, apresentou-se menor entre indivíduos com consumo > 1 ovo por semana em comparação aos que relataram consumir < 1 ovo por semana. ^456^ De modo similar, o consumo de 2 ovos por dia por 6 semanas não afetou a função endotelial de indivíduos portadores de DAC. ^457^

 Revisão sistemática de estudos de coorte, em portadores de DM 2, concluiu que o consumo de 1 ovo ou mais/dia aumentou em 69% o risco para o desenvolvimento de DCV (IAM, DAC, AVC e cardiopatia isquêmica) quando comparados com indivíduos que ingeriram < 1 ovo /semana, sem associação com aumento de mortalidade. ^439^

 Com relação à IC, estudo suíço analisando o resultado de duas coortes prospectivas concluiu que o consumo diário de ovo não aumentou o risco de IC entre homens e mulheres, porém o consumo > 1 ovo por dia aumentou em 30% o risco de IC entre os homens, sem ainda efeito causal definido. ^444^

Análise conjunta das evidências atuais não é capaz de estabelecer relação de causalidade entre consumo de ovos e DCV. Entretanto, a divergência de resultados de estudos observacionais sugere cautela de consumo especialmente entre portadores de DM2 e indivíduos hiper-responsivos ao colesterol alimentar. Por se tratar de um alimento de alta densidade nutritiva e proteica, sugere-se que faça parte da dieta, desde que integrante de um padrão alimentar saudável.

### 14.1. N-óxido de Trimetilamina nas Doenças Cardiovasculares

 Estudos mostram que a microbiota intestinal está envolvida no desenvolvimento da DAC, ^458^ sendo o N-óxido de trimetilamina (TMAO) um dos focos de pesquisa mais emergente sobre a progressão da aterosclerose. TMAO é um óxido de amina que pode ser tanto encontrado naturalmente na dieta como também metabolizado a partir da colina (abundante em ovos), carnitina (presente na carne vermelha), betaína, fosfatidilcolina. Esses precursores são convertidos em trimetilamina (TMA) no intestino delgado, por bactérias específicas como firmicutes, proteobactérias e actinobactérias presentes na microbiota intestinal. ^459 , 460^ TMA é então absorvida e oxidada em TMAO através de reação reversível no fígado, catalisada pela enzima flavina monoxigenase 3 (FMO3). ^461^

 O peixe parece ser a maior fonte alimentar de TMAO. Estudos após ingestão de peixes mostram elevação nas concentrações plasmáticas de TMAO (50 vezes maior), quando comparado com outros alimentos fonte de carnitina ou colina. Entretanto, a excreção urinária de TMAO e dimetilamina (derivado de TMA) após o consumo de peixe é mais elevada em comparação ao consumo de carnes, laticínios, frutas, legumes ou grãos. ^462 - 464^

 Elevadas concentrações plasmáticas de TMAO foram correlacionadas com maior risco de grandes eventos cardiovasculares, prevalência de DCV, piora do prognóstico e aumento do risco de morte. ^461^ Isso porque TMAO pode exacerbar a resposta inflamatória da parede vascular e induzir a produção de espécies reativas de oxigênio. Mais recentemente, foi demonstrado o papel do TMAO na modulação do metabolismo do colesterol e dos ácidos biliares, promovendo progresso da aterosclerose. ^463^

 Um dos mecanismos pelo qual TMAO pode contribuir para o avanço da DCV é aumentar a expressão de receptores do tipo *scavenger,* responsáveis pela captação de LDL oxidada, incluindo receptores *scavenger* Classe A (SR-A) e proteína de superfície 36 (CD36) em macrófagos, ambos com função na absorção do colesterol. Alguns estudos sugerem também que o TMAO impede o transporte reverso do colesterol, o que pode contribuir para a patogênese da DCV, promovendo acúmulo de colesterol nos macrófagos. ^464^

 Eventos vasculares, como IAM e AVC, observados entre indivíduos com altas concentrações plasmáticas de TMAO, podem estar relacionados com aumento da atividade plaquetária, em virtude da liberação citoplasmática de cálcio, que pode predispor hipercoagulação e aumento de eventos trombóticos. ^465 , 466^

 Metanálise com mais de 26.000 participantes acompanhados por cerca de 4 anos mostrou aumento do risco relativo (7,6%) de mortalidade por todas as causas por cada incremento de TMAO. ^467^

 Estudo recente avaliou a relação do consumo de diferentes fontes de proteína (carne vermelha, branca ou proteína vegetal) no metabolismo de TMAO. O consumo crônico de carnes vermelhas aumentou em mais de 3 vezes as concentrações plasmáticas de TMAO, bem como excreção urinária, em comparação aos demais grupos. ^468^ Estudos com consumo de ovos não observaram associação entre o consumo de ovo e aumento de TMAO. Estudo com 50 participantes saudáveis mostrou que o consumo de 2 ovos (400 mg colina) por dia não alterou as concentrações plasmáticas de TMAO. ^460 , 468^

### 14.2. Esteatose Hepática

 Estudos em animais indicam que dietas ricas em colesterol induzem a progressão da esteato-hepatite não alcoólica, especialmente se associadas a dietas hiperlipídicas e hipercalóricas. ^469 - 472^ No entanto, em seres humanos, não há estudos que evidenciem o efeito do colesterol alimentar sobre desenvolvimento de esteatose hepática. A Diretriz atual para o tratamento da NAFLD não faz referência em relação ao consumo de colesterol e a etiologia ou tratamento dessa doença. ^297^

## 15. Gorduras Interesterificadas

 As gorduras interesterificadas vêm sendo utilizadas em substituição aos ácidos graxos *trans* , os quais são preparados a partir da hidrogenação parcial dos óleos vegetais. As gorduras interesterificadas são praparadas a partir de uma base sólida totalmente hidrogenada que é misturada a um óleo vegetal. Para o preparo dessa base sólida, utiliza-se misturas de frações sólidas, como estearina de palma ou ácido láurico encontrado em óleo de coco com a parte líquida, oleína de palma. ^26^

 A característica principal dessas gorduras é a ausência de ácidos graxos *trans* ; no entanto, apresenta elevada concentração de ácidos graxos SAT. A interesterificação ocorre por meio de processo químico, que utiliza como catalisador o metóxido de sódio, que promove o rearranjo dos ácidos graxos na molécula de glicerol, ^26^ formando triglicérides com novas propriedades físicas, organolépticas e químicas, com enriquecimento de ácidos graxos SAT na posição sn-2 do glicerol, a qual é normalmente ocupada por POLI nos óleos vegetais. ^473^ Neste processo, formam-se grande quantidade de triglicérides constituídos por 3 ácidos graxos saturados. Os ácidos graxos palmítico (este com maior frequência) e esteárico são os mais utilizados na indústria alimentícia em substituição à gordura *trans.* ^473^

### 15.1. Estudos com Animais

 O consumo de dieta normolipídica contendo gordura interesterificada produzida a partir do óleo de soja, comparada com óleo de soja, por ratos Wistar durante 8 semanas demonstrou maior expressão de ATF3 marcador de estresse de retículo e maior concentração da citocina inflamatória TNF-α, sem diferença em relação ao ganho de peso e tolerância à glicose. No entanto, após 16 semanas de tratamento, observou-se maior ganho de peso, acompanhado por maior conteúdo de tecido adiposo retroperitoneal e tolerância à glicose alterada no grupo que consumiu gordura interesterificada. ^474^

 O efeito do óleo de coco e farelo de arroz ou óleo de coco e de gergelim misturados ou submetidos à interesterificação enzimática, com proporção de ácidos graxos SAT: MONO: POLI 1:1:1 e razão POLI/SAT 0,8 a 1 por 60 dias, também foi avaliado em ratos Wistar. ^475^ Os animais que consumiram óleos interesterificados apresentaram concentrações reduzidas de CT, LDLc e TG quando comparados com ratos alimentados com óleos misturados, decorrente de maior expressão hepática do receptor de LDL e da proteína de ligação ao elemento responsivo a esteroides *2* (SREBP2), que induz a síntese de colesterol, em comparação com a mesma gordura sem passar pelo processo de interesterificação. ^476^

 O consumo crônico de dieta hiperlipídica enriquecida com gordura interesterificada rica em ácido palmítico por camundongos com ablação gênica para receptor de LDL demonstrou que o processo de interesterificação não alterou as concentrações plasmáticas de colesterol. No entanto, houve maior concentração de colesterol nas partículas LDL, condição que resultou em maior lesão aterosclerótica, juntamente com maior infiltrado de macrófagos na artéria desses animais. ^477^ Outro trabalho do mesmo grupo demonstrou que o consumo crônico dessas dietas, pelo mesmo modelo animal, levou a maior ganho de peso, expansão de tecido adiposo, hipertrofia de adipócitos com maior inflamação, marcado pela maior presença de pIKK e TNF-α. ^478^

 Outros estudos avaliaram o efeito do consumo de dieta normolipídica contendo gordura interesterificada rica em ácido palmítico pelas fêmeas durante a gestação e lactação sobre a prole. Os resultados apontam que o consumo das gorduras interesterificadas predispõe a prole ao desenvolvimento da obesidade na vida adulta, ^479 , 480^ indicando influência negativa a epigenética. Somado a esses resultados, o estudo de Misan et al. (2015) ^480^ ainda constatou que os filhotes, após 90 dias de vida, apresentaram maior ganho de peso, menor concentração de EPA e maior circulação de leucócitos, ambos no cérebro, sem aumentar TLR4. 

### 15.2. Estudos em Humanos

 Em humanos, tanto o óleo de soja parcialmente hidrogenado quanto o interesterificado proporcionaram aumento na razão LDLc/HDLc quando comparados ao óleo de palma. Além da alteração nas concentrações de lípides plasmáticos, a gordura interesterificada exerceu um efeito adverso sobre o metabolismo da glicose, reduzindo a concentração plasmática de insulina e aumentando a glicemia de jejum. ^481^ No entanto, trabalho mais recente não demonstrou alteração na glicemia e insulina de jejum após consumo da gordura interesterificada. ^482^ Quando, porém, comparado à margarina, contendo elevados teores de ácido linoleico e moderados de *trans* , o consumo de margarinas contendo óleo de palma (láurico, mirístico, palmítico, oleico e linoleico) ou óleo de palma interesterificado favoreceu o aumento nas concentrações de LDLc em homens hipercolesterolêmicos. ^483^ É provável que os diferentes resultados tenham sido obtidos pelo fato de a gordura interesterificada utilizada por Sundram et al. ^481^ ser composta de ácido esteárico, enquanto Filippou et al. ^482^ utilizaram ácido palmítico. Ambos os trabalhos compararam as gorduras interesterificadas com óleo de palma. 

 Além disso, foi demonstrado que a interesterificação transferiu quantidades significativas de ácido palmítico para a posição sn-2 e de ácidos graxos INSAT para as posições sn-1 e sn-3, o que foi refletido nos quilomícrons do plasma. ^484^

 Outro estudo conduzido em mulheres demonstrou, no período pós-prandial, que essas gorduras induziram menor concentração plasmática de triglicérides em mulheres saudáveis menopausadas, ^485^ em adultos jovens saudáveis ^486^ e em adultos hipertrigliceridêmicos, ^487^ em comparação ao óleo de palma. 

 Com relação à influência do estado nutricional dos indivíduos e o consumo dessas gorduras sobre o perfil de lipoproteínas, ^488^ verificou-se que a interesterificação aumentou a concentração pós-prandial de TG (85%) em indivíduos obesos, fato não observado em eutróficos, sugerindo que a interesterificação pode afetar indivíduos saudáveis de maneira diferente daqueles que já têm um risco para desenvolver DCV e DM2. 

 Em indivíduos saudáveis, a interesterificação não alterou as concentrações plasmáticas de lípides, mas favoreceu a menor concentração do dímero D, um produto de degradação da fibrina associado com o risco para doença cardiovascular. ^489^

Até o presente momento, não há evidências científicas que permitam concluir o efeito do processo de interesterificação das gorduras sobre parâmetros metabólicos e de desenvolvimento da aterosclerose e desfecho cardiovascular. Contudo, é importante enfatizar o alto teor de ácidos graxos SAT presentes na gordura interesterificada, utilizada atualmente pela indústria de alimentos.

## 16. Triglicérides de Cadeia Média

 Triglicérides de cadeia média são definidos como lípides estruturados compostos por uma mistura de ácidos graxos de cadeia saturada, contendo de 6 a 12 carbonos, formados por ácido caproico (C6, 1% a 2%), ácido caprílico (C8: 65% a 75%), ácido cáprico (C10: 25% a 35%) e láurico (C12: 1% a 2%). ^367 , 490^ Os ácidos graxos do TCM são obtidos através do fracionamento dos óleos de coco ou de palma. ^491^ Com exceção do láurico, os demais ácidos graxos são absorvidos diretamente por meio da circulação portal e, por não serem incoporados aos quilomícrons, não induzem elevação da concentração plasmática de triglicérides. ^491 , 492^ O ácido láurico é preferencialmente transportado via linfática por meio dos quilomícrons. ^38 , 493^ Por esse motivo, para o tratamento da hiperquilomicronemia familiar, em que se observa ausência de LPL, indica-se o uso de TCM preferencialmente composto por ácido caproico, caprílico e cáprico. ^491^

## 17. Síndrome da Quilomicronemia Familiar

 A SQF é uma doença rara, autossômica recessiva, que afeta 1 a 2 pessoas por milhão. ^494 , 495^ Caracteriza-se por hipertrigliceridemia grave, mesmo em jejum, devido à deficiência da enzima LPL, ou de outras proteínas necessárias para a atividade normal da lipase. As mutações mais comuns na SQF são encontradas nos genes: *LPL, APOA5, GPBIHBP1* (do inglês, *Glycosylphosphatidylinositol Anchored High Density Lipoprotein Binding Protein 1* ), *APOC2, LMF1* ( *Lipase Maturation Factor 1* ), em homozigose, mas também podem ser heterozigotos duplos ou compostos, para mutações em diferentes genes que causam SQF. ^496 - 498^ As concentrações de triglicérides são frequentemente de 10 a 100 vezes aquelas encontradas em indivíduos normais (<150 mg/dL), podendo variar de 1500 a 15000 mg/dL, ou maiores. ^499 , 500^ A hipertrigliceridemia na SQF resulta da incapacidade em metabolizar os TG e outras gorduras. Normalmente, os TG são metabolizados por uma via dependente da LPL. ^500^ Embora exista uma via independente da LPL, esta não é suficiente para compensar a perda da função da LPL. Na SQF, o acúmulo de quilomicrons e seus remanescentes não pode ser metabolizado e se acumula no plasma. No pâncreas ocorre um comprometimento do fluxo sanguíneo, ativação do processo inflamatório, resultando em pancreatites, ^501 - 503^ representando 10% de todas as causas de pancreatite ^501^ . Esses pacientes com pancreatites induzidas por TG elevados apresentam quadros mais graves, internações mais longas, necessidade de permanência em terapia intensiva, altas taxas de evolução para necrose pancreática e, maior frequência de falência de órgãos e mortalidade. ^504^ A pancreatite pode também evoluir para a forma crônica, com insuficiência pancreática exócrina e endócrina, incluindo diabetes pancreático (tipo 3c), que pode ser fatal. Dores abdominais recorrentes, lipemia retinalis, hepatoesplenomegalia, plasma lipêmico, xantomas eruptivos, além de má qualidade de vida são outros achados comuns. ^505 - 510^ Como esses pacientes não são capazes de metabolizar os TG, a orientação nutricional atual consiste de dieta com muito baixo teor de gorduras (de <10-15% das calorias totais, ou cerca de 15-20 g de gorduras por dia), restrição a carboidratos refinados, e retirada de álcool. ^511^ Adicionalmente, indivíduos portadores de FCS, de todas as idades, devem ser regularmente monitorados quanto ao consumo de micronutrientes, particularmente as vitaminas lipossolúveis. ^511^ Dependendo da tolerabilidade individual, os TCM podem ser indicados para aporte calórico da dieta. ^491^ Os medicamentos que sabidamente elevam os TG também devem ser usados com cautela, como diuréticos, beta-bloqueadores, corticosteroides sistêmicos, retinóides, sequestrantes de ácidos biliares, inibidores de protease, anti-depressivos (sertralina). A suplementação com ácidos graxos ω3 e outros medicamentos utilizados para tratamento das hipertrigliceridemias tem resultado inconsistente na redução dos TG. ^512 - 514^

## 18. Aspectos Práticos da Intervenção Nutricional

 A composição nutricional da dieta deve ser ajustada de acordo com os objetivos propostos para cada indivíduo, considerando as necessidades calóricas e preferências individuais e culturais. Diversas estratégias nutricionais são capazes de contribuir para prevenção cardiovascular, tendo como preceito a exclusão de *trans,* adequação do consumo de SAT e, proporcionalmente, maior consumo de gorduras INSAT, além de incentivo ao consumo de frutas, hortaliças e grãos integrais. ^9 , 515^

**Figura 1:**
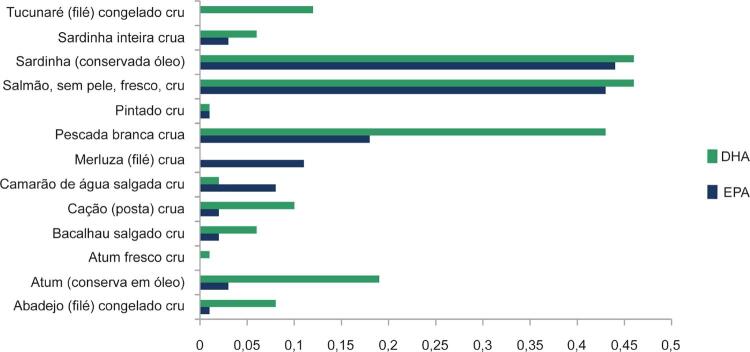
– Conteúdo de EPA e DHA em peixes (g/100 g)

 Alimentos de origem animal – tais como carnes, leite e derivados – contêm naturalmente maior teor de ácidos graxos SAT, enquanto óleos vegetais apresentam elevado teor de INSAT, com exceção do óleo de coco e palma, os quais são ricos em SAT. Entre os óleos vegetais ( Tabela 1 ), destacam-se os óleos de soja, canola e milho, os quais apresentam boa distribuição de ácidos graxos, sendo que o óleo de soja e canola apresenta vantagem adicional ao milho pelo menor teor de ácidos graxos SAT e maior conteúdo de ALA (ω3), o qual é essencial para humanos e é precursor de EPA e DHA, também encontrados em peixes (Figuras 1 e 2 ). 

A quantidade de gorduras das carnes varia em função do tipo de corte, sendo assim, cortes magros, como lombo e filé mignon suíno, apresentam teor de gorduras saturadas similar a cortes bovinos comumente recomendados como patinho e alcatra (Figura 3 ), possibilitando ampliação das opções de consumo de alimentos fonte de proteínas na alimentação com enfoque cardioprotetor.

Produtos lácteos produzidos com leite integral apresentam teor de gorduras saturadas maior que aqueles produzidos com leite desnatado ou parcialmente desnatado. Com relação aos queijos, aqueles com menor teor de água e mais duros, como parmesão, proporcionalmente, apresentam maior concentração de SAT em comparação a requeijão cremoso, queijo minas frescal e ricota (Figura 4 ). A escolha entre o tipo do produto deve considerar o tamanho da porção, uma vez que mesmo produtos lácteos com menor teor de gordura poderão ser importantes fontes de SAT se consumidos em grande quantidade.

 A orientação nutricional deve capacitar o indivíduo quanto ao conhecimento da composição dos alimentos, especialmente os processados, uma vez que um mesmo produto pode apresentar variações na quantidade e tipo de nutrientes, especialmente gorduras, em função de seu fabricante (Tabela S1, Material Suplementar). Nesse contexto, a adequada rotulagem dos alimentos torna-se fundamental para os processos de educação nutricional e liberdade de escolha do consumidor. Outro ponto importante a ser considerado é a forma de preparo dos alimentos, pois preparações fritas podem incorporar grande quantidade de gordura, aumentando consideravelmente o valor calórico. Importante enfatizar que óleos vegetais, fontes de ω3 e ω6, não devem ser substituídos por óleos tropicais, (palma e coco), ou por gorduras de origem animal (banha e manteiga), por serem ricos em SAT e por não fornecerem quantidades adequadas de ácidos graxos essenciais à dieta. Esta orientação está alinhada com a última recomendação da AHA para prevenção do risco cardiovascular ^8 , 9^ e com a Diretriz da ESC/EAS, que recomenda o uso ocasional em pequenas quantidades de óleos tropicais. ^10^

 Por fim, deve-se ter cautela quanto à recomendação do uso de suplementos alimentares que não tenham o seu benefício comprovado cientificamente. Assim, estratégias não farmacológicas para redução do risco cardiovascular devem considerar as evidências disponíveis que apontem para benefício, segurança, custo, tolerabilidade, além de possíveis efeitos de interação fármaco-nutriente. Outro ponto importante é o fato de o uso indevido de suplementos poder comprometer a aderência tanto ao tratamento farmacológico indicado como ao nutricional. ^516^

##  19. Rotulagem e Ácidos Graxos *TRANS*

 O uso da gordura *trans* confere uma série de vantagens à indústria de alimentos, tais como redução de custo, maior tempo de prateleira, alto ponto de fusão e ampla possibilidade de utilização. Entretanto, sua associação com aumento de risco cardiovascular já está claramente estabelecida, de forma que diversas diretrizes internacionais, bem como nacionais, recomendam sua exclusão da dieta. A redução das doenças crônicas não transmissíveis é um dos objetivos da Estratégia Global para Promoção da Alimentação Saudável, Atividade Física e Saúde (Organização Mundial da Saúde [OMS]), ^517^ que, em linha com diretrizes internacionais, ^9 , 10 , 518^ recomenda a exclusão da gordura *trans* da dieta. ^517^

 No Brasil, a Agência Nacional de Vigilância Sanitária (Anvisa), órgão responsável pelo controle da rotulagem de alimentos, estabeleceu, em 2003, que o rótulo dos alimentos deve, obrigatoriamente, declarar a quantidade por porção de gordura *trans* presente no produto. ^519^ Entretanto, apesar da obrigatoriedade, a resolução permite que alimentos que apresentem quantidade de *trans* menor ou igual a 0,2 g por porção possam ser declarados como isentos de *trans* (“zero” ou “não contém”). Importante ressaltar que essa tolerância pode acarretar aumento do consumo de *trans* por meio da ingestão elevada de alimentos declarados como isentos, mas que contêm valores próximos a 0,2 g por porção. ^520^ Além disso, a porção estabelecida no rótulo e enquadrada como isenta de *trans* é, muitas vezes, menor que a quantidade usualmente consumida do produto. ^520^

 Dessa forma, é importante que o consumidor seja orientado para identificar a presença de gordura *trans* na lista de ingredientes, de modo a evitar o consumo dos alimentos nos quais estejam contidos. 

## 20. Considerações Finais

 Este posicionamento evidencia que os achados recentes a respeito da ação dos ácidos graxos em vias de sinalização intracelulares e os resultados dos estudos clínicos e epidemiológicos reiteram as orientações nutricionais vigentes para a prevenção e tratamento de doenças cardiometabólicas. O grau de recomendação e nível de evidência dos ácidos graxos sobre o risco cardiovascular estão demonstrados nas Tabelas 2 e 3. As Diretrizes internacionais preconizam retirada de ácidos graxos *trans* , redução do consumo de ácidos graxos saturados e inclusão, em quantidades adequadas, de alimentos fontes de ácidos graxos insaturados. Estudos epidemiológicos mostram que tanto o excesso do consumo de SAT, como a insuficiência da ingestão de POLI associam-se ao aumento de risco cardiovascular. Além disso, o efeito do consumo dos ácidos graxos depende ainda do perfil alimentar em que se inserem, visto que a substituição de SAT por carboidratos refinados pode aumentar o risco cardiovascular. Por outro lado, quando a substituição isocalórica é feita por insaturados ou mesmo carboidratos complexos, o desfecho CV tende a ser favorável. Os benefícios atribuídos ao perfil adequado de ácidos graxos somente são observados na vigência de padrões alimentares. 

## 21. Quantidade de Ácidos Graxos e Colesterol em Alimentos

**Tabela 1:** – Tabela nutricional com quantidade de ácidos graxos e colesterol em óleos e gorduras. Composição de alimentos por 100 g de parte comestível: ácidos graxos e colesterol

Alimento	Total	Saturados (g/100 g)					Monoinsaturados (g/100 g)		Poli-insaturados (g/100 g)					*Trans* (g/100 g)	Colesterol (mg)
		Total	Láurico 12:0	Mirístico 14:0	Palmítico 16:0	Esteárico 18:0	Total	Oleico 18:1	Total	ALA 18:3	EPA 20:5	DHA 22:6	Linoleico 18:2	Elaídico 18:1t	
Azeite de dendê	100	43,1	0,28	0,79	36,77	4,61	40,1	39,86	16,1	0,83	0	0	15,69	0	NA
Azeite de oliva extravirgem	100	14,9	0	0	11,30	2,96	75,5	74,01	9,5	0,75	0	0	8,74	0	NA
Banha de porco	100	39,2	0,2	1,3	23,8	13,5	45,1	41,2	11,2		0	0	10,2	0	95
*Chantilly spray* com gordura vegetal	27,3	25,9	10,70	3,64	2,63	7,46	0,1	0,05	0,1		0	0	0,08	0	tr.
Maionese industrializada tradicional com ovos	30,5	4,1	0	0,02	2,84	0,37	6,4	6,24	15,4	1,43	0	0	13,86	0	42
Manteiga de cacau	100	59,7	0	0,1	25,5	33,2	32,9	32,6	3	0,1	0	0	2,8	0	0
Manteiga sem sal	86	51,5	2,11	7,96	23,87	9,64	21,9	19,80	1,5	0,27	0	0	1,22	2,31	214
Margarina com óleo interesterificado sem sal (65% de lípides)	67,1	20,9	2,35	0,94	12,41	4,15	14,4	14,07	26,5	2,58	0	0	23,79	0,12	NA
Óleo de abacate	100	11,5	0	0	10,9	0,66	70,5	67,88	13,48	0,95	0	0	12,53	0	0
Óleo de algodão	100	25,9	0	0,8	22,7	2,3	17,8	17,0	51,9	0,2	0	0	51,5	0	0
Óleo de canola	100	7,9	0	0,06	4,59	2,21	62,6	61,14	28,4	6,78	0	0	20,87	0	NA
Óleo de coco	99	82,4	41,8	16,6	8,63	2,5	6,3	6,25	1,7	0,019	0	0	1,67	0,02	0
Óleo de gergelim	100	14,2	0		8,9	4,8	39,7	39,3	41,7	0,3	0	0	41,3	0	0
Óleo de girassol	100	10,8	0	0,07	6,10	3,42	25,4	25,15	62,6	0,39	0	0	62,22	0	NA
Óleo de milho	100	15,2	0		12,12	2,18	33,4	33,04	50,9	0,96	0	0	49,44	0	NA
Óleo de soja	100	15,2	0	0,08	10,83	3,36	23,3	22,98	60,0	5,72	0	0	53,85	0	NA

* Fonte: Núcleo de Estudos e Pesquisas em Alimentação – NEPA/Universidade Estadual de Campinas (UNICAMP). Tabela brasileira de composição de alimentos/NEPA-UNICAMP. Versão II. 2. ed. Campinas, SP: NEPAUNICAMP, 2006. Disponível em: www.unicamp.br/nepa.25 USDA Food Composition Databases. United States Department of Agriculture. Agricultural Research Service USDA National Nutrient Database for Standard Reference Legacy Release, April 2018. USDA Branded Food Products Database. Disponível em: https://ndb.nal.usda.gov/ndb/search/list?home=true. ^*520 *^ ALA: ácido alfalinolênico; DHA: ácido docosahexaenóico; EPA: ácido eicosapentaenoico; NA: não aplicável; tr.: traços. *

**Figura 2:**
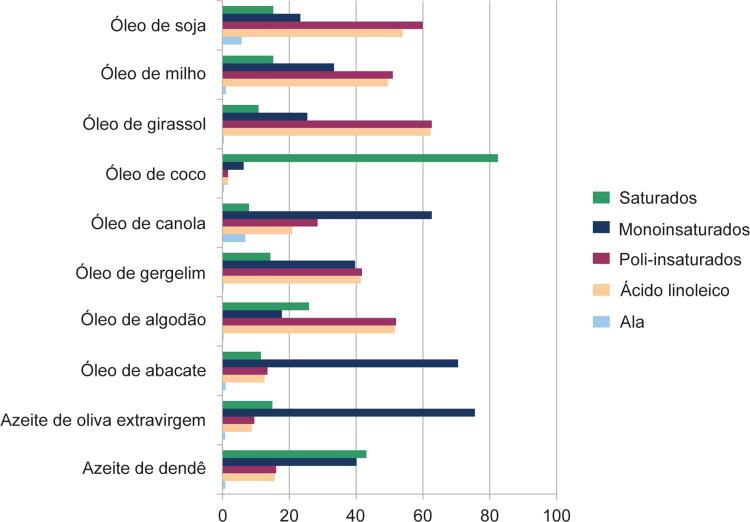
– Conteúdo de ácidos graxos monoinsaturados, poli-insaturados e saturados em óleos vegetais (g/100 g)

**Figura 3:**
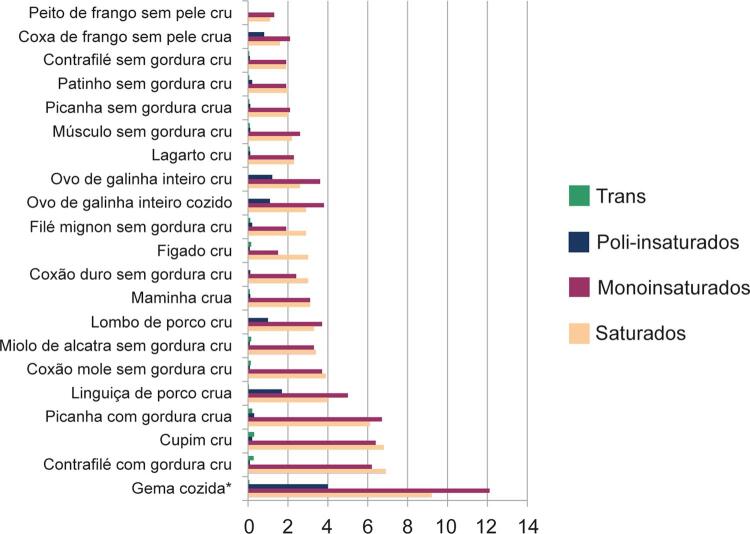
– Conteúdo de ácidos graxos monoinsaturados, poli-insaturados, saturados e trans em carnes e ovos (g/100 g)

**Figura 4:**
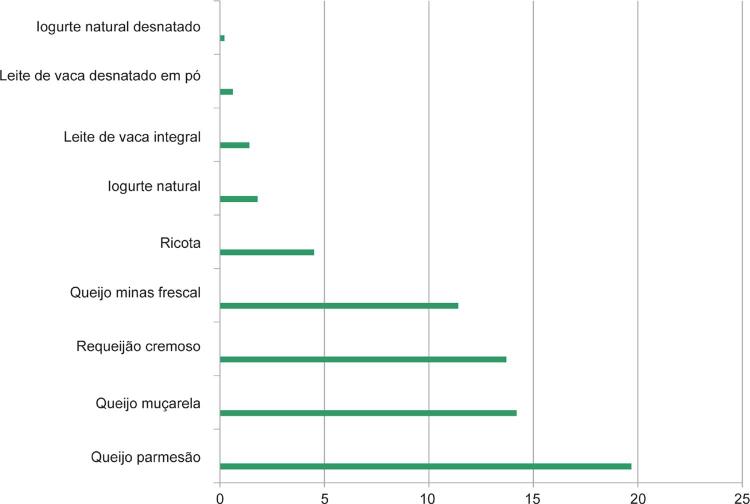
– Conteúdo de ácidos graxos saturados totais em alimentos lácteos (g/100 g)

## 22. Grau de Recomendação e Nível de Evidência: Ácidos Graxos e Risco Cardiovascular

**Tabela 2:** – Ácidos Graxos Alimentares e Risco Cardiovascular

Recomendação	Grau de recomendação	Nível de evidência
Ácidos graxos *trans* devem ser excluídos da dieta	III	A
Limitar o consumo de saturados até 7% do VCT para indivíduos com aumento de risco cardiovascular, como os portadores de hipercolesterolemia familial e Diabetes Mellitus	I	A
Substituir parcialmente ácidos graxos saturados da dieta por poli-insaturados, deve ser recomendado para otimizar a redução das concentrações plasmáticas de LDL-colesterol	I	A
Substituir parcialmente ácidos graxos saturados da dieta por poli-insaturados ômega-6, que podem ser recomendados para melhorar a sensibilidade à insulina	IIa	B
A substituição de ácidos graxos saturados por poli-insaturados pode ser recomendado para reduzir o risco cardiovascular	IIa	A
As recomendações dietéticas devem ser feitas com base no consumo total de poli-insaturados e não apenas com base na relação Ômega-6/Ômega-3	IIa	C
Estimular o consumo de ácidos graxos poli-insaturados Ômega-3 de origem vegetal, como parte de uma dieta saudável, que pode ser recomendado para reduzir o risco cardiovascular	IIb	B
Estimular o consumo de peixe como parte de uma dieta saudável, que deve ser recomendado para diminuir o risco cardiovascular	I	B
Óleos Tropicais (coco e palma) devem ser consumidos apenas ocasionalmente, em razão do alto teor de saturados	III	B

**Tabela 3:** – Suplementação de ômega-3 e Risco Cardiovascular

Suplementação com ômega-3 marinho (2-4 g/dia) pode ser recomendada para hipertrigliceridemia grave (> 500 mg/dL), como parte do tratamento, a critério médico	I	B
Ômega 3 Purificado: Suplementação com formulação a base de EPA (icosapenta-etil, 4 g/dia) em pacientes de alto risco cardiovascular com triglicérides elevados, em uso de estatinas, que pode ser recomendada, pois parece reduzir o risco de eventos isquêmicos, incluindo morte cardiovascular. Formulação não disponível no país	I	A

## *Material suplementar

Para informação adicional, por favor,clique aqui



## References

[1] . Global Burden Disease. (GBD) 2017 Diet Collaborators. Health effects of dietary risks in 195 countries,1990-2017: a systematic analysis for the Global Burden of Disease Study 2017. Lancet. 2019;393(10184):1958-1972. 10.1016/S0140-6736(19)30041-8 PMC6899507 30954305

[2] . World Health Organization (WHO). Global Health Observatory. [Cited in 2019 Dec 12]. Available from: https://www.who.int/gho/ncd/mortality_morbidity/en/

[3] . Brasil. Ministério da Saúde. Vigitel Brasil 2018: vigilância de fatores de risco e proteção para doenças crônicas por inquérito telefônico: estimativas sobre frequência e distribuição sócio demográfica de fatores de risco e proteção para doenças crônicas nas capitais dos 26 estados brasileiros e no Distrito Federal em 2018. Brasília; 2019.

[4] . Page IH, Stareb FJ, Corcoran AC et al. Atherosclerosis and the fat content of the diet. J Am Med Assoc. 1957; 164(18):2048-51. 10.1001/jama.1957.62980180004013 13462729

[5] . The Facts on Fats. 50 years of American Heart Association - Dietary Fats Recomendations. American Heart Association; American Stroke Association. https://www.heart.org/-/media/files/healthy-living/company collaboration/inap/fats-white-paper-ucm_475005.pdf.

[6] . Santos RD, Gagliardi AC, Xavier HT et al; Sociedade Brasileira de Cardiologia. First guidelines on fat consumption and cardiovascular health. Arq Bras Cardiol. 2013;100(1 Suppl 3):1-40. 23598539

[7] . U.S. Department of Health and Human Services and U.S. Department of Agriculture. 2015 – 2020 Dietary Guidelines for Americans. 8th ed Dec, 2015.

[8] . Eckel RH, Jakicic JM, Ard JD et al; American College of Cardiology/American Heart Association Task Force on Practice Guidelines. 2013 AHA/ACC guideline on lifestyle management to reduce cardiovascular risk: a report of the American College of Cardiology/American Heart Association Task Force on Practice Guidelines. Circulation. 2014; 129(25 Suppl 2):S76-99. 10.1161/01.cir.0000437740.48606.d1 24222015

[9] . Grundy SM, Stone NJ, Bailey AL et al. 2018 AHA/ACC/AACVPR/AAPA/ABC/ACPM/ ADA/AGS/APhA/ASPC/NLA/PCNA Guideline on the Management of Blood Cholesterol: A Report of the American College of Cardiology/American Heart Association Task Force on Clinical Practice Guidelines. Circulation. 2019; 139(25):e1082-143 10.1161/CIR.0000000000000625 PMC7403606 30586774

[10] . Mach F, Baigent C, Catapano AL et al; ESC Scientific Document Group. 2019 ESC/EAS Guidelines for the management of dyslipidaemias: lipid modification to reduce cardiovascular risk. Eur Heart J. 2020; 41(1):111-88. 10.1093/eurheartj/ehz455 31504418

[11] . Mensink RP. Effects of saturated fatty acids on serum lipids and lipoproteins: a systematic review and regression analysis. Geneva, Switzerland: World Health Organization; 2016.

[12] . Siri-Tarino PW, Sun Q, Hu FB et al. Saturated fat, carbohydrate, and cardiovascular disease. Am J Clin Nutr. 2010;91(3):502-9. 10.3945/ajcn.2008.26285 PMC2824150 20089734

[13] . Astrup A, Bertram HC, Bonjour JP et al. WHO draft guidelines on dietary saturated and trans fatty acids: time for a new approach? BMJ. 2019 Jul; 366:l4137 10.1136/bmj.l4137 31270106

[14] . Forouhi NG, Koulman A, Sharp SJ et al. Differences in the prospective association between individual plasma phospholipid saturated fatty acids and incident type 2 diabetes: the EPIC-InterAct case-cohort study. Lancet Diabetes Endocrinol. 2014; 2(10):810-8. 10.1016/S2213-8587(14)70146-9 PMC4196248 25107467

[15] . O’Reilly M, Dillon E, Guo W et al. High-density lipoprotein proteomic composition, and not efflux capacity, reflects differential modulation of reverse cholesterol transport by saturated and monounsaturated fat diets. Circulation. 2016; 133(19):1838-50. 10.1161/CIRCULATIONAHA.115.020278 PMC6122580 27081117

[16] . Estruch R, Ros E, Salas-Salvadó J et al. Primary prevention of cardiovascular disease with a Mediterranean diet. N Engl J Med. 2013; 368(14):1279-90 10.1056/NEJMoa1200303 23432189

[17] . Estruch R, Ros E, Salas-Salvadó J et al. Primary prevention of cardiovascular disease with a Mediterranean diet supplemented with extra-virgin olive oil or nuts. N Engl J Med. 2018; 378(25):e34. 10.1056/NEJMoa1800389 29897866

[18] . Estruch R, Ros E, Salas-Salvado J et al. Retraction and republication: primary prevention of cardiovascular disease with a Mediterranean diet. N Engl J Med 2018; 378(25):2441-2. 10.1056/NEJMc1806491 29897867

[19] . Moore TJ, Vollmer WM, Appel LJ et al. Effect of dietary patterns on ambulatory blood pressure: results from the Dietary Approaches to Stop Hypertension (DASH) Trial. DASH Collaborative Research Group. Hypertension. 1999; 34(3):472-77. 10.1161/01.hyp.34.3.472 10489396

[20] . Brasil. Ministério da Saúde. Secretaria de Atenção à Saúde. Departamento de Atenção Básica. Guia alimentar para a população brasileira / Ministério da Saúde, Secretaria de Atenção à Saúde, Departamento de Atenção Básica. 2. ed., 1. reimpr. Brasília: 2014. pp. 156.

[21] . Shan Z, Rehm CD, Rogers G et al. Trends in Dietary Carbohydrate, Protein, and Fat Intake and Diet Quality Among US Adults, 1999-2016. JAMA. 2019; 322(12):1178-87. 10.1001/jama.2019.13771 PMC6763999 31550032

[22] . Pesquisa de orçamentos familiares 2017-2018: primeiros resultados/IBGE, Coordenação de Trabalho e Rendimento. Rio de Janeiro: IBGE, 2019. pp. 69.

[23] . Ricardo CZ, Peroseni IM, Mais LA et al. Trans Fat Labeling Information on Brazilian Packaged Foods. Nutrients. 2019; 11(9). pii: E2130. 10.3390/nu11092130 PMC6770177 31500088

[24] . Kris-Etherton PM. AHA Science Advisory. Monounsaturated fatty acids and risk of cardiovascular disease. American Heart Association. Nutrition Committee. Circulation. 1999; 100(11):1253-8. 10.1161/01.cir.100.11.1253 10484550

[25] . Universidade Estadual de Campinas – UNICAMP. Tabela brasileira de composição de alimentos – TACO. 4. ed. rev. e ampl. Campinas: UNICAMP/NEPA, 2011. pp. 161. Disponível em: http://www.unicamp.br/nepa/taco/tabela.

[26] . Tarrago-Trani MT, Phillips KM, Lemar LE et al. New and existing oils and fats used in products with reduced trans-fatty acids content. J Am Diet Assoc. 2006; 106(6):867-80. 10.1016/j.jada.2006.03.010 16720128

[27] . Krumreich FD, Borges CD, Mendonça CRB et al. Bioactive compounds and quality parameters of avocado oil obtained by different processes. Food Chem. 2018 Aug; 257:376-81. 10.1016/j.foodchem.2018.03.048 29622225

[28] . Almeida JC, Perassolo MS, Camargo JL et al. Fatty acid composition and cholesterol content of beef and chicken meat in Southern Brazil. Rev Bras Cienc Farm. 2006; 42(1):109-17.

[29] . Alfaia CM, Lopes PA, Madeira MS et al. Current feeding strategies to improve pork intramuscular fat content and its nutritional quality. Adv Food Nutr Res. 2019 Apr; 89:53-94. 10.1016/bs.afnr.2019.03.006 31351530

[30] . Lee JH, O’Keefe JH, Lavie CJ et al. Omega-3 fatty acids: Cardiovascular benefits, sources and sustainability. Nat Rev Cardiol. 2009;6(12):753-58. 10.1038/nrcardio.2009.188 19859067

[31] . Bodkowski R, Czyz K, Kupczynski R et al. Lipid complex effect on fatty acid profile and chemical composition of cow milk and cheese. J Dairy Sci. 2016; 99(1):57–67. 10.3168/jds.2015-9321 26506539

[32] . Trumbo P, Schlicker S, Yates AA et al. Food and Nutrition Board of the Institute of Medicine, The National Academies. Dietary reference intakes for energy, carbohydrate, fiber, fat, fatty acids, cholesterol, protein and amino acids. J Am Diet Assoc. 2002; 102(11):1621-30. 10.1016/s0002-8223(02)90346-9 12449285

[33] . Burdge GC, Wootton SA. Conversion of alpha-linolenic acid to eicosapentaenoic, docosapentaenoic and docosahexaenoic acids in young women. Br J Nutr. 2002; 88(4):411-20. 10.1079/BJN2002689 12323090

[34] . Harper CR, Edwards MJ, DeFilippis AP et al. Flaxseed oil increases the plasma concentrations of cardioprotective (n-3) fatty acids in humans. J Nutr. 2006;136(1):83-7. 10.1093/jn/136.1.83 16365063

[35] . Baker EJ, Miles EA, Burdge GC et al. Metabolism and functional effects of plant-derived omega-3 fatty acids in humans. Prog Lipid Res. 2016 Oct; 64:30-56. 10.1016/j.plipres.2016.07.002 27496755

[36] . Berge K, Musa-Veloso K, Harwood M et al. Krill oil supplementation lowers triglycerides without increasing low-density lipoprotein cholesterol in adults with borderline high or high triglyceride levels. Nutr Res. 2014;34(2):126-33. 10.1016/j.nutres.2013.12.003 24461313

[37] . Tvrzicka E, Kremmyda LS, Stankova B et al. Fatty acids as biocompounds: their role in human metabolism, health and disease--a review. Part 1: classification, dietary sources and biological functions. Biomed Pap Med Fac Univ Palacky Olomouc Czech Repub. 2011; 155(22):117-30. 10.5507/bp.2011.038 21804620

[38] . McDonald GB, Saunders DR, Weidman M et al. Portal venous transport of long-chain fatty acids absorbed from rat intestine. Am J Physiol. 1980; 239(3):G141-50. 10.1152/ajpgi.1980.239.3.G141 7435568

[39] . Rioux V, Legrand P. Saturated fatty acids: simple molecular structures with complex cellular functions. Curr Opin Clin Nutr Metab Care 2007; 10(6):752-8. 10.1097/MCO.0b013e3282f01a75 18089958

[40] . Mitchell DA, Vasudevan A, Linder ME et al. Protein palmitoylation by a family of DHHC protein S-acyltransferases. J Lipid Res. 2006; 47(6):1118-27. 10.1194/jlr.R600007-JLR200 16582420

[41] . Calder PC. Functional roles of fatty acids and their effects on human health. JPEN J Parenter Enteral Nutr. 2015; 39(1 Supp):18S-32S. 10.1177/0148607115595980 26177664

[42] . Carta G, Murru E, Banni S, Manca C. Palmitic Acid: Physiological Role, Metabolism and Nutritional Implications. Front Physiol. 2017 Nov 8;8:902. 10.3389/fphys.2017.00902 PMC5682332 29167646

[43] . Orsavova J, Misurcova L, Ambrozova JV et al. Fatty acids composition of vegetable oils and its contribution to dietary energy intake and dependence of cardiovascular mortality on dietary intake of fatty acids. Int J Mol Sci. 2015; 16(6):12871-90. 10.3390/ijms160612871 PMC4490476 26057750

[44] . Wolff RL, Precht D, Nasser B et al. Trans- and cis-octadecenoic acid isomers in the hump and milk lipids from Camelusdromedarius. Lipids. 2001;36(10):1175-8. 10.1007/s11745-001-0829-y 11768163

[45] . Ference BA, Ginsberg HN, Graham I et al. Low-density lipoproteins cause atherosclerotic cardiovascular disease. 1. Evidence from genetic, epidemiologic, and clinical studies. A consensus statement from the European Atherosclerosis Society Consensus Panel. Eur Heart J. 2017; 38(32):2459-72. 10.1093/eurheartj/ehx144 PMC5837225 28444290

[46] . Mente A, Dehghan M, Rangarajan S et al. Prospective Urban Rural Epidemiology (PURE) study investigators. Association of dietary nutrients with blood lipids and blood pressure in 18 countries: a cross-sectional analysis from the PURE study. Lancet Diabetes Endocrinol. 2017; 5(10):774-87. 10.1016/S2213-8587(17)30283-8 28864143

[47] . Foster GD, Wyatt HR, Hill JO et al. A randomized trial of a low-carbohydrate diet for obesity. N Engl J Med. 2003; 348(21):2082-90. 10.1056/NEJMoa022207 12761365

[48] . Grundy SM. Influence of stearic acid on cholesterol metabolism relative to other long-chain fatty acids. Am J Clin Nutr. 1994; 60(6 Suppl):986S-90S. 10.1093/ajcn/60.6.986S 7977157

[49] . Spritz N, Mishkel MA. Effects of dietary fats on plasma lipids and lipoproteins: an hypothesis for the lipid-lowering effect of unsaturated fatty acids. J Clin Invest. 1969; 48(1):78-86. 10.1172/JCI105976 PMC322193 5765029

[50] . Srivastava RA, Ito H, Hess M et al. Regulation of low-density lipoprotein receptor gene expression in HepG2 and Caco2 cells by palmitate, oleate, and 25-hydroxycholesterol. J Lipid Res.1995; 36(7):1434-46. 7595067

[51] . Mustad VA, Ellsworth JL, Cooper AD et al. Dietary linoleic acid increases and palmitic acid decreases hepatic LDL receptor protein and mRNA abundance in young pigs. J Lipid Res. 1996; 37(11):2310-23. 8978483

[52] . Nicolosi RJ, Stucchi AF, Kowala MC et al. Effect of dietary fat saturation and cholesterol on LDL composition and metabolism. In vivo studies of receptor and nonreceptor-mediated catabolism of LDL in cebus monkeys. Arteriosclerosis. 1990; 10(1):119-28 10.1161/01.atv.10.1.119 2297342

[53] . Jackson KG, Maitin V, Leake DS et al. Saturated fat-induced changes in Sf 60-400 particle composition reduces uptake of LDL by HepG2 cells. J Lipid Res. 2006; 47(2):393-403. 10.1194/jlr.M500382-JLR200 16278492

[54] . Lin J, Yang R, Tarr PT et al. Hyperlipidemic effects of dietary saturated fats mediated through PGC-1beta coactivation of SREBP. Cell. 2005; 120(2):261-73. 10.1016/j.cell.2004.11.043 15680331

[55] . Zong G, Li Y, Wanders AJ et al. Intake of individual saturated fatty acids and risk of coronary heart disease in US men and women: two prospective longitudinal cohort studies. BMJ. 2016 Nov; 355:i5796. 10.1136/bmj.i5796 PMC5121105 27881409

[56] . Mensink RP, Zock PL, Kester AD et al. Effects of dietary fatty acids and carbohydrates on the ratio of serum total to HDL cholesterol and on serum lipids and apolipoproteins: a meta-analysis of 60 controlled trials. Am J Clin Nutr 2003; 77(5):1146-55. 10.1093/ajcn/77.5.1146 12716665

[57] . Schwab U, Lauritzen L, Tholstrup T et al. Effect of the amount and type of dietary fat on cardiometabolic risk factors and risk of developing type 2 diabetes, cardiovascular diseases, and cancer: a systematic review. Food Nutr Res. 2014; 10:58. 10.3402/fnr.v58.25145 PMC4095759 25045347

[58] . Li Y, Hruby A, Bernstein AM et al. Saturated fats compared with unsaturated fats and sources of carbohydrates in relation to risk of coronary heart disease: a prospective cohort study. J Am Coll Cardiol. 2015; 66(14):1538-48. 10.1016/j.jacc.2015.07.055 PMC4593072 26429077

[59] . Howard BV, Van Horn L, Hsia J et al. Low-fat dietary pattern and risk of cardiovascular disease: the Women’s Health Initiative Randomized Controlled Dietary Modification Trial. JAMA. 2006; 295(6):655-66. 10.1001/jama.295.6.655 16467234

[60] . Hegsted DM, Ausman LM, Johnson JA et al. Dietary fat and serum lipids: An evaluation of the experimental data. Am J Clin Nutr. 1993;57(6):875-83. 10.1093/ajcn/57.6.875 8503356

[61] . Gardner CD, Kraemer HC. Monounsaturated versus polyunsaturated dietary fat and serum lipids. A meta-analysis. Arterioscler. Arterioscler Thromb Vasc Biol. 1995; 15(11):1917-27. 10.1161/01.atv.15.11.1917 7583572

[62] . Mensink RP, Katan MB. Effect of dietary fatty acids on serum lipids and lipoproteins. A meta-analysis of 27 trials. Arterioscler Thromb. 1992; 12(8):911-9. 10.1161/01.atv.12.8.911 1386252

[63] . Egert S, Kratz M, Kannenberg F et al. Effects of high-fat and low-fat diets rich in monounsaturated fatty acids on serum lipids, LDL size and indices of lipid peroxidation in healthy non-obese men and women when consumed under controlled conditions. Eur J Nutr. 2011; 50(1):71-9. 10.1007/s00394-010-0116-9 20521076

[64] . Gill JM, Brown JC, Caslake MJ et al. Effects of dietary monounsaturated fatty acids on lipoprotein concentrations, compositions, and subfraction distributions and on VLDL apolipoprotein B kinetics: dose-dependent effects on LDL. Am J Clin Nutr. 2003; 78(1):47-56. 10.1093/ajcn/78.1.47 12816770

[65] . Hooper L, Al-Khudairy L, Abdelhamid AS et al. Omega-6 fats for the primary and secondary prevention of cardiovascular disease. Cochrane Database Syst Rev. 2018 Nov;11:CD011094. 10.1002/14651858.CD011094.pub4 PMC6516799 30488422

[66] . Balk EM, Lichtenstein AH, Chung M et al. Effects of omega-3 fatty acids on serum markers of cardiovascular disease risk: a systematic review. Atherosclerosis. 2006; 189(1):19-30. 10.1016/j.atherosclerosis.2006.02.012 16530201

[67] . Wendland E, Farmer A, Glasziou P et al. Effect of alpha linolenic acid on cardiovascular risk markers: a systematic review. Heart. 2006; 92(2):166-9. 10.1136/hrt.2004.053538 PMC1860766 15890766

[68] . Harris WS, Miller M, Tighe AP et al. Omega-3 fatty acids and coronary heart disease risk: clinical and mechanistic perspectives. Atherosclerosis 2008;197(1):12-24 10.1016/j.atherosclerosis.2007.11.008 18160071

[69] . Ishida T, Ohta M, Nakakuki M et al. Distinct regulation of plasma LDL cholesterol by eicosapentaenoic acid and docosahexaenoic acid in high fat diet-fed hamsters: participation of cholesterol ester transfer protein and LDL receptor. Prostaglandins Leukot Essent Fatty Acids. 2013; 88(4):281-8 10.1016/j.plefa.2013.01.001 23375839

[70] . Le Jossic-Corcos C, Gonthier C, Zaghini I et al. Hepatic farnesyl diphosphate synthase expression is suppressed by polyunsaturated fatty acids. Biochem J. 2005; 385(Pt 3):787-94. 10.1042/BJ20040933 PMC1134755 15473864

[71] . Goyens PL, Mensink RP. Effects of alpha-linolenic acid versus those of EPA/DHA on cardiovascular risk markers in healthy elderly subjects. Eur J Clin Nutr. 2006;60(8): 978-84 10.1038/sj.ejcn.1602408 16482073

[72] . Egert S, Somoza V, Kannenberg F et al. Influence of three rapeseed oil-rich diets, fortified with alpha-linolenic acid, eicosapentaenoic acid or docosahexaenoic acid on the composition and oxidizability of low-density lipoproteins: Results of a controlled study in healthy volunteers. Eur J Clin Nutr. 2007; 61(3):314-25. 10.1038/sj.ejcn.1602523 16969378

[73] . Machado RM, Nakandakare ER, Quintao EC et al. Omega-6 polyunsaturated fatty acids prevent atherosclerosis development in LDLr-KO mice, in spite of displaying a pro-inflammatory profile similar to trans fatty acids. Atherosclerosis. 2012; 224(1):66-74. 10.1016/j.atherosclerosis.2012.06.059 22809447

[74] . Matthan NR, Ausman LM, Lichtenstein AH et al. Hydrogenated fat consumption affects cholesterol synthesis in moderately hypercholesterolemic women. J Lipid Res. 2000; 41(5):834-9. 10787444

[75] . Matthan NR, Welty FK, Barrett PH et al. Dietary hydrogenated fat increases high-density lipoprotein apoA-I catabolism and decreases low-density lipoprotein apoB-100 catabolism in hypercholesterolemic women. Arterioscler Thromb Vasc Biol. 2004;24(6):1092-7. 10.1161/01.ATV.0000128410.23161.be 15087307

[76] . Mozaffarian D, Katan MB, Ascherio A et al. Trans fatty acids and cardiovascular disease. N Engl J Med. 2006; 354(15):1601. 10.1056/NEJMra054035 16611951

[77] . Hadj Ahmed S, Kharroubi W, Kaoubaa N et al. Correlation of trans fatty acids with the severity of coronary artery disease lesions. Lipids Health Dis. 2018; 17(1):52. 10.1186/s12944-018-0699-3 PMC5856295 29544473

[78] . Khosla P, Hajri T, Pronczuk A et al. Replacing dietary palmitic acid with elaidic acid (t-C18:1 delta9) depresses HDL and increases CETP activity in cebus monkeys. J Nutr. 1997; 127(3):531S-6S. 10.1093/jn/127.3.531S 9082041

[79] . Mauger JF, Lichtenstein AH, Ausman LM et al. Effect of different forms of dietary hydrogenated fats on LDL particle size. Am J Clin Nutr. 2003; 78(3):370-5. 10.1093/ajcn/78.3.370 12936917

[80] . Mozaffarian D, Clarke R. Quantitative effects on cardiovascular risk factors and coronaryheart disease risk of replacing partially hydrogenated vegetable oils with other fats and oils. Eur J Clin Nutr. 2009; 63(Suppl 2):S22-33. 10.1038/sj.ejcn.1602976 19424216

[81] . Harris WS, Bulchandani D. why do omega-3 fatty acids lower serum triglycerides? Curr Opin Lipidol. 2006; 17(4):387-93. 10.1097/01.mol.0000236363.63840.16 16832161

[82] . Gale SE, Westover EJ, Dudley N et al. Side chain oxygenated cholesterol regulates cellular cholesterol homeostasis through direct sterol-membrane interactions. J Biol Chem. 2009;284(3):1755-64. 10.1074/jbc.M807210200 PMC2615513 18996837

[83] . Hernández-Rodas MC, Valenzuela R, Echeverría F et al. Supplementation with docosahexaenoic acid and extra virgin olive oil prevents liver steatosis induced by a high-fat diet in mice through PPAR-α and Nrf2 upregulation with concomitant SREBP-1c and NF-kB downregulation. Mol Nutr Food Res. 2017;61(12). 10.1002/mnfr.201700479 28940752

[84] . Prasad K. Flaxseed and cardiovascular health. J Cardiovasc Pharmacol. 2009; 54(5):369-77. 10.1097/FJC.0b013e3181af04e5 19568181

[85] . Harris WS. n-3 fatty acids and serum lipoproteins: human studies. Am J Clin Nutr. 1997; 65(5 Suppl):1645S-54S. 10.1093/ajcn/65.5.1645S 9129504

[86] . Hartweg J, Perera R, Montori V et al. Omega-3 polyunsaturated fatty acids (PUFA) for type 2 diabetes mellitus. Cochrane Database Syst Rev. 2008 Jan;(1):CD003205. 10.1002/14651858.CD003205.pub2 PMC9006221 18254017

[87] . Park Y, Harris WS. Omega-3 fatty acid supplementation accelerates chylomicron triglyceride clearance. J. Lipid Res. 2003; 44(3):455-63. 10.1194/jlr.M200282-JLR200 12562865

[88] . Caputo M, Zirpoli H, Torino G et al. Selective regulation of UGT1A1 and SREBP-1c mRNA expression by docosahexaenoic, eicosapentaenoic, and arachidonic acids. J Cell Physiol. 2011;226(1):187-93. 10.1002/jcp.22323 20648548

[89] . Howell G, Deng X, Yellaturu C et al. n-3 polyunsaturated fatty acids suppress insulin-induced SREBP-1c transcription via reduced trans-activating capacity of LXR-alpha. Biochim Biophys Acta. 2009; 1791(12):1190-6. 10.1016/j.bbalip.2009.08.008 PMC2783506 19716432

[90] . Kajikawa S, Harada T, Kawashima A et al. Highly purified eicosapentaenoic acid prevents the progression of hepatic steatosis by repressing monounsaturated fatty acid synthesis in high-fat/high-sucrose diet-fed mice. Prostaglandins Leukot Essent Fatty Acids. 2009; 80(4):229-38. 10.1016/j.plefa.2009.02.004 19328666

[91] . Miller M, Stone NJ, Ballantyne C et al; American Heart Association Clinical Lipidology, Thrombosis, and Prevention Committee of the Council on Nutrition, Physical Activity, and Metabolism, Council on Arteriosclerosis, Thrombosis and Vascular Biology, Council on Cardiovascular N. Triglycerides and cardiovascular disease: a scientific statement from the American Heart Association. Circulation. 2011; 123(20):2292-333. 10.1161/CIR.0b013e3182160726 21502576

[92] . Jacobson TA. Role of n-3 fatty acids in the treatment of hypertriglyceridemia and cardiovascular disease. Am J Clin Nutr. 2008; 87(6):1981S-90S. 10.1093/ajcn/87.6.1981S 18541599

[93] . Mente A, de Koning L, Shannon HS et al. A systematic review of the evidence supporting a causal link between dietary factors and coronary heart disease. Arch Intern Med. 2009; 169(7):659-69. 10.1001/archinternmed.2009.38 19364995

[94] . Mozaffarian D, Micha R, Wallace S. Effects on coronary heart disease of increasing polyunsaturated fat in place of saturated fat: a systematic review and meta-analysis of randomized controlled trials. PLoS Med. 2010; 7(3):e1000252. 10.1371/journal.pmed.1000252 PMC2843598 20351774

[95] . Astrup A, Dyerberg J, Elwood P et al. The role of reducing intakes of saturated fat in the prevention of cardiovascular disease: where does the evidence stand in 2010? Am J Clin Nutr. 2011; 93(4):684-8. 10.3945/ajcn.110.004622 PMC3138219 21270379

[96] . Hooper L, Martin N, Abdelhamid A et al. Reduction in saturated fat intake for cardiovascular disease. Cochrane Database Syst Rev. 2015 Jun; (6):CD011737. 10.1002/14651858.CD011737 26068959

[97] . Farvid MS, Ding M, Pan A et al. Dietary linoleic acid and risk of coronary heart disease: a systematic review and meta-analysis of prospective cohort studies. Circulation. 2014; 130(16):1568-78. 10.1161/CIRCULATIONAHA.114.010236 PMC4334131 25161045

[98] . Chowdhury R, Warnakula S, Kunutsor S et al. Association of dietary, circulating, and supplement fatty acids with coronary risk: a systematic review and meta-analysis. Ann Intern Med. 2014;160(6):398-406. 10.7326/M13-1788 24723079

[99] . Jakobsen MU, O’Reilly EJ, Heitmann BL et al. Major types of dietary fat and risk of coronary heart disease: a pooled analysis of 11 cohort studies. Am J Clin Nutr. 2009;89(5):1425-32. 10.3945/ajcn.2008.27124 PMC2676998 19211817

[100] . Zelman K. The great fat debate: a closer look at the controversy-questioning the validity of age-old dietary guidance. J Am Diet Assoc. 2011; 111(5):655-8. 10.1016/j.jada.2011.03.026 21515106

[101] . Siri-Tarino PW, Sun Q, Hu FB et al. Meta-analysis of prospective cohort studies evaluating the association of saturated fat with cardiovascular disease. Am J Clin Nutr 2010; 91(3):535-46. 10.3945/ajcn.2009.27725 PMC2824152 20071648

[102] . Dehghan M, Mente A, Zhang X et al. Prospective Urban Rural Epidemiology (PURE) study investigators. Associations of fats and carbohydrate intake with cardiovascular disease and mortality in 18 countries from five continents (PURE): a prospective cohort study. Lancet. 2017; 390(10107):2050-62. 10.1016/S0140-6736(17)32252-3 28864332

[103] . Dehghan M, Mente A, Rangarajan S et al; Prospective Urban Rural Epidemiology (PURE) study investigators. Association of dairy intake with cardiovascular disease and mortality in 21 countries from five continents (PURE): a prospective cohort study. Lancet. 2018; 392(10161):2288-97. 10.1016/S0140-6736(18)31812-9 30217460

[104] . Hjermann I, Velve Byre K, Holme I et al. Effect of diet and smoking intervention on the incidence of coronary heart disease. Report from the Oslo Study Group of a randomised trial in healthy men. Lancet. 1981; 2(8259):1303. 10.1016/s0140-6736(81)91338-6 6118715

[105] . Anderson CA, Appel LJ. Dietary modification and CVD prevention: a matter of fat. JAMA. 2006; 295(6):693. 10.1001/jama.295.6.693 16467240

[106] . Praagman J, Beulens JW, Alssema M et al. The association between dietary saturated fatty acids and ischemic heart disease depends on the type and source of fatty acid in the European Prospective Investigation into Cancer and Nutrition-Netherlands cohort. Am J Clin Nutr 2016; 103(2):356-65. 10.3945/ajcn.115.122671 26791181

[107] . Praagman J, de Jonge EA, Kiefte-de Jong JC et al. Dietary saturated fatty acids and coronary heart disease risk in a Dutch middle-aged and elderly population. Arterioscler Thromb Vasc Biol 2016; 36(9):2011-8. 10.1161/ATVBAHA.116.307578 27417581

[108] . Khaw KT, Friesen MD, Riboli E et al. Plasma phospholipid fatty acid concentration and incident coronary heart disease in men and women: the EPIC-Norfolk prospective study. PLoS Med. 2012; 9(7):e1001255. 10.1371/journal.pmed.1001255 PMC3389034 22802735

[109] . Imamura F, Sharp SJ, Koulman A et al. A combination of plasma phospholipid fatty acids and its association with incidence of type 2 diabetes: The EPIC-InterAct case-cohort study. PLoS Med. 2017; 14(10):e1002409. 10.1371/journal.pmed.1002409 PMC5636062 29020051

[110] . de Oliveira Otto MC, Nettleton JA, Lemaitre RN et al. Biomarkers of dairy fatty acids and risk of cardiovascular disease in the Multi-ethnic Study of Atherosclerosis. J Am Heart Assoc. 2013; 2(4):e000092. 10.1161/JAHA.113.000092 PMC3828802 23868191

[111] . Reedy J, Krebs-Smith SM, Miller PE et al. Higher diet quality is associated with decreased risk of allcause, cardiovascular disease, and cancer mortality among older adults. J Nutr. 2014; 144(6):881-9. 10.3945/jn.113.189407 PMC4018951 24572039

[112] . Casas R, Urpi-Sardà M, Sacanella E et al. anti-inflammatory effects of the Mediterranean diet in the early and late stages of atheroma plaque development. Mediators Inflamm. 2017 Apr; 2017:3674390. 10.1155/2017/3674390 PMC5412172 28484308

[113] . Joris PJ, Mensink RP. Role of cis-monounsaturated fatty acids in the prevention of coronary heart disease. Curr Atheroscler Rep. 2016; 18(7):38. 10.1007/s11883-016-0597-y PMC4879159 27221500

[114] . Harris WS, Poston WC, Haddock CK. Tissue n-3 and n-6 fatty acids and risk for coronary heart disease events. Atherosclerosis. 2007; 193(1):1-10. 10.1016/j.atherosclerosis.2007.03.018 17507020

[115] . Miettinen M, Turpeinen O, Karvonen MJ et al. Dietary prevention of coronary heart disease in women: the Finnish mental hospital study. Int J Epidemiol. 1983;12(1):17-25. 10.1093/ije/12.1.17 6840954

[116] . Frantz ID Jr, Dawson EA, Ashman PL et al. Test of effect of lipid lowering by diet on cardiovascular risk: the Minnesota Coronary Survey. Arteriosclerosis. 1989; 9(1):129-35. 10.1161/01.atv.9.1.129 2643423

[117] . Kris-Etherton P, Fleming J, Harris WS. The debate about n-6 polyunsaturated fatty acid recommendations for cardiovascular health. J Am Diet Assoc. 2010; 110(2):201-4. 10.1016/j.jada.2009.12.006 20102846

[118] . Lloyd-Williams F, O’Flaherty M, Mwatsama M et al. Estimating the cardiovascular mortality burden attributable to the European Common Agricultural Policy on dietary saturated fats. Bull World Health Organ. 2008; 86(7):535-41A. 10.2471/BLT.08.053728 PMC2647494 18670665

[119] . Ramsden CE, Hibbeln JR, Majchrzak SF et al. n-6 fatty acid-specific and mixed polyunsaturate dietary interventions have different effects on CHD risk: a meta-analysis of randomised controlled trials. Br J Nutr. 2010; 104(11):1586-600. 10.1017/S0007114510004010 PMC9422343 21118617

[120] . Al-Khudairy L, Hartley L, Clar C et al. Omega 6 fatty acids for the primary prevention of cardiovascular disease. Cochrane Database Syst Rev. 2015 Nov; (11):CD011094. 10.1002/14651858.CD011094.pub2 26571451

[121] . Marklund M, Wu JHY, Imamura F et al; Cohorts for Heart and Aging Research in Genomic Epidemiology (CHARGE) Fatty Acids and Outcomes Research Consortium (FORCE). Biomarkers of dietary omega-6 fatty acids and incident cardiovascular disease and mortality. Circulation. 2019; 139(21):2422-36. 10.1161/CIRCULATIONAHA.118.038908 PMC6582360 30971107

[122] . Bosch Egert S, Stehle P. Impact of n-3 fatty acids on endothelial function: results from human interventions studies. Curr Opin Clin Nutr Metab Care. 2011; 14(2):121-31. 10.1097/MCO.0b013e3283439622 21252652

[123] . Flock MR, Skulas-Ray AC, Harris WS et al. Effects of supplemental long-chain omega-3 fatty acids and erythrocyte membrane fatty acid content on circulating inflammatory markers in a randomized controlled trial of healthy adults. Prostaglandins Leukot Essent Fatty Acids. 2014; 91(4):161-8. 10.1016/j.plefa.2014.07.006 PMC4156902 25091379

[124] . Ito MK. Long-chain omega-3 fatty acids, fibrates and niacin as therapeutic options in the treatment of hypertriglyceridemia: a review of the literature. Atherosclerosis. 2015; 242(2):647-56. 10.1016/j.atherosclerosis.2015.06.012 26296750

[125] . Burr ML, Fehily AM, Gilbert JF et al. Effects of changes in fat, fish, and fibre intakes on death and myocardial reinfarction: diet and reinfarction trial (DART). Lancet. 1989; 2(8666):757-61. 10.1016/s0140-6736(89)90828-3 2571009

[126] . GISSI-Prevenzione Investigators. Dietary supplementation with n-3 polyunsaturated fatty acids and vitamin E after myocardial infarction: results of the GISSI-Prevenzione trial. Gruppo Italiano per lo Studio della Sopravvivenza nell’Infarto miocardico. Lancet. 1999; 354(9177):447-55. 10465168

[127] . Yokoyama M, Origasa H, Matsuzaki M et al.; Japan EPA lipid intervention study (JELIS) Investigators. Effects of eicosapentaenoic acid on major coronary events in hypercholesterolaemic patients (JELIS): a randomised open-label, blinded endpoint analysis. Lancet. 2007; 369(9567):1090-8. 10.1016/S0140-6736(07)60527-3 17398308

[128] . Kromhout D, Giltay EJ, Geleijnse JM. Alpha Omega Trial Group. n-3 fatty acids and cardiovascular events after myocardial infarction. N Engl J Med. 2010; 363(21):2015-26. 10.1056/NEJMoa1003603 20929341

[129] . Rauch B, Schiele R, Schneider S et al.; OMEGA Study Group. OMEGA, a randomized, placebo-controlled trial to test the effect of highly purified omega-3 fatty acids on top of modern guideline-adjusted therapy after myocardial infarction. Circulation. 2010; 122(21):2152-9. 10.1161/CIRCULATIONAHA.110.948562 21060071

[130] . Galan P, Kesse-Guyot E, Czernichow S et al. SU.FOL.OM3 Collaborative Group. Effects of B vitamins and omega 3 fatty acids on cardiovascular diseases: a randomised placebo controlled trial. BMJ. 2010 Nov; 341:c6273. 10.1136/bmj.c6273 PMC2993045 21115589

[131] . Alexander DD, Miller PE, Van Elswyk ME et al. A meta-analysis of randomized controlled trials and prospective cohort studies of eicosapentaenoic and docosahexaenoic long-chain omega-3 fatty acids and coronary heart disease risk. Mayo Clin Proc. 2017; 92(1):15-29. 10.1016/j.mayocp.2016.10.018 28062061

[132] . ASCEND Study Collaborative Group, Bowman L, Mafham M, Wallendszus K, Stevens W et al. Effects of n-3 fatty acid supplements in diabetes mellitus. N Engl J Med. 2018; 379(16):1540-50. 10.1056/NEJMoa1804989 30146932

[133] . Abdelhamid AS, Brown TJ, Brainard JS, Biswas P, Thorpe GC, Moore HJ, et al. Omega-3 fatty acids for the primary and secondary prevention of cardiovascular disease. Cochrane Database of Syst Rev. 2018 Jul; (11):CD003177. 10.1002/14651858.CD003177.pub4 PMC6517311 30521670

[134] . Bhatt DL, Steg PG, Miller M et al; REDUCE-IT Investigators. Cardiovascular risk reduction with icosapent ethyl for hypertriglyceridemia. N Engl J Med. 2019; 380(1):11-22. 10.1056/NEJMoa1812792 30415628

[135] . Wang HH, Hung TM, Wei J et al. Fish oil increases antioxidant enzyme activities in macrophages and reduces atherosclerotic lesions in apoE-knockout mice. Cardiovasc Res. 2004;61(1):169-76. 10.1016/j.cardiores.2003.11.002 14732214

[136] . Saraswathi V, Gao L, Morrow JD et al. Fish oil increases cholesterol storage in white adipose tissue with concomitant decreases in inflammation, hepatic steatosis, and atherosclerosis in mice. J Nutr. 2007; 137(7):1776-82. 10.1093/jn/137.7.1776 17585030

[137] . Zampolli A, Bysted A, Leth T et al. Contrasting effect of fish oil supplementation on the development of atherosclerosis in murine models. Atherosclerosis. 2006;184(1):78-85. 10.1016/j.atherosclerosis.2005.04.018 15946668

[138] . Casós K, Sáiz MP, Ruiz-Sanz JI et al. Atherosclerosis prevention by a fish oil-rich diet in apoE(-/-) mice is associated with a reduction of endothelial adhesion molecules. Atherosclerosis. 2008; 201(2):306-17. 10.1016/j.atherosclerosis.2008.02.033 18439610

[139] . Matsumoto M, Sata M, Fukuda D et al. Orally administered eicosapentaenoic acid reduces and stabilizes atherosclerotic lesions in ApoE-deficient mice. Atherosclerosis. 2008; 197(2):524-33. 10.1016/j.atherosclerosis.2007.07.023 17765904

[140] . Xu Z, Riediger N, Innis S et al. Fish oil significantly alters fatty acid profiles in various lipid fractions but not atherogenesis in apo E-KO mice. Eur J Nutr. 2007; 46(2):103-10. 10.1007/s00394-006-0638-3 17225919

[141] . Sekikawa A, Curb JD, Ueshima H et al.; ERA JUMP (Electron-beam tomography, risk factor assessment among japanese and u.s. men in the post-world war ii birth cohort) Study Group. Marine-derived n-3 fatty acids and atherosclerosis in Japanese, Japanese- American, and white men: a cross-sectional study. J Am Coll Cardiol. 2008; 52(6):417-24. 10.1016/j.jacc.2008.03.047 PMC2736602 18672160

[142] . Heine-Bröring RC, Brouwer IA, Proença RV et al. Intake of fish and marine n-3 fatty acids in relation to coronary calcification: the Rotterdam Study. Am J Clin Nutr. 2010; 91(5):1317-23. 10.3945/ajcn.2009.28416 20219958

[143] . He K, Liu K, Daviglus ML et al. Intakes of long-chain n-3 polyunsaturated fatty acids and fish in relation to measurements of subclinical atherosclerosis. Am J Clin Nutr. 2008; 88(4):1111-8. 10.1093/ajcn/88.4.1111 PMC4151325 18842801

[144] . von Schacky C, Angerer P, Kothny W et al. The effect of dietary omega-3 fatty acids on coronary atherosclerosis: a randomized, double-blind, placebo-controlled trial. Ann Intern Med. 1999; 130(7):554-62. 10.7326/0003-4819-130-7-199904060-00003 10189324

[145] . Angerer P, Kothny W, Störk S et al. Effect of dietary supplementation with omega-3 fatty acids on progression of atherosclerosis in carotid arteries. Cardiovasc Res. 2002;54(1):183-90 10.1016/s0008-6363(02)00229-8 12062374

[146] . Mita T, Watada H, Ogihara T et al. Eicosapentaenoic acid reduces the progression of carotid intima-media thickness in patients with type 2 diabetes. Atherosclerosis. 2007; 191(1):162-7. 10.1016/j.atherosclerosis.2006.03.005 16616147

[147] . Thies F, Garry JM, Yaqoob P et al. Association of n-3 polyunsaturated fatty acids with stability of atherosclerotic plaques: a randomised controlled trial. Lancet. 2003; 361(9356):477-85. 10.1016/S0140-6736(03)12468-3 12583947

[148] . Leng GC, Lee AJ, Fowkes FG et al. Randomized controlled trial of gamma-linolenic acid and eicosapentaenoic acid in peripheral arterial disease. Clin Nutr. 1998; 17(6):265-71. 10.1016/s0261-5614(98)80318-x 10205349

[149] . Carrero JJ, Lopez-Huertas E, Salmeron LM et al. Daily supplementation with (n-3) PUFAs, oleic acid, folic acid, and vitamins B-6 and E increases pain-free walking distance and improves risk factors in men with peripheral vascular disease. J Nutr. 2005; 135(6):1393-99. 10.1093/jn/135.6.1393 15930443

[150] . Carrero JJ, López-Huertas E, Salmerón LM et al. Simvastatin and supplementation with ω-3 polyunsaturated fatty acids and vitamins improves claudication distance in a randomized PILOT study in patients with peripheral vascular disease. Nutr Res. 2006; 26(12):637-43.

[151] . Gans RO, Bilo HJ, Weersink EG et al. Fish oil supplementation in patients with stable claudication. Am J Surg. 1990;160(5):490-5. 10.1016/s0002-9610(05)81012-8 2240382

[152] . Ishikawa Y, Yokoyama M, Saito Y et al. JELIS Investigators. Preventive effects of eicosapentaenoic acid on coronary artery disease in patients with peripheral artery disease. Circ J. 2010; 74(7):1451-7 10.1253/circj.cj-09-0520 20484828

[153] . Enns JE, Yeganeh A, Zarychanski R et al. The impact of omega-3 polyunsaturated fatty acid supplementation on the incidence of cardiovascular events and complications in peripheral arterial disease: a systematic review and meta-analysis. BMC Cardiovasc Disord. 2014 May; 14:70. 10.1186/1471-2261-14-70 PMC4065588 24885361

[154] . Saravanan P, Davidson NC, Schmidt EB et al. Cardiovascular effects of marine omega-3 fatty acids. Lancet. 2010; 376(9740):540-50. 10.1016/S0140-6736(10)60445-X 20638121

[155] . Marchioli R, Barzi F, Bomba E et al. GISSI-Prevenzione Investigators. Early protection against sudden death by n-3 polyunsaturated fatty acids after myocardial infarction: time-course analysis of the results of the Gruppo Italiano per lo Studio della Sopravvivenza nell’Infarto Miocardico (GISSI)-Prevenzione. Circulation. 2002; 105(16):1897-903. 10.1161/01.cir.0000014682.14181.f2 11997274

[156] . Leaf A, Albert CM, Josephson M et al. Fatty Acid Antiarrhythmia Trial Investigators. Prevention of fatal arrhythmias in high-risk subjects by fish oil n-3 fatty acid intake. Circulation. 2005; 112(18):2762-8. 10.1161/CIRCULATIONAHA.105.549527 16267249

[157] . Raitt MH, Connor WE, Morris C et al. Fish oil supplementation and risk of ventricular tachycardia and ventricular fibrillation in patients with implantable defibrillators: a randomized controlled trial. JAMA. 2005; 293(23):2884-91. 10.1001/jama.293.23.2884 15956633

[158] . Khoueiry G, Rafeh NA, Sullivan E et al. Do omega-3 polyunsaturated fatty acids reduce risk of sudden cardiac death and ventricular arrhythmias? A meta-analysis of randomized trials. Heart Lung. 2013; 42(4):251-6. 10.1016/j.hrtlng.2013.03.006 23714269

[159] . Albert CM. Omega-3 fatty acids, ventricular arrhythmias, and sudden cardiac death: antiarrhythmic, proarrhythmic, or neither. Circ Arrhythm Electrophysiol. 2012; 5(3):456-9. 10.1161/CIRCEP.112.971416 PMC3399517 22715236

[160] . Gissi-HF Investigators, Tavazzi L, Maggioni AP et al. Effect of n-3 polyunsaturated fatty acids in patients with chronic heart failure (the GISSI-HF trial): a randomised, double-blind, placebo-controlled trial. Lancet. 2008; 372(9645):1223-30. 10.1016/S0140-6736(08)61239-8 18757090

[161] . Mozaffarian D, Bryson CL, Lemaitre RN et al. Fish intake and risk of incident heart failure. J Am Coll Cardiol. 2005; 45(12):2015-21. 10.1016/j.jacc.2005.03.038 15963403

[162] . Yamagishi K, Iso H, Date C et al. Japan Collaborative Cohort Study for Evaluation of Cancer Risk Study Group. Fish, omega-3 polyunsaturated fatty acids, and mortality from cardiovascular diseases in a nationwide community-based cohort of Japanese men and women the JACC (Japan Collaborative Cohort Study for Evaluation of Cancer Risk) Study. J Am Coll Cardiol. 2008; 52(12):988-96. 10.1016/j.jacc.2008.06.018 18786479

[163] . Mozaffarian D. Does alpha-linolenic acid intake reduce the risk of coronary heart disease? A review of the evidence. Altern Ther Health Med. 2005; 11(3):24-30. 15945135

[164] . Mozaffarian D, Ascherio A, Hu FB et al. Interplay between different polyunsaturated fatty acids and risk of coronary heart disease in men. Circulation. 2005; 111(2):157-164. 10.1161/01.CIR.0000152099.87287.83 PMC1201401 15630029

[165] . Albert CM, Oh K, Whang W et al. Dietary alpha-linolenic acid intake and risk of sudden cardiac death and coronary heart disease. Circulation. 2005; 112(21):3232-8. 10.1161/CIRCULATIONAHA.105.572008 16301356

[166] . Wang C, Harris WS, Chung M et al. n-3 Fatty acids from fish or fish-oil supplements, but not alpha-linolenic acid, benefit cardiovascular disease outcomes in primary- and secondary- prevention studies: a systematic review. Am J Clin Nutr. 2006; 84(1):5-17. 10.1093/ajcn/84.1.5 16825676

[167] . Brouwer IA, Katan MB, Zock PL. Dietary alpha-linolenic acid is associated with reduced risk of fatal coronary heart disease, but increased prostate cancer risk: a meta-analysis. J Nutr. 2004; 134(4):919-22. 10.1093/jn/134.4.919 15051847

[168] . Simopoulos AP. The importance of the ratio of omega-6/ omega-3 essential fatty acids. Biomed Pharmacother. 2002; 56(8):365-79. 10.1016/s0753-3322(02)00253-6 12442909

[169] . Simopoulos AP. Evolutionary aspects of diet, the omega-6/ omega-3 ratio and genetic variation: nutritional implications for chronic diseases. Biomed Pharmacother. 2006; 60(9):502-7. 10.1016/j.biopha.2006.07.080 17045449

[170] . Gómez Candela C, Bermejo López LM, Loria Kohen V. Importance of a balanced omega 6/omega 3 ratio for the maintenance of health: nutritional recommendations. Nutr Hosp. 2011; 26(2):323-9. 10.1590/S0212-16112011000200013 21666970

[171] . de Lorgeril M, Renaud S, Mamelle N et al. Mediterranean alpha-linolenic acid-rich diet in secondary prevention of coronary heart disease. Lancet. 1994; 343(8911):1454-9. 10.1016/s0140-6736(94)92580-1 7911176

[172] . Harris WS. The omega-6/omega-3 ratio and cardiovascular disease risk: uses and abuses. Curr Atheroscler Rep. 2006; 8(6):453-9. 10.1007/s11883-006-0019-7 17045070

[173] . Griffin BA. How relevant is the ratio of dietary n-6 to n-3 polyunsaturated fatty acids to cardiovascular disease risk? Evidence from the OPTILIP study. Curr Opin Lipidol. 2008; 19(1):57-62 10.1097/MOL.0b013e3282f2e2a8 18196988

[174] . Liou YA, King DJ, Zibrik D et al. Decreasing linoleic acid with constant alpha-linolenic acid in dietary fats increases (n-3) eicosapentaenoic acid in plasma phospholipids in healthy men. J Nutr. 2007; 137(4):945-52. 10.1093/jn/137.4.945 17374659

[175] . Hu FB, Stampfer MJ, Manson JE et al. Dietary fat intake and the risk of coronary heart disease in women. N Engl J Med. 1997; 337(21):1491. 10.1056/NEJM199711203372102 9366580

[176] . Willett WC, Stampfer MJ, Manson JE et al. Intake of trans fatty acids and risk of coronary heart disease among women. Lancet. 1993; 341(8845):581. 10.1016/0140-6736(93)90350-p 8094827

[177] . Gillman MW, Cupples LA, Gagnon D et al. Margarine intake and subsequent coronary heart disease in men. Epidemiology. 1997; 8(2):144-9. 10.1097/00001648-199703000-00004 9229205

[178] . Oomen CM, Ocké MC, Feskens EJ et al. Association between trans fatty acid intake and 10-year risk of coronary heart disease in the Zutphen Elderly Study: a prospective population-based study. Lancet. 2001; 357(9258):746-51. 10.1016/s0140-6736(00)04166-0 11253967

[179] . Guasch-Ferré M, Babio N, Martínez-González MA et al. Dietary fat intake and risk of cardiovascular disease and all-cause mortality in a population at high risk of cardiovascular disease. Am J Clin Nutr. 2015; 102(6):1563-73. 10.3945/ajcn.115.116046 26561617

[180] . Wang DD, Li Y, Chiuve SE et al. Association of Specific Dietary Fats With Total and Cause-Specific Mortality. JAMA Intern Med. 2016; 176(8):1134-45. 10.1001/jamainternmed.2016.2417 PMC5123772 27379574

[181] . Menotti A, Kromhout D, Blackburn H et al. Food intake patterns and 25-year mortality from coronary heart disease: cross cultural correlations in the Seven Countries Study. The Seven Countries Study Research Group. Eur J Epidemiol. 1999; 15(6):507-15. 10.1023/a:1007529206050 10485342

[182] . Wang Q, Imamura F, Lemaitre RN et al. Plasma phospholipid trans-fatty acids levels, cardiovascular diseases, and total mortality: the cardiovascular health study. J Am Heart Assoc. 2014; 3(4). pii: e000914. 10.1161/JAHA.114.000914 PMC4310377 25164946

[183] . Fournier N, Attia N, Rousseau-Ralliard D et al. Deleterious impact of elaidic fatty acid on ABCA1-mediated cholesterol efflux from mouse and human macrophages. Biochim Biophys Acta. 2012; 1821(2):303-12. 10.1016/j.bbalip.2011.10.005 22074701

[184] . Godo S, Shimokawa H. Endothelial functions. Arterioscler Thromb Vasc Biol. 2017; 37(9):e108-14. 10.1161/ATVBAHA.117.309813 28835487

[185] . Ghosh A, Gao L, Thakur A et al. Role of free fatty acids in endothelial dysfunction. J Biomed Sci. 2017;24(1):50. 10.1186/s12929-017-0357-5 PMC5530532 28750629

[186] . Mundi S, Massaro M, Scoditti E et al. Endothelial permeability, LDL deposition, and cardiovascular risk factors-a review. Cardiovasc Res. 2018; 114(1):35-52. 10.1093/cvr/cvx226 PMC7729208 29228169

[187] . Geovanini GR, Libby P. Atherosclerosis and inflammation: overview and updates. Clin Sci. 2018; 132(12):1243-52. 10.1042/CS20180306 29930142

[188] . Gori T. Endothelial Function: A Short Guide for the Interventional Cardiologist. Int J Mol Sci. 2018; 19(12). pii: E3838. 10.3390/ijms19123838 PMC6320818 30513819

[189] . Hadi HA, Carr CS, Al Suwaidi J. Endothelial dysfunction: cardiovascular risk factors, therapy, and outcome. Vasc Health Risk Manag. 2005;1(3):183-98. PMC1993955 17319104

[190] . Nakamura K, Miyoshi T, Yunoki K et al. Postprandial hyperlipidemia as a potential residual risk factor. J Cardiol. 2016; 67(4):335-9. 10.1016/j.jjcc.2015.12.001 26744235

[191] . Newens KJ, Thompson AK, Jackson KG et al. Endothelial function and insulin sensitivity during acute non-esterified fatty acid elevation: Effects of fat composition and gender. Nutr Metab Cardiovasc Dis. 2015; 25(6):575-81. 10.1016/j.numecd.2015.03.004 PMC4456421 25921849

[192] . Oishi JC, Castro CA, Silva KA et al. Endothelial dysfunction and inflammation precedes elevations in blood pressure induced by a high-fat diet. Arq Bras Cardiol. 2018; 110(6):558-67. 10.5935/abc.20180086 PMC6023639 30226915

[193] . Ishiyama J, Taguchi R, Akasaka Y et al. Unsaturated FAs prevent palmitate-induced LOX-1 induction via inhibition of ER stress in macrophages. J Lipid Res. 2011; 52(2):299-307. 10.1194/jlr.M007104 PMC3023550 21078775

[194] . Lee CH, Lee SD, Ou HC et al. Eicosapentaenoic acid protects against palmitic acid-induced endothelial dysfunction via activation of the AMPK/eNOS pathway. Int J Mol Sci. 2014; 15(6):10334-49. 10.3390/ijms150610334 PMC4100154 24918290

[195] . Wen H, Gris D, Lei Y et al. Fatty acid-induced NLRP3-ASC inflammasome activation interferes with insulin signaling. Nat Immunol. 2011; 12(5):408-15. 10.1038/ni.2022 PMC4090391 21478880

[196] . Wang XL, Zhang L, Youker K et al. Free fatty acids inhibit insulin signaling-stimulated endothelial nitric oxide synthase activation through upregulating PTEN or inhibiting Akt kinase. Diabetes. 2006; 55(8):2301-10. 10.2337/db05-1574 16873694

[197] . Kim F, Tysseling KA, Rice J et al. Free fatty acid impairment of nitric oxide production in endothelial cells is mediated by IKKbeta. Arterioscler Thromb Vasc Biol. 2005; 25(5):989-94. 10.1161/01.ATV.0000160549.60980.a8 15731493

[198] . Sokolova M, Vinge LE, Alfsnes K et al. Palmitate promotes inflammatory responses and cellular senescence in cardiac fibroblasts. Biochim Biophys Acta. 2017; 1862(2):234-45. 10.1016/j.bbalip.2016.11.003 27845246

[199] . Zhang Y, Xia G, Zhang Y et al. Palmitate induces VSMC apoptosis via toll like receptor (TLR)4/ROS/p53 pathway. Atherosclerosis. 2017 Aug; 263:74-81. 10.1016/j.atherosclerosis.2017.06.002 28609685

[200] . Lambert EA, Phillips S, Belski R et al. Endothelial function in healthy young individuals is associated with dietary consumption of saturated fat. Front Physiol. 2017 Nov; 8:876. 10.3389/fphys.2017.00876 PMC5684178 29170641

[201] . Vafeiadou K, Weech M, Altowaijri H et al. Replacement of saturated with unsaturated fats had no impact on vascular function but beneficial effects on lipid biomarkers, E-selectin, and blood pressure: results from the randomized, controlled Dietary Intervention and VAScular function (DIVAS) study. Am J Clin Nutr. 2015; 102(1):40-8. 10.3945/ajcn.114.097089 26016869

[202] Rathnayake KM, Weech M, Jackson KG et al. Meal fatty acids have differential effects on postprandial blood pressure and biomarkers of endothelial function but not vascular reactivity in postmenopausal women in the randomized controlled dietary intervention and vascular function (DIVAS)-2 Study. J Nutr. 2018; 148(3):348-57. 10.1093/jn/nxx042 29546297

[203] . Nestel P, Shige H, Pomeroy S et al. The n-3 fatty acids eicosapentaenoic acid and docosahexaenoic acid increase systemic arterial compliance in humans. Am J Clin Nutr. 2002; 76(2):326-30. 10.1093/ajcn/76.2.326 12145002

[204] . Tomiyama H, Takazawa K, Osa S et al. Do eicosapentaenoic acid supplements attenuate age-related in- creases in arterial stiffness in patients with dyslipidemia? A preliminary study. Hypertens Res. 2005; 28(8):651-5. 10.1291/hypres.28.651 16392769

[205] . Zapolska-Downar D, Kosmider A, Naruszewicz M. Trans fatty acids induce apoptosis in human endothelial cells. J Physiol Pharmacol 2005; 56(6):611-25. 16391418

[206] . Bryk D, Zapolska-Downar D, Malecki M et al. Trans fatty acids induce a proinflammatory response in endothelial cells through ROS-dependent nuclear factor-KB activation. J Physiol Pharmacol. 2011; 62(2):229-38. 21673371

[207] . Baer DJ, Judd JT, Clevidence BA et al. Dietary fatty acids affect plasma markers of inflammation in healthy men fed controlled diets: a randomized crossover study. Am J Clin Nutr. 2004;79(6):969-73. 10.1093/ajcn/79.6.969 15159225

[208] . Lopez-Garcia E, Schulze MB, Meigs JB et al. Consumption of trans fatty acids is related to plasma biomarkers of inflammation and endothelial dysfunction. J Nutr. 2005; 135(3):562-6. 10.1093/jn/135.3.562 15735094

[209] . Iwata NG, Pham M, Rizzo NO et al. Trans fatty acids induce vascular inflammation and reduce vascular nitric oxide production in endothelial cells. PLoS One. 2011; 6(12):e29600. 10.1371/journal.pone.0029600 PMC3247279 22216328

[210] . Hirata Y, Takahashi M, Kudoh Y et al. Trans-Fatty acids promote proinflammatory signaling and cell death by stimulating the apoptosis signal-regulating kinase 1 (ASK1)-p38 pathway. J Biol Chem. 2017; 292(12):8174-85. 10.1074/jbc.M116.771519 PMC5437226 28360100

[211] . Bao DQ, Mori T, Burke V et al. Effects of dietary fish and weight reduction on ambulatory blood pressure in over- weight hypertensives. Hypertension. 1998; 32(4):710-7. 10.1161/01.hyp.32.4.710 9774368

[212] . Morris MC, Sacks F, Rosner B. Does fish oil lower blood pressure? A meta-analysis of controlled trials. Circulation. 1993; 88(2):523-33. 10.1161/01.cir.88.2.523 8339414

[213] . Geleijnse JM, Giltay EJ, Grobbee DE et al. Blood pressure response to fish oil supplementation: metaregression analysis of randomized trials. J Hypertens. 2002; 20(8):1493-9. 10.1097/00004872-200208000-00010 12172309

[214] . Vernaglione L, Cristofano C, Chimienti S. Omega-3 polyunsaturated fatty acids and proxies of cardiovascular disease in hemodialysis: a prospective cohort study. J Nephrol. 2008; 21(1):99-105. 18264942

[215] . Hartweg J, Farmer AJ, Holman RR et al. Meta-analysis of the effects of n-3 polyunsaturated fatty acids on haematological and thrombogenic factors in type 2 diabetes. Diabetologia. 2007;50(2):250-8. 10.1007/s00125-006-0486-y 17119918

[216] . Theobald HE, Goodall AH, Sattar N et al. Low-dose docosahexaenoic acid lowers diastolic blood pressure in middle-aged men and women. J Nutr. 2007; 137(4):973-8. 10.1093/jn/137.4.973 17374663

[217] . Hall WL, Sanders KA, Sanders TA et al. A high-fat meal enriched with eicosapentaenoic acid reduces postprandial arterial stiffness measured by digital volume pulse analysis in healthy men. J Nutr. 2008; 138(2):287-91. 10.1093/jn/138.2.287 18203893

[218] . Schwingshackl L, Strasser B, Hoffmann G. Effects of monounsaturated fatty acids on cardiovascular risk factors: a systematic review and meta-analysis. Ann Nutr Metab. 2011; 59(2-4):176-86. 10.1159/000334071 22142965

[219] . Maki KC, Hasse W, Dicklin MR et al. Corn oil lowers plasma cholesterol compared with coconut oil in adults with above-desirable levels of cholesterol in a randomized crossover trial. J Nutr. 2018; 148(10):1556-63. 10.1093/jn/nxy156 PMC6168703 30204921

[220] . Storniolo CE, Casillas R, Bulló M et al. A Mediterranean diet supplemented with extra virgin olive oil or nuts improves endothelial markers involved in blood pressure control in hypertensive women. Eur J Nutr. 2017; 56(1):89-97. 10.1007/s00394-015-1060-5 26450601

[221] . Carnevale R, Pignatelli P, Nocella C et al. Extra virgin olive oil blunt post-prandial oxidative stress via nox2 down-regulation. Atherosclerosis. 2014; 235(2):649-58. 10.1016/j.atherosclerosis.2014.05.954 24980290

[222] . Rallidis LS, Kolomvotsou A, Lekakis J et al. Short-term effects of Mediterranean-type diet intervention on soluble cellular adhesion molecules in subjects with abdominal obesity. Clin Nutr ESPEN. 2017 Feb; 17:38-43. 10.1016/j.clnesp.2016.11.002 28361746

[223] . de Souza RJ, Mente A, Maroleanu A et al. Intake of saturated and trans unsaturated fatty acids and risk of all cause mortality, cardiovascular disease, and type 2 diabetes: Systematic review and meta-analysis of observational studies. BMJ. 2015 Aug; 351:h3978. 10.1136/bmj.h3978 PMC4532752 26268692

[224] . Jeppesen J, Hein HO, Suadicani P et al. Triglyceride concentration and ischemic heart disease: an eight-year follow-up in the Copenhagen male study. Circulation. 1998; 97(11):1029-36. 10.1161/01.cir.97.11.1029 9531248

[225] . Yamagishi K, Iso H, Yatsuya H et al; JACC Study Group. Dietary intake of saturated fatty acids and mortality from cardiovascular disease in Japanese: the Japan Collaborative Cohort Study for Evaluation of Cancer Risk (JACC) Study. Am J Clin Nutr. 2010; 92(4):759-65. 10.3945/ajcn.2009.29146 20685950

[226] . Cheng P, Wang J, Shao W et al. Can dietary saturated fat be beneficial in prevention of stroke risk? A meta-analysis. Neurol Sci. 2016; 37(7):1089-98. 10.1007/s10072-016-2548-3 26979840

[227] . Mozaffarian D, Wu JHY. Omega-3 fatty acids and cardiovascular disease: effects on risk factors, molecular pathways, and clinical events. J Am Coll Cardiol. 2011; 58(20):2047-67. 10.1016/j.jacc.2011.06.063 22051327

[228] . Nobili V, Bedogni G, Alisi A et al. Docosahexaenoic acid supplementation decreases liver fat content in children with non-alcoholic fatty liver disease: double-blind randomised controlled clinical trial. Arch Dis Child. 2011; 96(4):350-3. 10.1136/adc.2010.192401 21233083

[229] . Yaemsiri S, Sen S, Tinker LF et al. Serum fatty acids and incidence of ischemic stroke among postmenopausal women. Stroke. 2013; 44(10):2710-7. 10.1161/STROKEAHA.111.000834 PMC3892376 23899914

[230] . Rizos EC, Ntzani EE, Bika E et al. Association between omega-3 fatty acid supplementation and risk of major cardiovascular disease events: a systematic review and meta-analysis. JAMA. 2012; 308(10):1024-33. 10.1001/2012.jama.11374 22968891

[231] . Iso H, Sato S, Umemura U et al. Linoleic acid, other fatty acids, and the risk of stroke. Stroke. 2002; 33(8):2086-93. 10.1161/01.str.0000023890.25066.50 12154268

[232] . Mozaffarian D, Lemaitre RN, King IB et al. Circulating long- chain ω-3 fatty acids and incidence of congestive heart failure in older adults: the cardiovascular health study: a cohort study. Ann Intern Med. 2011; 155(3):160-70. 10.1059/0003-4819-155-3-201108020-00006 PMC3371768 21810709

[233] . Saber H, Yakoob MY, Shi P et al. Omega-3 fatty acids and incident ischemic stroke and its atherothrombotic and cardioembolic subtypes in 3 US cohorts. Stroke. 2017; 48(10):2678-85. 10.1161/STROKEAHA.117.018235 PMC5769157 28830976

[234] . Banoub JH, El Aneed A, Cohen AM et al. Structural investigation of bacterial lipopolysaccharides by mass spectrometry and tandem mass spectrometry. Mass Spectrom Rev. 2010; 29(4):606-50. 10.1002/mas.20258 20589944

[235] . Hwang DH, Kim JA, Lee JY. Mechanisms for the activation of Toll-like receptor 2/4 by saturated fatty acids and inhibition by docosahexaenoic acid. Eur J Pharmacol. 2016 Aug; 785:24-35. 10.1016/j.ejphar.2016.04.024 PMC5815395 27085899

[236] . Huang S, Rutkowsky JM, Snodgrass RG et al. Saturated fatty acids activate TLR-mediated proinflammatory signaling pathways. J Lipid Res. 2012; 53(9):2002-13. 10.1194/jlr.D029546 PMC3413240 22766885

[237] . Reynolds CM, McGillicuddy FC, Harford KA et al. Dietary saturated fatty acids prime the NLRP3 inflammasome via TLR4 in dendritic cells-implications for diet-induced insulin resistance. Mol Nutr Food Res. 2012; 56(8):1212-22. 10.1002/mnfr.201200058 22700321

[238] . Wang Y, Tao J, Yao Y. Prostaglandin E2 activates NLRP3 inflammasome in endothelial cells to promote diabetic retinopathy. Horm Metab Res. 2018; 50(9):704-710. 10.1055/a-0664-0699 30142638

[239] . Satoh M, Tabuchi T, Itoh T et al. NLRP3 inflammasome activation in coronary artery disease: results from prospective and randomized study of treatment with atorvastatin or rosuvastatin. Clin Sci (Lond). 2014; 126(3):233-41. 10.1042/CS20130043 23944632

[240] . Suzuki M, Takaishi S, Nagasaki M et al. Medium-chain fatty acid-sensing receptor, GPR84, is a proinflammatory receptor. J Biol Chem. 2013; 288(15):10684-91 10.1074/jbc.M112.420042 PMC3624448 23449982

[241] . Lee JY, Sohn KH, Rhee SH et al. Saturated fatty acids, but not unsaturated fatty acids, induce the expression of cyclooxygenase-2 mediated through Toll-like receptor 4. J Biol Chem. 2001; 276(20):16683-9. 10.1074/jbc.M011695200 11278967

[242] . Lee JY, Zhao L, Youn HS et al. Saturated fatty acid activates but polyunsaturated fatty acid inhibits Toll-like receptor 2 dimerized with Toll-like receptor 6 or 1. J Biol Chem. 2004; 279(17):16971-9. 10.1074/jbc.M312990200 14966134

[243] . Caricilli AM, Nascimento PH, Pauli JR et al. Inhibition of toll-like receptor 2 expression improves insulin sensitivity and signalling in muscle and white adipose tissue of mice fed a high-fat diet. J Endocrinol. 2008; 199(3):399-406. 10.1677/JOE-08-0354 18787058

[244] . Perreault M, Roke K, Badawi A et al. Plasma levels of 14:0, 16:0, 16:1n-7, and 20:3n-6 are positively associated, but 18:0 and 18:2n-6 are inversely associated with markers of inflammation in young healthy adults. Lipids. 2014; 49(3):255-63. 10.1007/s11745-013-3874-3 24338596

[245] . de Roos B, Mavrommatis Y, Brouwer IA. Long-chain n-3 polyunsaturated fatty acids: new insights into mechanisms relating to inflammation and coronary heart disease. Br J Pharmacol. 2009; 158(2):413-28. 10.1111/j.1476-5381.2009.00189.x PMC2757681 19422375

[246] . Lopez-Garcia E, Schulze MB, Manson JE et al. Consumption of (n-3) fatty acids is related to plasma biomarkers of inflammation and endothelial activation in women. J Nutr. 2004;134(7):1806-11. 10.1093/jn/134.7.1806 15226473

[247] . Niu K, Hozawa A, Kuriyama S et al. Dietary long-chain n-3 fatty acids of marine origin and serum C-reactive protein concentrations are associated in a population with a diet rich in marine products. Am J Clin Nutr. 2006; 84(1):223-9. 10.1093/ajcn/84.1.223 16825699

[248] . Micallef MA, Munro IA, Garg ML. An inverse relationship between plasma n-3 fatty acids and C-reactive protein in healthy individuals. Eur J Clin Nutr. 2009; 63(9):1154-6. 10.1038/ejcn.2009.20 19352379

[249] . Farzaneh-Far R, Harris WS, Garg S et al. Inverse association of erythrocyte n-3 fatty acid levels with inflammatory biomarkers in patients with stable coronary artery disease: the Heart and Soul Study. Atherosclerosis. 2009; 205(2):538-43. 10.1016/j.atherosclerosis.2008.12.013 PMC2717185 19185299

[250] . Madsen T, Skou HA, Hansen VE et al. C-reactive protein, dietary n-3 fatty acids, and the extent of coronary artery disease. Am J Cardiol. 2001; 88(10):1139-42. 10.1016/s0002-9149(01)02049-5 11703959

[251] . Kelley DS, Siegel D, Fedor DM et al. DHA supplementation decreases serum C-reactive protein and other markers of inflammation in hypertriglyceridemic men. J Nutr. 2009; 139(3):495-501. 10.3945/jn.108.100354 PMC2646223 19158225

[252] . Murphy KJ, Meyer BJ, Mori TA et al. Impact of foods enriched with n-3 long-chain polyunsaturated fatty acids on erythrocyte n-3 levels and cardiovascular risk factors. Br J Nutr. 2007; 97(4):749-57. 10.1017/S000711450747252X 17349088

[253] . Rizza S, Tesauro M, Cardillo C et al. Fish oil supplementation improves endothelial function in normoglycemic offspring of patients with type 2 diabetes. Atherosclerosis. 2009; 206(2):569-74. 10.1016/j.atherosclerosis.2009.03.006 PMC2772138 19394939

[254] . Petersson H, Risérus U, McMonagle J et al. Effects of dietary fat modification on oxidative stress and inflammatory markers in the LIPGENE study. Br J Nutr. 2010; 104(9):1357-62. 10.1017/S000711451000228X 20569506

[255] . Madsen T, Christensen JH, Schmidt EB. C-reactive protein and n-3 fatty acids in patients with a previous myocardial infarction: a placebo-controlled randomized study. Eur J Nutr. 2007; 46(7):428-30. 10.1007/s00394-007-0673-8 17676423

[256] . Madsen T, Christensen JH, Blom M et al. The effect of dietary n-3 fatty acids on serum concentrations of C-reactive protein: a dose-response study. Br J Nutr. 2003; 89(4):517-22. 10.1079/BJN2002815 12654170

[257] . Yoneyama S, Miura K, Sasaki S et al. Dietary intake of fatty acids and serum C-reactive protein in Japanese. J Epidemiol. 2007; 17(3):86-92. 10.2188/jea.17.86 PMC7058455 17545695

[258] . Poudel-Tandukar K, Nanri A, Matsushita Y et al. Dietary intakes of alpha-linolenic and linoleic acids are inversely associated with serum C-reactive protein levels among Japanese men. Nutr Res. 2009; 29(6):363-370. 10.1016/j.nutres.2009.05.012 19628101

[259] . Rallidis LS, Paschos G, Liakos GK et al. Dietary alpha-linolenic acid decreases C-reactive protein, serum amyloid A and interleukin-6 in dyslipidaemic patients. Atherosclerosis. 2003; 167(2):237-242 10.1016/s0021-9150(02)00427-6 12818406

[260] . Mozaffarian D, Rimm EB, King IB et al. Trans fatty acids and systemic inflammation in heart failure. AmJ Clin Nutr. 2004; 80(6):1521-5. 10.1093/ajcn/80.6.1521 PMC1201402 15585763

[261] . Holland WL, Bikman BT, Wang LP et al. Lipid-induced insulin resistance mediated by the proinflammatory receptor TLR4 requires saturated fatty acid-induced ceramide biosynthesis in mice. J Clin Invest. 2011; 121(5):1858-70. 10.1172/JCI43378 PMC3083776 21490391

[262] . Patel PS, Buras ED, Balasubramanyam A. The role of the immune system in obesity and insulin resistance. J Obes. 2013; 2013:616193. 10.1155/2013/616193 PMC3618935 23577240

[263] . Vessby B, Uusitupa M, Hermansen K et al. Substituting dietary saturated for monounsaturated fat impairs insulin sensitivity in healthy men and women: the KANWU Study. Diabetologia. 2001; 44(3):312-9. 10.1007/s001250051620 11317662

[264] . Tierney AC1, McMonagle J, Shaw DI et al. Effects of dietary fat modification on insulin sensitivity and on other risk factors of the metabolic syndrome – LIPGENE: a European randomized dietary intervention study. Int J Obes (Lond). 2011; 35(6):800-9. 10.1038/ijo.2010.209 20938439

[265] . Jebb SA, Lovegrove JA, Griffin BA et al; RISCK Study Group. Effect of changing the amount and type of fat and carbohydrate on insulin sensitivity and cardiovascular risk: the RISCK (Reading, Imperial, Surrey, Cambridge, and Kings) trial. Am J Clin Nutr. 2010; 92(4):748–758. 10.3945/ajcn.2009.29096 PMC3594881 20739418

[266] . Koska J, Ozias MK, Deer J et al. A human model of dietary saturated fatty acid induced insulin resistance. Metabolism. 2016; 65(11):1621-1628. 10.1016/j.metabol.2016.07.015 27733250

[267] . Feskens EJ, Virtanen SM, Räsänen L et al. Dietary factors determining diabetes and impaired glucose tolerance. A 20-year follow-up of the Finnish and Dutch cohorts of the Seven Countries Study. Diabetes Care. 1995; 18(8):1104-12. 10.2337/diacare.18.8.1104 7587845

[268] . Mayer EJ1, Newman B, Quesenberry CP Jr et al. Usual dietary fat intake and insulin concentrations in healthy women twins. Diabetes Care. 1993; 16(11):1459-1469. 10.2337/diacare.16.11.1459 8299435

[269] . van Dam RM, Willett WC, Rimm EB et al. Dietary fat and meat intake in relation to risk of type 2 diabetes in men. Diabetes Care. 2002; 25(3):417-424. 10.2337/diacare.25.3.417 11874924

[270] . Meyer KA, Kushi LH, Jacobs DR Jr et al. Dietary fat and incidence of type 2 diabetes in older Iowa women. Diabetes Care. 2001; 24(9):1528-35 10.2337/diacare.24.9.1528 11522694

[271] . Salmerón J, Hu FB, Manson JE et al. Dietary fat intake and risk of type 2 diabetes in women. Am J Clin Nutr. 2001; 73(6):1019-1026. 10.1093/ajcn/73.6.1019 11382654

[272] . Tinker LF1, Bonds DE, Margolis KL et al; Women’s Health Initiative. Low-fat dietary pattern and risk of treated diabetes mellitus in postmenopausal women: the Women’s Health Initiative randomized controlled dietary modification trial. Arch Intern Med. 2008; 168(14):1500-1511. 10.1001/archinte.168.14.1500 18663162

[273] . Li SX, Imamura F, Schulze MB et al. Interplay between genetic predisposition, macronutrient intake and type 2 diabetes incidence: analysis within EPIC-InterAct across eight European countries. Diabetologia. 2018; 61(6):1325-1332. 10.1007/s00125-018-4586-2 PMC6445347 29549418

[274] . Alhazmi A, Stojanovski E, McEvoy M et al. Diet quality score is a predictor of type 2 diabetes risk in women: the Australian Longitudinal Study on Women’s Health. Br J Nutr. 2014; 112(6):945-51. 10.1017/S0007114514001688 25201303

[275] . Kaushik M, Mozaffarian D, Spiegelman D et al. Long-chain omega-3 fatty acids, fish intake, and the risk of type 2 diabetes mellitus. Am J Clin Nutr. 2009; 90(3):613-20. 10.3945/ajcn.2008.27424 PMC2728645 19625683

[276] . Wu JHY, Micha R, Imamura F et al. Omega-3 fatty acids and incident type 2 diabetes: a systematic review and meta-analysis. Br J Nutr. 2012; 107(Suppl 2):S214-27. 10.1017/S0007114512001602 PMC3744862 22591895

[277] . Djoussé L, Biggs ML, Lemaitre RN et al. Plasma omega-3 fatty acids and incident diabetes in older adults. Am J Clin Nutr. 2011; 94(2):527-33. 10.3945/ajcn.111.013334 PMC3142727 21593500

[278] . Friedberg CE, Janssen MJ, Heine RJ et al. Fish oil and glycemic control in diabetes: a meta-analysis. Diabetes Care. 1998; 21(4):494-500. 10.2337/diacare.21.4.494 9571330

[279] . ORIGIN Trial Investigators, Bosch J, Gerstein HC, Dagenais GR et al. n-3 fatty acids and cardiovascular outcomes in patients with dysglycemia. N Engl J Med. 2012; 367(4):309-18. 10.1056/NEJMoa1203859 22686415

[280] . Brostow DP, Odegaard AO, Koh WP et al. Omega-3 fatty acids and incident type 2 diabetes: the Singapore Chinese Health Study. Am J Clin Nutr. 2011; 94(2):520-6. 10.3945/ajcn.110.009357 PMC3142726 21593505

[281] . Bloedon LT, Balikai S, Chittams J et al. Flaxseed and cardiovascular risk factors: results from a double blind, randomized, controlled clinical trial. J Am Coll Nutr. 2008; 27(1):65-74. 10.1080/07315724.2008.10719676 18460483

[282] . Barre DE. The role of consumption of alpha-linolenic, eicosapentaenoic and docosahexaenoic acids in human metabolic syndrome and type 2 diabetes: a mini-review. J Oleo Sci. 2007; 56(7):319-25. 10.5650/jos.56.319 17898498

[283] . Lichtenstein AH, Schwab US. Relationship of dietary fat to glucose metabolism. Atherosclerosis. 2000; 150(2):227-43. 10.1016/s0021-9150(99)00504-3 10856515

[284] . Heine RJ, Mulder C, Popp-Snijders C et al. Linoleic-acid-enriched diet: long-term effects on serum lipoprotein and apolipoprotein concentrations and insulin sensitivity in noninsulin- dependent diabetic patients. Am J Clin Nutr. 1989; 49(3):448-56. 10.1093/ajcn/49.3.448 2923077

[285] . Risérus U, Willett WC, Hu FB. Dietary fats and prevention of type 2 diabetes. Prog Lipid Res. 2009; 48(1):44-51. 10.1016/j.plipres.2008.10.002 PMC2654180 19032965

[286] . Zhao X, Shen C, Zhu H et al. Trans-fatty acids aggravate obesity, insulin resistance and hepatic steatosis in C57BL/6 mice, possibly by suppressing the IRS1 dependent pathway. Molecules. 2016; 21(6):1-11. 10.3390/molecules21060705 PMC6273562 27248994

[287] . Dorfman SE, Laurent D, Gounarides JS et al. Metabolic implications of dietary trans-fatty acids. Obesity. 2009; 17(6):1200-7. 10.1038/oby.2008.662 19584878

[288] . Thompson AK, Minihane AM, Williams CM. Trans fatty acids, insulin resistance and diabetes. Eur J Clin Nutr. 2011; 65(5):553-64. 10.1038/ejcn.2010.240 20978530

[289] . Longhi R. Effect of a trans fatty acid-enriched diet on biochemical and inflammatory parameters in Wistar rats. Eur J Nutr. 2017; 56(4):1003-16. 10.1007/s00394-015-1148-y 26754301

[290] . Zhang Z, Gillespie C, Yang Q. Plasma trans-fatty acid concentrations continue to be associated with metabolic syndrome among US adults after reductions in trans-fatty acid intake. Nutr Res. 2017 Jul; 43:51-9. 10.1016/j.nutres.2017.05.008 PMC10084882 28739054

[291] . Christiansen E, Schnider S, Palmvig B et al. Intake of a diet high in trans monounsaturated fatty acids or saturated fatty acids: Effects on postprandial insulinemia and glycemia in obese patients with NIDDM. Diabetes Care. 1997; 20(5):881-7. 10.2337/diacare.20.5.881 9135961

[292] . Wang Q, Imamura F, Ma W et al. Circulating and dietary trans fatty acids and incident type 2 diabetes in older adults: The cardiovascular health study. Diabetes Care. 2015; 38(6):1099-107. 10.2337/dc14-2101 PMC4439533 25784660

[293] . Leclercq IA, Horsmans Y. Nonalcoholic fatty liver disease: the potential role of nutritional management. Curr Opin Clin Nutr Metab Care. 2008; 11(6):766-73. 10.1097/MCO.0b013e328312c353 18827582

[294] . Benedict M, Zhang X. Non-alcoholic fatty liver disease: An expanded review. World J Hepatol. 2017; 9(16):715-32. 10.4254/wjh.v9.i16.715 PMC5468341 28652891

[295] . Serfaty L, Lemoine M. Definition and natural history of metabolic steatosis: clinical aspects of NAFLD, NASH and cirrhosis. Diabetes Metab. 2008; 34(6 Pt 2):634-7. 10.1016/S1262-3636(08)74597-X 19195623

[296] . Haas JT, Francque S, Staels B. Pathophysiology and mechanisms of nonalcoholic fatty liver disease. Annu Rev Physiol. 2016 Nov; 78:181-205. 10.1146/annurev-physiol-021115-105331 26667070

[297] . Chalasani N, Younossi Z, Lavine JE et al. The diagnosis and management of nonalcoholic fatty liver disease: Practice guidance from the American Association for the Study of Liver Diseases. Hepatology. 2018; 67(1):328-57. 10.1002/hep.29367 28714183

[298] . Targher G, Arcaro G. Non-alcoholic fatty liver disease and increased risk of cardiovascular disease. Atherosclerosis. 2007; 191(2):235-40. 10.1016/j.atherosclerosis.2006.08.021 16970951

[299] . Zivkovic AM, German JB, Sanyal AJ. Comparative review of diets for the metabolic syndrome: implications for nonalcoholic fatty liver disease. Am J Clin Nutr. 2007; 86(2):285-300. 10.1093/ajcn/86.2.285 17684197

[300] . Donnelly KL, Smith CI, Schwarzenberg SJ et al. Sources of fatty acids stored in liver and secreted via lipoproteins in patients with nonalcoholic fatty liver disease. J Clin Invest. 2005;115(5):1343-51. 10.1172/JCI23621 PMC1087172 15864352

[301] . Fabbrini E, Mohammed BS, Magkos F et al. Alterations in adipose tissue and hepatic lipid kinetics in obese men and women with nonalcoholic fatty liver disease. Gastroenterology. 2008; 134(2):424-31. 10.1053/j.gastro.2007.11.038 PMC2705923 18242210

[302] . Postic C, Girard J. The role of the lipogenic pathway in the development of hepatic steatosis. Diabetes Metab. 2008; 34(6 Pt 2):643-8. 10.1016/S1262-3636(08)74599-3 19195625

[303] . Wei Y, Rector RS, Thyfault JP et al. Nonalcoholic fatty liver disease and mitochondrial dysfunction. World J Gastroenterol. 2008; 14(2):193-9. 10.3748/wjg.14.193 PMC2675113 18186554

[304] . Duvnjak M, Lerotić I, Barsić N et al. Pathogenesis and management issues for non-alcoholic fatty liver disease. World J Gastroenterol. 2007;13(34):4539-50. 10.3748/wjg.v13.i34.4539 PMC4611824 17729403

[305] . Lottenberg AM, Afonso Mda S, Lavrador MS et al. The role of dietary fatty acids in the pathology of metabolic syndrome. J Nutr Biochem. 2012; 23(9):1027-40. 10.1016/j.jnutbio.2012.03.004 22749135

[306] . Westerbacka J, Kolak M, Kiviluoto T, et al. Genes involved in fatty acid partitioning and binding, lipolysis, monocyte/macrophage recruitment, and inflammation are overexpressed in the human fatty liver of insulin-resistant subjects. Diabetes. 2007;56(11):2759-2765. 10.2337/db07-0156 17704301

[307] . Pettinelli P, Videla LA. Up-regulation of PPAR-gamma mRNA expression in the liver of obese patients: an additional reinforcing lipogenic mechanism to SREBP-1c induction. J Clin Endocrinol Metab. 2011; 96(5):1424-30. 10.1210/jc.2010-2129 21325464

[308] . Machado RM, Stefano JT, Oliveira CP et al. Intake of trans fatty acids causes nonalcoholic steatohepatitis and reduces adipose tissue fat content. J Nutr. 2010; 140(6):1127-32. 10.3945/jn.109.117937 20357081

[309] . Tetri LH, Basaranoglu M, Brunt EM et al. Severe NAFLD with hepatic necroinflammatory changes in mice fed trans fats and a high-fructose corn syrup equivalent. Am J Physiol Gastrointest Liver Physiol. 2008; 295(5):G987-95. 10.1152/ajpgi.90272.2008 PMC4059366 18772365

[310] . van Herpen NA, Schrauwen Hinderling VB, Schaart G et al. Three weeks on a high-fat diet increases intrahepatic lipid accumulation anddecreases metabolic flexibility in healthy overweight men. J Clin Endocrinol Metab. 2011; 96(4):E691-5. 10.1210/jc.2010-2243 21252252

[311] . Cave M, Deaciuc I, Mendez C et al. Nonalcoholic fatty liver disease: predisposing factors and the role of nutrition. J Nutr Biochem. 2007; 18(3):184-95. 10.1016/j.jnutbio.2006.12.006 17296492

[312] . Malhi H, Bronk SF, Werneburg NW et al. Free fatty acids induce JNK dependent hepatocyte lipoapoptosis. J Biol Chem. 2006; 281(17):12093-101. 10.1074/jbc.M510660200 16505490

[313] . Shen C, Ma W, Ding L et al. The TLR4-IRE1α pathway activation contributes to palmitate-elicited lipotoxicity in hepatocytes. J Cell Mol Med. 2018; 22(7):3572-81. 10.1111/jcmm.13636 PMC6010797 29673059

[314] . Chen X, Li L, Liu X et al. Oleic acid protects saturated fatty acid mediated lipotoxicity in hepatocytes and rat of non-alcoholic steatohepatitis. Life Sci. 2018 Jun; 203:291-304. 10.1016/j.lfs.2018.04.022 29709653

[315] . Wang D, Wei Y, Pagliassotti MJ. Saturated fatty acids promote endoplasmic reticulum stress and liver injury in rats with hepatic steatosis. Endocrinology 2006; 147(2):943–51. 10.1210/en.2005-0570 16269465

[316] . Cheng Y, Zhang K, Chen Y et al. Associations between dietary nutrient intakes and hepatic lipid contents in NAFLD patients quantified by^1^H-MRS and dual-echo MRI. Nutrients. 2016;8(9). pii: E527. 10.3390/nu8090527 PMC5037514 27618908

[317] . Rosqvist F, Iggman D, Kullberg J et al. Overfeeding polyunsaturated and saturated fat causes distinct effects on liver and visceral fat accumulation in humans. Diabetes. 2014; 63(7):2356-68. 10.2337/db13-1622 24550191

[318] . Luukkonen PK, Sädevirta S, Zhou Y et al. Saturated fat is more metabolically harmful for the human liver than unsaturated fat or simple sugars. Diabetes Care. 2018; 41(8):1732-9. 10.2337/dc18-0071 PMC7082640 29844096

[319] . Cintra DE, Pauli JR, Araújo EP et al. Interleukin-10 is a protective factor against diet-induced insulin resistance in liver. J Hepatol. 2008; 48(4):628-37. 10.1016/j.jhep.2007.12.017 18267346

[320] . Errazuriz I, Dube S, Slama M et al. Randomized controlled trial of a MUFA or fiber-rich diet on hepatic fat in prediabetes. J Clin Endocrinol Metab. 2017; 102(5):1765-74. 10.1210/jc.2016-3722 PMC5443322 28323952

[321] . Bozzetto L, Prinster A, Annuzzi G et al. Liver fat is reduced by an isoenergetic MUFA diet in a controlled randomized study in type 2 diabetic patients. Diabetes Care. 2012; 35(7):1429-35. 10.2337/dc12-0033 PMC3379578 22723581

[322] . Bozzetto L, Costabile G, Luongo D et al. Reduction in liver fat by dietary MUFA in type 2 diabetes is helped by enhanced hepatic fat oxidation. Diabetologia. 2016; 59(12):2697-701. 10.1007/s00125-016-4110-5 27650287

[323] . Morari J, Torsoni AS, Anhê GF et al. The role of proliferator-activated receptor γ coactivator-1α in the fatty-acid-dependent transcriptional control of interleukin-10 in hepatic cells of rodents. Metabolism. 2010; 59(2):215-23. 10.1016/j.metabol.2009.07.020 19766270

[324] . Gormaz JG, Rodrigo R, Videla LA et al. Biosynthesis and bioavailability of long-chain polyunsaturated fatty acids in non-alcoholic fatty liver disease. Prog Lipid Res. 2010; 49(4):407-19. 10.1016/j.plipres.2010.05.003 20553760

[325] . Yamaguchi K, Yang L, McCall S et al. Inhibiting triglyceride synthesis improves hepatic steatosis but exacerbates liver damage and fibrosis in obese mice with nonalcoholic steatohepatitis. Hepatology. 2007; 45(6):1366-74. 10.1002/hep.21655 17476695

[326] . Nogueira MA, Oliveira CP, Ferreira Alves VA et al. Omega-3 polyunsaturated fatty acids in treating non-alcoholic steatohepatitis: A randomized, double-blind, placebo-controlled trial. Clin Nutr. 2016; 35(3):578-86. 10.1016/j.clnu.2015.05.001 26047766

[327] . St-Jules DE, Watters CA, Brunt EM et al. Estimation of fish and ω-3 fatty acid intake in pediatric nonalcoholic fatty liver disease. J Pediatr Gastroenterol Nutr. 2013; 57(5):627-33. 10.1097/MPG.0b013e3182a1df77 PMC3864540 24177784

[328] . Dasarathy S, Dasarathy J, Khiyami A et al. Double-blind Randomized Placebo-controlled Clinical Trial of Omega 3 Fatty Acids for the Treatment of Diabetic Patients with Nonalcoholic Steatohepatitis. J Clin Gastroenterol. 2015; 49(2):137-44. 10.1097/MCG.0000000000000099 PMC4147029 24583757

[329] . Janczyk W, Lebensztejn D, Wierzbicka-Rucińska A et al. Omega-3 fatty acids therapy in children with nonalcoholic fatty liver disease: a randomized controlled trial. J Pediatr. 2015 Jun; 166(6):1358-63.e1-3. 10.1016/j.jpeds.2015.01.056 25771388

[330] . Tobin D, Brevik-Andersen M, Qin Y et al. Evaluation of a high concentrate omega-3 for correcting the omega-3 fatty acid nutritional deficiency in non-alcoholic fatty liver disease (CONDIN). Nutrients. 2018; 10(8). pii: E1126. 10.3390/nu10081126 PMC6115838 30127297

[331] . Dossi CG, Tapia GS, Espinosa A et al. Reversal of high-fat diet-induced hepatic steatosis by n-3 LCPUFA: role of PPAR-α and SREBP-1c. J Nutr Biochem. 2014; 25(9):977-84. 10.1016/j.jnutbio.2014.04.011 24993917

[332] . Tajima-Shirasaki N, Ishii KA, Takayama H et al. Eicosapentaenoic acid down-regulates expression of the selenoprotein P gene by inhibiting SREBP-1c protein independently of the AMP-activated protein kinase pathway in H4IIEC3 hepatocytes. J Biol Chem. 2017; 292(26):10791-800. 10.1074/jbc.M116.747006 PMC5491766 28465347

[333] . Mantovani A. Plasma trans-fatty acid and risk of nonalcoholic fatty liver disease: New data from National Health and Nutrition Examination Survey (NHANES). Int J Cardiol. 2018 Dec; 272:329-330. 10.1016/j.ijcard.2018.07.136 30077528

[334] . Musso G, Cassader M, Rosina F et al. Impact of current treatments on liver disease, glucose metabolism and cardiovascular risk in non-alcoholic fatty liver disease (NAFLD): a systematic review and meta-analysis of randomised trials. Diabetologia. 2012; 55(4):885-904. 10.1007/s00125-011-2446-4 22278337

[335] . Suárez M, Boqué N, Del Bas JM et al. Mediterranean diet and multi-ingredient-based interventions for the management of non-alcoholic fatty liver disease. Nutrients. 2017; 9(10). pii: E1052. 10.3390/nu9101052 PMC5691669 28937599

[336] . Baratta F, Pastori D, Polimeni L et al. Adherence to mediterranean diet and non-alcoholic fatty liver disease: effect on insulin resistance. Am J Gastroenterol. 2017; 112(12):1832-9. 10.1038/ajg.2017.371 29063908

[337] . Vilar-Gomez E, Martinez-Perez Y, Calzadilla-Bertot L et al. Weight loss through lifestyle modification significantly reduces features of nonalcoholic steatohepatitis. Gastroenterology. 2015 Aug; 149(2):367-78.e5; quiz e14-5. 10.1053/j.gastro.2015.04.005 25865049

[338] . Proença AR, Sertié RA, Oliveira AC et al. New concepts in white adipose tissue physiology. Braz J Med Biol Res. 2014;47(3):192-205. 10.1590/1414-431X20132911 PMC3982940 24676492

[339] . Suganami T, Ogawa Y. Adipose tissue macrophages: their role in adipose tissue remodeling. J Leukoc Biol. 2010; 88(1):33-9. 10.1189/jlb.0210072 20360405

[340] . Reilly SM, Saltiel AR. Adapting to obesity with adipose tissue inflammation. Nat Rev Endocrinol. 2017; 13(11):633-43. 10.1038/nrendo.2017.90 28799554

[341] . Weisberg SP, McCann D, Desai M et al. Obesity is associated with macrophage accumulation in adipose tissue. J Clin Invest. 2003; 112(12):1796-808. 10.1172/JCI19246 PMC296995 14679176

[342] . Hotamisligil GS. Inflammation and metabolic disorders. Nature. 2006; 444(7121):860-7. 10.1038/nature05485 17167474

[343] . Cignarelli A, Genchi VA, Perrini S et al. Insulin and insulin receptors in adipose tissue development. Int J Mol Sci. 2019;20(3). pii: E759. 10.3390/ijms20030759 PMC6387287 30754657

[344] . Suganami T, Nishida J, Ogawa Y. A paracrine loop between adipocytes and macrophages aggravates inflammatory changes: role of free fatty acids and tumor necrosis factor alpha. Arterioscler Thromb Vasc Biol. 2005; 25(10):2062-8. 10.1161/01.ATV.0000183883.72263.13 16123319

[345] . Savonen R, Hiden M, Hultin M et al. The tissue distribution of lipoprotein lipase determines where chylomicrons bind. J Lipid Res. 2015; 56(3):588-98. 10.1194/jlr.M056028 PMC4340306 25589507

[346] . Takahashi K, Yamaguchi S, Shimoyama T et al. JNK- and IkappaB-dependent pathways regulate MCP-1 but not adiponectin release from artificially hypertrophied 3T3-L1 adipocytes preloaded with palmitate in vitro. Am J Physiol Endocrinol Metab. 2008; 294(5):E898-909. 10.1152/ajpendo.00131.2007 18303122

[347] . Ajuwon KM, Spurlock ME. Palmitate activates the NF-kappaB transcription factor and induces IL-6 and TNFalpha expression in 3T3-L1 adipocytes. J Nutr. 2005; 135(8):1841–6. 10.1093/jn/135.8.1841 16046706

[348] . Lee JH, Zhang Y, Zhao Z et al. Intracellular ATP in balance of pro- and anti-inflammatory cytokines in adipose tissue with and without tissue expansion. Int J Obes (Lond). 2017; 41(4):645-51. 10.1038/ijo.2017.3 PMC5380535 28074058

[349] . Finucane OM, Lyons CL, Murphy AM et al. Monounsaturated fatty acid-enriched high-fat diets impede adipose NLRP3 inflammasome-mediated IL-1β secretion and insulin resistance despite obesity. Diabetes. 2015; 64(6):2116-28. 10.2337/db14-1098 25626736

[350] . Kolak M, Westerbacka J, Velagapudi VR et al. Adipose tissue inflammation and increased ceramide content characterize subjects with high liver fat content independent of obesity. Diabetes. 2007; 56(8):1960-8. 10.2337/db07-0111 17620421

[351] . Kurotani K, Sato M, Yasuda K et al. Even- and odd-chain saturated fatty acids in serum phospholipids are differentially associated with adipokines. PLoS One. 2017; 12(5):e0178192. 10.1371/journal.pone.0178192 PMC5446160 28552966

[352] . Cintra DE, Costa AV, Peluzio Mdo C et al. Lipid profile of rats fed high-fat diets based on flaxseed, peanut, trout, or chicken skin. Nutrition. 2006; 22(2):197-205. 10.1016/j.nut.2005.09.003 16459232

[353] . Camell C, Smith CW. Dietary oleic acid increases m2 macrophages in the mesenteric adipose tissue. PLoS One. 2013; 8(9):e75147. 10.1371/journal.pone.0075147 PMC3787090 24098682

[354] . Camargo A, Rangel-Zúñiga OA, Alcalá-Díaz J et al. Dietary fat may modulate adipose tissue homeostasis through the processes of autophagy and apoptosis. Eur J Nutr. 2017; 56(4):1621-8. 10.1007/s00394-016-1208-y 27029919

[355] . Lang PD, Degott M, Heuck CC et al. Fatty acid composition of adipose tissue, blood lipids, and glucose tolerance in patients with different degrees of angiographically documented coronary arteriosclerosis. Res Exp Med (Berl). 1982; 180(2):161-8. 10.1007/BF01851055 6808619

[356] . Wood DA, Riemersma RA, Butler S et al. Linoleic and eicosapentaenoic acids in adipose tissue and platelets and risk of coronary heart disease. Lancet. 1987; 1(8526):177-83. 10.1016/s0140-6736(87)90001-8 2880015

[357] . Kark JD, Kaufmann NA, Binka F et al. Adipose tissue n-6 fatty acids and acute myocardial infarction in a population consuming a diet high in polyunsaturated fatty acids. Am J Clin Nutr. 2003; 77(4):796-802. 10.1093/ajcn/77.4.796 12663274

[358] . Ba Baylin A, Campos H. Arachidonic acid in adipose tissue is associated with nonfatal acute myocardial infarction in the central valley of Costa Rica. J Nutr. 2004; 134(11):3095-9. 10.1093/jn/134.11.3095 15514281

[359] . Nielsen MS, Schmidt EB, Stegger J et al. Adipose tissue arachidonic acid content is associated with the risk of myocardial infarction: A Danish case-cohort study. Atherosclerosis. 2013; 227(2):386-90. 10.1016/j.atherosclerosis.2012.12.035 23390891

[360] . Spencer M, Finlin BS, Unal R et al. Omega-3 fatty acids reduce adipose tissue macrophages in human subjects with insulin resistance. Diabetes. 2013; 62(5):1709-17. 10.2337/db12-1042 PMC3636648 23328126

[361] . Haghiac M, Yang XH, Presley L et al. Dietary omega-3 fatty acid supplementation reduces inflammation in obese pregnant women: a randomized double-blind controlled clinical trial. PLoS One. 2015; 10(9):e0137309. 10.1371/journal.pone.0137309 PMC4560373 26340264

[362] . Hames KC, Morgan-Bathke M, Harteneck DA et al. Very-long-chain ω -3 fatty acid supplements and adipose tissue functions: a randomized controlled trial. Am J Clin Nutr. 2017; 105(6):1552-8. 10.3945/ajcn.116.148114 PMC5445674 28424185

[363] . Fleckenstein-Elsen M, Dinnies D, Jelenik T et al. Eicosapentaenoic acid and arachidonic acid differentially regulate adipogenesis, acquisition of a brite phenotype and mitochondrial function in primary human adipocytes. Mol Nutr Food Res. 2016; 60(9):2065-75. 10.1002/mnfr.201500892 27159788

[364] . Itariu BK, Zeyda M, Hochbrugger EE et al. Long-chain n-3 PUFAs reduce adipose tissue and systemic inflammation in severely obese nondiabetic patients: a randomized controlled trial. Am J Clin Nutr. 2012; 96(5):1137-49. 10.3945/ajcn.112.037432 23034965

[365] . Eyres L, Eyres MF, Chisholm A et al. Coconut oil consumption and cardiovascular risk factors in humans. Nutr Rev. 2016; 74(4):267-80. 10.1093/nutrit/nuw002 PMC4892314 26946252

[366] . Wallace TC. Health effects of coconut oil-a narrative review of current evidence. J Am Coll Nutr. 2019; 38(2):97-107. 10.1080/07315724.2018.1497562 30395784

[367] . Bach A, Babayan V. Medium-chain triglycerides: an update. Am J Clin Nutr. 1982; 36(5):950-62. 10.1093/ajcn/36.5.950 6814231

[368] . Prior IA, Davidson F, Salmond CE et al. Cholesterol, coconuts, and diet on Polynesian atolls: a natural experiment: the Pukapuka and Tokelau Island studies. Am J Clin Nutr.1981; 34(8):1552-61. 10.1093/ajcn/34.8.1552 7270479

[369] . . Pacific islanders pay heavy price for abandoning traditional diet. Bull World Health Organ. 2010; 88(7):484-5. 10.2471/BLT.10.010710 PMC2897991 20616964

[370] . Voon PT, Ng TK, Lee VK et al. Diets high in palmitic acid (16:0), lauric and myristic acids (12:0 + 14:0), or oleic acid (18:1) do not alter postprandial or fasting plasma homocysteine and inflammatory markers in healthy Malaysian adults. Am J Clin Nutr. 2011; 94(6):1451-7. 10.3945/ajcn.111.020107 22030224

[371] . Cox C, Sutherland W, Mann J et al. Effects of dietary coconut oil, butter and safflower oil on plasma lipids, lipoproteins and lathosterol levels. Eur J Clin Nutr. 1998; 52(9):650-4. 10.1038/sj.ejcn.1600621 9756121

[372] . Denke MA, Grundy SM. Comparison of effects of lauric acid and palmitic acid on plasma lipids and lipoproteins. Am J Clin Nutr.1992; 56(5):895-8. 10.1093/ajcn/56.5.895 1415008

[373] . Mendis S, Kumarasunderam R. The effect of daily consumption of coconut fat and soya-bean fat on plasma lipids and lipoproteins of young normolipidaemic men. Br J Clin Nutr. 1990; 63(3):547-52. 10.1079/bjn19900141 2383532

[374] . Feranil AB, Duazo PL, Kuzawa CW et al. Coconut oil is associated with a beneficial lipid profile in pre-menopausal women in the Philippines. Asia Pac J Clin Nutr. 2011; 20(2):190-5. PMC3146349 21669587

[375] . Velloso LA, Folli F, Saad MJ. TLR4 at the crossroads of nutrients, gut microbiota, and metabolic inflammation. Endocr Rev. 2015; 36(3):245-71. 10.1210/er.2014-1100 25811237

[376] . Lee JY, Ye J, Gao Z et al. Reciprocal modulation of Toll-like receptor-4 signaling pathways involving MyD88 and phosphatidylinositol 3-kinase/AKT by saturated and polyunsaturated fatty acids. J Biol Chem. 2003; 278(39):37041-51. 10.1074/jbc.M305213200 12865424

[377] . Weatherill AR, Lee JY, Zhao L et al. Saturated and polyunsaturated fatty acids reciprocally modulate dendritic cell functions mediated through TLR4. J Immunol. 2005; 174(9):5390-7. 10.4049/jimmunol.174.9.5390 15843537

[378] . Valente FX, Cândido FG, Lopes LL et al. Effects of coconut oil consumption on energy metabolism, cardiometabolic risk markers, and appetitive responses in women with excess body fat. Eur J Nutr. 2017; 57(4):1627-37. 10.1007/s00394-017-1448-5 28405814

[379] . Karupaiah T, Chuah KA, Chinna K et al. Comparing effects of soybean oil- and palm olein-based mayonnaise consumption on the plasma lipid and lipoprotein profiles in human subjects: a double-blind randomized controlled trial with cross-over design. Lipids Health Dis. 2016.17;15(1):131. 10.1186/s12944-016-0301-9 PMC4989497 27535127

[380] . Sun Y, Neelakantan N, Wu Y et al. Palm Oil Consumption Increases LDL cholesterol compared with vegetable oils low in saturated fat in a meta-analysis of clinical trials. J Nutr. 2015; 145(7):1549-58. 10.3945/jn.115.210575 25995283

[381] . Tholstrup T, Hjerpsted J, Raff M. Palm olein increases plasma cholesterol moderately compared with olive oil in healthy individuals. Am J Clin Nutr. 2011; 94(6):1426-32. 10.3945/ajcn.111.018846 22071711

[382] . Torres-Moreno M, Torrescasana E, Salas-Salvadó J et al. Nutritional composition and fatty acids profile in cocoa beans and chocolates with different geographical origin and processing conditions. Food Chem. 2015; 166:125-32. 10.1016/j.foodchem.2014.05.141 25053037

[383] . Bonanome A, Grundy SM. Effect of dietary stearic acid on plasma cholesterol and lipoprotein levels. N Engl J Med. 1988; 318(19):1244-8. 10.1056/NEJM198805123181905 3362176

[384] . Brassard D, Tessier-Grenier M, Allaire J et al. Comparison of the impact of SFAs from cheese and butter on cardiometabolic risk factors: a randomized controlled trial. Am J Clin Nutr. 2017; 105(4):800-9. 10.3945/ajcn.116.150300 28251937

[385] . Ericson U, Hellstrand S, Brunkwall L et al. Food sources of fat may clarify the inconsistent role of dietary fat intake for incidence of type 2 diabetes. Am J Clin Nutr. 2015; 101(5):1065-80. 10.3945/ajcn.114.103010 25832335

[386] . Avalos EE, Barrett-Connor E, Kritz-Silverstein D et al. Is dairy product consumption associated with the incidence of CHD? Public Health Nutr. 2013; 16(11):2055-63. 10.1017/S1368980012004168 PMC10271263 23026077

[387] . de Oliveira Otto MC, Mozaffarian D, Kromhout D et al. Dietary intake of saturated fat by food source and incident cardiovascular disease: the Multi-Ethnic Study of Atherosclerosis. Am J Clin Nutr. 2012; 96(2):397-404. 10.3945/ajcn.112.037770 PMC3396447 22760560

[388] . Pimpin L, Wu JH, Haskelberg H et al. Is butter back? A systematic review and meta-analysis of butter consumption and risk of cardiovascular disease, diabetes, and total mortality. Plos One. 2016; 11(6):e0158118. 10.1371/journal.pone.0158118 PMC4927102 27355649

[389] . Azadbakht L, Fard NR, Karimi M et al. Effects of the Dietary Approaches to Stop Hypertension (DASH) eating plan on cardiovascular risks among type 2 diabetic patients: a randomized crossover clinical trial. Diabetes Care. 2011; 34(1):55-7. 10.2337/dc10-0676 PMC3005461 20843978

[390] . Buse JB, Ginsberg HN, Bakris GL et al. American Heart Association; American Diabetes Association. Primary prevention of cardiovascular diseases in people with diabetes mellitus: a scientific statement from the American Heart Association and the American Diabetes Association. Circulation. 2007; 115(1):114-26. 10.1161/CIRCULATIONAHA.106.179294 17192512

[391] . Yakoob MY, Shi P, Willett WC et al. Circulating biomarkers of dairy fat and risk of incident diabetes mellitus among men and women in the United States in two large prospective cohorts. Circulation. 2016; 133(17):1645-54. 10.1161/CIRCULATIONAHA.115.018410 PMC4928633 27006479

[392] . Yakoob MY, Shi P, Hu FB et al. Circulating biomarkers of dairy fat and risk of incident stroke in U.S. men and women in 2 large prospective cohorts. Am J Clin Nutr 2014; 100(6):1437-47. 10.3945/ajcn.114.083097 PMC4232012 25411278

[393] . Afshin A, Micha R, Khatibzadeh S et al; 2010 Global Burden of Diseases, Injuries, and Risk Factors Study: NUTRItrition and ChrOnic Diseases Expert Group (NUTRICODE), and Metabolic Risk Factors of ChrOnic Diseases Collaborating Group. The impact of dietary habits and metabolic risk factors on cardiovascular and diabetes mortality in countries of the Middle East and North Africa in 2010: a comparative risk assessment analysis. BMJ Open. 2015; 5(5):e006385. 10.1136/bmjopen-2014-006385 PMC4442236 25995236

[394] . Lenighan YM, Nugent AP, Li KF et al. Processed red meat contribution to dietary patterns and the associated cardio-metabolic outcomes. Br J Nutr. 2017; 118(3):222-8. 10.1017/S0007114517002008 28831958

[395] . Bellavia A, Stilling F, Wolk A. High red meat intake and all-cause cardiovascular and cancer mortality: is the risk modified by fruit and vegetable intake? Am J Clin Nutr. 2016; 104(4):1137-43. 10.3945/ajcn.116.135335 27557655

[396] . O’Sullivan TA, Hafekost K, Mitrou F, Lawrence D. Food sources of saturated fat and the association with mortality: a meta-analysis. Am J Public Health. 2013; 103(9):e31-42. 10.2105/AJPH.2013.301492 PMC3966685 23865702

[397] . Wang X, Lin X, Ouyang YY et al. Red and processed meat consumption and mortality: dose-response meta-analysis of prospective cohort studies. Public Health Nutr. 2016; 19(5):893-905. 10.1017/S1368980015002062 PMC10270853 26143683

[398] . de Wit N, Derrien M, Bosch-Vermeulen H et al. Saturated fat stimulates obesity and hepatic steatosis and affects gut microbiota composition by an enhanced overflow of dietary fat to the distal intestine. Am J Physiol Gastrointest Liver Physiol. 2012; 303(5):G589-99. 10.1152/ajpgi.00488.2011 22700822

[399] . Liu T, Hougen H, Vollmer AC et al. Gut bacteria profiles of Mus musculus at the phylum and family levels are influenced by saturation of dietary fatty acids. Anaerobe. 2012; 18(3):331-7. 10.1016/j.anaerobe.2012.02.004 22387300

[400] . Devkota S, Wang Y, Musch MW et al. Dietary-fat-induced taurocholic acid promotes pathobiont expansion and colitis in Il10−/− mice. Nature. 2012; 487(7405):104-8. 10.1038/nature11225 PMC3393783 22722865

[401] . Cani PD, Amar J, Iglesias MA et al. Metabolic endotoxemia initiates obesity and insulin resistance. Diabetes. 2007; 56(7):1761-72. 10.2337/db06-1491 17456850

[402] . Cani PD, Bibiloni R, Knauf C et al. Changes in gut microbiota control metabolic endotoxemia-induced inflammation in high-fat diet–induced obesity and diabetes in mice. Diabetes. 2008; 57(6):1470-81. 10.2337/db07-1403 18305141

[403] . Pendyala S, Walker JM, Holt PR. A high-fat diet is associated with endotoxemia that originates from the gut. Gastroenterology. 2012; 142(5):1100-1.e2. 10.1053/j.gastro.2012.01.034 PMC3978718 22326433

[404] . Laugerette F, Vors C, Peretti N et al. Complex links between dietary lipids, endogenous endotoxins and metabolic inflammation. Biochimie. 2011; 93(1):39-45. 10.1016/j.biochi.2010.04.016 20433893

[405] . Carvalho BM, Saad MJ. Influence of gut microbiota on subclinical inflammation and insulin resistance. Mediators Inflamm. 2013 Jun; 2013:986734. 10.1155/2013/986734 PMC3694527 23840101

[406] . Huang EY, Leone VA, Devkota S et al. Composition of dietary fat source shapes gut microbiota architecture and alters host inflammatory mediators in mouse adipose tissue. JPEN J Parenter Enteral Nutr. 2013; 37(6):746-54. 10.1177/0148607113486931 PMC3812400 23639897

[407] . López-Moreno J, García-Carpintero S, Jimenez-Lucena R et al. Effect of dietary lipids on endotoxemia influences postprandial inflammatory response. J Agric Food Chem. 2017; 65(35):7756-63. 10.1021/acs.jafc.7b01909 28793772

[408] . He C, Shan Y, Song W. Targeting gut microbiota as a possible therapy for diabetes. Nutr Res. 2015; 35(5):361-7. 10.1016/j.nutres.2015.03.002 25818484

[409] . Lam YY, Ha CW, Hoffmann JM et al. Effects of dietary fat profile on gut permeability and microbiota and their relationships with metabolic changes in mice. Obesity (Silver Spring). 2015; 23(7):1429-39. 10.1002/oby.21122 26053244

[410] . Wiest R, Rath HC. Bacterial translocation in the gut. Best Pract Res Clin Gastroenterol. 2003; 17(3):397-425. 10.1016/s1521-6918(03)00024-6 12763504

[411] . Fasano A. Zonulin and its regulation of intestinal barrier function: the biological door to inflammation, autoimmunity, and cancer. Physiol Rev. 2011; 91(1):151-75. 10.1152/physrev.00003.2008 21248165

[412] . Kim KA, Gu W, Lee IA et al. High fat diet-induced gut microbiota exacerbates inflammation and obesity in mice via the TLR4 signaling pathway. PLoS One. 2012; 7(10):e47713. 10.1371/journal.pone.0047713 PMC3473013 23091640

[413] . de La Serre CB, Ellis CL, Lee J et al. Propensity to high-fat diet-induced obesity in rats is associated with changes in the gut microbiota and gut inflammation. Am J Physiol Gastrointest Liver Physiol. 2010; 299(2):G440-8. 10.1152/ajpgi.00098.2010 PMC2928532 20508158

[414] . Paulino G, Barbier de la Serre C, Knotts TA et al. Increased expression of receptors for orexigenic factors in nodose ganglion of diet-induced obese rats. Am J Physiol Endocrinol Metab. 2009; 296(4):E898-903. 10.1152/ajpendo.90796.2008 PMC2670626 19190260

[415] . Wu GD, Chen J, Hoffmann C et al. Linking long-term dietary patterns with gut microbial enterotypes. Science. 2011; 334(6052):105-8. 10.1126/science.1208344 PMC3368382 21885731

[416] . Wan Y, Wang F, Yuan J et al. Effects of dietary fat on gut microbiota and faecal metabolites, and their relationship with cardiometabolic risk factors: a 6-month randomised controlled-feeding trial. Gut. 2019; 68(8):1417-29. 10.1136/gutjnl-2018-317609 30782617

[417] . Kaoutari A El, Armougom F, Gordon JI et al. The abundance and variety of carbohydrate-active enzymes in the human gut microbiota. Nat Rev Microbiol. 2013; 11(7):497-504. 10.1038/nrmicro3050 23748339

[418] . Canfora EE, Jocken JW, Blaak EE. Short-chain fatty acids in control of body weight and insulin sensitivity. Nat Rev Endocrinol. 2015; 11(10):577-91. 10.1038/nrendo.2015.128 26260141

[419] . Louis P, Flint HJ. Formation of propionate and butyrate by the human colonic microbiota. Environ Microbiol. 2017;19(1):29-41. 10.1111/1462-2920.13589 27928878

[420] . Russell WR, Gratz SW, Duncan SH et al. High-protein, reduced-carbohydrate weight-loss diets promote metabolite profiles likely to be detrimental to colonic health. Am J Clin Nutr. 2011; 93(5):1062-72. 10.3945/ajcn.110.002188 21389180

[421] . Djousse L, Gaziano JM. Egg consumption in relation to cardiovascular disease and mortality: the Physicians’ Health Study. Am J Clin Nutr. 2008; 87(4):964-9. 10.1093/ajcn/87.4.964 PMC2386667 18400720

[422] . McGill HC, Jr. The relationship of dietary cholesterol to serum cholesterol concentration and to atherosclerosis in man. Am J Clin Nutr. 1979; 32(12 Suppl):2664-702. 10.1093/ajcn/32.12.2664 389024

[423] . Song JW, Chung KC. Observational studies: cohort and case-control studies. Plast Reconstr Surg. 2010; 126(6):2234-42. 10.1097/PRS.0b013e3181f44abc PMC2998589 20697313

[424] . Hu FB, Manson JE, Willett WC. Types of dietary fat and risk of coronary heart disease: a critical review. J Am Coll Nutr. 2001; 20(1):5-19. 10.1080/07315724.2001.10719008 11293467

[425] . Institute of Medicine. Dietary Reference Intakes for Energy, Carbohydrate, Fiber, Fat, Fatty Acids, Cholesterol, Protein, and Amino Acids. Washington (DC): The National Academies Press; 2002. 10.1016/s0002-8223(02)90346-9 12449285

[426] . McNamara DJ, Kolb R, Parker TS et al. Heterogeneity of cholesterol homeostasis in man. Response to changes in dietary fat quality and cholesterol quantity. J Clin Invest. 1987; 79(6):1729-39. 10.1172/JCI113013 PMC424515 3584466

[427] . Berger S, Raman G, Vishwanathan R et al. Dietary cholesterol and cardiovascular disease: a systematic review. Am J Clin Nutr. 2015; 102(2):276-94. 10.3945/ajcn.114.100305 26109578

[428] . Kosmas CE, Martinez I, Sourlas A et al. High-density lipoprotein (HDL) functionality and its relevance to atherosclerotic cardiovascular disease. Drugs Context. 2018 Mar; 7:212525. 10.7573/dic.212525 PMC5877920 29623098

[429] . Fuller NR, Caterson ID, Sainsbury A et al. The effect of a high-egg diet on cardiovascular risk factors in people with type 2 diabetes: the Diabetes and Egg (DIABEGG) study-a 3-mo randomized controlled trial. Am J Clin Nutr. 2015; 101(4):705-13. 10.3945/ajcn.114.096925 25833969

[430] . Radzeviciene L, Ostrauskas R. Egg consumption and the risk of type 2 diabetes mellitus: a case-control study. Pub Health Nutr 2012; 15(8):1437-41. 10.1017/S1368980012000614 22390963

[431] . Djoussé L, Gaziano JM, Buring JE et al. Egg consumption and risk of type 2 diabetes in men and women. Diabetes Care. 2009; 32(2):295-300. 10.2337/dc08-1271 PMC2628696 19017774

[432] . Kurotani K, Nanri A, Goto A et al. Cholesterol and egg intakes and the risk of type 2 diabetes: the Japan Public Health Center-based Prospective Study. Br J Nutr. 2014; 112(10):1636-43. 10.1017/S000711451400258X 25230771

[433] . Virtanen JK, Mursu J, Tuomainen TP et al. Egg consumption and risk of incident type 2 diabetes in men: the Kuopio Ischaemic Heart Disease Risk Factor Study. Am J Clin Nutr. 2015; 101(5):1088-96. 10.3945/ajcn.114.104109 25832339

[434] . Djoussé L, Petrone AB, Hickson DA et al. Egg consumption and risk of type 2 diabetes among African Americans: The Jackson Heart Study. Clin Nutr. 2016; 35(3):679-84. 10.1016/j.clnu.2015.04.016 PMC4627855 25971658

[435] . Geiker NRW, Larsen ML, Dyerberg J et al. Egg consumption, cardiovascular diseases and type 2 diabetes. Eur J Clin Nutr. 2018; 72(1):44-56. 10.1038/ejcn.2017.153 28952608

[436] . Tran NL, Barraj LM, Heilman JM et al. Egg consumption and cardiovascular disease among diabetic individuals: a systematic review of the literature. Diab Metab Syndr Obes. 2014 Mar; 7:121-37. 10.2147/DMSO.S58668 PMC3969252 24711708

[437] . Richard C, Cristall L, Fleming E et al. Impact of egg consumption on cardiovascular risk factors in individuals with type 2 diabetes and at risk for developing diabetes: a systematic review of randomized nutritional intervention studies. Can J Diabetes. 2017; 41(4):453-63 10.1016/j.jcjd.2016.12.002 28359773

[438] . Jang J, Shin MJ, Kim OY et al. Longitudinal association between egg consumption and the risk of cardiovascular disease: interaction with type 2 diabetes mellitus. Nutr Diabetes. 2018; 8(1):20. 10.1038/s41387-018-0033-1 PMC5916923 29695709

[439] . Shin JY, Xun P, Nakamura Y et al. Egg consumption in relation to risk of cardiovascular disease and diabetes: a systematic review and meta-analysis. Am J Clin Nutr. 2013; 98(1):146-59. 10.3945/ajcn.112.051318 PMC3683816 23676423

[440] . Tanasescu M, Cho E, Manson JE et al. Dietary fat and cholesterol and the risk of cardiovascular disease among women with type 2 diabetes. Am J Clin Nutr. 2004; 79(6):999-1005. 10.1093/ajcn/79.6.999 15159229

[441] . Baghdasarian S, Lin HP, Pickering RT et al. Dietary Cholesterol Intake Is Not Associated with Risk of Type 2 Diabetes in the Framingham Offspring Study. Nutrients. 2018; 10(6). pii: E665. 10.3390/nu10060665 PMC6024792 29794966

[442] . Díez-Espino J, Basterra-Gortari FJ, Salas-Salvadó J et al; PREDIMED Investigators. Egg consumption and cardiovascular disease according to diabetic status: The PREDIMED study. ClinNutr. 2017; 36(4):1015-21. 10.1016/j.clnu.2016.06.009 27448949

[443] . Cheng P, Pan J, Xia J et al. Dietary cholesterol intake and stroke risk: a meta-analysis. Oncotarget. 2018; 9(39):25698-707. 10.18632/oncotarget.23933 PMC5986647 29876017

[444] . Larsson SC, Åkesson A, Wolk A. Egg consumption and risk of heart failure, myocardial infarction, and stroke: results from 2 prospective cohorts. Am J Clin Nutr. 2015; 102(5):1007-13. 10.3945/ajcn.115.119263 26399866

[445] . Rhee EJ, Ryu S, Lee JY et al. The association between dietary cholesterol intake and subclinical atherosclerosis in Korean adults: The Kangbuk Samsung Health Study. J Clin Lipidol. 2017; 11(2):432-41.e3. 10.1016/j.jacl.2017.01.021 28502500

[446] . Virtanen JK, Mursu J, Virtanen HEK et al. Associations of egg and cholesterol intakes with carotid intima-media thickness and risk of incident coronary artery disease according to apolipoprotein E phenotype in men: the Kuopio Ischaemic Heart Disease Risk Factor Study. Am J Clin Nutr. 2016; 103(3):895-901. 10.3945/ajcn.115.122317 26864369

[447] . Zhong VW, Van Horn L, Cornelis MC et al. Associations of dietary cholesterol or egg consumption with incident cardiovascular disease and mortality. JAMA. 2019; 321(11):1081-95. 10.1001/jama.2019.1572 PMC6439941 30874756

[448] . Clayton ZS, Fusco E, Kern M. Egg consumption and heart health: A review. Nutrition. 2017 May; 37:79-85. 10.1016/j.nut.2016.12.014 28359368

[449] . Blesso CN, Fernandez ML. Dietary cholesterol, serum lipids, and heart disease: Are eggs working for or against you? Nutrients. 2018; 10(4). pii: E426. 10.3390/nu10040426 PMC5946211 29596318

[450] . Mott MM, McCrory MA, Bandini LG et al. Egg intake has no adverse association with blood lipids or glucose in adolescent girls. J Am Coll Nutr. 2019; 38(2):119-24. 10.1080/07315724.2018.1469437 PMC6377328 30280988

[451] . Clayton ZS, Scholar KR, Shelechi M et al. Influence of resistance training combined with daily consumption of an egg-based or bagel-based breakfast on risk factors for chronic diseases in healthy untrained individuals. J Am Coll Nutr. 2015; 34(2):113-9. 10.1080/07315724.2014.946622 25785868

[452] . Rouhani MH, Rashidi-Pourfard N, Salehi-Abargouei A et al. Effects of Egg Consumption on Blood Lipids: A Systematic Review and Meta-Analysis of Randomized Clinical Trials. J Am Coll Nutr. 2018; 37(2):99-110. 10.1080/07315724.2017.1366878 29111915

[453] . Alexander DD, Miller PE, Vargas AJ et al. Meta-analysis of Egg Consumption and Risk of Coronary Heart Disease and Stroke. J Am Coll Nutr. 2016; 35(8):704-16. 10.1080/07315724.2016.1152928 27710205

[454] . Xu L, Lam TH, Qiang C et al. Egg consumption and the risk of cardiovascular disease and all-cause mortality: Guangzhou Biobank Cohort Study and meta-analyses. Eur J Nutr. 2019; 58(2):785-96. 10.1007/s00394-018-1692-3 29680985

[455] . Dehghan M, Mente A, Rangarajan S, et al. Association of egg intake with blood lipids, cardiovascular disease, and mortality in 177,000 people in 50 countries. *Am J Clin Nutr* . 2020;111(4):795-803. 10.1093/ajcn/nqz348 PMC7138651 31965140

[456] . Chagas P, Caramori P, Galdino TP et al. Egg consumption and coronary atherosclerotic burden. Atherosclerosis. 2013;229(2):381-4. 10.1016/j.atherosclerosis.2013.05.008 23880191

[457] . Katz DL, Gnanaraj J, Treu JA et al. Effects of egg ingestion on endothelial function in adults with coronary artery disease: A randomized, controlled, crossover trial. Am Heart J. 2015; 169(1):162-9. 10.1016/j.ahj.2014.10.001 25497262

[458] . Wu ZX, Li SF, Chen H et al. The changes of gut microbiota after acute myocardial infarction in rats. PLoS One. 2017; 12(7):e0180717. 10.1371/journal.pone.0180717 PMC5501596 28686722

[459] . Davies A, Lüscher TF. The red and the white, and the difference it makes. Eur Heart J. 2019; 40(7):595-7. 10.1093/eurheartj/ehy905 30649383

[460] . Lemos BS, Medina-Vera I, Malysheva OV et al. Effects of Egg Consumption and Choline Supplementation on Plasma Choline and Trimethylamine-N-Oxide in a Young Population. J Am Coll Nutr. 2018 May;1-8. 10.1080/07315724.2018.1466213 29764315

[461] . Jonsson AL, Bäckhed F. Role of gut microbiota in atherosclerosis. Nat Rev Cardiol. 2017;14(2):79-87. 10.1038/nrcardio.2016.183 27905479

[462] . Canyelles M, Tondo M, Cedó L et al. Trimethylamine N-oxide: A link among diet, gut microbiota, gene regulation of liver and intestine cholesterol homeostasis and HDL function. Int J Mol Sci. 2018; 19(10). pii: E3228. 10.3390/ijms19103228 PMC6214130 30347638

[463] . Ding L, Chang M, Guo Y et al. Trimethylamine-N-oxide (TMAO)-induced atherosclerosis is associated with bile acid metabolism. Lipids Health Dis. 2018; 17(1):286. 10.1186/s12944-018-0939-6 PMC6300890 30567573

[464] . Cho CE, Caudill MA. Trimethylamine-N-Oxide: Friend, Foe, or Simply Caught in the Cross-Fire? Trends Endocrinol Metab. 2017; 28(2):121-130. 10.1016/j.tem.2016.10.005 27825547

[465] . QI J, You T, Li J et al. Circulating trimethylamine N-oxide and the risk of cardiovascular diseases: a systematic review and meta-analysis of 11 prospective cohort studies. J Cell Mol Med. 2018; 22(1):185-94. 10.1111/jcmm.13307 PMC5742728 28782886

[466] . Wang Z, Bergeron N, Levison BS et al. Impact of chronic dietary red meat, white meat, or non-meat protein on trimethylamine N-oxide metabolism and renal excretion in healthy men and women. Eur Heart J. 2019; 40(7):583-94. 10.1093/eurheartj/ehy799 PMC6374688 30535398

[467] . Schiattarella GG, Sannino A, Toscano E et al. Gut microbe-generated metabolite trimethylamine-N-oxide as cardiovascular risk biomarker: A systematic review and dose-response meta-analysis. Eur Heart J. 2017; 38(39):2948-56. 10.1093/eurheartj/ehx342 29020409

[468] . Missimer A, Fernandez ML, DiMarco DM et al. Compared to an oatmeal breakfast, two eggs/day increased plasma carotenoids and choline without increasing trimethyl amine N-Oxide concentrations. J Am Coll Nutr. 2018; 37(2):140-8. 10.1080/07315724.2017.1365026 29313753

[469] . Wang X, Tanaka N, Hu X et al. A high-cholesterol diet promotes steatohepatitis and livertumorigenesis in HCV core gene transgenic mice. Arch Toxicol. 2019; 93(6):1727-8. 10.1007/s00204-019-02491-w 31177290

[470] . Ioannou GN. The Role of Cholesterol in the Pathogenesis of NASH. TrendsEndocrinolMetab. 2016; 27(2):84-95. 10.1016/j.tem.2015.11.008 26703097

[471] . Subramanian S, Goodspeed L, Wang S et al. Dietary cholesterol exacerbateshepatic steatosis and inflammation in obese LDL receptor-deficient mice. J Lipid Res. 2011; 52(9):1626-35. 10.1194/jlr.M016246 PMC3151683 21690266

[472] . Savard C, Tartaglione EV, Kuver R et al. Synergistic interaction of dietary cholesteroland dietary fat in inducing experimental steatohepatitis. Hepatology. 2013; 57(1):81-92. 10.1002/hep.25789 PMC5341743 22508243

[473] . Berry S. Triacylglycerol structure and interesterification of palmitic and stearic acid-rich fats: an overview and implications for cardiovascular disease. Nutr Res Rev. 2009; 22(1):3-17. 10.1017/S0954422409369267 19442321

[474] . Miyamoto JÉ, Ferraz ACG, Portovedo M et al. Interesterified soybean oil promotes weight gain, impaired glucose tolerance and increased liver cellular stress markers. J Nutr Biochem. 2018 Sep; 59:153-9. 10.1016/j.jnutbio.2018.05.014 30005920

[475] . Reena MB, Lokesh BR. Hypolipidemic effect of oils with balanced amounts of fatty acids obtained by blending and interesterification of coconut oil with rice bran oil or sesame oil. J Agric Food Chem. 2007; 55(25):10461-9 10.1021/jf0718042 17994696

[476] . Reena MB, Gowda LR, Lokesh BR. Enhanced hypocholesterolemic effects of interesterified oils are mediated by upregulating LDL receptor and cholesterol 7-α- hydroxylase gene expression in rats. J Nutr. 2011; 141(1):24-30. 10.3945/jn.110.127027 21106933

[477] . Afonso MS, Lavrador MS, Koike MK et al. Dietary interesterified fat enriched with palmitic acid induces atherosclerosis by impairing macrophage cholesterol efflux and eliciting inflammation. J Nutr Biochem. 2016 Jun; 32:91-100. 10.1016/j.jnutbio.2016.01.005 27142741

[478] . Lavrador MSF, Afonso MS, Cintra DE et al. Interesterified fats induce deleterious effects on adipose tissue and liver in LDLr-KO mice. Nutrients. 2019; 11(2). pii: E466. 10.3390/nu11020466 PMC6412707 30813339

[479] . Magri TP, Fernandes FS, Souza AS et al. Interesterified fat or palm oil as substitutes for partially hydrogenated fat in maternal diet can predispose obesity in adult male offspring. Clin Nutr. 2015; 34(5):904-10. 10.1016/j.clnu.2014.09.014 25444555

[480] . Misan V, Estato V, de Velasco PC et al. Interesterified fat or palm oil as substitutes for partially hydrogenated fat during the perinatal period produces changes in the brain fatty acids profile and increases leukocyte- endothelial interactions in the cerebral microcirculation from the male offspring in adult life. Brain Res. 2015 Aug; 1616:123-33. 10.1016/j.brainres.2015.05.001 25982597

[481] . Sundram K, Karupaiah T, Hayes KC. Stearic acid-rich interesterified fat and trans-rich fat raise the LDL/HDL ratio and plasma glucose relative to palm olein in humans. Nutr Metab. (London) 2007 Jan; 4:3. 10.1186/1743-7075-4-3 PMC1783656 17224066

[482] . Filippou A, Teng KT, Berry SE et al. Palmitic acid in the sn-2 position of dietary triacylglycerols does not affect insulin secretion or glucose homeostasis in healthy men and women. Eur J Clin Nutr. 2014; 68(9):1036-41. 10.1038/ejcn.2014.141 PMC4155797 25052227

[483] . Nestel PJ, Noakes M, Belling GB et al. Effect on plasma lipids of interesterifying a mix edible oils. Am J Clin Nutr. 1995; 62(5):950-5. 10.1093/ajcn/62.5.950 7572740

[484] . Wang CH, Kuksis A, Manganaro F. Studies of the substrate specificity of purified human milk lipoprotein lipase. Lipids. 1982; 17(4):278-84. 10.1007/BF02534942 7078358

[485] . Yli-Jokipii K, Kallio H, Schwab U et al. Effects of palm oil and transesterified palm oil on chylomicron and VLDL triacylglycerol structures and postprandial lipid response. J Lipid Res. 2001; 42(10):1618-25. 11590218

[486] . Sanders TA, Filippou A, Berry SE et al. Palmitic acid in the sn-2 position of triacylglycerols acutely influences postprandial lipid metabolism. Am J Clin Nutr. 2011;94(6):1433-41. 10.3945/ajcn.111.017459 22030225

[487] . Hall WL, Brito MF, Huang J et al. An interesterified palm olein test meal decreases early-phase postprandial lipemia compared to palm olein: a randomized controlled trial. Lipids. 2014; 49(9):895-904. 10.1007/s11745-014-3936-1 25103522

[488] . Robinson DM, Martin NC, Robinson LE et al. Influence of interesterification of a stearic Acid-rich spreadable fat on acute metabolic risk factor. Lipids. 2009;44(1):17-26. 10.1007/s11745-008-3253-7 18982377

[489] . Meijer GW, Weststrate JA. Interesterification of fats in margarine: effect on blood lipids, blood enzymes, and hemostasis parameters. Eur J Clin Nutr. 1997; 51(8):527-34. 10.1038/sj.ejcn.1600437 11248878

[490] . Bach AC, Ingenbleek Y, Frey A. The usefulness of dietary medium-chain triglycerides in body weight control: fact or fancy? J Lipid Res. 1996; 37(4):708-26. 8732772

[491] . Williams L, Wilson DP. Editorial commentary: Dietary management of familial chylomicronemia syndrome. J Clin Lipidol. 2016; 10(3):462-5. 10.1016/j.jacl.2015.12.023 27206931

[492] . Hill JO, Peters JC, Swift LL et al. Changes in blood lipids during six days of overfeeding with medium or long chain triglycerides. J Lipid Res. 1990; 31(3):407-16. 2187945

[493] . Swift LL, Hill JO, Peters JC et al. Medium-chain fatty acids: evidence for incorporation into chylomicron triglycerides in humans. Am J Clin Nutr. 1990;52(5):834-6. 10.1093/ajcn/52.5.834 2239759

[494] . Burnett JR, Hooper AJ, Hegele RA. Familial lipoprotein lipase deficiency. In: Adam MP, Ardinger HH, Pagon RA et al., editors. GeneReviews. Seattle (WA): University of Washington, Seattle. p. 1993–2018, Available at: https://www.ncbi.nlm.nih.gov/books/ NBK1308/. Accessed June 09, 2020. 20301485

[495] . Pouwels ED, Blom DJ, Firth JC et al. Severe hypertriglyceridaemia as a result of familial chylomicronaemia: the Cape Town experience. S Afr Med J. 2008;98:105–108. 18350203

[496] . Brahm AJ, Hegele RA. Chylomicronaemia- current diagnosis and future therapies. Nat Rev Endocrinol. 2015;11:352–362. 10.1038/nrendo.2015.26 25732519

[497] . Moulin P, Dufour R, Averna M et al. Identification and Diagnosis of Patients With Familial Chylomicronaemia Syndrome (FCS): Expert Panel Recommendations and Proposal of an “FCS Score”. Atherosclerosis 2018;275:265-272. 10.1016/j.atherosclerosis.2018.06.814 29980054

[498] . Witzum JL, Gaudet D, Freedman SD et al. Volanesorsen and Triglyceride Levels in Familial Chylomicronemia Syndrome. N Engl J Med 2019; 381:531-542. 10.1056/NEJMoa1715944 31390500

[499] . Stroes E, Moulin P, Parhofer KG et al. Diagnostic algorithm for familial chylomicronemia syndrome. Atheroscler Suppl. 2017;23:1–7. 10.1016/j.atherosclerosissup.2016.10.002 27998715

[500] . Hegele RA, Ginsberg HM, Chapman MJ et al, European Atherosclerosis Society Consensus Panel. The polygenic nature of hypertriglyceridaemia: implications for definition, diagnosis and management. Lancet Diabetes Endocrinol. 2014;2:655–666. 10.1016/S2213-8587(13)70191-8 PMC4201123 24731657

[501] . Gan SI, Edwards AL, Symonds CJ, Beck PL. Hypertriglyceridemia - induced pancreatitis: A case-based review. World J Gastroenterol. 2006;12:7197–7202. 10.3748/wjg.v12.i44.7197 PMC4087786 17131487

[502] . Valdivielso P, Ramirez-Bueno A, Ewald N. Current knowledge of hypertriglyceridemic pancreatitis. Eur J Intern Med. 2014;25:689–694. 10.1016/j.ejim.2014.08.008 25269432

[503] . Brown WV, Goldberg IJ, Young SG. JCL Roundtable: Hypertriglyceridemia due to defects in lipoprotein lipase function. J Clin Lipidol. 2015;9:274–280. 10.1016/j.jacl.2015.03.009 PMC4578811 26073384

[504] . Nawaz H, Koutroumpakis E, Easler J et al. Elevated serum triglycerides are independently associated with persistent organ failure in acute pancreatitis. Am J Gastroenterol. 2015;110:1497–1503. 10.1038/ajg.2015.261 26323188

[505] . Yang F, Wang Y, Sternfeld L et al. The role of free fatty acids, pancreatic lipase and Ca21 signalling in injury of isolated acinar cells and pancreatitis model in lipoprotein lipase-deficient mice. Acta Physiol (Oxf). 2009;195:13–28. 10.1111/j.1748-1716.2008.01933.x 18983441

[506] . Berglund L, Brunzell JD, Goldberg AC et al. Endocrine society. Evaluation and treatment of hypertriglyceridemia: An Endocrine Society clinical practice guideline. J Clin Endocrinol Metab. 2012;97:2969–2989. 10.1210/jc.2011-3213 PMC3431581 22962670

[507] . Gaudet D, Methot T, Dery S et al. Efficacy and long-term safety of alipogene tiparvovec (AAV1-LPLS447X) gene therapy for lipoprotein lipase deficiency: An open-label trial. Gene Ther. 2013;20:361–369. 10.1038/gt.2012.43 PMC4956470 22717743

[508] . Davidson M, Stevenson M, Hsieh A et al. The burden of familial chylomicronemia syndrome: interim results from the IN-FOCUS study. Expert Rev Cardiovasc Ther. 2017;15:415–423. 10.1080/14779072.2017.1311786 28338353

[509] . Brunzell JD, Deeb SS. Familial lipoprotein lipase deficiency, apo C-II deficiency and hepatic lipase deficiency. In: Scriver CR, Beaudet AL, Sly WS, Valle D, editors. The Metabolic and Molecular Bases of In- herited Disease. 8th ed. New York, NY: McGraw-Hill, 2001. p. 2789–2816.

[510] . Ahmad Z, Halter R, Stevenson M. Building a better understanding of the burden of disease in familial chylomicronemia syndrome. Expert Rev Clin Pharmacol. 2017;10:1–3. 10.1080/17512433.2017.1251839 27771961

[511] . Williams L, Rhodes KS, Karmally W et al, for the patients and families living with FCS. Familial chylomicronemia syndrome: Bringing to life dietary recommendations throughout the life span. J Clin Lipidol. 2018;12:908-919. 10.1016/j.jacl.2018.04.010 29804909

[512] . Yuan G, Al-Shali KZ, Hegele RA. Hypertriglyceridemia: its etiology, effects and treatment. CMAJ. 2007;176:1113–1120. 10.1503/cmaj.060963 PMC1839776 17420495

[513] . Connor WE, DeFrancesco CA, Connor SL. N-3 fatty acids from fish oil. Effects on plasma lipoproteins and hypertriglyceridemic patients. Ann NY Acad Sci. 1993;683:16–34. 10.1111/j.1749-6632.1993.tb35689.x 8352438

[514] . Pschierer V, Richter WO, Schwandt P. Primary chylomicronemia in patients with severe familial hypertriglyceridemia responds to long- term treatment with (n-3) fatty acids. J Nutr. 1995;125:1490–1494. 10.1093/jn/125.6.1490 7782902

[515] . Arnett DK, Blumenthal RS, Albert MA et al. 2019 ACC/AHA Guideline on the Primary Prevention of Cardiovascular Disease: A Report of the American College of Cardiology/American Heart Association Task Force on Clinical Practice Guidelines. Circulation. 2019; 140(11):e596-e646 10.1161/CIR.0000000000000678 PMC7734661 30879355

[516] . Lloyd-Jones DM, Morris PB, Ballantyne CM et al. 2017 Focused Update of the 2016 ACC Expert Consensus Decision Pathway on the Role of Non-Statin Therapies for LDL-Cholesterol Lowering in the Management of Atherosclerotic Cardiovascular Disease Risk: A Report of the American College of Cardiology Task Force on Expert Consensus Decision Pathways. J Am Coll Cardiol. 2017; 70(14):1785-822. 10.1016/j.jacc.2017.07.745 28886926

[517] . Nishida C, Uauy R. WHO Scientific Update on health consequences of trans fatty acids: introduction. Eur J Clin Nutr. 2009; 63(Suppl 2):S1-4. 10.1038/ejcn.2009.13 19424215

[518] . Sacks FM, Lichtenstein AH, Wu JHY et al. Dietary Fats and Cardiovascular Disease: A Presidential Advisory From the American Heart Association. Circulation. 2017; 136(3):e1-23. 10.1161/CIR.0000000000000510 28620111

[519] . Agência Nacional de Vigilância Sanitária (Anvisa). RDC Nº 360, de 23 de dezembro de 2003. Regulamento técnico sobre rotulagem nutricional de alimentos embalados. Disponível em: http://portal.anvisa.gov.br/documents/33880/2568070/res0360_23_12_2003.pdf/5d4fc713-9c66-4512-b3c1-afee57e7d9bc. Acesso em 11 de janeiro de 2019.

[520] . Pinto ALD, Miranda TLS, Ferraz VP et al. Determinação e verificação de como a gordura trans é notificada nos rótulos de alimentos, em especial naqueles expressos “0% gordura trans”. Braz. J. Food Technol. 2016 May;19:e2015043.

