# Supplementary material for: Posicionamento sobre o Consumo de Gorduras e Saúde Cardiovascular – 2021
Source: Arq Bras Cardiol. 2021 Jan 27;116(1):160–212. [Article in Portuguese] doi: 10.36660/abc.20201340 (PMC8159504; doi:10.36660/abc.20201340)
Supplement: Supplementary file 1 [file material-suplementar-posicionamento-consumo-de-gorduras-versao-portugues.pdf]

Material Suplementar

Tabela S1 – Tabela nutricional com quantidade de ácidos graxos e colesterol dos alimentos. Composição de alimentos por 100 g de parte comestível: ácidos graxos e colesterol

Alimento	Total	Saturados (g/100 g)					Monoinsaturados (g/100 g)		Poli-insaturados (g/100 g)					*Trans* (g/100 g)	Colesterol (mg)
		Total	Láurico 12:0	Mirístico 14:0	Palmítico 16:0	Esteárico 18:0	Total	Oleico 18:1	Total	ALA 18:3	EPA 20:5	DHA 22:6	Linoleico 18:2	Elaídico 18:1t	
Banha de porco	100	39,2	0,2	1,3	23,8	13,5	45,1	41,2	11,2	1,0	0	0	10,2	0	95
Manteiga com sal	82,4	49,2	2,09	8,06	23,01	9,30	20,4	17,94	1,2	0,27	0	0	0,89	2,50	201
Manteiga sem sal	86	51,5	2,11	7,96	23,87	9,64	21,9	19,80	1,5	0,27	0	0	1,22	2,31	214
Margarina com óleo hidrogenado com sal (65% de lípides)	67,4	14,9	0,06	0,11	8,29	5,75	18,2	17,87	21,4	1,74	0	0	19,48	8,69	NA
Margarina com óleo interesterificado com sal (65% de lípides)	67,2	21,9	2,50	1,00	12,91	4,35	15,0	14,70	27,6	2,64	0	0	24,85	0,09	NA
Margarina com óleo interesterificado sem sal (65% de lípides)	67,1	20,9	2,35	0,94	12,41	4,15	14,4	14,07	26,5	2,58	0	0	23,79	0,12	NA
Manteiga de cacau	100	59,7	0	0,1	25,5	33,2	32,9	32,6	3	0,1	0	0	2,8	0	0
Óleo de abacate	100	11,5	0	0	10,9	0,66	70,5	67,88	13,48	0,95	0	0	12,53	0	0
Óleo de algodão	100	25,9	0	0,8	22,7	2,3	17,8	17,0	51,9	0,2	0	0	51,5	0	0
Óleo de gergelim	100	14,2	0		8,9	4,8	39,7	39,3	41,7	0,3	0	0	41,3	0	0
Óleo de canola	100	7,9	0	0,06	4,59	2,21	62,6	61,14	28,4	6,78	0	0	20,87	0	NA
Óleo de coco	99	82,4	41,8	16,6	8,63	2,5	6,3	6,25	1,7	0,019	0	0	1,67	0,02	0
Óleo de girassol	100	10,8	0	0,07	6,10	3,42	25,4	25,15	62,6	0,39	0	0	62,22	0	NA
Óleo de milho	100	15,2	0		12,12	2,18	33,4	33,04	50,9	0,96	0	0	49,44	0	NA
Óleo de soja	100	15,2	0	0,08	10,83	3,36	23,3	22,98	60,0	5,72	0	0	53,85	0	NA
Abadejo (filé) congelado cru	0,4	0,1	tr.	tr.	0,06	0,02	tr.	0,04	0,1		0,01	0,08		0	31
Atum (conserva em óleo)	6,0	1,0	0	0,02	0,70	0,26	1,3	1,27	3,2	0,29	0,03	0,19	2,68	0	53
Atum fresco cru	0,9	0,5	0	0,01	0,27	0,17	0,2	0,18	tr.	0,01	tr.	0,01	0,01	tr.	48
Bacalhau salgado cru	1,3	0,6	tr.	0,03	0,43	0,14	0,3	0,28	0,2	0,08	0,02	0,06	0,02	tr.	139
Cação (posta) crua	0,8	0,1	tr.	tr.	0,07	0,07	0,1	0,06	0,2		0,02	0,10	tr.	0	36

Camarão de água salgada cru	0,1	0,1	0	tr.	0,08	0,04	0,1	0,06	0,2	tr.	0,08	0,02	0	tr.	124
Merluza (filé) crua	2,0	0,9	0	0,17	0,59	0,08	0,5	0,03	0,4	0,05	0,11	0	0,03	0	57
Pescada branca crua	4,6	0,8	0	0,04	0,40	0,22	2,4	1,61	0,9	0,04	0,18	0,43	0,03	0,01	51
Pintado cru	1,3	0,6	0	0,03	0,40	0,18	0,4	0,32	0,1	0,02	0,01	0,01	0,02	tr.	50
Salmão, sem pele, fresco, cru	9,7	2,5	0,01	0,3	1,39	0,49	2,9	2,26	3,1	0,03	0,43	0,46	1,73	0	53
Sardinha (conserva em óleo)	24,0	4,1	0	0,32	2,66	0,84	5,5	5,03	11,9	0,99	0,44	0,46	9,78	0	73
Sardinha inteira crua	2,7	1,7	tr.	0,21	1,00	0,27	0,5	0,28	0,2	0,02	0,03	0,06	0,03	tr.	61
Tucunaré (filé) congelado cru	1,2	0,6	tr.	0,03	0,37	0,17	0,4	0,26	0,4	0,02	0	0,12	0,09	0,01	47
Contrafilé com gordura cru	15,0	6,9	0,01	0,53	3,82	2,03	6,2	5,33	0,1	0,05	0	0	0,15	0,28	73
Contrafilé sem gordura cru	4,3	1,9	0	0,12	1,05	0,58	1,9	1,66	0,1	tr.	0	0	0,07	0,06	59
Coxão duro sem gordura cru	6,2	3,0	0	0,21	1,66	0,98	2,4	2,12	0,1	0,01	0	0	0,05	0,01	60
Coxão mole sem gordura cru	8,7	3,9	0	0,29	2,34	1,06	3,7	3,23	0,1	0,01	0	0	0,08	0,12	84
Cupim cru	15,3	6,8	0,01	0,46	3,80	2,06	6,4	5,56	0,2	0,06	0	0	0,15	0,30	51
Fígado cru	5,4	3,0	tr.	0,18	1,28	1,44	1,5	1,43	0,1	tr.	0	0	0,05	0,16	393
Filé mignon sem gordura cru	5,6	2,9	0	0,17	1,44	1,16	1,9	1,69	0,2	0,02	0,01		0,11	0,10	55
Lagarto cru	5,2	2,3	0	0,14	1,36	0,66	2,3	1,98	0,1	0,01	0	0	0,06	0,08	56
Maminha crua	7,0	3,1	0,01	0,21	1,71	0,93	3,1	2,70	0,1	0,01	0	0	0,09	0,08	51
Miolo de alcatra sem gordura crua	7,8	3,4	0	0,20	1,79	1,10	3,3	2,85	0,1	0,04	0,01	0		0,16	60
Músculo sem gordura cru	5,5	2,2	0	0,12	1,27	0,71	2,6	2,35	0,1	0,01	0	0	0,07	0,08	51
Patinho sem gordura cru	4,5	2,0	0	0,11	1,08	0,66	1,9	1,65	0,2	0,02	0	0	0,1	0,07	56
Picanha com gordura crua	14,7	6,1	0	0,42	3,46	1,83	6,7	5,86	0,3	0,05	0	0	0,22	0,22	* análise em reavaliação
Picanha sem gordura crua	4,7	2,0	tr.	0,15	1,18	0,61	2,1	1,83	0,1	tr.	0	0	0,08	0,04	75

Posicionamento

Asa de frango com pele crua	15,1	4,4	0	0,09	3,36	0,93	6,6	5,75	3,0	0,01	0	0	2,96	0,03	113
Coração de frango cru	18,6	4,9	0	0,12	3,49	1,16	6,3	5,56	3,4	0,13	0	0	3,15	0,09	159
Coxa de frango com pele crua	9,8	3,0	0	0,05	2,24	0,68	4,1	3,61	2,2	0,09	0	0	2	0,04	97
Coxa de frango sem pele crua	4,9	1,6	0	0,03	1,19	0,40	2,1	1,82	0,8	0,02	0	0	0,8	0,01	91
Fígado de frango cru	3,5	1,3	tr.	0,02	0,69	0,58	0,7	0,58	0,6	0,01	0	0,02	0,38	0,01	341
Peito de frango com pele cru	6,7	2,2	0	0,06	1,66	0,46	3,2	2,75	0,9	0,03	0	0	0,8	0,03	80
Peito de frango sem pele cru	3,0	1,1	0	0,03	0,79	0,25	1,3	1,16	tr.	0,01	0	0	tr.	0,01	59
Sobrecoxa de frango com pele crua	20,9	6,5	0	0,12	4,96	1,31	9,6	8,44	3,6	0,12	0	0	3,41	0,06	88
Sobrecoxa de frango sem pele crua	9,6	3,0		0,06	2,30	0,61	4,5	3,87	1,6	0,04	0	0	1,45	0,03	84
Linguiça de frango crua	17,4	5,2	0,01	0,16	3,59	1,35	7,3	6,70	3,5	0,17	0	0	3,08	0,04	64
Linguiça de porco crua	17,6	4,0	0,01	0,15	2,52	1,24	5,0	4,66	1,7	0,05	0	0	1,48	0,03	53
Peru congelado cru	1,8	0,4	0,01	0,01	0,26	0,14	0,4	0,41	0,7	0,03	0	0	0,63	0	68
Bisteca de porco crua	8,0	3,5	0,01	0,13	2,17	1,12	3,9	3,37	1,2	0,05	0	0	1,12	0	56
Costela de porco crua	19,8	7,4	0,02	0,28	4,59	2,42	8,3	7,64	2,3	0,09	0	0	2,11	0	69
Lombo de porco crua	8,8	3,3	0,01	0,12	2,08	1,00	3,7	3,39	1,0	0,04	0	0	0,88	0	55
Pernil de porco cru	11,1	4,2	0,01	0,16	2,58	1,29	5,0	4,64	1,7	0,06	0	0	1,51	0	59
Toucinho cru	60,3	17,7	0,06	0,75	11,42	5,21	20,1	18,82	10,1	0,68	0	0	9,32	0,21	73
Iogurte natural	3,0	1,8	0,07	0,30	0,91	0,40	0,9	0,83	0,1	0,03	0	tr.	0,06		14
Iogurte natural desnatado	0,3	0,2	0,01	0,03	0,10	0,05	0,1	0,09			0	0	tr.	0,01	3
Leite de vaca desnatado em pó	0,9	0,6	0,02	0,09	0,29	0,12	0,2	0,20	tr.	tr.	0	0	0,03	0,02	25
Leite de vaca integral	*	1,4	0,06	0,25	0,71	0,29	0,7	0,65	0,1	0,02	0	tr.	0,04	0	10
Leite de vaca integral em pó	26,9	16,3	0,58	2,62	8,11	3,48	7,1	6,25	0,5	0,1	0	0	0,41	0,84	85
Queijo minas frescal	20,2	11,4	0,41	1,73	5,78	2,53	5,8	5,14	0,4	0,06	0	0	0,28	0,54	62
Queijo parmesão	33,5	19,7	0,70	3,26	10,07	4,24	8,7	7,67	0,4	0,11	0	0	0,29	1,01	106

Requeijão cremoso	23,4	13,7	0,52	2,26	7,16	2,72	6,4	5,54	0,3	0,07	0	0	0,24	0,55	74
Ricota	8,1	4,5	0,15	0,66	2,28	1,06	2,4	2,16	0,2	0,02	0	0	0,14	0,21	49
Ovo de codorna inteiro cru	12,7	8,9	tr.	0,13	6,39	2,31	12,1	11,01	2,7	0,1	0	0	2,2	0,04	305
Gema cozida (10 minutos)	30,8	9,2	0	0,08	6,56	2,43	12,1	11,29	4,0	0,05	0	0,10	3,25	0,05	1272
Ovo de galinha inteiro cozido (10 minutos)	9,5	2,9	0	0,02	2,07	0,76	3,8	3,51	1,1	0,02	0	0,02	0,94	0,02	397
Ovo de galinha inteiro cru	8,9	2,6	0	0,02	1,87	0,69	3,6	3,33	1,2	0,02	0	0,04	0,88	0	356
Azeitona preta em conserva	20,3	3,5	0	0	2,87	0,47	11,0	10,57	3,0	0,19	0	0	2,77	0	NA
Azeitona verde em conserva	14,2	2,3	0	0	1,93	0,29	8,3	8,07	1,0	0,13	0	0	0,91	0	NA
Chantilly spray com gordura vegetal	27,3	25,9	10,70	3,64	2,63	7,46	0,1	0,05	0,1		0	0	0,08	0	tr.
Leite de coco industrializado	18,4	15,6	8,25	2,99	1,33	0,51	0,9	0,92	0,2		0	0	0,17	0	NA
Maionese industrializada tradicional com ovos	30,5	4,1	0	0,02	2,84	0,37	6,4	6,24	15,4	1,43	0	0	13,86	0	42
Gergelim, semente	50,4	7,8	0	0,03	4,86	2,58	19,9	19,72	22,5	0,16	0	0	22,39	0	NA
Linhaça, semente	32,3	4,2	0	0,03	2,49	1,62	7,1	7,06	25,3	19,81	0	0	5,42	0	NA
Noz crua	59,4	5,6	0		4,26	1,34	8,7	8,66	44,1	8,82	0	0	35,3	0	NA
Castanha-do-pará	63,5	15,3		0,04	0,04	6,14	27,4	27,14	21,0	0,04	0	0	20,97	0	0
Macadâmia	75,77	12,0	0,076	0,65	6,0	2,32	58,8	43,7	1,5	0,20	0	0	1,29	0	0
Avelã	60,7	4,46	0	0	3,09	1,26	45,6	45,4	7,9	0,08	0	0	7,8	0	0
Amendoim	49,24	6,27	0	0,02	5,15	1,10	24,42	23,75	15,55	0,003	0	0	15,55	0	0
Amêndoa	49,93	3,80	0	0,003	3,08	0,70	31,55	31,29	12,32	0,003	0	0	12,32	0	0
Castanha-de-caju	43,85	7,78	0,015	0,015	3,91	3,22	23,79	23,52	7,84	0,06	0	0	7,78	0	0
Pistache	45,32	5,90	0	0,019	5,26	0,47	23,25	22,67	14,38	0,28	0	0	14,09	0	0

Fonte: Núcleo de Estudos e Pesquisas em Alimentação – NEPA/Universidade Estadual de Campinas (UNICAMP). Tabela brasileira de composição de alimentos/NEPA-UNICAMP. Versão II. 2. ed. Campinas, SP: NEPA-UNICAMP, 2006. Disponível em: www.unicamp.br/nepa.[1] USDA Food Composition Databases. United States Department of Agriculture. Agricultural Research Service USDA National Nutrient Database for Standard Reference Legacy Release, April 2018. USDA Branded Food Products Database. Disponível em: https://ndb.nal.usda.gov/ndb/search/list?home=true.[2] ALA: ácido alfalinolênico; DHA: ácido docosahexaenóico; EPA: ácido eicosapentaenoico; NA: não aplicável; tr.: traços.

Referências

1. Universidade Estadual de Campinas – UNICAMP. Tabela brasileira de composição de alimentos – TACO. 4. ed. rev. e ampl. Campinas: UNICAMP/NEPA, 2011. pp. 161. Available at: http://www.unicamp.br/nepa/taco/tabela.

2. USDA Food Composition Databases. United States Department of Agriculture. Agricultural Research Service USDA National Nutrient Database for Standard Reference Legacy Release, April 2018. USDA Branded Food Products Database. Disponível em: https://ndb.nal.usda.gov/ndb/search/list?home=true.
