# Supplementary material for: Posicionamento sobre o Consumo de Gorduras e Saúde Cardiovascular – 2021
Source: Arq Bras Cardiol. 2021 Jan 27;116(1):160–212. [Article in Portuguese] doi: 10.36660/abc.20201340 (PMC8159504; doi:10.36660/abc.20201340)
Supplement: Supplementary file 2 [file material-suplementar-posicionamento-consumo-de-gorduras-versao-ingles.pdf]

SUPPLEMENTARY MATERIAL

Table S1 – Nutritional table with amounts of fatty acids and cholesterol in foods. Food composition per 100 g of edible portion: fatty acids and cholesterol

Food	Total	Saturated fatty acids (g/100 g)					Monounsaturated fatty acids (g/100 g)		Polyunsaturated fatty acids (g/100 g)					Trans fats (g/100 g)	Cholesterol (mg)
		Total	Lauric acid 12:0	Myristic acid 14:0	Palmitic acid 16:0	Stearic acid 18:0	Total	Oleic acid 18:1	Total	ALA 18:3	EPA 20:5	DHA 22:6	Linoleic acid 18:2	Elaidic acid 18:1t	
Lard	100	39.2	0.2	1.3	23.8	13.5	45.1	41.2	11.2	1.0	0	0	10.2	0	95
Salted butter	82.4	49.2	2.09	8.06	23.01	9.30	20.4	17.94	1.2	0.27	0	0	0.89	2.50	201
Unsalted butter	86	51.5	2.11	7.96	23.87	9.64	21.9	19.80	1.5	0.27	0	0	1.22	2.31	214
Salted margarine with hydrogenated oil (65% lipids)	67.4	14.9	0.06	0.11	8.29	5.75	18.2	17.87	21.4	1.74	0	0	19.48	8.69	NA
Salted margarine with interesterified oil (65% lipids)	67.2	21.9	2.50	1.00	12.91	4.35	15.0	14.70	27.6	2.64	0	0	24.85	0.09	NA
Unsalted margarine with interesterified oil (65% lipids)	67.1	20.9	2.35	0.94	12.41	4.15	14.4	14.07	26.5	2.58	0	0	23.79	0.12	NA
Cocoa butter	100	59.7	0	0.1	25.5	33.2	32.9	32.6	3	0.1	0	0	2.8	0	0
Avocado oil	100	11.5	0	0	10.9	0.66	70.5	67.88	13.48	0.95	0	0	12.53	0	0
Cottonseed oil	100	25.9	0	0.8	22.7	2.3	17.8	17.0	51.9	0.2	0	0	51.5	0	0
Sesame oil	100	14.2	0		8.9	4.8	39.7	39.3	41.7	0.3	0	0	41.3	0	0
Canola oil	100	7.9	0	0.06	4.59	2.21	62.6	61.14	28.4	6.78	0	0	20.87	0	NA
Coconut oil	99	82.4	41.8	16.6	8.63	2.5	6.3	6.25	1.7	0.019	0	0	1.67	0.02	0
Sunflower oil	100	10.8	0	0.07	6.10	3.42	25.4	25.15	62.6	0.39	0	0	62.22	0	NA
Corn oil	100	15.2	0		12.12	2.18	33.4	33.04	50.9	0.96	0	0	49.44	0	NA
Soybean oil	100	15.2	0	0.08	10.83	3.36	23.3	22.98	60.0	5.72	0	0	53.85	0	NA
Whiting (fillet)	0.4	0.1	tr.	tr.	0.06	0.02	tr.	0.04	0.1		0.01	0.08		0	31
Canned tuna (in oil)	6.0	1.0	0	0.02	0.70	0.26	1.3	1.27	3.2	0.29	0.03	0.19	2.68	0	53
Raw fresh tuna	0.9	0.5	0	0.01	0.27	0.17	0.2	0.18	tr.	0.01	tr.	0.01	0.01	tr.	48
Raw salted cod	1.3	0.6	tr.	0.03	0.43	0.14	0.3	0.28	0.2	0.08	0.02	0.06	0.02	tr.	139

Raw dogfish (fillet)	0.8	0.1	tr.	tr.	0.07	0.07	0.1	0.06	0.2		0.02	0.10	tr.	0	36
Raw saltwater shrimp	0.1	0.1	0	tr.	0.08	0.04	0.1	0.06	0.2	tr.	0.08	0.02	0	tr.	124
Raw hake (steak)	2.0	0.9	0	0.17	0.59	0.08	0.5	0.03	0.4	0.05	0.11	0	0.03	0	57
Raw smooth weakfish	4.6	0.8	0	0.04	0.40	0.22	2.4	1.61	0.9	0.04	0.18	0.43	0.03	0.01	51
Raw spotted sorubim	1.3	0.6	0	0.03	0.40	0.18	0.4	0.32	0.1	0.02	0.01	0.01	0.02	tr.	50
Raw fresh skinless salmon	9.7	2.5	0.01	0.3	1.39	0.49	2.9	2.26	3.1	0.03	0.43	0.46	1.73	0	53
Canned sardine (in oil)	24.0	4.1	0	0.32	2.66	0.84	5.5	5.03	11.9	0.99	0.44	0.46	9.78	0	73
Raw sardine	2.7	1.7	tr.	0.21	1.00	0.27	0.5	0.28	0.2	0.02	0.03	0.06	0.03	tr.	61
Raw frozen peacock bass (fillet)	1.2	0.6	tr.	0.03	0.37	0.17	0.4	0.26	0.4	0.02	0	0.12	0.09	0.01	47
Raw striploin with fat	15.0	6.9	0.01	0.53	3.82	2.03	6.2	5.33	0.1	0.05	0	0	0.15	0.28	73
Raw fatless striploin	4.3	1.9	0	0.12	1.05	0.58	1.9	1.66	0.1	tr.	0	0	0.07	0.06	59
Raw fatless outside flat	6.2	3.0	0	0.21	1.66	0.98	2.4	2.12	0.1	0.01	0	0	0.05	0.01	60
Raw fatless topside	8.7	3.9	0	0.29	2.34	1.06	3.7	3.23	0.1	0.01	0	0	0.08	0.12	84
Raw hump steak	15.3	6.8	0.01	0.46	3.80	2.06	6.4	5.56	0.2	0.06	0	0	0.15	0.30	51
Raw liver	5.4	3.0	tr.	0.18	1.28	1.44	1.5	1.43	0.1	tr.	0	0	0.05	0.16	393
Raw fatless tenderloin	5.6	2.9	0	0.17	1.44	1.16	1.9	1.69	0.2	0.02	0.01		0.11	0.10	55
Raw eyeround	5.2	2.3	0	0.14	1.36	0.66	2.3	1.98	0.1	0.01	0	0	0.06	0.08	56
Raw rump tail	7.0	3.1	0.01	0.21	1.71	0.93	3.1	2.70	0.1	0.01	0	0	0.09	0.08	51
Raw fatless rump heart	7.8	3.4	0	0.20	1.79	1.10	3.3	2.85	0.1	0.04	0.01	0		0.16	60
Raw fatless shin	5.5	2.2	0	0.12	1.27	0.71	2.6	2.35	0.1	0.01	0	0	0.07	0.08	51
Raw fatless knuckle	4.5	2.0	0	0.11	1.08	0.66	1.9	1.65	0.2	0.02	0	0	0.1	0.07	56
Raw rump cap with fat	14.7	6.1	0	0.42	3.46	1.83	6.7	5.86	0.3	0.05	0	0	0.22	0.22	* analysis under review
Raw fatless rump cap	4.7	2.0	tr.	0.15	1.18	0.61	2.1	1.83	0.1	tr.	0	0	0.08	0.04	75
Raw chicken wings with skin	15.1	4.4	0	0.09	3.36	0.93	6.6	5.75	3.0	0.01	0	0	2.96	0.03	113
Raw chicken heart	18.6	4.9	0	0.12	3.49	1.16	6.3	5.56	3.4	0.13	0	0	3.15	0.09	159
Raw chicken drumstick with skin	9.8	3.0	0	0.05	2.24	0.68	4.1	3.61	2.2	0.09	0	0	2	0.04	97

Raw skinless chicken drumstick	4.9	1.6	0	0.03	1.19	0.40	2.1	1.82	0.8	0.02	0	0	0.8	0.01	91
Raw chicken liver	3.5	1.3	tr.	0.02	0.69	0.58	0.7	0.58	0.6	0.01	0	0.02	0.38	0.01	341
Raw chicken breast with skin	6.7	2.2	0	0.06	1.66	0.46	3.2	2.75	0.9	0.03	0	0	0.8	0.03	80
Raw skinless chicken breast	3.0	1.1	0	0.03	0.79	0.25	1.3	1.16	tr.	0.01	0	0	tr.	0.01	59
Raw chicken thigh with skin	20.9	6.5	0	0.12	4.96	1.31	9.6	8.44	3.6	0.12	0	0	3.41	0.06	88
Raw skinless chicken thigh	9.6	3.0		0.06	2.30	0.61	4.5	3.87	1.6	0.04	0	0	1.45	0.03	84
Raw chicken sausage	17.4	5.2	0.01	0.16	3.59	1.35	7.3	6.70	3.5	0.17	0	0	3.08	0.04	64
Raw pork sausage	17.6	4.0	0.01	0.15	2.52	1.24	5.0	4.66	1.7	0.05	0	0	1.48	0.03	53
Raw frozen turkey	1.8	0.4	0.01	0.01	0.26	0.14	0.4	0.41	0.7	0.03	0	0	0.63	0	68
Raw pork chop	8.0	3.5	0.01	0.13	2.17	1.12	3.9	3.37	1.2	0.05	0	0	1.12	0	56
Raw pork rib	19.8	7.4	0.02	0.28	4.59	2.42	8.3	7.64	2.3	0.09	0	0	2.11	0	69
Raw pork loin	8.8	3.3	0.01	0.12	2.08	1.00	3.7	3.39	1.0	0.04	0	0	0.88	0	55
Raw pork shank	11.1	4.2	0.01	0.16	2.58	1.29	5.0	4.64	1.7	0.06	0	0	1.51	0	59
Raw bacon	60.3	17.7	0.06	0.75	11.42	5.21	20.1	18.82	10.1	0.68	0	0	9.32	0.21	73
Plain yogurt	3.0	1.8	0.07	0.30	0.91	0.40	0.9	0.83	0.1	0.03	0	tr.	0.06		14
Plain skim-milk yogurt	0.3	0.2	0.01	0.03	0.10	0.05	0.1	0.09			0	0	tr.	0.01	3
Skim cow's milk powder	0.9	0.6	0.02	0.09	0.29	0.12	0.2	0.20	tr.	tr.	0	0	0.03	0.02	25
Whole cow's milk	*	1.4	0.06	0.25	0.71	0.29	0.7	0.65	0.1	0.02	0	tr.	0.04	0	10
Whole cow's milk powder	26.9	16.3	0.58	2.62	8.11	3.48	7.1	6.25	0.5	0.1	0	0	0.41	0.84	85
Minas cheese	20.2	11.4	0.41	1.73	5.78	2.53	5.8	5.14	0.4	0.06	0	0	0.28	0.54	62
Parmesan cheese	33.5	19.7	0.70	3.26	10.07	4.24	8.7	7.67	0.4	0.11	0	0	0.29	1.01	106
Brazilian cream cheese	23.4	13.7	0.52	2.26	7.16	2.72	6.4	5.54	0.3	0.07	0	0	0.24	0.55	74
Ricotta cheese	8.1	4.5	0.15	0.66	2.28	1.06	2.4	2.16	0.2	0.02	0	0	0.14	0.21	49
Raw quail egg	12.7	8.9	tr.	0.13	6.39	2.31	12.1	11.01	2.7	0.1	0	0	2.2	0.04	305

Hard-boiled egg yolk (10 minutes)	30.8	9.2	0	0.08	6.56	2.43	12.1	11.29	4.0	0.05	0	0.10	3.25	0.05	1272
Hard-boiled chicken egg (10 minutes)	9.5	2.9	0	0.02	2.07	0.76	3.8	3.51	1.1	0.02	0	0.02	0.94	0.02	397
Raw chicken egg	8.9	2.6	0	0.02	1.87	0.69	3.6	3.33	1.2	0.02	0	0.04	0.88	0	356
Canned black olives	20.3	3.5	0	0	2.87	0.47	11.0	10.57	3.0	0.19	0	0	2.77	0	NA
Canned green olives	14.2	2.3	0	0	1.93	0.29	8.3	8.07	1.0	0.13	0	0	0.91	0	NA
Spray whipped cream with vegetable fat	27.3	25.9	10.70	3.64	2.63	7.46	0.1	0.05	0.1		0	0	0.08	0	tr.
Canned coconut milk	18.4	15.6	8.25	2.99	1.33	0.51	0.9	0.92	0.2		0	0	0.17	0	NA
Commercial mayonnaise made with eggs	30.5	4.1	0	0.02	2.84	0.37	6.4	6.24	15.4	1.43	0	0	13.86	0	42
Sesame seed	50.4	7.8	0	0.03	4.86	2.58	19.9	19.72	22.5	0.16	0	0	22.39	0	NA
Flaxseed	32.3	4.2	0	0.03	2.49	1.62	7.1	7.06	25.3	19.81	0	0	5.42	0	NA
Raw walnut	59.4	5.6	0		4.26	1.34	8.7	8.66	44.1	8.82	0	0	35.3	0	NA
Brazil nut	63.5	15.3		0.04	0.04	6.14	27.4	27.14	21.0	0.04	0	0	20.97	0	0
Macadamia	75.77	12.0	0.076	0.65	6.0	2.32	58.8	43.7	1.5	0.20	0	0	1.29	0	0
Hazelnut	60.7	4.46	0	0	3.09	1.26	45.6	45.4	7.9	0.08	0	0	7.8	0	0
Peanut	49.24	6.27	0	0.02	5.15	1.10	24.42	23.75	15.55	0.003	0	0	15.55	0	0
Almond	49.93	3.80	0	0.003	3.08	0.70	31.55	31.29	12.32	0.003	0	0	12.32	0	0
Cashew nut	43.85	7.78	0.015	0.015	3.91	3.22	23.79	23.52	7.84	0.06	0	0	7.78	0	0
Pistachio	45.32	5.90	0	0.019	5.26	0.47	23.25	22.67	14.38	0.28	0	0	14.09	0	0

Source: Núcleo de Estudos e Pesquisas em Alimentação – NEPA/Universidade Estadual de Campinas (UNICAMP). Tabela brasileira de composição de alimentos/NEPA-UNICAMP. Versão II. 2. ed. Campinas, SP: NEPA-UNICAMP, 2006. Available at: www.unicamp.br/nepa.1 USDA Food Composition Databases. United States Department of Agriculture. Agricultural Research Service USDA National Nutrient Database for Standard Reference Legacy Release, April 2018. USDA Branded Food Products Database. Available at: https://ndb.nal.usda.gov/ndb/search/list?home=true.2 ALA: alpha-linolenic acid; DHA: docosahexaenoic acid; EPA: eicosapentaenoic acid; NA: not applicable; tr.: trace.

References

1. Universidade Estadual de Campinas – UNICAMP. Tabela brasileira de composição de alimentos – TACO. 4. ed. rev. e ampl. Campinas: UNICAMP/NEPA, 2011. pp. 161. Available at: http://www.unicamp.br/nepa/taco/tabela.

2. USDA Food Composition Databases. United States Department of Agriculture. Agricultural Research Service USDA National Nutrient Database for Standard Reference Legacy Release, April 2018. USDA Branded Food Products Database. Disponível em: https://ndb.nal.usda.gov/ndb/search/list?home=true.
