# Supplementary material for: Posicionamento sobre o Consumo de Gorduras e Saúde Cardiovascular – 2021
Source: Arq Bras Cardiol. 2021 Jan 27;116(1):160–212. [Article in Portuguese] doi: 10.36660/abc.20201340 (PMC8159504; doi:10.36660/abc.20201340)
Supplement: Translation

# Translation: Position Statement on Fat Consumption and Cardiovascular Health – 2021

**Authors:** Maria Cristina de Oliveira Izar, Ana Maria Lottenberg, Viviane Zorzanelli Rocha Giraldez, Raul Dias dos Santos, Roberta Marcondes Machado, Adriana Bertolami, Marcelo Heitor Vieira Assad, José Francisco Kerr Saraiva, André Arpad Faludi, Annie Seixas Bello Moreira, Bruno Geloneze, Carlos Daniel Magnoni, Carlos Scherr, Cristiane Kovacs Amaral, Daniel Branco de Araújo, Dennys Esper Corrêa Cintra, Edna Regina Nakandakare, Francisco Antonio Helfenstein Fonseca, Isabela Cardoso Pimentel Mota, José Ernesto dos Santos, Juliana Tieko Kato, Lis Mie Misuzawa Beda, Lis Proença Vieira, Marcelo Chiara Bertolami, Marcelo Macedo Rogero, Maria Silvia Ferrari Lavrador, Miyoko Nakasato, Nagila Raquel Teixeira Damasceno, Renato Jorge Alves, Lara Roberta Soares, Rosana Perim Costa, Valéria Arruda Machado

**Affiliations:** 1 Brasil Universidade Federal de São Paulo (UNIFESP), São Paulo, SP – Brasil; 2 Brasil Hospital Israelita Albert Einstein (HIAE) - Faculdade Israelita de Ciências da Saúde Albert Einstein (FICSAE), São Paulo, SP – Brasil; 3 Brasil Faculdade de Medicina da Universidade de São Paulo, Laboratório de Lípides (LIM10),São Paulo, São Paulo, SP – Brasil; 4 Brasil Instituto do Coração do Hospital das Clínicas da Faculdade de Medicina da Universidade de São Paulo (FMUSP),São Paulo, São Paulo, SP – Brasil; 5 Brasil Instituto Dante Pazzanese de Cardiologia, São Paulo, São Paulo, SP – Brasil; 6 Brasil Instituto Nacional de Cardiologia, Rio de Janeiro, RJ – Brasil; 7 Brasil Sociedade Campineira de Educação e Instrução, Campinas, SP – Brasil; 8 Brasil Universidade do Estado do Rio de Janeiro (UERJ), Rio de Janeiro, RJ – Brasil; 9 Brasil Universidade Estadual de Campinas (UNICAMP), Campinas, SP – Brasil; 10 Brasil Ministério da Saúde, Brasília, DF – Brasil; 11 Brasil Universidade de São Paulo (USP), São Paulo, São Paulo, SP – Brasil; 12 Brasil Centro Universitário Senac, São Paulo, São Paulo, SP – Brasil; 13 Brasil Faculdade de Medicina da Universidade de São Paulo, Liga de Diabetes, São Paulo, São Paulo, SP – Brasil; 14 Brasil Santa Casa de Misericórdia de São Paulo, São Paulo, São Paulo, SP – Brasil; 15 Brasil Núcleo de Alimentação e Nutrição da Sociedade Brasileira de Cardiologia, Rio de Janeiro, RJ – Brasil; 16 Brasil Hospital do Coração (HCor), São Paulo, São Paulo, SP – Brasil

**Table:** 

Position Statement on Fat Consumption and Cardiovascular Health – 2021	
The report below lists declarations of interest as reported to the SBC by the experts during the period of the development of these update, 2020.	
Expert	Type of relationship with industry
Adriana Bertolami	Nothing to be declared
Ana Maria Lottenberg	FINANCIAL DECLARATION A - ECONOMICALLY RELEVANT PAYMENTS OF ANY KIND MADE TO (i) YOU, (ii) YOUR SPOUSE/PARTNER OR ANY OTHER PERSON LIVING WITH YOU, (iii) ANY LEGAL PERSON IN WHICH ANY OF THESE IS EITHER A DIRECT OR INDIRECT CONTROLLING OWNER, BUSINESS PARTNER, SHAREHOLDER OR PARTICIPANT; ANY PAYMENTS RECEIVED FOR LECTURES, LESSONS, TRAINING INSTRUCTION, COMPENSATION, FEES PAID FOR PARTICIPATION IN ADVISORY BOARDS, INVESTIGATIVE BOARDS OR OTHER COMMITTEES, ETC. FROM THE BRAZILIAN OR INTERNATIONAL PHARMACEUTICAL, ORTHOSIS, PROSTHESIS, EQUIPMENT AND IMPLANTS INDUSTRY: - PTC: medicines
Andre Arpad Faludi	Nothing to be declared
Annie Seixas Bello Moreira	Nothing to be declared
Bruno Geloneze	FINANCIAL DECLARATION A - ECONOMICALLY RELEVANT PAYMENTS OF ANY KIND MADE TO (i) YOU, (ii) YOUR SPOUSE/PARTNER OR ANY OTHER PERSON LIVING WITH YOU, (iii) ANY LEGAL PERSON IN WHICH ANY OF THESE IS EITHER A DIRECT OR INDIRECT CONTROLLING OWNER, BUSINESS PARTNER, SHAREHOLDER OR PARTICIPANT; ANY PAYMENTS RECEIVED FOR LECTURES, LESSONS, TRAINING INSTRUCTION, COMPENSATION, FEES PAID FOR PARTICIPATION IN ADVISORY BOARDS, INVESTIGATIVE BOARDS OR OTHER COMMITTEES, ETC. FROM THE BRAZILIAN OR INTERNATIONAL PHARMACEUTICAL, ORTHOSIS, PROSTHESIS, EQUIPMENT AND IMPLANTS INDUSTRY: - Novo Nordisk: diabetes - AstraZeneca: diabetes - MSD: diabetes OTHER RELATIONSHIPS FUNDING OF CONTINUING MEDICAL EDUCATION ACTIVITIES, INCLUDING TRAVEL, ACCOMMODATION AND REGISTRATION IN CONFERENCES AND COURSES, FROM THE BRAZILIAN OR INTERNATIONAL PHARMACEUTICAL, ORTHOSIS, PROSTHESIS, EQUIPMENT AND IMPLANTS INDUSTRY: - Novo Nordisk: diabetes
Carlos Scherr	OTHER RELATIONSHIPS FUNDING OF CONTINUING MEDICAL EDUCATION ACTIVITIES, INCLUDING TRAVEL, ACCOMMODATION AND REGISTRATION IN CONFERENCES AND COURSES, FROM THE BRAZILIAN OR INTERNATIONAL PHARMACEUTICAL, ORTHOSIS, PROSTHESIS, EQUIPMENT AND IMPLANTS INDUSTRY: - Bayer: thrombosis
Cristiane Kovacs Amaral	Nothing to be declared
Daniel Branco de Araújo	FINANCIAL DECLARATION A - ECONOMICALLY RELEVANT PAYMENTS OF ANY KIND MADE TO (i) YOU, (ii) YOUR SPOUSE/PARTNER OR ANY OTHER PERSON LIVING WITH YOU, (iii) ANY LEGAL PERSON IN WHICH ANY OF THESE IS EITHER A DIRECT OR INDIRECT CONTROLLING OWNER, BUSINESS PARTNER, SHAREHOLDER OR PARTICIPANT; ANY PAYMENTS RECEIVED FOR LECTURES, LESSONS, TRAINING INSTRUCTION, COMPENSATION, FEES PAID FOR PARTICIPATION IN ADVISORY BOARDS, INVESTIGATIVE BOARDS OR OTHER COMMITTEES, ETC. FROM THE BRAZILIAN OR INTERNATIONAL PHARMACEUTICAL, ORTHOSIS, PROSTHESIS, EQUIPMENT AND IMPLANTS INDUSTRY: - Novo Nordisk: diabetes - Sanofi: dislipidemia OTHER RELATIONSHIPS FUNDING OF CONTINUING MEDICAL EDUCATION ACTIVITIES, INCLUDING TRAVEL, ACCOMMODATION AND REGISTRATION IN CONFERENCES AND COURSES, FROM THE BRAZILIAN OR INTERNATIONAL PHARMACEUTICAL, ORTHOSIS, PROSTHESIS, EQUIPMENT AND IMPLANTS INDUSTRY: - Sanofi: dislipidemia
Carlos Daniel Magnoni	Nothing to be declared
Dennys Esper Corrêa Cintra	Nothing to be declared
Edna Regina Nakandakare	Nothing to be declared
Francisco Antonio Helfenstein Fonseca	FINANCIAL DECLARATION A - ECONOMICALLY RELEVANT PAYMENTS OF ANY KIND MADE TO (i) YOU, (ii) YOUR SPOUSE/PARTNER OR ANY OTHER PERSON LIVING WITH YOU, (iii) ANY LEGAL PERSON IN WHICH ANY OF THESE IS EITHER A DIRECT OR INDIRECT CONTROLLING OWNER, BUSINESS PARTNER, SHAREHOLDER OR PARTICIPANT; ANY PAYMENTS RECEIVED FOR LECTURES, LESSONS, TRAINING INSTRUCTION, COMPENSATION, FEES PAID FOR PARTICIPATION IN ADVISORY BOARDS, INVESTIGATIVE BOARDS OR OTHER COMMITTEES, ETC. FROM THE BRAZILIAN OR INTERNATIONAL PHARMACEUTICAL, ORTHOSIS, PROSTHESIS, EQUIPMENT AND IMPLANTS INDUSTRY: - Novo Nordisk: antidiabetic - Libbs: lipid-lowering - Ache: lipid-lowering C - PERSONAL RESEARCH FUNDING PAID BY THE BRAZILIAN OR INTERNATIONAL PHARMACEUTICAL, ORTHOSIS, PROSTHESIS, EQUIPMENT AND IMPLANTS INDUSTRY: - AstraZeneca: projeto de iniciativa do investigador, já concluído - Amgen: lipid-lowering - Biolab: vitamins OTHER RELATIONSHIPS FUNDING OF CONTINUING MEDICAL EDUCATION ACTIVITIES, INCLUDING TRAVEL, ACCOMMODATION AND REGISTRATION IN CONFERENCES AND COURSES, FROM THE BRAZILIAN OR INTERNATIONAL PHARMACEUTICAL, ORTHOSIS, PROSTHESIS, EQUIPMENT AND IMPLANTS INDUSTRY: - Bayer: anticoagulant - Sanofi: lipid-lowering - Amgen: lipid-lowering
Isabela Cardoso Pimentel Mota	Nothing to be declared
Jose Ernesto dos Santos	Nothing to be declared
José Francisco Kerr Saraiva	Nothing to be declared
Juliana Tieko Kato	Nothing to be declared
Lis Mie Masuzawa Beda	Nothing to be declared
Lis Proença Vieira	Nothing to be declared
Marcelo Chiara Bertolami	FINANCIAL DECLARATION A - ECONOMICALLY RELEVANT PAYMENTS OF ANY KIND MADE TO (i) YOU, (ii) YOUR SPOUSE/PARTNER OR ANY OTHER PERSON LIVING WITH YOU, (iii) ANY LEGAL PERSON IN WHICH ANY OF THESE IS EITHER A DIRECT OR INDIRECT CONTROLLING OWNER, BUSINESS PARTNER, SHAREHOLDER OR PARTICIPANT; ANY PAYMENTS RECEIVED FOR LECTURES, LESSONS, TRAINING INSTRUCTION, COMPENSATION, FEES PAID FOR PARTICIPATION IN ADVISORY BOARDS, INVESTIGATIVE BOARDS OR OTHER COMMITTEES, ETC. FROM THE BRAZILIAN OR INTERNATIONAL PHARMACEUTICAL, ORTHOSIS, PROSTHESIS, EQUIPMENT AND IMPLANTS INDUSTRY: - Abbott: dislipidemias - fenofibrato - Ache: dislipidemias - fenofibrato - Libbs: dislipidemias - fenofibrato - Merck: dislipidemias - fenofibrato - Novo Nordisk: dislipidemias - fenofibrato - Sanofi: dislipidemias - fenofibrato OTHER RELATIONSHIPS FUNDING OF CONTINUING MEDICAL EDUCATION ACTIVITIES, INCLUDING TRAVEL, ACCOMMODATION AND REGISTRATION IN CONFERENCES AND COURSES, FROM THE BRAZILIAN OR INTERNATIONAL PHARMACEUTICAL, ORTHOSIS, PROSTHESIS, EQUIPMENT AND IMPLANTS INDUSTRY: - Merck: dyslipidemia - statins - Novo Nordisk: dyslipidemia - statins - Sanofi: dyslipidemia - statins
Marcelo Heitor Vieira Assad	FINANCIAL DECLARATION A - ECONOMICALLY RELEVANT PAYMENTS OF ANY KIND MADE TO (i) YOU, (ii) YOUR SPOUSE/PARTNER OR ANY OTHER PERSON LIVING WITH YOU, (iii) ANY LEGAL PERSON IN WHICH ANY OF THESE IS EITHER A DIRECT OR INDIRECT CONTROLLING OWNER, BUSINESS PARTNER, SHAREHOLDER OR PARTICIPANT; ANY PAYMENTS RECEIVED FOR LECTURES, LESSONS, TRAINING INSTRUCTION, COMPENSATION, FEES PAID FOR PARTICIPATION IN ADVISORY BOARDS, INVESTIGATIVE BOARDS OR OTHER COMMITTEES, ETC. FROM THE BRAZILIAN OR INTERNATIONAL PHARMACEUTICAL, ORTHOSIS, PROSTHESIS, EQUIPMENT AND IMPLANTS INDUSTRY: - AstraZeneca: prevenção cardiovascular - Boeringher: diabetes/anticoagulação - Novo Nordisk: diabetes OTHER RELATIONSHIPS FUNDING OF CONTINUING MEDICAL EDUCATION ACTIVITIES, INCLUDING TRAVEL, ACCOMMODATION AND REGISTRATION IN CONFERENCES AND COURSES, FROM THE BRAZILIAN OR INTERNATIONAL PHARMACEUTICAL, ORTHOSIS, PROSTHESIS, EQUIPMENT AND IMPLANTS INDUSTRY: - Boeringher: diabetes - Novo Nordisk: diabetes
Marcelo Macedo Rogero	Nothing to be declared
Maria Cristina de Oliveira Izar	FINANCIAL DECLARATION A - ECONOMICALLY RELEVANT PAYMENTS OF ANY KIND MADE TO (i) YOU, (ii) YOUR SPOUSE/PARTNER OR ANY OTHER PERSON LIVING WITH YOU, (iii) ANY LEGAL PERSON IN WHICH ANY OF THESE IS EITHER A DIRECT OR INDIRECT CONTROLLING OWNER, BUSINESS PARTNER, SHAREHOLDER OR PARTICIPANT; ANY PAYMENTS RECEIVED FOR LECTURES, LESSONS, TRAINING INSTRUCTION, COMPENSATION, FEES PAID FOR PARTICIPATION IN ADVISORY BOARDS, INVESTIGATIVE BOARDS OR OTHER COMMITTEES, ETC. FROM THE BRAZILIAN OR INTERNATIONAL PHARMACEUTICAL, ORTHOSIS, PROSTHESIS, EQUIPMENT AND IMPLANTS INDUSTRY: - Amgen: dyslipidemia - Ache: dyslipidemia - Novartis: dyslipidemia - PTCbio: dyslipidemia B - RESEARCH FUNDING UNDER YOUR DIRECT/PERSONAL RESPONSIBILITY (DIRECTED TO THE DEPARTMENT OR INSTITUTION) FROM THE BRAZILIAN OR INTERNATIONAL PHARMACEUTICAL, ORTHOSIS, PROSTHESIS, EQUIPMENT AND IMPLANTS INDUSTRY: - Amgen: dyslipidemia - PTCbio: dyslipidemia - Novartis: dyslipidemia OTHER RELATIONSHIPS FUNDING OF CONTINUING MEDICAL EDUCATION ACTIVITIES, INCLUDING TRAVEL, ACCOMMODATION AND REGISTRATION IN CONFERENCES AND COURSES, FROM THE BRAZILIAN OR INTERNATIONAL PHARMACEUTICAL, ORTHOSIS, PROSTHESIS, EQUIPMENT AND IMPLANTS INDUSTRY: - Amgen: dyslipidemia - Novo Nordisk: GLP-1RA - PTCbio: dyslipidemia
Maria Silvia Ferrari Lavrador	Nothing to be declared
Miyoko Nakasato	Nothing to be declared
Nagila Raquel Teixeira Damasceno	Nothing to be declared
Raul Dias dos Santos Filho	FINANCIAL DECLARATION A - ECONOMICALLY RELEVANT PAYMENTS OF ANY KIND MADE TO (i) YOU, (ii) YOUR SPOUSE/PARTNER OR ANY OTHER PERSON LIVING WITH YOU, (iii) ANY LEGAL PERSON IN WHICH ANY OF THESE IS EITHER A DIRECT OR INDIRECT CONTROLLING OWNER, BUSINESS PARTNER, SHAREHOLDER OR PARTICIPANT; ANY PAYMENTS RECEIVED FOR LECTURES, LESSONS, TRAINING INSTRUCTION, COMPENSATION, FEES PAID FOR PARTICIPATION IN ADVISORY BOARDS, INVESTIGATIVE BOARDS OR OTHER COMMITTEES, ETC. FROM THE BRAZILIAN OR INTERNATIONAL PHARMACEUTICAL, ORTHOSIS, PROSTHESIS, EQUIPMENT AND IMPLANTS INDUSTRY: Abbott: cardiology Ache: cardiology AstraZeneca: cardiology Amgen: cardiology Novo Nordisk: cardiology Novartis: cardiology PTC Therapeutics: cardiology Sanofi/Regeneron: cardiology B - RESEARCH FUNDING UNDER YOUR DIRECT/PERSONAL RESPONSIBILITY (DIRECTED TO THE DEPARTMENT OR INSTITUTION) FROM THE BRAZILIAN OR INTERNATIONAL PHARMACEUTICAL, ORTHOSIS, PROSTHESIS, EQUIPMENT AND IMPLANTS INDUSTRY: Amgen: cardiology Esperion: cardiology Kowa: cardiology Sanofi/Regeneron: cardiology
Renato Jorge Alves	Nothing to be declared
Roberta Marcondes Machado	Nothing to be declared
Roberta Soares Lara	Nothing to be declared
Rosana Perim Costa	Nothing to be declared
Valéria Arruda Machado	Nothing to be declared
Viviane Zorzanelli Rocha	FINANCIAL DECLARATION A - ECONOMICALLY RELEVANT PAYMENTS OF ANY KIND MADE TO (i) YOU, (ii) YOUR SPOUSE/PARTNER OR ANY OTHER PERSON LIVING WITH YOU, (iii) ANY LEGAL PERSON IN WHICH ANY OF THESE IS EITHER A DIRECT OR INDIRECT CONTROLLING OWNER, BUSINESS PARTNER, SHAREHOLDER OR PARTICIPANT; ANY PAYMENTS RECEIVED FOR LECTURES, LESSONS, TRAINING INSTRUCTION, COMPENSATION, FEES PAID FOR PARTICIPATION IN ADVISORY BOARDS, INVESTIGATIVE BOARDS OR OTHER COMMITTEES, ETC. FROM THE BRAZILIAN OR INTERNATIONAL PHARMACEUTICAL, ORTHOSIS, PROSTHESIS, EQUIPMENT AND IMPLANTS INDUSTRY: - Amgen: evolocumabe - Novo Nordisk: diabetes - AstraZeneca: dapagliflozina OTHER RELATIONSHIPS FUNDING OF CONTINUING MEDICAL EDUCATION ACTIVITIES, INCLUDING TRAVEL, ACCOMMODATION AND REGISTRATION IN CONFERENCES AND COURSES, FROM THE BRAZILIAN OR INTERNATIONAL PHARMACEUTICAL, ORTHOSIS, PROSTHESIS, EQUIPMENT AND IMPLANTS INDUSTRY: - Amgen: evolocumabe

## List of Abbreviations

ABCA1 - ATP binding cassette transporters A1

ABCG1 - ATP binding cassette transporters G1

ACC - American College of Cardiology

Acetyl-CoA - acetyl-coenzyme A

AHA - American Heart Association

Akt - protein kinase B

ALA - alpha-linolenic acid

AMI - acute myocardial infarction

AMPK – AMP-activated protein kinase

APoA-I - apolipoprotein AI

APoB - apolipoprotein B

AST - aspartate transaminase

BMI – body mass index

CAD - coronary artery disease

CE - cholesteryl ester

CETP - cholesteryl ester transfer protein

CHS - Cardiovascular Health Study

CRP - C-reactive protein

CVD - cardiovascular disease

DASH - Dietary Approaches to Stop Hypertension

DHA - docosahexaenoic acid

DIVAS - Dietary Intervention and VAScular function

DPA - docosapentaenoic acid

DRI - Dietary Reference Intakes

EAS - European Atherosclerosis Society

eNOS - endothelial nitric oxide synthase

EPA - eicosapentaenoic acid

EPIC - European Prospective Investigation into Cancer and Nutrition

ER - endoplasmic reticulum

ESC - European Society of Cardiology

FCS - familial chylomicronemia syndrome

FFA - free fatty acid

HbA1c - glycated hemoglobin

HF - heart failure

HMG-CoA - 3-hydroxy-3-methylglutaryl coenzyme A

HOMA-IR - homeostasis model assessment of insulin resistance

HPFS - Health Professionals Follow-up Study

ICAM - intercellular adhesion molecule

IKK - IκB kinase

IL - interleukin

iNOS - inducible nitric oxide synthase

IRS-1 - insulin receptor substrate-1

JACC - Japan Collaborative Cohort Study for Evaluation of Cancer Risk

JNK - c-Jun N-terminal kinase

LOX-1 - oxidized LDL receptor-1

LPL - lipoprotein lipase

LPS - lipopolysaccharide

MCP - monocyte chemoattractant protein

MRI - magnetic resonance imaging

MUFA - monounsaturated fatty acid

NAFLD - nonalcoholic fatty liver disease

NASH - nonalcoholic steatohepatitis

NCD - noncommunicable disease

NF-kB - nuclear factor kappa B

NHANES - National Health and Nutrition Examination Survey

NHS - Nurses’ Health Study

NO - nitric oxide

NYHA - New York Heart Association

ORIGIN - Outcome Reduction with an Initial Glargine Intervention

PGC - peroxisome proliferator-activated receptor gamma coactivator

PGE2 - prostaglandin E2

PKC – protein kinase C

PPARγ-2 - peroxisome proliferator-activated receptor gamma

PREDIMED - *Prevención con Dieta Mediterránea* /Prevention with Mediterranean Diet

PUFA - polyunsaturated fatty acid

PURE - Prospective Urban Rural Epidemiology

ROS - reactive oxygen species

SFA – saturated fatty acid

SBC - *Sociedade Brasileira de Cardiologia* /Brazilian Society of Cardiology

SCD1 - stearoyl-CoA desaturase-1

SCFA - short-chain fatty acid

SMC - smooth muscle cell

SREBP - sterol regulatory element-binding protein

T2D - type 2 diabetes

TC - total cholesterol

TG - triglyceride

TLR - toll-like receptor

TMA - trimethylamine

TMAO - trimethylamine N-oxide

TNF - tumor necrosis factor

UFA - unsaturated fatty acid

VCAM - vascular cell adhesion molecule

WHI - Women’s Health Initiative

WHO - World Health Organization

ω3 - omega-3

ω6 - omega-6

ω9 - omega-9

## Definition of Grades of Recommendation and Levels of Evidence

### Classes (grades) of recommendation:

Class I: conditions for which there is conclusive evidence, or, in the absence of conclusive evidence, there is general agreement that a given procedure is safe and useful/effective.

Class II: conditions for which there is conflicting evidence and/or a divergence of opinion about the safety and usefulness/efficacy of a procedure.

Class IIA: weight of evidence/opinion is in favor of the procedure; the majority agrees.

Class IIB: the safety and usefulness/efficacy are less well established, with no prevailing opinions in favor of the procedure.

Class III: conditions for which there is evidence and/or general agreement that the procedure is not useful/effective and in some cases may be harmful.

### Levels of evidence:

Level A: data were derived from multiple randomized clinical trials that involved large numbers of patients with similar outcomes and/or robust meta-analyses of randomized clinical trials.

Level B: data were derived from less robust meta-analyses, a single randomized clinical trial, or non-randomized (observational) studies.

Level C: data were derived from consensus of expert opinion.

### Cover Letter

Nutrition plays a key role in the genesis of noncommunicable diseases, which are currently considered one of the most important public health problems worldwide. The quality and quantity of food, in particular dietary sources of fats, can influence both the pathogenesis and prevention of cardiovascular diseases (CVDs). Experts all over the world have developed evidence-based guidelines on fat consumption and suggested an adequate amount of dietary fat, as well as limited consumption of saturated and trans fats. Priority has been given to assessing and proposing healthier eating patterns instead of valuing individual foods, with a much more rational approach to cardiovascular prevention by ensuring an adequate energy intake with the dietary inclusion of grains, fruits and vegetables, restriction of refined carbohydrates and ultra-processed foods, and intake of healthier fats rather than saturated and trans fats.

This position statement aims to guide health professionals in understanding the effects of different fatty acids and to propose appropriate dietary measures targeted at CVD prevention and control.

The Department of Atherosclerosis of the Brazilian Society of Cardiology brought together the country’s leading experts to prepare this document in a clear and objective manner in order to provide the best information available to improve clinical practice in our country for the prevention and treatment of CVD.

Yours sincerely,

Prof. Maria Cristina de Oliveira Izar, PhD

## Content

1. Introduction 169

2. Fatty Acid Classification and Sources 170

2.1. Monounsaturated Fatty Acids 170

2.2. Polyunsaturated Fatty Acids 171

2.3. Saturated Fatty Acids 171

2.4. Trans Fats 171

3. Plasma Concentration of Total Cholesterol and Lipoproteins 171

4. Plasma Concentration of Triglycerides 172

5. Cardiovascular and Coronary Heart Disease 173

5.1. Saturated Fatty Acids 173

5.2. Replacement of Saturated with Unsaturated Fatty Acids 174

5.3. Replacement of Saturated Fatty Acids with Carbohydrates 174

5.4. Polyunsaturated Fatty Acids (Omega-6) 174

5.5. Polyunsaturated Fatty Acids (Marine Omega-3) 175

**5.5.1. Effects on Peripheral Vascular Disease** 176

**5.5.2. Effects on Cardiac Arrhythmia and Sudden Cardiac Death** 176

**5.5.3. Effects on Heart Failure** 176

5.6. Polyunsaturated Fatty Acids (Vegetable Omega-3) 176

5.7. Trans Fats 177

6. Endothelial Dysfunction 177

6.1. Blood Pressure 178

6.2. Stroke 178

7. Inflammation 179

8. Insulin Resistance and Diabetes 180

9. Fatty Liver Disease 181

9.1. Hepatic Steatosis 181

9.2. Saturated Fatty Acids and Nonalcoholic Steatohepatitis 182

9.3. Unsaturated Fatty Acids and Nonalcoholic Steatohepatitis 182

9.4. Trans Fatty Acids and Nonalcoholic Steatohepatitis 183

10. Lipid Metabolism in Adipose Tissue 183

10.1. Saturated Fatty Acids and Adipose Tissue Metabolism 184

10.2. Unsaturated Fatty Acids and Adipose Tissue Metabolism 184

11. Food 185

11.1. Coconut Oil 185

11.2. Palm Oil 185

11.3. Chocolate 186

11.4. Butter 186

11.5. Dairy 186

11.6. Meat 186

12. Gut Microbiota 187

12.1. Dietary Patterns and Gut Microbiota 187

12.2. Importance of Dietary Pattern in Short-chain Fatty Acid Synthesis 187

13. Dietary Cholesterol 188

13.1. Plasma Concentration of Lipids and Lipoproteins 188

13.2. Risk of Developing Type 2 Diabetes 188

13.3. Risk of Cardiovascular Diseases in Type 2 Diabetes 188

13.4. Impact on Cardiovascular Diseases 189

14. Egg 189

14.1. Trimethylamine N-oxide in Cardiovascular Diseases 189

14.2. Hepatic Steatosis 190

15. Interesterified Fats 190

15.1. Studies in Animals 190

15.2. Studies in Humans 191

16. Medium-chain Triglycerides 191

17. Familial Chylomicronemia Syndrome 191

18. Practical Aspects of Nutritional Intervention 192

19. Labeling and Trans Fatty Acids 192

20. Final Considerations 192

21. Nutritional Amounts of Fatty Acids and Cholesterol in Foods 193

22. Grade of Recommendations and Level of Evidence: Fatty Acids and Cardiovascular Disease 196

References 196

## 1. Introduction

The relevance of diet in the genesis of noncommunicable diseases (NCDs) is well documented in the literature.^1^This set of diseases is currently considered one of the most important public health problems and accounts for approximately 71% of mortality worldwide.^2^In Brazil, in 2016, NCDs were associated with 74% of total deaths, especially cardiovascular diseases (CVDs).^3^The quality and quantity of food, in particular dietary sources of fats, can influence both the pathogenesis and prevention of CVD.

Guidelines and statements on fat consumption have been developed for over 50 years, first published by the American Heart Association (AHA).^4^In the last decades, government agencies and international medical societies, such as the World Health Organization (WHO), United States Government, Institute of Medicine, and European Food Safety Authority, among others, have been engaged in the development of scientific reports based on high-quality evidence.^5^In Brazil, the first guideline on fat consumption was published in 2013 by the Brazilian Society of Cardiology (SBC).^6^

The first studies, published in the 1950s, showed that increased fat intake was significantly associated with an increased prevalence of atherosclerosis.^4^Preliminary studies were based on the analysis of population-based data obtained from dietary surveys, which evaluated the effects of the amount and types of saturated (SFA) and unsaturated (UFA) fatty acids on mortality and CVD. Therefore, the first recommendation regarding fat consumption established a maximum limit of 30% of total energy intake from fat and recommended a reduction in the intake of SFAs.^4^Subsequent guidelines published by the AHA^5^and the 2015-2020 Dietary Guidelines for Americans^7^followed the same line of recommendation for CVD prevention, establishing a maximum limit of 35% of energy from fat, varying according to the lipid profile of each individual. In addition, recommendations included a maximum SFA intake of 10% of energy, promotion of UFA intake, and exclusion of trans fatty acids from the diet.

Thus, the AHA recommendation of a low-fat diet has, in fact, the aim to suggest an adequate amount of fat intake. This recommendation was based on the very high intake of fat by the American population (36-46% of energy), which was associated with increased cardiovascular risk. In addition, only for hypercholesterolemic individuals, the American College of Cardiology (ACC) and AHA^8 , 9^recommend a limit of 5 to 6% of calories from SFAs. Likewise, the 2019 European Society of Cardiology (ESC)/European Atherosclerosis Society (EAS) guidelines for the management of dyslipidemias: lipid modification to reduce cardiovascular risk^10^recommend limiting the intake of SFAs (<7% of energy) and total fat (<35% of energy).

Controversial results are common in the field of nutrition research, due to inconsistency in protocols regarding study period, study population, sample size, and type of nutrient used in the comparison group.^10^The replacement of calories from SFAs with polyunsaturated (PUFA) and monounsaturated (MUFA) fatty acids reduces cardiovascular risk,^11^whereas the replacement of SFAs with refined carbohydrates, such as sugar, elicits the opposite effect.^12^In addition, SFAs can be found in a wide variety of foods, with different structure and composition, such as meat, milk, oils, and processed foods. The fact that SFAs are present in fat emulsions, such as milk, and in solid matrices of palm oil with sugar, such as store-bought cookies, induce different effects on plasma lipids.^13^

Another important factor that can interfere in the analysis of the role of fatty acids is the dietary pattern in which they are consumed. From a cardiovascular point of view, SFAs can be associated with deleterious effects when consumed in a context of a diet high in sugar and low in fiber, whereas their negative impact may be attenuated in a context of healthy eating patterns.^14^

Although, in general, SFAs are associated with increased cardiovascular risk, it is important to note that not all SFAs increase plasma cholesterol levels and cardiovascular risk.^11^In addition, several studies have shown that increased SFA intake can induce an increase in HDLc.^11^However, this information should be interpreted with caution, because these HDL particles are enriched with pro-inflammatory proteins, which may reduce their functionality and affect some stage of reverse cholesterol transport.^15^

International guidelines point out the importance of following healthy eating patterns, such as a Mediterranean diet^16 - 18^and the Dietary Approaches to Stop Hypertension (DASH) diet.^19^The Dietary Guidelines for the Brazilian Population^20^also highlight the importance of following healthy eating patterns and emphasize that the study of isolated nutrients does not fully clarify the influence of diet on health. These guidelines also explain that benefits should be attributed less to an individual food and more to the combination of foods that characterize the dietary pattern.

As a common point, all these guidelines emphasize the importance of an adequate energy intake, inclusion of grains, fruits, and vegetables in the diet, and reduction of refined carbohydrates, especially sugars. Regarding fat intake, priority should be given to the consumption of MUFAs and PUFAs, with limited intake of SFAs, which is consistent with the AHA guidelines^9^on the recommended healthy profile of fat intake.

According to recent data from the National Health and Nutrition Examination Survey (NHANES) study, there has been a reduction in the intake of refined carbohydrates and SFAs by the American population. Nevertheless, this population still exceeds the recommended amount of these nutrients.^21^

In Brazil, no study has provided sufficient data for a detailed over-time analysis of the percentage consumption of fats. However, important results from the 2019 Brazilian Household Budget Survey (POF/IBGE),^22^which compared the period from 2017-2018 to 2002-2003, showed a significant decrease in household expenses with oils and fats. In addition, this survey showed a reduction in the intake of legumes (grains). The survey also showed that almost one-third of the population eats out, which increases the likelihood of eating in snack bars, where people often choose foods of low nutritional quality, that is, with a low content of fibers and vitamins and a high concentration of fats and refined carbohydrates. Although there was a small increase in household expenses with fruits, data from the 2018 Brazilian Telephone Survey for Surveillance of Risk and Protective Factors for Chronic Diseases (VIGITEL) show that only 24.4% of the population consumes fruits and vegetables within the amounts recommended by the Brazilian Ministry of Health^3^and that 32% of the population eats high-fat meat daily. Moreover, ultra-processed foods that have low nutritional value, such as sandwich cookies, are those that most contribute to the consumption of SFAs and sugar.^22^

Brazil was one of the 195 countries included in the Global Burden of Disease Study 2017,^1^whose main objective was to evaluate the impact of diet on NCD morbidity and mortality. The main causes of cardiovascular mortality attributable to diet included high intakes of sodium and trans fats and low intakes of fruits, vegetables, whole grains, and foods that are sources of PUFAs. The study also showed that, in Brazil, the main dietary risk factor associated with cardiovascular mortality and morbidity was low intake of grains, which, in our population, are mainly represented by beans. In fact, data collected by both Brazilian surveys, VIGITEL and POF, emphasize that there was a reduction in the consumption of beans, which, in addition to being part of the Brazilian food culture, are part of a healthy dietary pattern due to their low fat content and significant amount of fiber.

Despite the deleterious impact of trans fats on cardiovascular risk, a recent study conducted in Brazil revealed that one-fifth of packaged foods are still prepared with this fatty acid.^23^In addition, other commonly consumed snack foods, such as fried or baked snacks, puff pastry, and pies, among others, are often prepared with trans fats. In this respect, the diet currently consumed by some Brazilians contrasts with current international recommendations on healthy eating.

The present position statement developed by SBC aims to describe recent advances regarding the effects of different fatty acids, ranging from their influence on the gut microbiota, liver lipid metabolism, and adipose tissue to the main aspects related to CVD risk and control.

## 2. Fatty Acid Classification and Sources

### 2.1. Monounsaturated Fatty Acids

MUFAs are characterized by the presence of a single double bond in the carbon chain. Oleic acid (omega-9) is the most abundant MUFA in nature, accounting for 90% of all MUFAs,^24^with olive and canola oils as the main oil sources. MUFAs also play a prominent role in the composition of fatty acids in several nuts, such as macadamia nuts (59%), hazelnuts (46%), peanuts (41%), almonds (31%), cashews (27%), and pistachios (24%).^25^Another oil rich in MUFAs is high oleic acid, which has been used in some countries and can be prepared from sunflower, canola, or soybean oils.^26 , 27^With due attention to the high SFA content, meat products are also considered important sources of MUFAs, accounting in some cases for 40 to 50% of the composition of foods such as beef, chicken,^28^and pork.^29^

### 2.2. Polyunsaturated Fatty Acids

PUFAs are part of a broad group of fats with two or more double bonds in the carbon chain. This characteristic confers widely different biological functions and, therefore, their impact on cardiovascular health is also distinct depending on the type of PUFA consumed. They are part of the omega-6 (ω6) or omega-3 (ω3) series depending on the position of the first double bond counted from the methyl end of the carbon chain. The ω6 fatty acids are classified as linoleic acid (18:2), whose main sources are oils (sunflower, corn, and soybean), walnuts, and Brazil nuts, and arachidonic acid (20:4), obtained from endogenous conversion of linoleic acid. The main ω3 fatty acids are alpha-linolenic acid (ALA [C18:3]) of plant origin, whose main sources are soybean, canola, flaxseed, and chia seeds,^30 , 31^and eicosapentaenoic acid (EPA [C20:5]) and docosahexaenoic acid (DHA [C22:6]), found in fish and cold-water crustaceans from the Pacific and Artic oceans. Linoleic and linolenic fatty acids are considered essential for humans, and must be obtained from food. However, according to the Dietary Reference Intakes (DRI), supplementation is not necessary since a moderate intake of soybean or canola oil (about 15 mL/day) ensures an adequate consumption.^32^EPA and DHA, on the other hand, can be produced endogenously by the enzymatic action of ALA desaturases and elongases, but this conversion is limited and affected by physiological and external factors.^33 - 35^Another source of EPA and DHA is krill oil, a shrimp-like crustacean found in the South Seas. Krill oil is a unique source of EPA and DHA, since most ω3 fatty acids are found in phospholipids, with greater bioavailability of krill ω3 compared to marine ω3.^36^

### 2.3. Saturated Fatty Acids

SFAs have a simple molecular structure and are characterized by the absence of double bonds in the straight carbon chain. They are classified as short-chain (acetic acid [C2:0], propionic acid [C3:0], and butyrate [C4:0]), medium-chain (caproic [C6:0], caprylic [C8:0], and capric [C10:0] acids), and long-chain (lauric [C12:0], myristic [C14:0], palmitic [C16:0], and stearic [C18:0] acids).^37^In addition, they are also classified according to the melting point, a key feature to determine the absorption mechanism. Short- and medium-chain fatty acids (C2-C10), which have a low melting point, are absorbed via the portal system, whereas long-chain fatty acids (C14-C18) are absorbed via the lymphatic system by chylomicrons. Lauric acid is absorbed mostly by chylomicrons, but also via the portal system.^38^

This structural difference allows SFAs to have different biological and metabolic actions,^39^acting as signaling agents to modulate the protein-protein and protein-plasma membrane interactions through processes known as myristoylation and protein palmitoylation.^40^

SFAs can be synthesized endogenously in most cells from acetyl-coenzyme A (acetyl-CoA) derived from the metabolism of carbohydrates, amino acids, and fats.^41^The most abundant source is palmitic acid (meat and palm oil), followed by stearic acid (cocoa), myristic acid (milk and coconut), and, in a small amount, lauric acid (coconut). The main dietary sources of palmitic acid are meat and palm oil.^42 , 43^

### 2.4. Trans Fats

The main dietary source of trans fats is elaidic acid (18:1, n-9t), present in vegetable fats prepared from the partial hydrogenation of vegetable oils, which are widely used in the food industry.^44^Trans fat is also found, in small amounts, in meat and milk in the form of vaccenic acid (18:1, n-11t), which is synthesized by the biohydrogenation of fats under microbial action in ruminant animals.^44^

## 3. Plasma Concentration of Total Cholesterol and Lipoproteins

Reduced SFA intake is recommended because SFAs increase plasma LDLc concentrations.^45^SFA intake has been shown to have a linear correlation with plasma lipid concentrations and to increase total cholesterol (TC), LDLc, and HDLc concentrations, as demonstrated in the WHO study.^11^One of the publications of the Prospective Urban Rural Epidemiology (PURE) study, which investigated the association between diet and plasma lipids in more than 100 000 participants, also revealed an increased plasma concentration of TC, LDLc, and HDLc.^46^The authors also showed a linear association between SFA intake and increased plasma lipids when comparing the highest quintile of intake (>11.2% of energy) to the lowest quintile (<4.03% of energy).

It is important to note that SFAs increase all lipoprotein classes, but the elevation observed in HDL may not be sufficient to overcome the deleterious effects of LDL on cardiovascular risk.^47^The different SFAs exert different effects on the lipid profile and, therefore, on cardiovascular risk. Compared to carbohydrate, myristic acid (C14:0) produces the largest increases in the concentrations of TC and LDLc, followed by palmitic acid (C16:0) and lauric acid (C12:0), an effect not observed with stearic acid.^11^The explanation is that stearic acid is rapidly converted to oleic acid in the liver by stearoyl-CoA desaturase-1 (SCD1).^48^Regarding HDLc, myristic, lauric, and palmitic acids increase HDLc concentrations when isocalorically replacing carbohydrates.^11^

SFAs act on plasma cholesterol by different mechanisms. In 1969, Spritz and Mishkel^49^demonstrated that, due to the straight carbon chain, SFAs can be packed in the core of lipoproteins, allowing them to carry a larger amount of cholesterol.^49^Later, it was demonstrated that SFAs, in combination with cholesterol, are able to reduce LDL receptor activity, protein, and mRNA,^50 , 51^thus impairing LDL clearance.^52 , 53^In addition, SFA intake increases the RNAm of 3-hydroxy-3-methylglutaryl coenzyme A (HMG-CoA) reductase, phosphomevalonate kinase, and lanosterol synthase—important enzymes in the cholesterol synthesis pathway.^54^

A study performed in the cohorts of the Nurses’ Health Study (NHS) (1984-2012) and Health Professionals Follow-up Study (HPFS) (1986-2010) showed that the isocaloric replacement of 1% energy from lauric, palmitic, or stearic acids with PUFAs or MUFAs reduced the risk of coronary heart disease.^55^This effect is associated with the impact of UFAs on plasma lipids, which reduces LDLc concentrations and may also reduce HDLc concentrations.^56^A meta-analysis showed that, for each replacement of 1% energy from SFAs with PUFAs, there is a reduction in plasma concentrations of TC, LDLc, HDLc, apolipoprotein AI (ApoA-I), and apolipoprotein B (ApoB).^56^When SFAs are isocalorically replaced with MUFAs, more modest reductions, although significant, are observed in plasma lipids, including TC, LDLc, HDLc, and ApoB.^11^

A review of observational and intervention studies concluded that the replacement of SFAs with PUFAs reduces LDLc levels and, subsequently, CVD risk.^57^A prospective cohort study involving 84 628 women and 42 908 men showed that the isocaloric replacement of SFAs (5% energy) with complex carbohydrates was associated with an 11% reduction in the risk of coronary heart disease.^58^Conversely, the Women’s Health Initiative (WHI) intervention trial, which investigated the effect of reducing fat intake and increasing vegetable, fruit, and grain intakes on cardiovascular outcomes, reported that the dietary intervention had no effect on reducing cardiovascular risk.^59^However, the intervention had only a mild effect on reducing LDLc levels and decreasing SFA intake (only 2.9% compared to controls). The reduction in total fat intake also reduced MUFA and PUFA intake.^59^Moreover, the isocaloric replacement of SFAs with carbohydrates reduces TC, LDLc, HDLc, ApoA-I, and ApoB.^11^

Two important meta-analyses of clinical trials showed a neutral effect of MUFAs on plasma lipid concentration.^60 , 61^A recent systematic review and regression analysis of intervention studies showed that the replacement of 1% energy from SFAs with an equivalent amount of MUFAs significantly reduced the plasma concentrations of TC, LDLc, and HDLc.^11^Conversely, the isocaloric replacement of carbohydrates with MUFAs increased HDLc levels, an effect that decreases with increasing unsaturation of fatty acids.^62^Also, a diet rich in MUFAs (20% of energy) was shown to reduce the plasma concentration of TC, LDLc, small LDL particles, oxidized LDL, and HDLc.^63^In a study of overweight individuals, increased MUFA intake (from 7 to 13% of energy) also contributed to a reduction in TC and LDLc levels, but with no changes in HDLc.^64^Overall, adequate MUFA intake has shown a positive effect on lipid metabolism, with effects opposite to those of SFAs.

The replacement of 1% energy from SFAs with ω6 was shown to reduce TC by 2 mg/dL, with minimal impact on HDLc.^56^An important meta-analysis of observational epidemiological studies points to the cholesterol-lowering effect of ω6 when replacing SFAs and trans fats in humans.^56^The replacement of 10% energy from SFAs with ω6 was associated with a reduction of 18 mg/dL in LDLc levels, a greater impact than that observed with the isocaloric replacement of carbohydrates. In addition, the high plasma concentration of ω6 was associated with a reduction in the TC/HDLc ratio.^56^

Increased ω6 intake was associated with a small reduction in plasma TC concentration, and only minimal or no effect was observed in HDLc and LDLc concentrations. Therefore, current evidence is insufficient to propose ω6 supplementation for the primary and secondary prevention of CVD.^65^

With regard to ω3 fatty acids, the results of a systematic review showed inconsistent data on the effect of ALA on plasma cholesterol.^66^A meta-analysis of randomized trials found no significant influence of ALA supplementation on TC and LDLc levels, with minimal effect on HDLc (reduction of 0.4 mg/dL).^67^DHA, however, was associated with elevated LDLc,^66^and the same result was observed with fish-oil supplementation.^68^This increase in cholesterol is probably attributable to the decreased expression of sterol regulatory element-binding protein 2 (SREBP-2), which regulates the LDL receptor synthesis,^69 , 70^induced in a dose-dependent manner by DHA.

Another study showed that ALA-rich or EPA/DHA-rich diets did not promote changes in the lipid profile compared to a MUFA-rich diet.^71^A similar result was obtained with oils enriched with EPA, DHA, and ALA.^72^In this study, a beneficial effect on plasma lipids was observed only in the wash-in period, when the participants who had a SFA-rich diet received a MUFA-rich diet.^72^It is important to note that, when analyzing the effects of ω3 fatty acids on cholesterolemia, the type of comparison made in the study should be considered, because UFAs, when used as a substitute in SFA-rich diets, promote beneficial effects; supplementation, however, shows different results.

Trans fatty acids have a greater atherogenic effect, due to their strong impact on cholesterolemia.^73^An important meta-analysis of randomized controlled trials showed the deleterious actions of these fatty acids on the plasma concentrations of TC, LDLc, and VLDLc.^56^Furthermore, trans fatty acids exert an additional adverse effect by reducing plasma HDLc concentrations compared to SFAs.^74 - 77^The reduction in HDLc results from the increased catabolism of ApoA-I.^74 , 75^Also, trans fatty acids increase the activity of cholesteryl ester transfer protein (CETP), a protein involved in the transfer of cholesteryl esters (CEs) and triglycerides (TGs) among plasma lipoproteins, thus enriching ApoB-rich particles with CEs. On the other hand, HDL particles become richer in TGs, favoring their catabolism.^78^Trans fat also acts deleteriously by reducing the clearance of ApoB100-containing particles, thus increasing its concentration in plasma,^75^which contributes to the formation of small, dense LDL particles that are more atherogenic.^79^A meta-analysis of randomized controlled trials showed that, each 1% energy replacement of TRANS fat with SFAs, MUFAs or PUFAs, decreased the total cholesterol/ HDL-C and the ApoB/ApoAI ratio.^80^Therefore, given the recognized negative impact of trans fats on the lipid profile, national and international guidelines recommend their exclusion from the diet.^7 , 8 , 20^

## 4. Plasma Concentration of Triglycerides

Fatty acids act differently on triglyceridemia by modulating transcription factors that participate in the synthesis of lipogenic enzymes involved in fatty acid production.

SFAs are able to modulate genes involved in lipid synthesis. SFAs have been shown to induce the hepatic expression of peroxisome proliferator-activated receptor gamma coactivator 1β (PGC-1β), which in turn activates SREBP, a transcription factor involved in gene transcription of lipogenic enzymes such as acetyl-CoA carboxylase-1 and fatty acid synthase,^81^related to fatty acid synthesis, favoring greater TG production.^54^In addition, SFAs increase SREBP processing and its translocation to the cell nucleus, inducing the transcription of target genes.^82^

A systematic review published by the WHO^11^showed that, for each replacement of 1% energy from SFAs with PUFAs or MUFAs, there was a reduction in plasma TG concentration (0.88 mg/dL and 0.35 mg/dL, respectively). The replacement of SFAs with carbohydrates, however, increased plasma TG concentration by 0.97 mg/dL.^11^Conversely, it is known that PUFAs are involved in the reduction of plasma TG concentration by blocking SREBP, with a more pronounced effect exerted by ω3 fatty acids.^83^

Regarding the action of ALA on triglyceridemia, an experimental study in animals observed a null to mild effect with the use of flaxseed.^84^In humans, a systematic review showed that the TG-lowering effect results from the intake of large amounts of flaxseed oil.^66^A meta-analysis of 14 randomized controlled trials observed no significant effect of ALA supplementation on plasma TG concentrations.^67^Similarly, increased ω6 intake was not associated with decreased plasma TG concentrations.^65^

Clinical studies show that supplementation with 2 to 4 g/day of EPA and DHA can reduce plasma TG concentration by 25 to 30%.^66 , 85 , 86^A 4-week EPA or DHA supplementation in healthy subjects reduced the postprandial concentrations of TG, ApoB48, and ApoB100 (16%, 28%, and 24%, respectively), possibly due to the increased activity of lipoprotein lipase (LPL).^87^

The triglyceride-lowering effect of PUFAs is related to their ability to reduce SREBP1 expression and activity.^81^In animal models and in vitro studies, both EPA and DHA decreased SREBP1, reducing the expression of lipogenic enzymes.^88 , 89 , 90^

The ability of ω3 fatty acids to reduce TGs appears to be dose-dependent, with reductions of about 5 to 10% for each 1 g of EPA/DHA consumed daily, being greater in individuals with higher baseline TG concentrations.^91 - 93^A study of individuals with borderline or high TG values who received 1 to 4 g/day of krill oil for 6 weeks showed a reduction in plasma TG concentrations (18.6 to 19.9 mg/dL). With a supplementation of 0.5 g/day of krill oil, the reduction in TG levels was 13.3 mg/dL.^36^

## 5. Cardiovascular and Coronary Heart Disease

### 5.1. Saturated Fatty Acids

Despite the important biological activities of SFAs, high SFA intake has a deleterious effect on lipid metabolism and cardiovascular risk,^94 , 95^as they increase plasma LDLc concentrations, which is one of the main risk factors for the development of atherosclerosis and, consequently, CVD.^11^A comprehensive systematic review conducted by the Cochrane Library, in 2015, showed that decreased SFA intake was able to reduce cardiovascular events by 17%, compared to usual diet.^96^In addition, in the same meta-analysis subgrouping the studies that replaced SFAs with PUFAs showed a 27% reduction in cardiovascular events. For this reason, nutritional recommendations to reduce cardiovascular risk include reducing SFA intake.

However, in recent years, meta-analyses and observational studies have drawn conflicting conclusions about the relationship between SFA intake and cardiovascular risk.^12 , 94 , 96 , 97 - 99^This discrepancy is due, in part, to the macronutrient used for SFA replacement, since a reduction in one dietary macronutrient leads to an increase in another.^100^Meta-analyses of prospective observational studies assessing the effect of SFAs on the occurrence of cardiovascular events, without considering the type of macronutrient used for SFA replacement, observed no effect of SFA intake on cardiovascular risk.^98 , 101^Conversely, the replacement of SFAs with PUFAs or complex carbohydrates from whole grains proved to be beneficial and was associated with a lower risk of coronary heart disease. The replacement of SFAs with simple carbohydrates, however, had no impact on the risk of cardiovascular events,^97 , 99^since high sugar intake has a detrimental effect on cardiovascular health.

The PURE study, conducted in 18 countries, evaluated the association of dietary components with total mortality and cardiovascular events and showed that the risk of total mortality and non-CVD mortality was positively associated with higher carbohydrate intake and negatively associated with higher intakes of fat (PUFAs, MUFAs, and SFAs) and proteins (% of energy). It is worth noting that the highest fat and SFA intake was 35% and 13% of energy, respectively, and the highest carbohydrate intake median reached 77% of energy. In addition, increased SFA intake was associated with a lower risk of stroke. Total fat intake, as well as SFA and UFA intake, was not associated with myocardial infarction risk or CVD mortality.^102^The type of carbohydrate consumed was not analyzed separately, but it was observed that, in low-income and middle-income countries, people consumed carbohydrates mainly from refined sources. Further analysis showed that total fat and SFA intake correlated with increased plasma concentrations of TC and LDLc.^46^In 2018, in that same cohort, dairy intake was shown to be negatively associated with total and CVD mortality, CVD, and stroke.^103^

Randomized studies have evaluated the effects of dietary interventions on the occurrence of cardiovascular events; however, the differences in total fat intake between the intervention and control groups were not substantial in most studies.^59 , 104 , 105^The WHI trial followed, for about 8 years, 48 835 women who were randomly assigned to either dietary modification (reducing fat intake to 20% of energy and increasing vegetable and grain intakes) or to a control group (guidance through diet-related education materials). After 6 years of follow-up, the dietary intervention did not reduce the occurrence of coronary artery disease (CAD) or stroke, despite the significant reduction in total fat intake.^59^

A prospective cohort study showed that higher SFA intake was associated with a lower risk of ischemic heart disease, but not with the risk of coronary heart disease.^106^In another cohort, the intake of palmitic acid, but not of total SFAs, was positively associated with the risk of CAD.^107^

Recent studies have shown that different types of SFAs have heterogeneous cardiometabolic effects and correlate differently with cardiovascular risk, coronary heart disease, and the incidence of type 2 diabetes (T2D). In this context, lauric, myristic, palmitic, and stearic acids are associated with an increased risk of coronary heart disease^55 , 108^and T2D,^14 , 109^whereas pentadecanoid acid (15:0)^110^and margaric acid (c17:0) are associated with the intake of dairy products, and long-chain SFAs (20:0 to 24:0) correlate inversely with the incidence of CVD and T2D.^14 , 110^

### 5.2. Replacement of Saturated with Unsaturated Fatty Acids

A prospective cohort study that investigated 83 349 women and 42 884 men, from 1986 to 2012, showed that the isocaloric replacement of 5% energy from SFAs with MUFAs or PUFAs was associated with an estimated decrease in total mortality by 13% and 27%, respectively. In addition, the replacement of SFAs with PUFAs reduced the risk of death from CVD, cancer, and neurodegenerative diseases.^93^Intervention studies have shown that the isocaloric replacement of 10% energy from SFAs with PUFAs reduces the risk of cardiovascular events by 27%,^111^and 5% replacement reduces the risk of CAD by 10%.^94^The isocaloric replacement (1% of energy) of SFAs (12:0 to 18:0) with complex carbohydrates reduced the risk of coronary heart disease, as demonstrated in the analysis of the HPFS and NHS studies.^55^

### 5.3. Replacement of Saturated Fatty Acids with Carbohydrates

A prospective cohort study involving 84 628 women and 42 908 men showed that the isocaloric replacement of SFAs (5% energy) with complex carbohydrates was associated with an 11% reduction in the risk of coronary heart disease.^58^Likewise, the isocaloric replacement of only 1% energy in the form of SFAs (12:0 to 18:0) with complex carbohydrates reduced the risk of coronary heart disease.^55^

Conversely, an intervention study evaluating the effect of reducing fat intake and increasing vegetable, fruit, and grain intakes on cardiovascular outcomes observed no effect of diet on reducing cardiovascular risk.^59^However, the intervention had only a mild effect on reducing LDLc levels (2.7 mg/dL) and decreasing SFA intake (only 2.9% compared to controls). It is worth noting that the reduction in total fat intake also reduced MUFA and PUFA intakes, which are associated with a favorable lipid profile from a cardiovascular point of view.^59^

Regarding plasma lipids, isocaloric replacement of SFAs with carbohydrates reduces TC (1.58 mg/dL), LDLc (1.27 mg/dL), HDLc (0.38 mg/dL), ApoA-I (7.0 mg/dL), and ApoB (3.6 mg/dL), whereas it increases TG concentrations (0.97 mg/dL).^11^

With regard to MUFAs, several studies based on a Mediterranean diet have shown positive effects in the prevention of cardiovascular risk factors and outcomes. Olive oil is the main source of MUFAs in the Mediterranean diet, followed by walnuts and chestnuts, which also provide PUFAs. It should be noted that this dietary pattern includes vegetables, fruits, and grains, which are also beneficial for cardiovascular health.^112^

The PREDIMED study followed for 5 years more than 5000 participants at high cardiovascular risk who were assigned to a Mediterranean diet supplemented with extra-virgin olive oil (50 g/day) or mixed nuts (30 g/day), both compared to control participants who consumed a diet with less fat content (30% of energy). The results showed that both intervention groups had fewer cardiovascular events (RR = 0.83).^17^Similar results were also observed with olive oil intake in the NHS study (1980-2010, n = 84 628, HR = 0.85), HPFS study (1986-2010, n = 42 908, HR = 0.85), Alpha-Tocopherol, Beta-Carotene Cancer Prevention Study (RR = 0.92), and European Prospective Investigation into Cancer and Nutrition (EPIC) study (HR = 0.87).^113^One of the arms of the EPIC study, conducted in the Dutch population, showed an increased risk of ischemic heart disease associated with MUFA intake (HR = 1.30).^106^However, it is worth noting that the authors of this study identified important confounding factors that could interfere with the final interpretation of the outcome, as they did not distinguish between cis and trans MUFAs.^106^

A review of studies published by the Cochrane Collaboration showed that the effectiveness of replacing SFAs with MUFAs in cardiovascular events is uncertain due to the small number of studies included.^96^The Dietary Guidelines for Americans,^7^however, state that the replacement of SFAs with MUFAs is associated with reduced cardiovascular risk, although the evidence is not so strong. A later cohort study showed that the replacement of 5% energy from SFAs with MUFAs reduced cardiovascular risk by 15%.^58^

### 5.4. Polyunsaturated Fatty Acids (Omega-6)

Regarding the effects ω6 series of UFAs on cardiovascular risk, randomized controlled trials and observational studies have provided evidence that the replacement of about 5 to 10% energy in the form of SFAs and refined carbohydrates (such as sugar, white bread, white rice) with ω6 reduces the risk of CVD without clinical evidence of adverse events.^114 - 117^The replacement of 1% energy from SFAs with ω6 has been associated with a reduction of 2 to 3% in the incidence of coronary heart disease.^94 , 118^This benefit may even be underestimated due to the large amount of SFAs in some foods that are also sources of ω6.

An important systematic review, which evaluated prospective cohort studies and randomized controlled trials involving individuals in primary and secondary prevention, showed that ω6 intake was not associated with a lower risk of CAD, in contrast to what was observed for fish or marine ω3 intake.^93^In fact, several studies have shown a lower reduction in cardiovascular outcomes with the replacement of SFAs with ω6 than with combined ω6 and ω3.^119^

The Cochrane Collaboration published a review of clinical trials evaluating the effect of ω6 intake on primary CVD prevention and concluded that the intake of ω6 fatty acids (linoleic, gamma-linolenic, dihomo-gamma-linolenic, and arachidonic acids) did not interfere with lipid or blood pressure markers; however, none of the studies assessed clinical outcomes.^65 , 120^In a more recent review, also conducted by the Cochrane Collaboration, which evaluated the effect of ω6 supplementation on risk factors (blood pressure, lipid profile, and adiposity) and cardiovascular outcomes (all-cause mortality, CVD mortality, and cardiovascular events), little or no benefit was observed from ω6 interventions on all-cause mortality (RR = 1.0; 95% CI: 0.88-1.12), CVD mortality (RR = 1.09; 95% CI: 0.76-1.55), and cardiovascular events (RR = 0.97; 95% CI: 0.81-1.15).^65^Likewise, ω6 intake was not associated with a lower risk of cardiac and cerebrovascular events (RR = 0.84; 95% CI: 0.59-1.20) or stroke (RR = 1.36; 95% CI: 0.45-4.11). However, a slight reduction in the risk of acute myocardial infarction (AMI) was observed with increased ω6 intake (RR = 0.88; 95% CI: 0.76-1.02).^65^

Higher plasma concentration of ω6 was associated with lower risk of cardiovascular events, ischemic stroke, and CVD mortality, based on the results of a recent study analyzing data from 30 prospective studies, for a total of 68 659 participants enrolled.^121^In this publication, the authors reinforce the cardiovascular benefits of ω6 intake.

### 5.5. Polyunsaturated Fatty Acids (Marine Omega-3)

EPA and DHA have been investigated for their potential to reduce cardiovascular risk. The mechanisms proposed for cardiovascular benefits include reduced inflammatory markers and platelet aggregation, improved endothelial function, reduced blood pressure, and reduced triglyceridemia.^122 - 124^Marine ω3 fatty acids (DHA and EPA) exert numerous effects on different physiological and metabolic processes, which can influence the likelihood of developing CVD.

Although initial evidence suggests a protective effect of the intake of fish and marine ω3 fatty acids on cardiovascular events, especially in people with established CVD,^125 - 127^recent studies have not shown benefits of ω3 supplementation in people with previous manifestations of atherosclerotic disease.^128 - 130^A possible explanation is related to the characteristics of the population studied, especially regarding the more frequent use of well-known protective agents (e.g., statins, beta-blockers, angiotensin-converting enzyme inhibitors), the more aggressive control of traditional risk factors, and the larger number of revascularization procedures in more recent studies. Therefore, it is questioned whether ω3 fatty acids can bring real additional benefits when patients are treated according to current recommendations. Questions regarding formulation, dose, and duration of supplementation may also be raised. In the Alpha Omega^128^and SU.FOL.OM3 trials,^130^the dose of EPA+DHA (400 to 600 mg/day) may have been insufficient to produce a clinical benefit.

A recent meta-analysis of randomized controlled trials and prospective cohort studies evaluating the association between EPA+DHA intake and CAD risk showed a significant benefit only in populations at higher risk, including those with hypertriglyceridemia. The results of prospective cohort studies showed a significant reduction in the risk of any coronary event with higher intakes of EPA+DHA. Therefore, EPA+DHA intake appears to be associated with a reduced risk of coronary events, with greater benefit in populations at higher risk in randomized controlled studies.^131^

However, different formulations of ω3 and the populations studied seem to contribute to the results. Two recent controlled trials showed conflicting data, but there were differences in the dose and formulation of ω3 used. The ASCEND (A Study of Cardiovascular Events in Diabetes),^132^which evaluated 15 840 patients with diabetes mellitus but without evidence of CVD, showed no significant differences between patients who consumed 1.0 g of EPA+DHA and those who received placebo. A review conducted by the Cochrane Collaboration, which included 79 clinical trials, for a total of 1 120 059 participants enrolled with a 12- to 72-month follow-up, showed that EPA, docosapentaenoic acid (DPA), and DHA had little or no effect on all-cause mortality (RR = 0.98; 95% CI: 0.90-1.03), CVD mortality (RR = 0.95; 95% CI: 0.87-1.03), and cardiovascular events (RR = 0.99; 95% CI: 0.94-1.04).^133^

In the Reduction of Cardiovascular Events with Icosapent Ethyl-Intervention Trial (REDUCE-IT),^134^involving high-risk patients with elevated TG levels receiving statin therapy, the risk of ischemic events, including CVD death, was significantly lower in patients who received 2 g of icosapent-ethyl ester twice daily (total daily dose of 4 g) than in those who received placebo. In a total sample of 8179 patients (70.7% enrolled for secondary prevention) followed for a median of 4.9 years, there was a 25% reduction in the risk of the primary composite endpoint (HR = 0.75; 95% CI: 0.68-0.83; P < 0.001), key secondary endpoint events (HR = 0.74; 95% CI: 0.65-0.83; P < 0.001), and prespecified events, including the rate of CVD death (HR = 0.80; 95% CI: 0.66-0.98; P = 0.03). However, a higher rate of patients in the EPA group were hospitalized for atrial fibrillation or flutter, with no differences in the risk of bleeding. It is worth noting that icosapent ethyl is not a fatty acid found in food, and its indication, in pharmacological doses, is made at the physician’s discretion.

Therefore, although there is a consensus that regular intake of fish rich in ω3 fatty acids should be part of a healthy diet, there is still no safe recommendation for supplementing fish-oil capsules. This occurs because the topic is still surrounded by controversy, fueled by conflicting results from clinical trials.

Using experimental models of atherosclerosis in mice, several studies have reported that fish oil and EPA can attenuate the atherosclerotic process, although the same has not been demonstrated in other experimental conditions.^135 - 140^Some population-based studies suggest an inverse association between fish or marine ω3 fatty acid intake and subclinical atherosclerosis markers, such as carotid intima-media thickness and coronary calcification, although this relationship seems to be subtle.^141 - 143^In a randomized trial of patients with CAD, supplementation with approximately 1.5 g/day of ω3 fatty acids for 2 years resulted in less progression and more regression of coronary atherosclerosis, as assessed by quantitative invasive angiography, compared to placebo, although the differences were small.^144^However, in another study, supplementation did not change the progression of carotid atherosclerosis, as assessed by ultrasound,^145^which disagrees with the results of the randomized trial conducted by Mita et al.,^146^who reported that highly purified EPA (1.8 g/day) attenuated the progression of carotid intima-media thickening in patients with diabetes.^146^

It is also possible that ω3 fatty acids play a protective role against cardiovascular events by modulating atherosclerotic plaque characteristics, making the plaque more stable. A randomized trial of patients awaiting carotid endarterectomy showed that atherosclerotic plaques readily incorporated ω3 fatty acids from fish-oil supplementation, making them less vulnerable to rupture and instability phenomena,^147^an observation consistent with experimental findings.^139^

#### 5.5.1. Effects on Peripheral Vascular Disease

Despite extensive research on the effects of ω3 fatty acids on improving vascular function, their effects on cardiovascular outcomes in individuals with peripheral arterial disease are less described. A meta-analysis of 5 studies with a total of 396 participants, published between 1990 and 2010, was conducted to evaluate this issue.^148 - 152^In patients with peripheral vascular disease, there is insufficient evidence to recommend ω3 fatty acids for the reduction of major cardiovascular events, need for revascularization or amputation, improvement in pain-free walking distance, or improvement in quality of life.^153^

#### 5.5.2. Effects on Cardiac Arrhythmia and Sudden Cardiac Death

Experimental studies have shown antiarrhythmic effects of ω3 fatty acids, mainly attributable to a direct effect on ion channels.^154^Other mechanisms include modulation of the autonomic tone (improved heart rate variability), reduction in basal heart rate, and restriction of reperfusion-induced arrhythmias.^154^These effects may explain the beneficial results of ω3 fatty acids in the prevention of sudden cardiac death reported in some studies.

Several observational studies have suggested that ω3 fatty acids can provide particular protection against sudden cardiac death, especially in patients with AMI. This beneficial effect was also observed in a subanalysis of the GISSI-Prevenzione randomized trial,^155^but not in the most recent randomized trial, OMEGA.^129^This hypothesis was also confirmed in patients with implantable cardioverter defibrillators. The results were inconsistent, ranging from a slight beneficial effect of ω3 fatty acids on the reduction of severe ventricular arrhythmias in this subset of patients^156^to a proarrhythmic effect in some patients.^157^

Due to conflicting results, data from a meta-analysis were evaluated, for a total of 32 919 participants included in 9 studies. Of these, 16 465 patients received ω3 and 16 454 received placebo. There was a non-significant reduction in the risk of sudden cardiac death or ventricular arrhythmias with the use of ω3 fatty acids (OR = 0.82; 95% CI: 0.60-1.21; P = 0.21).^158^

Another review evaluated the results of studies using ω3 fatty acids in ventricular arrhythmias and sudden cardiac death, questioning whether these lipids produce antiarrhythmic, proarrhythmic, or neutral effects, which, in turn, would require randomized controlled trials with a specific design for these populations.^159^

#### 5.5.3. Effects on Heart Failure

A large randomized controlled trial, the GISSI-HF trial, showed a slight reduction in mortality when ω3 (1 g/day) was supplemented in patients with New York Heart Association (NYHA) class II-IV heart failure (HF),^160^which is consistent with other epidemiological and observational studies that suggested an inverse relationship between fish or ω3 intake and HF-related events.^161 , 162^

Recommendations from national and international guidelines consider ω3 supplementation in HF a class IIb indication (level of evidence B) based on data from the GISSI-HF trial,^160^but not from other studies in which ω3 fatty acids have been supplemented.

In the GISSI-HF trial, which included 6975 patients with NYHA class II-IV HF or an ejection fraction <40% or who had been hospitalized in the preceding year for HF, 1 g of ω3 was added to standard therapy. This therapy included angiotensin-converting enzyme inhibitors/angiotensin receptor blockers in 94% of patients, beta blockers in 65%, and spironolactone in 39%. Patients were followed for a median of 3.9 years. Supplementation with ω3 fatty acids reduced by 8% the co-primary endpoint of CVD death or hospitalization: 10% in the relative risk of CVD death and 7% in cardiovascular hospitalizations.^160^

## 5.6. Polyunsaturated Fatty Acids (Vegetable Omega-3)

Although the real impact of vegetable-derived ω3 fatty acids on CVD is still under debate, most prospective observational studies suggest that ALA intake may protect against cardiovascular events.^163^In the HPFS study, the prospective analysis of more than 45 000 men showed that ω3 intake, both of marine and vegetable origin, was associated with a reduction in cardiovascular risk, with little influence of ω6 intake.^164^In the NHS study, which assessed cardiovascular outcomes in more than 76 000 women, ALA intake was inversely associated with the risk of sudden cardiac death, but not with other types of fatal coronary outcomes or non-fatal AMI.^165^

Meta-analyses and systematic reviews have produced conflicting results.^93 , 166 , 167^In the Alpha Omega randomized controlled trial, intake of a margarine supplemented with ALA for 40 months did not reduce the rate of cardiovascular events in patients who had had an AMI.^128^As for the effectiveness of ALA, there was a slight reduction in the risk of cardiovascular events (RR = 0.95; 95% CI: 0.83-1.07), CVD mortality (RR = 0.95; 95% CI: 0.72-1.26), and arrhythmias (RR = 0.79; 95% CI: 0.57-1.10).^133^

The role of the dietary ω6/ω3 ratio in the pathogenesis of cardiovascular, inflammatory, and autoimmune diseases has also been the subject of controversy in recent years. Humans have experienced dramatic changes in their diet regarding fatty acid intake in the last millennia. With the agricultural and industrial revolutions, there was an increase in the intake of cereals, oils, and grains rich in ω6, while the intake of ω3 decreased. The ω6/ω3 ratio, originally from 1:1 to 3:1, currently ranges from 15:1 to 40:1 in the Western diet.^168 , 169^

Most studies have concluded that, for general health promotion, the ω6/ω3 ratio should be lower than that currently observed in the general Western population.^170^Some experts advocate for a reduction in this ratio both by increasing ω3 intake and by reducing ω6 intake. Accordingly, in a prospective randomized secondary prevention trial of post-AMI patients, an experimental Mediterranean diet characterized, among other factors, by being richer in ALA (C18:3 – ω3) and oleic acid (C18:1 – ω9) and poorer in linoleic acid (C18:2 – ω6) was associated with a reduction of up to 70% in overall mortality.^171^The diet included the replacement of corn oil with olive oil, with a consequent decrease in the ω6/ω3 ratio to up to 4:1.^171^

The evidence so far suggests that increased intake of ω3, particularly DHA and EPA, provides protection against CVD. In addition, several experts have questioned the validity of using the ω6/ω3 ratio alone in clinical practice and its relationship with cardiovascular risk.^172 , 173^Both fatty acids, ω6 and ω3, have been associated with beneficial effects on cardiovascular health. However, the importance of the ω6/ω3 ratio is based on the enzymatic competition between ω6 and ω3 due to the action of delta-6 desaturase, which converts both into different subspecies. On the one hand, high ω6 intake can decrease the metabolism of ω3 (ALA – C18:3) to EPA (C20:5) and DHA (C22:6),^174^thus limiting the benefits of ω3 fatty acids. On the other hand, the higher affinity of delta-6 desaturase for ω3 fatty acids may lead the essential metabolites derived from the bioconversion of ω6 not to be produced satisfactorily, which would support a recommendation for a small increase in its intake compared to ω3.^172^

In view of these issues and until further scientific evidence is available to support changes in current approaches, dietary recommendations should be based on the total intake of each fatty acid type (ω6 and ω3), and not only on the ω6/ω3 ratio.

## 5.7. Trans Fats

Several observational studies have associated the intake of trans fatty acids, or foods containing trans fats, with adverse cardiovascular outcomes.^76 , 175 - 180^An analysis of data from the NHS study showed that, for every 2% increase in trans fat intake, there was a 1.93-fold increase in the relative risk of coronary heart disease.^175^Likewise, the replacement of 2% energy from trans fats with UFAs reduced cardiovascular risk by 53%, as shown in the Seven Countries Study population.^181^

The Cardiovascular Health Study (CHS)^182^evaluated the plasma concentration of trans fatty acids (elaidic acid) in 2742 adults and showed that these fatty acids were associated with an increase in total mortality, mainly due to increased cardiovascular risk. A study evaluating the NHS and HPFS studies’ databases also showed that trans fat intake increased total mortality to 13%, when comparing the highest to the lowest quintile of intake.^180^

This deleterious effect of trans fats on cardiovascular risk may be attributable to its action on increasing LDLc and decreasing ATP binding cassette transporters A1 (ABCA1) and G1 (ABCG1), responsible for cholesterol efflux from macrophages to ApoA-I and HDL, respectively.^183^

## 6. Endothelial Dysfunction

Endothelial dysfunction is one of the initial events in the genesis of CVD and results mainly from reduced production and/or availability of nitric oxide (NO) and from an imbalance between endothelium-derived vasodilator and vasoconstrictor factors.^184 , 185^Cardiovascular risk factors, such as oxidized LDL, dyslipidemia, hypertension, hyperglycemia, hyperinsulinemia, and smoking, can induce endothelial activation, which induces increased production of cytokines, chemokines, and reactive oxygen species (ROS), thus reducing the capacity for NO-dependent vasodilation. In addition, there is an increase in endothelial permeability, which facilitates the transport of LDL to the subendothelial layer, where LDL can undergo modifications (by oxidation or glycation) and trigger an inflammatory response. This can lead endothelial cells to express cell adhesion molecules and produce mediators that will promote chemotaxis of inflammatory cells, platelet activation, and smooth muscle cell (SMC) proliferation and migration, thus contributing to the genesis of atherosclerosis.^186 , 187^NO, on the other hand, is able to reduce the expression of inflammatory mediators and endothelial cell adhesion molecules and to decrease vascular reactivity, thus preventing vasoconstriction at the injury site.^188 , 189^

A high-fat diet has been shown to reduce the activation of the endothelial AMPK-PI3K-Akt-eNOS pathway, leading to endothelial dysfunction.^185 , 190 , 191^In experimental animals, consumption of a high-fat diet for 6 weeks increased the plasma concentration of pro-inflammatory cytokines and reduced adiponectin concentrations, while reducing NO production and promoting endothelial dysfunction.^192^

SFAs, especially palmitic acid, activate inflammatory responses and oxidative stress, which impair endothelial integrity and cause endothelial dysfunction. SFAs are able to activate the transcription nuclear factor kappa B (NF-κB), which controls inflammatory signaling and oxidative stress pathways,^193^and, consequently, induce endothelial dysfunction by increasing ROS and secreting pro-inflammatory cytokines, such as interleukin (IL)-6 and tumor necrosis factor (TNF)-α.^194 , 195^

In a study on endothelial cells, palmitic acid inhibited insulin-dependent activation of endothelial NO synthase (eNOS), thereby reducing NO production, an effect mediated by the activation of PTEN (phosphatase and tensin homolog deleted on chromosome 10). Such phosphatase, when activated, reduces protein kinase B (Akt) phosphorylation.^196^In another study, treatment of endothelial cells with palmitic acid decreased NO production by reducing insulin-mediated phosphorylation of insulin receptor substrate-1 (IRS-1), Akt, and eNOS. This effect was dependent on increased palmitic acid-mediated IκB kinase (IKK)-β activation.^197^

SFAs can promote inflammation and endoplasmic reticulum (ER) stress in different cell types.^69 , 193 , 194 , 198 , 199^In cardiac fibroblasts, palmitic acid activated inflammatory pathways and induced mitochondrial dysfunction and ER stress, leading to increased ROS production and inflammasome activation, an effect that was mitigated by the presence of EPA.^198^In SMCs, palmitic acid is able to induce apoptosis through toll-like receptor 4 (TLR4) activation, increased ROS production, and increased caspase 3 and caspase 9 expression.^199^In macrophages, SFAs increase the content of oxidized LDL receptor-1 (LOX-1) with a subsequent increase in the uptake of oxidized LDL, leading to increased ROS production and ER stress, effects that were corrected by adding UFAs to the medium.^193^In endothelial cells, treatment with palmitic acid induced endothelial dysfunction and reduced eNOS and AMPK phosphorylation, with a subsequent reduction in NO production. Also, palmitic acid induced increases in ROS, inducible nitric oxide synthase (iNOS), and apoptosis, actions that were attenuated by concomitant incubation with EPA.^194^.

Habitual consumption of an SFA-rich diet was associated with changes in endothelial function in overweight young adults.^200^However, intervention studies assessing the effect of acute SFA intake on endothelial function have produced controversial results. The Dietary Intervention and VAScular function (DIVAS) study, involving adults with moderate cardiovascular risk, reported that 16-week isocaloric replacement of SFAs with MUFAs or linoleic acid had no effect on endothelial function, inflammatory markers, or insulin resistance. However, there was a reduction in the plasma concentrations of TC, LDLc, and E-selectin.^201^The DIVAS-2 study, which evaluated the acute effect of high-fat meals on endothelial function and cardiovascular risk markers in postmenopausal women, found no difference in the impact of different fatty acids on markers of endothelial function.^202^

SFAs, especially palmitic acid, activate inflammatory responses and oxidative stress, which impair endothelial integrity and cause endothelial dysfunction. Fish-oil supplementation significantly improved endothelial function in forearm resistance vessels.^123^Compared to placebo, systemic vascular compliance improved after 3 g/day of DHA or EPA for 7 weeks.^203^The proposed mechanisms include the incorporation of ω3 into membrane phospholipids, thus changing vascular compliance.^68^Attenuation of age-related vascular stiffness in patients with dyslipidemia and carotid artery distensibility is another proposed mechanism.^204^Endothelial dysfunction is closely associated with vascular wall inflammation. The effects of marine ω3 supplementation on in vivo endothelial function in humans are still controversial. An analysis of 33 intervention trials suggests that marine ω3 fatty acids may improve endothelial function in overweight dyslipidemic patients and in patients with diabetes, although the results are conflicting in patients with CVD and inconsistent in healthy individuals.^63^

A study of endothelial cells showed that elaidic acid can cause cell death by activating the caspase pathway,^205^as well as NF-κB activation by increasing ROS production, resulting in increased vascular cell and intercellular adhesion molecule (VCAM-1 and ICAM-1) expression and greater leukocyte adhesion.^206^Consistent with these results, a study in humans reported an increase in plasma concentrations of E-selectin and C-reactive protein (CRP) with trans fat intake.^207^Increased trans fat intake was also shown to increase the plasma concentrations of E-selectin, VCAM, and ICAM in 730 women who participated in the NHS study.^208^A study on endothelial cells investigating the effect of trans fatty acids on NF-κB activation showed that elaidic acid induced IκB phosphorylation, as assessed by an increase in IL-6 concentrations.^209^It also led to a decrease in both NO production and insulin signaling, and promoted pro-inflammatory signaling and cell death.^210^

### 6.1. Blood Pressure

In dietary intervention studies in overweight patients, those consuming a daily fish meal showed a decrease in systolic and diastolic blood pressure, and this reduction was even greater when combined with a weight loss program, even after adjustment for other covariates.^211^A meta-analysis conducted in the 1990s concluded that the effect of ω3 supplementation on blood pressure is dose-dependent, being effective from a dose of 3.0 g/day, with a reduction of 0.66 and 0.35 mm Hg in systolic and diastolic blood pressure, respectively, per gram of ω3 consumed.^212^

In another meta-analysis of 36 randomized trials, fish-oil supplementation (median dose of 3.7 g/day) reduced systolic blood pressure by 2.1 mm Hg and diastolic blood pressure by 1.6 mm Hg.^213^These modest results can be explained by the low degree of purity and low concentrations in the formulations used. Other studies using low doses (1.6 g of DHA and 0.6 g of EPA) have not shown benefits in blood pressure, possibly because of the low doses used. In high-risk patients, such as those on hemodialysis, 4-month supplementation with 2 g of ω3 was associated with lower systolic (−9 mm Hg) and diastolic (−11 mm Hg) blood pressure, compared to olive oil.^214^

In a meta-analysis involving patients with T2D, ω3 supplementation reduced diastolic pressure by 1.8 mm Hg.^215^Theobald et al.^216^also showed a reduction in blood pressure with the consumption of low doses of ω3.^216^However, when endothelial function or arterial stiffness rates are assessed, data are conflicting between studies.^216 , 217^

Schwingshackl et al.^218^conducted a systematic review and meta-analysis to investigate the impact of MUFAs on lipid metabolism, blood pressure, and cardiovascular events. The results showed that diets with MUFA content above 12% of energy had a beneficial effect only on diastolic and systolic blood pressure.

In addition to the benefits observed in the lipid profile,^219^the Mediterranean dietary pattern also improves blood pressure^220^and provides additional protection against oxidative stress,^221^inflammatory markers,^222^and endothelial dysfunction.^112^In this respect, it is noted that additional health benefits were conferred by other substances, independently of MUFAs. For such substances, there is currently no specific recommended intake.

Therefore, there is little evidence of the protective role of MUFAs against hypertension and endothelial dysfunction that could support specific recommendations.

### 6.2. Stroke

Elevated blood pressure is the main risk factor for stroke. Regarding SFA intake, some studies have observed little or no effect on stroke risk.^12 , 96 , 223 , 224^In the WHI study, which followed women for about 8 years, reduced SFA intake did not reduce the risk of stroke.^59^Conversely, other cohort studies, such as the Japan Collaborative Cohort Study for Evaluation of Cancer Risk (JACC), that followed 58 453 Japanese adults for 14 years,^225^and the PURE study,^102^reported an inverse association between SFA intake and stroke risk.

A meta-analysis found an inverse association between SFA intake and stroke risk only for Asian men with body mass index (BMI) <24 kg/m^2^, indicating that factors such as ethnicity, sex, and body weight influence the association between SFAs and stroke risk.^226^Thus, to date, there is no robust evidence to recommend the reduction of SFAs to prevent the risk of stroke.^96^

In randomized trials, the use of ω3 fatty acids, such as EPA, DHA and DPA (C20:5), reduced risk factors and mechanisms for cardiovascular events, including hypertension, hyperlipidemia, and endothelial dysfunction,^213 , 227 , 228^suggesting their protective role in CVD. However, the impact of these fatty acids on ischemic stroke is still controversial. Observational studies have shown inverse associations between self-reported dietary ω3 intake and ischemic stroke,^229^which were not confirmed in a meta-analysis of randomized trials using ω3 supplementation.^227^However, the meta-analysis data were derived from short-term supplementation studies of high-risk patients who, in general, had previous stroke, in which stroke was not a predetermined outcome. Therefore, it is not possible to generalize these results to populations in primary prevention.^230^In addition, ischemic stroke may be related to atherothrombotic or cardioembolic disease, whose pathophysiological mechanisms are different.^231^DHA can reduce the risk of atherothrombotic stroke by reducing endothelial dysfunction and atherosclerosis, whereas EPA and DPA can have a greater impact on cardioembolic stroke due to their effects on coagulation and atrial fibrillation.^232^Moreover, almost all studies of ω3 intake and stroke risk were based on self-reported dietary intake of these fatty acids, making it impossible to distinguish between the types of fatty acid consumed.

In a systematic review of 3 large US cohorts, the CHS, NHS and HPFS, the circulating levels of fatty acids were measured at baseline to assess their relationship with the incidence of ischemic stroke. Ischemic strokes were prospectively adjudicated and classified into atherothrombotic or cardioembolic, and the risk was calculated according to the circulating levels of fatty acids. Higher circulating levels of DHA were inversely associated with the incidence of atherothrombotic stroke, and DPA, with cardioembolic stroke. There was no association between EPA and stroke. These findings suggest differential benefits according to the ω3 fatty acid involved.^233^

## 7. Inflammation

SFAs are essential components of the lipid A present in the cell wall of Gram-negative bacteria – it is the endotoxic portion of lipopolysaccharide (LPS).^234^It is well documented that SFAs trigger inflammatory signaling, as they modulate both the NF-kB pathway, through the structure of TLR4 receptors,^235^and the TLR2 pathway.^236^Another mechanism that enhances the inflammatory process induced by SFA intake is intracellular NLRP3 inflammasome activation. The activated inflammasome then processes IL-1β and IL-18 into their mature forms, induced by NF-kB. Dietary SFAs have been shown to activate this mechanism via TLR4 receptors,^237^as have prostaglandins E2 (PGE2) derived from arachidonic acid,^238^with important implications for coronary heart disease^239^and comorbidities associated with T2D, such as diabetic retinopathy.^238^

In macrophages, lauric acid^240^showed greater inflammatory capacity, as assessed by the activation of the TLR4 pathway, compared to myristic, palmitic, and stearic acids, whereas MUFAs and PUFAs did not activate this pathway. The pretreatment of cells with different UFAs significantly reduced the pro-inflammatory effect induced by lauric acid.^241 , 242^Also, inhibition of TLR2 expression improved insulin action in muscle cells treated with palmitic acid as well as in skeletal muscle and adipose tissue in mice fed a high-SFA diet.^243^A study of 965 healthy young adults showed a positive association of plasma levels of myristic and palmitic acids with CRP levels, whereas stearic and linoleic acids were inversely associated.^244^

As precursors of eicosanoids and other anti-inflammatory mediators, ω3 fatty acids can exert anti-inflammatory effects, with benefits in several pathological conditions, including CVD. Many experimental studies have shown a wide range of ω3 anti-inflammatory effects, but in vivo investigations in humans have shown conflicting results.^154 , 245^

PUFAs of the ω3 series, such as EPA and DHA, are precursors of anti-inflammatory eicosanoids with cardiovascular benefits. Although experimental studies have demonstrated the anti-inflammatory effects of ω3, some studies in humans have shown conflicting results regarding cardiovascular outcomes.^133 , 154 , 245^

In cross-sectional and cohort studies, dietary intake of marine ω3 was associated with lower plasma levels of inflammatory markers, including adhesion molecules and CRP.^246 , 247^Concentrations of marine ω3 in plasma and in erythrocyte or granulocyte membranes were inversely associated with CRP concentrations in healthy individuals or patients with stable CAD.^248 - 250^An intervention study showed that food containing marine ω3 or supplementation with fish oil or DHA produced results compatible with attenuation of the inflammatory response in patients with T2D and hypertriglyceridemia.^251 - 253^In other trials, a diet supplemented with ω3 did not cause significant changes in inflammatory parameters in patients with cardiometabolic risk (1.24 g/day)^254^or in patients with previous AMI (5.2 g/day),^255 , 256^and the same was observed with PUFA supplementation in plasma CRP concentrations of healthy individuals (2.0 or 6.6 g/day).^256^Differences in the population profile, route of administration, supplementation dose, concomitant use of statins, and analyzed parameters may have contributed to the discrepant results. Therefore, the real clinical relevance of the anti-inflammatory effects of ω3 fatty acids of marine origin is still uncertain.

Studies involving ALA have shown an inverse relationship between ALA intake and inflammatory parameters, including serum CRP.^246 , 257 , 258^ALA supplementation reduced the concentration of inflammatory markers in patients with dyslipidemia, which occurred especially when the baseline diet was high in SFAs and low in MUFAs.^259^

Trans fat intake was positively associated with systemic inflammation, characterized by an increase in IL-1β, IL-6, TNF-α, and monocyte chemoattractant protein (MCP) levels in patients with CVD.^260^A case-control study of 111 patients with CAD showed that the incorporation of trans fatty acids into erythrocytes was associated with higher plasma levels of CRP and IL-6.^77^

## 8. Insulin Resistance and Diabetes

Inflammatory signaling induced by SFA intake can activate proteins with serine kinase activity, such as c-Jun N-terminal kinase (JNK) and IKK. These proteins negatively interfere with insulin signal transduction by reducing tyrosine phosphorylation of IRS-1.^261 , 262^

Intake of a high-SFA diet for 3 months reduced insulin sensitivity in individuals without diabetes.^263^In the LIPGENE cohort study, which evaluated 417 individuals with metabolic syndrome, reduced SFA intake had no impact on fasting plasma glucose and insulin concentrations, homeostasis model assessment of insulin resistance (HOMA-IR), insulin sensitivity, and inflammatory markers.^264^It is worth noting that, in the LIPGENE study, energy from SFAs was replaced with energy from UFAs or complex carbohydrates. In the Reading, Imperial, Surrey, Cambridge, and Kings (RISCK) trial, involving 548 overweight participants with high cardiometabolic risk, the isocaloric replacement of a SFA-rich diet (with high glycemic index) with a MUFA-rich diet (with high or low glycemic index) for 6 months caused no change in insulin sensitivity.^265^However, it was demonstrated that diets enriched with SFAs, especially palmitic acid, acutely induced insulin resistance in individuals with and without glucose intolerance.^266^

Prospective studies have found a positive association between SFA intake and glucose intolerance.^267 , 268^The HPFS study, which included 42 504 men, found an association of total fat and SFA intake with an increased risk of T2D, but the association was dependent of BMI.^269^In the Iowa Women’s Health Study,^270^involving 35 988 women without a previous diagnosis of T2D, SFA intake was not associated with the risk of T2D; however, the risk of diabetes was inversely related to the replacement of SFAs with PUFAs. In addition, consumption of animal fat was associated with a 20% increase in T2D risk.^270^Another prospective study, the NHS study, which assessed the relationship between fat intake and T2D risk in 84 204 women, concluded that total fat and SFA intake was not associated with an increased risk of T2D.^271^

The WHI trial investigated the effects of dietary intervention in postmenopausal women followed for about 8 years and found that reduced intakes of total fat (9.1% of energy) and SFAs (3.2% of energy) did not change the risk of developing T2D. It is worth noting that the reduction in fat intake was offset by a 10% increase in carbohydrate intake.^272^

The development of T2D is known to result from the interaction of genetic factors and lifestyle, such as diet. The EPIC-InterAct study^273^evaluated potential interactions of genetic susceptibility and the effect of macronutrient intake on the risk of developing T2D and reported that SFA intake was not associated with T2D risk. Also, genetic susceptibility to T2D did not influence the relationship between macronutrient intake and T2D risk.^273^In another cohort of the EPIC-InterAct study, investigating the association between T2D risk and the concentration of different fatty acids in plasma phospholipids,^14^myristic, stearic, and palmitic acids were positively associated with T2D risk. It should be noted that a higher plasma concentration of these fatty acids was positively associated with the intakes of alcohol, margarine, and soft drinks and negatively with the intakes of fruit and vegetables, olive oil, and vegetable oil. Pentadecanoic acid (15:0) and heptadecanoic acid (17:0), however, were positively associated with the intakes of milk and dairy products, nuts, cakes, and fruit and vegetables and inversely associated with T2D risk.^14^Therefore, the observed deleterious effects cannot be attributed solely to the isolated activity of these SFAs, but rather to a context of inadequate diet.

A meta-analysis of observational studies found no association between SFA intake and T2D risk.^223^In a meta-analysis of cohort studies investigating the association between dietary patterns and T2D risk, a reduction in the risk of T2D was associated with healthy eating patterns, and not with a specific macronutrient.^274^In a meta-analysis of dietary intervention controlled studies evaluating the effect of isocaloric replacement of macronutrients on plasma glucose and insulin concentrations and on insulin resistance-related parameters, the replacement of SFAs with PUFAs reduced the glucose levels, glycated hemoglobin (HbA1c), C-peptide, and HOMA.^109^

To date, the evidence on the impact of SFAs on T2D risk is inconclusive. Results indicate that the influence of other dietary nutrients and components cannot be discarded, which is in line with international and Brazilian dietary guidelines. Therefore, the adoption of healthy eating patterns is recommended. Priority should be given to the consumption of fruit and vegetables, dairy products, lean meats, and complex carbohydrates, with low intake of simple carbohydrates, processed meats, and ultra-processed foods—such diet is considered more efficient in reducing the risk of cardiometabolic diseases.

Prospective cohort studies involving a large number of participants have suggested that a higher intake of ω3 fatty acids is associated with a higher incidence of T2D.^270 , 275^However, in a meta-analysis evaluating the relationship between marine ω3 PUFAs and T2D risk,^276^both the intake of fish and crustaceans (13 studies, RR per 100 g of fish/day = 1.12, 95% CI: 0.94-1.34) and supplementation with EPA+DHA (16 cohorts, RR per 250 mg/day = 1.04, 95% CI: 0.97-1.10) were not associated with the risk of diabetes. Plasma concentrations of EPA+DHA (5 cohorts, RR per 3% of total fatty acids = 0.94, 95% CI: 0.75-1.17) were also not associated with T2D risk.^276^Given the heterogeneity between studies and inconsistent effects related to follow-up duration, there is no evidence of beneficial or harmful effects of fish/seafood intake or EPA+DHA supplementation on the risk of developing diabetes.

However, there is evidence that higher plasma EPA/DHA levels may be associated with a lower risk of new-onset diabetes.^277^Despite the benefits described after ω3 intake in patients with T2D, a meta-analysis involving 23 randomized controlled trials showed no significant changes in HbA1c, fasting glucose, or fasting insulin when ω3 was supplemented at a mean dose of 3.5 g/day.^86^. Likewise, another meta-analysis of 26 controlled trials found that fish-oil supplementation, ranging from 2 to 22 g/day, did not change plasma HbA1c levels in patients with diabetes;^278^however, the high doses used in the studies should be taken into consideration. In addition, the Outcome Reduction with an Initial Glargine Intervention (ORIGIN) trial showed that ω3 supplementation did not reduce the rate of cardiovascular events in patients with glucose intolerance, impaired fasting glucose, and T2D.^279^

The effects of ALA on the glycemic profile have also been inconsistent.^217^However, it has been suggested that ALA intake may benefit glucose metabolism. Prospective data from the CHS study showed that higher plasma ALA levels were associated with a lower risk of new-onset T2D.^277^Similarly, in a large prospective analysis of more than 43 000 Chinese adults, ALA intake was inversely associated with the risk of incident T2D.^280^In a systematic review and meta-analysis of randomized controlled trials, ALA supplementation reduced blood glucose by 3.6 mg/dL.^67^Regarding flaxseed, a randomized controlled trial showed an improvement in insulin sensitivity.^281^

A systematic review identified 16 prospective studies, including cohort studies, that evaluated the relationship of ω3 intake and plasma levels with the incidence of T2D. Of a total of 540 184 individuals, 25 670 were cases of incident T2D.^276^Both ALA intake (n = 7 studies) and plasma ALA concentration (n = 6 studies) were not associated with T2D risk. Moderate heterogeneity (<55%) was observed for circulating ALA levels and diabetes, which may suggest a slightly lower risk of T2D.^276^

A review on ω3 fatty acids, cardiometabolic risk, and T2D concluded that there are no data demonstrating that ALA reduces the conversion of cardiometabolic risk to T2D or reduces mortality in people with T2D or cardiometabolic risk. ALA appears to reduce platelet aggregation in people with diabetes.^282^

Observational studies, using biological markers of fat intake or dietary surveys, suggest an inverse association between ω6 intake and T2D risk, although the data are not always consistent.^271 , 283^In the NHS study, involving 84 204 women aged 34 to 59 years without diabetes, CVD, or cancer who were prospectively followed for 6 years,ω6 intake assessed by validated food-frequency questionnaires was associated with a lower risk of T2D.^271^In men, a large prospective study, the HPFS study, also showed that the intake of ω6 as linoleic acid was associated with a lower risk of T2D in those aged <65 years and with BMI <25 kg/m^2^.^269^Also, in the Singapore Chinese Health Study, in which more than 43 000 Chinese adults were prospectively assessed, neither ω6 intake nor the ω6/ω3 ratio was associated with new-onset T2D.^280^

Data from small intervention studies are also conflicting regarding the effect of ω6 on insulin sensitivity.^284^Further long-term, controlled studies are needed to identify the best dietary fatty acid composition to reduce the risk of T2D. Few data are available, and the effects of dietary fatty acid types (PUFAs and SFAs) on glycemic control in people with diabetes remain uncertain.^285^

Regarding trans fatty acids, experimental studies have shown adverse effects on glucose homeostasis and development of diabetes.^286 - 288^In addition, trans fatty acids increase plasma levels of TG, insulin, and postprandial glucose^289^and reduce glucose uptake by the skeletal muscle—changes that are accompanied by increased visceral and hepatic fat.^287^A study using data from the NHANES survey to investigate the association between trans fatty acids and metabolic syndrome found that plasma trans-fatty-acid concentration was positively associated with risk of metabolic syndrome and its individual components.^290^Even in small amounts, trans fatty acids have deleterious effects on glucose homeostasis, stimulating glycogenesis and increasing visceral fat.^286 , 289^Consumption of a trans fat-rich diet has been shown to induce greater weight gain, hepatic steatosis, and insulin resistance by suppressing the IRS-1 signaling pathway, with a consequent reduction in Akt and protein kinase C (PKC) phosphorylation.^286^In overweight patients with T2D, the intake of trans fatty acids has been consistently correlated with reduced insulin sensitivity and increased postprandial glucose and insulinemia.^291^

The CHS study, investigating the association of the incidence of T2D with both plasma phospholipid trans fat concentration and their consumption, found that plasma trans fatty acid concentrations were positively associated with the incidence of T2D after correction for de novo lipogenesis.^292^However, after adjusting for the intake of other foods, trans fatty acid intake was not associated with the incidence of T2D.^292^An important systematic review showed that trans fat intake was associated with a 28% increase in the risk of T2D, when studies with a low risk of bias were analyzed, in addition to being associated with increased all-cause mortality (34%), coronary heart disease mortality (28%), and cardiovascular risk (21%).^223^

## 9. Fatty Liver Disease

### 9.1. Hepatic Steatosis

The liver has a great metabolic capacity for the metabolism of all nutrients, especially fats. However, intracellular TG accumulation in more than 5% of hepatocytes characterizes nonalcoholic fatty liver disease (NAFLD),^293^a broad-spectrum clinical condition that initiates with hepatic steatosis and then progresses to nonalcoholic steatohepatitis (NASH), marked by the presence of fat and inflammatory infiltrate. This condition predisposes the person to the appearance of hepatic complications, such as fibrosis, cirrhosis, and hepatocellular carcinoma,^294 , 295^and extrahepatic complications, such as CVD and T2D.^296^The diagnosis should exclude secondary causes of hepatic steatosis, such as alcohol abuse, viral or autoimmune hepatitis, or steatosis due to use of steatogenic drugs.^296 , 297^

NAFLD is strongly associated with factors that compose the cardiometabolic risk profile, such as obesity, insulin resistance, T2D, and dyslipidemia.^296 , 297^About 90% of patients with NAFLD have at least one cardiometabolic risk factor, and 30% have all factors. The risk of NAFLD incidence has been shown to increase proportionally to the sum of factors related to cardiometabolic risk. For this reason, NAFLD is identified as the hepatic manifestation of cardiometabolic risk.^298^Individuals with T2D are at a 2-to-4-fold increased risk of progression to steatohepatitis together with the development of fatty liver disease complications.^294^

The development of NAFLD is related to an increased influx of free fatty acids (FFAs) to the liver, mainly due to increased lipolysis in adipose tissue, associated with insulin resistance and excess calories in the diet.^299^In patients with NAFLD, about 60% of hepatic TGs stem from adipose tissue lipolysis, 26% from de novo lipogenesis, and 14% from the diet.^300^Additionally, there is an increase in hepatic lipogenesis together with a decrease in mitochondrial β-oxidation or VLDL secretion by the liver, contributing to hepatic lipid accumulation.^301 , 302^Hepatic lipid accumulation may then lead to inflammation, development of fibrosis, and loss of function. Fibrosis is the most important predictor of NAFLD-related mortality, and its presence increases the risk of death from CVD and liver diseases.^296^

Other factors may be related to the progression of the disease, such as: 1) increased ROS generation, promoting oxidative stress due to mitochondrial dysfunction or ER stress;^303^2) lipid peroxidation; 3) activation of inflammatory pathways with a consequent increase in hepatic secretion of cytokines and inflammatory mediators such as TNF-α and IL-6, which may deteriorate the condition.^304^Moreover, lack of physical activity associated with a poor diet, i.e., rich in fats and excess calories, predisposes the development of NAFLD.^305^

Individuals with NAFLD have increased hepatic expression of genes related to fatty acid transport (fatty acid-binding proteins 4 and 5), TG hydrolysis (LPL), and recruitment of monocytes (MCP1) and PPAR-γ2.^306^PPAR-γ has been shown to induce SREBP-1c expression, with enhanced expression of genes that control proteins related to hepatic TG synthesis.^307^

Studies in animal models^308 , 309^or clinical trials using human participants^306 , 310^have strongly demonstrated the participation of a high-fat diet in the induction of hepatic steatosis. Insulin resistance plays a major role in hepatic lipid accumulation^311^and, within this context, the amount of fat (especially the type of fatty acid) influences hepatic lipogenesis and the action of insulin.^301 - 303^

### 9.2. Saturated Fatty Acids and Nonalcoholic Steatohepatitis

In hepatocytes, stearic acid and palmitic acid are able to induce apoptosis via excess JNK activation.^312^Another finding was that palmitate treatment can activate the IRE1α signaling pathway via TLR4. IRE1 is an ER transmembrane protein that governs the response to malformed proteins in the reticulum and induces apoptosis.^313^

A recent study demonstrated that palmitic acid promotes oxidative stress, ER stress, mitochondrial dysfunction, and inflammation in HepG2 cells. Animals that were given a high-fat diet rich in SFAs developed hepatic steatosis, NASH and fibrosis, conditions associated with ER stress, and insulin resistance. Conversely, the addition of oleic acid to the diet protected against SFA-induced hepatic lipotoxicity.^314^SFA or sucrose intake by experimental animals induced SFA accumulation in the liver, ER stress, and apoptosis compared to a PUFA-rich diet.^315^

A study in humans showed that total fat intake and SFA intake were positively associated with hepatic lipid content.^316^A 7-week randomized double-blind study in healthy individuals revealed that diets rich in palmitic or linoleic acid promoted similar weight gain. However, excess calories from SFAs increased the deposition of liver fat, visceral adipose tissue, and total fat as well as reduced the percentage of lean tissue when compared to a PUFA-rich diet. Additionally, increased body and liver fat correlated positively with elevated plasma concentrations of palmitic acid and inversely with linoleic acid.^317^

A recent study showed that an additional consumption of 1000 kcal in the form of SFAs for 3 weeks led to a greater increase in intrahepatic lipid content (55%) when compared to the same extra intake of UFAs or sugars, which elevated hepatic lipid content by 15% and 33%, respectively. SFA intake also induced insulin resistance and increased plasma ceramide concentrations by 49%.^318^

### 9.3. Unsaturated Fatty Acids and Nonalcoholic Steatohepatitis

In the liver, SCD1 is the enzyme primarily responsible for inserting double bonds in saturated chains of fatty acids such as palmitic acid (C16:0) and stearic acid (C18:0), converting them to palmitoleic acid (C16:1) and oleic acid (C18:1), respectively. The aim is to control excess SFA content in the body, either from food or from excess endogenous conversion of palmitic acid derived from de novo lipogenesis. In NAFLD, lipogenic pathways are activated, and desaturation (SCD1) and oxidation pathways are reduced. This is partly due to insulin resistance and mainly due to a local inflammatory process.^319^Errazuriz et al.^320^found that patients with NAFLD had reduced liver fat (assessed by spectroscopic magnetic resonance imaging [MRI]) when they consumed a MUFA-rich diet for 12 weeks (22% of energy) compared to the control group (8% of energy). Such changes occurred even though the diets were isocaloric and the participants had no weight loss at the end of the study.^320^

In a randomized study conducted by Bozzetto et al.,^321^patients with T2D were assigned to one of the following interventions: (1) high-MUFA diet; (2) high-carbohydrate/high-fiber/low-glycemic index diet; (3) high-carbohydrate/high-fiber/low-glycemic index diet plus exercise; or (4) high-MUFA diet plus exercise. There was a reduction of up to 30% in hepatic lipid content in patients assigned to the high-MUFA diet, regardless of exercise.^321^The same group of researchers demonstrated, in a subsequent study, that liver fat reduction was due to the activation of hepatic oxidative pathways, based on measurement of β-hydroxybutyrate. Despite having identified an increase in β-oxidation, they found no increase in the ratio of palmitoleic to palmitic acid, which implies that there was no difference in SCD1 activity.^322^

Together with the lipolytic action promoted by MUFAs, the anti-inflammatory action coordinated by oleic acid may be involved in the potency of the restoration of liver function, as demonstrated by Morari et al.^323^In their study, HepG2 cells treated with oleic acid showed increased gene expression and protein content of IL-10, a protein with a potent anti-inflammatory action. Oleic acid activates the protein PGC-1α, which binds to another protein, cMAF. In the form of a dimer, PGC-1α and cMAF migrate to the nucleus and induce exclusive transcription of the IL-10 gene.^323^

Similarly, PUFAs have different hepatic metabolic responses. Omega-6 fatty acids (linoleic and arachidonic acids) and ω3 fatty acids (ALA, EPA, and DHA) participate in hepatic metabolism but are primarily intended for constitution of cell membranes, intracellular signaling as second messengers, and other functions, thus being diverted from their use as an energy substrate.^324^In 2007, Yamaguchi et al.^325^experimentally inhibited hepatic TG synthesis. Despite an improvement in steatosis, liver damage intensified and then progressed to fibrosis and cirrhosis. The study demonstrated that, with an increase in FFAs in the cytoplasm, there was greater ROS oxidation, inducing important inflammation.^325^

Although some studies suggest an improvement in NAFLD with ω3 fatty acid supplementation,^326^there are still inconsistencies in the literature regarding its benefits.^327 , 328^In a randomized study of children with NAFLD, daily intake of 1300 mg of ω3 fatty acids for 6 months reduced aspartate aminotransferase and gamma-glutamyl transpeptidase, in addition to increasing serum adiponectin, but these changes were not sufficient to reduce the degree of steatosis on ultrasound.^329^Some of the inconsistencies found in the studies are usually related to the experimental design, the certification of the content of the chosen capsule, and the choice of placebo, among other factors. Tobin et al.^330^conducted a randomized, double-blind study in which they treated 291 patients with a concentrated ω3 fatty acid capsule (460 mg EPA + 380 mg DHA) or placebo (olive oil) for 24 weeks. MRI-proton density fat fraction assessment showed a significant reduction in hepatic lipid content, similar in both groups, which was attributed to adherence to a healthy dietary pattern.^330^

Although ω3 fatty acids reduce TG synthesis by blocking SREBP,^83 , 331 , 332^results of clinical trials^329 , 330^do not support the recommendation of ω3 fatty acid supplementation in the treatment of NAFLD and NASH, as discussed in a position statement by the American Association for the Study of Liver Diseases.^297^

### 9.4. Trans Fatty Acids and Nonalcoholic Steatohepatitis

A high-fat diet enriched with trans fatty acids induced an increase in the expression of transcription factors involved in hepatic lipogenesis (SREBP-1c and PPAR-γ) and reduced MTP, suggesting less ability to export TGs, which led to the development of NASH.^308^A study that evaluated 4242 participants in the NHANES cohort showed a positive association between plasma concentration of trans fatty acids and NAFLD, which was estimated by plasma biomarkers of liver function such as alkaline phosphatase, alanine aminotransferase, aspartate aminotransferase, and gamma-glutamyl transferase.^333^

Diet composition may influence the development of NAFLD,^334^and, within this context, excess SFAs may contribute to intrahepatic lipid accumulation.^318^Conversely, healthy dietary patterns rich in UFAs, such as the Mediterranean diet, seem to have beneficial effects, including improved steatosis even if there is no weight loss.^335 , 336^However, further prospective studies comparing the effect of macronutrients on NAFLD and evaluating pre- and post-treatment histological components are needed.

The treatment of NAFLD consists primarily of weight loss, which is achieved by reducing energy intake by approximately 30%. Losing 3% to 5% of body weight reduces steatosis, and losing 7% to 10% of baseline weight contributes to the improvement of histological components of steatohepatitis and fibrosis.^337^Physical activity combined with caloric restriction aids weight loss and maintenance.^297^

Thus, individuals with NAFLD should be instructed to follow a calorie-restricted diet and practice physical activity to lose weight. The adoption of healthy dietary patterns should be encouraged, including a large amount and a varied range of fruits and vegetables, in addition to favoring complex carbohydrates over simple carbohydrates, with increased UFA intake and adequate SFA intake.^297^

## 10. Lipid Metabolism in Adipose Tissue

Adipose tissue is composed of adipocytes, preadipocytes, immune cells, fibroblasts, lymph nodes, and nervous tissue. The adipocyte is the only cell that can store fat without compromising its function, which primarily is to promote lipogenesis and lipolysis.^338^Additionally, adipose tissue is able to secrete several bioactive substances such as leptin, cytokines (TNF, IL-6, MCP1, IL-1β), and other adipokines, performing autocrine, paracrine, and endocrine functions.^339^Such actions can be modulated by different fatty acids from the diet.

In response to excess energy and in an attempt to restore tissue homeostasis, the adipose tissue undergoes a remodeling process consisting of adipocyte hypertrophy and hyperplasia and high cytokine secretion, which characterizes them as proinflammatory cytokines.^340^However, in the long term, secretion of TNF-ɑ, IL-6, iNOS, and MCP1, together with recruitment of inflammatory cells such as neutrophils, T cells, and macrophages, promote inflammation, fibrosis,^339 - 341^and insulin resistance in adipose tissue,^342^which plays a key role in the metabolic derangements observed in obese patients.^343^

Cell signaling mediated by TNF-α receptors culminates in NF-kB activation, which increases cytokine secretion and characterizes local inflammation. In this condition, the adipocyte shows increased lipolysis with increased FFA release. SFAs derived from adipocyte lipolysis activate TLR4s in tissue-resident macrophages, intensifying the local inflammatory response and establishing a vicious circle.^344^Concomitantly with these actions, there is a gradual polarization of macrophages from the M2 subpopulation (anti-inflammatory action linked to resolution of injury) to the M1 subpopulation (classic activation pathway associated with Th1 response). Thus, there is an intensification of the inflammatory state and induction of insulin resistance in adipose tissue.^339^In obese patients, other factors such as adipose tissue hypoxia, ER stress, and endotoxemia also contribute to the maintenance of inflammation in adipose tissue.

Insulin has an important effect on adipose tissue, as it inhibits lipolysis and stimulates lipogenesis and glucose and FFA uptake. The activation of inflammatory pathways antagonizes the action of insulin by inducing resistance to the hormone and favors the appearance of diseases associated with cardiometabolic risk.^343^

TGs from the diet are packaged into chylomicrons and hydrolyzed by the action of LPL,^343^releasing FFAs, which are then directed to adipose tissue and to a lesser extent to the muscle.^345^Thus, the type of fatty acid in adipose tissue has a strong correlation with the fatty acid in the diet.

### 10.1. Saturated Fatty Acids and Adipose Tissue Metabolism

An in vitro study showed that preincubation of adipocytes with palmitic acid induced cell hypertrophy with a consequent increase in MCP1 secretion and hydroperoxide concentration, a marker of oxidative stress.^345^These effects were not observed with oleic acid.^345 , 346^In another study, palmitic acid activated NF-kB and increased the expression of proinflammatory cytokines in 3T3-L1 adipocytes.^347^In experimental animals, a high-fat diet rich in lauric acid induced the activation of proinflammatory cytokines (TNF-α, IL-6, MCP1, IL-1β, IFNγ) and activated serine kinases such as IKKβ and JNK in adipose tissue, with a reduction in AMPK phosphorylation.^348^Conversely, it increased the production of cytokines with anti-inflammatory action in an attempt to rescue tissue homeostasis.^348^

In animal models, consumption of a high-fat diet rich in palmitic acid led to increased dendritic cell infiltrate in adipose tissue, together with the development of insulin resistance. In dendritic cells, palmitic acid induced increased expression of maturation markers such as CD40, CD80, MHCII, and TLR4. An increased expression of caspase-1 and IL-1β genes suggests parallel activation of the inflammasome pathway, another intracellular structure involved in the control of inflammatory tone.^237^

A subsequent study conducted by the same research group showed that a SFA-rich diet induced insulin resistance, reduced glucose uptake, and increased plasma insulin concentrations. Moreover, there was a reduction in the expression of IRS1 and glucose transporter type 4 in adipose tissue, as well as tyrosine phosphorylation of IRS1 and AKT. These effects were not observed in the groups undergoing the MUFA-rich diet.^349^

Kolak et al.^350^found that an increase in macrophage infiltrate, MCP1 and PAI1 expression, and ceramide accumulation occurred in subcutaneous adipose tissue regardless of BMI. In addition, these changes positively correlated with hepatic lipid accumulation.

A cross-sectional study that included 484 participants in Japan showed that SFA consumption (assessed by fatty acid concentration in plasma phospholipids) correlated with a reduction in adiponectin and an increase in resistin and visfatin, which are adipokines related to insulin resistance and adipogenesis.^351^

A study of overweight individuals that evaluated the additional consumption of 1000 kcal/day in SFAs (coconut oil and butter), UFAs (olive oil and nuts), or sugars showed that SFAs induced insulin resistance and increased the expression of genes related to inflammatory pathways in adipose tissue.^318^

### 10.2. Unsaturated Fatty Acids and Adipose Tissue Metabolism

Adipose tissue stores SFAs more efficiently; however, if there is a high proportion of UFAs in the diet, lipid deposition on adipose tissue may follow the same dietary profile.^352^Because of the difficulty in investigating tissue dispersion profile of fatty acids obtained from food in humans, most of the studies are conducted in animals.^353^Providing a high-fat diet to mice for only 3 days was shown to increase the amounts of palmitic and oleic acid in adipose tissue, with oleic acid being deposited preferably in the mesenteric adipose tissue.

The study also showed that only oleic acid was able to change the inflammatory profile of M1 macrophages to the anti-inflammatory M2 profile, both in animal tissue and in adipocyte culture.^353^In the LIPIGENE study, 39 patients with cardiometabolic risk assigned to a high-oleic acid diet showed increased expression of genes that control autophagy (Beclin-1 and ATG7) and apoptosis (CASP3 and CASP7) compared to both the low-fat, high-complex carbohydrate group and the high-complex carbohydrate, high-ω3 fatty acid group.^354^

Several studies have demonstrated the correlation between arachidonic acid content in adipose tissue and AMI.^355 - 358^A case-cohort study showed a strong correlation (39% of participants) between arachidonic acid content in adipose tissue and AMI.^359^This is explained by the rapid release of arachidonic acid by the adipocyte, which is a substrate for the synthesis of proinflammatory and prothrombotic eicosanoids, favoring inflammation and destabilization of the atherosclerotic plaque. In addition, this fatty acid has been associated with insulin resistance and may increase cardiovascular risk.^359^

The known anti-inflammatory potential of ω3 fatty acids seems to positively interfere with the control of tissue inflammation in patients, but more robust evidence is still needed. Spencer et al.^360^treated insulin-resistant but nondiabetic patients with 4 g of ω3 fatty acid (ethyl ester) for 12 weeks and observed a significant reduction in MCP1, and thus macrophages, in adipose tissue but not in the muscle. These phenomena were not followed by a reduction in plasma cytokine concentration, insulin sensitivity, or adiponectin. In a coculture experiment of adipocytes and macrophages from the same participants, the adipocytes of patients who consumed ω3 fatty acids had reduced MCP1 content even in the presence of macrophages.^360^In a randomized, double-blind controlled study, overweight and obese pregnant women were supplemented with 2 g of ω3 fatty acid (EPA + DHA) twice a day, from gestational week 10 to birth. Plasma concentration of CRP decreased significantly, followed by reduced TLR4 in adipose tissue and decreased gene expression of TNF, IL-6, and IL-8 in placental tissue.^361^

Difficulties in the development of general recommendations for fatty acid intake in patients with diseases are due to a wide variation in experimental protocols, including different types of food, duration of diets, conflicts of interest of the study authors, and the quality of scientific information, among others.

In a double-blind, placebo-controlled study, insulin-resistant patients were given a daily supplementation of 3.9 g of ω3 fatty acids (EPA + DHA) for 6 months and underwent adipose tissue biopsy before and after the intervention. No benefit associated with tissue metabolism was observed.^362^However, in a study of human adipocytes, EPA induced an increase in the expression of genes involved in adipocyte “beiging”. Proteins involved in mitochondrial biogenesis, such as uncoupling protein 1 and carnitine palmitoyltransferase 1, were stimulated. The same study showed, however, that arachidonic acid reduced mitochondrial respiration and then energy expenditure.^363^Finally, considering the evidence found to consolidate the decision-making process regarding ω3 fatty acids and their relationship with adipose tissue function, Iturari et al.^364 , 363^treated 55 obese, nondiabetic patients eligible for bariatric surgery with 3.3 g of ω3 fatty acids (EPA + DHA) for 8 weeks. There was a significant reduction in subcutaneous adipose tissue, content of chemokines CCL2 and CCL3, IL-6, hypoxia-inducible factor 1-alpha and transforming growth factor-beta, and CD40, as well as an increase in adiponectin. No changes induced by ω3 fatty acid consumption in visceral adipose tissue were observed in the experimental group compared to the placebo group.

Despite the potential metabolic benefits from ω3 fatty acid consumption, there is no consensus on its relevance for the treatment of dysmetabolism regarding adipose tissue function. Conversely, there is a greater body of evidence supporting incremental metabolic benefits of MUFAs for conditions associated with dysmetabolism.

## 11. Food

### 11.1. Coconut Oil

Coconut oil is composed almost entirely (92%) of SFAs, of which lauric acid accounts for approximately 50%, followed by myristic acid (16%), palmitic acid (8%), and finally caprylic, capric, and stearic acids. Regarding essential fatty acid concentrations, coconut oil has a low concentration of linoleic acid (18:2) and no linolenic acid (18:3).^43 , 365^

The largest coconut oil producers are the Philippines, Indonesia, and India, extracting two different types of oil: one is refined, bleached, and deodorized, and the other is virgin, cold-pressed, with no refining processes.^366^Coconut oil consumption has grown significantly in recent years, and this is partly due to the fact that its properties have been erroneously associated with those of medium-chain triglycerides, formed mainly by caprylic acid (8:0) and capric acid (10:0),^367^which are absorbed bound to albumin and reach the liver via portal system, with no consequent increase in TGs. Lauric acid, the main fatty acid in coconut oil, is largely transported by the lymphatic system after being absorbed,^38^and its presence in chylomicrons is dose-dependent.^38^

Beneficial associations regarding coconut oil consumption possibly stem from a study conducted on people from Pukapuka and Tokelau, two Polynesian islands that exhibit low incidence of CVD. The typical diet of this population is rich in saturated fat, and coconut is the main source of fat and energy; protein is obtained mainly from fish, and carbohydrate is obtained from native fruits such as breadfruit. In addition, the diet is high in fiber and low in sucrose and processed foods, because of the limited access to these foods.^368^This situation has changed in recent decades, possibly because of the migration to Western dietary habits, even though coconut oil consumption was maintained. In 2010, about 40% of the Polynesian population was diagnosed with chronic diseases (CVD, T2D, and hypertension), which were responsible for three-quarters of deaths in the archipelago.^369^

Coconut oil is able to increase plasma concentrations of TC and LDLc compared to other fats such as olive oil^370^and safflower oil.^371^A study in humans showed that lauric acid elevates TC and LDLc, compared to a MUFA-rich diet, but less markedly than palmitic acid.^372 , 373^Mendis et al.^373^found that the isocaloric replacement of coconut oil, typically found in the diet of Sri Lankan people, with soybean oil rich in PUFAs reduced the plasma concentrations of TC, LDLc, and TG in normolipidemic individuals. The same result was obtained with corn oil in dyslipidemic individuals.^219^

Furthermore, studies showing increased HDLc concentrations with coconut oil intake have shown a concomitant increase in LDLc, which is known to elevate cardiovascular risk.^374^

SFAs are known to activate inflammatory signaling pathways, as well as ER stress, autophagy, and apoptosis, via activation of TLRs linked to the innate immune response.^375^TLRs recognize pathogen-associated molecular patterns such as LPS, found in the cell wall of gram-negative bacteria, and then alert the immune system. When activated, TLRs trigger signaling that culminates in the transcription and secretion of proinflammatory cytokines.^375^

Lauric acid, among all SFAs, has the greatest inflammatory potential.^241^An in vitro study in macrophages showed that lauric acid induced NF-κB activation, leading to increased expression of cyclooxygenase-2 via activation of TLRs 2 and 4.^376^The ability of lauric acid to activate inflammatory pathways by activating TLR4, leading to inflammatory cytokine secretion and T-cell activation, has already been described in different cell types.^241 , 377^

A study that compared the effect of consuming coconut, palm, or olive oil for 5 weeks on inflammatory parameters of normocholesterolemic individuals found no difference in plasma concentrations of homocysteine and inflammatory markers such as TNF-α, IL-1β, IL-6, INF-γ, and IL-8. However, in that study, the standard deviation was excessively high and may have masked differences in inflammatory profile.^370^

Valente et al.^378^evaluated the acute effect of a diet rich in coconut oil compared to olive oil in 15 overweight women and found no difference regarding energy metabolism and lipid oxidation.

Regarding the antioxidant properties attributed to polyphenols found in virgin coconut oil, studies are still preliminary and were conducted mostly in experimental animals, thus their findings cannot be translated into humans.

To date, there are no randomized controlled studies and epidemiological studies evaluating the effect of coconut oil on lipid profile, inflammatory profile, and cardiovascular outcome. Thus, there is no evidence to indicate coconut oil as a substitute for UFA-rich vegetable oils.

### 11.2. Palm Oil

Palm oil, together with interesterified fats, has been widely used by the industry as a substitute for trans fat in food. Despite being a vegetable oil, palm oil is composed of SFAs (45% palmitic acid and 5% stearic acid) and UFAs (40% oleic acid and 10% linoleic acid). Thus, an increase in direct consumption of palm oil, or indirect consumption via processed foods, will contribute to a greater SFA intake, which elevates cardiovascular risk.

In humans, a palm oil-rich diet increased plasma concentrations of TC and LDLc compared to consumption of high-UFA vegetable oil.^379^A meta-analysis of intervention studies found that, compared to vegetable oils with low SFA concentrations such as canola, soybean, and olive oil, palm oil increased the concentrations of TC, LDLc, and, to a modest extent, HDLc, which is consistent with the effect of SFAs on lipoprotein profile. Compared to trans fat, the increase in HDLc was more pronounced, as trans fat intake reduces its concentrations.^380^Conversely, palm oil seems to have similar effects to animal fat on plasma lipids.^380 , 381^

Palm oil consumption should be kept within the recommended SFA intake range. Despite being a vegetable oil, palm oil is very rich in palmitic acid and thus seems to act similarly to animal fats.

### 11.3. Chocolate

Chocolate is obtained from the cocoa bean, which comes mainly from countries in South America and the west coast of Africa. In addition to cocoa, cocoa butter, sugar, milk, and lecithin, other ingredients such as nuts, cereals, and fruits may be incorporated into the manufacture of chocolate, characterizing it as a high-energy density product rich in carbohydrates and fats. Chocolate also has polyphenols and minerals such as potassium, magnesium, iron, and zinc. Approximately 63% of cocoa fat is composed of stearic (34%) and palmitic (27%) acids. The remaining 37% are in the form of MUFAs (33.5%) and PUFAs (3.5%).^382^

Because it is rich in stearic acid, cocoa fat has a neutral effect on cholesterolemia. Studies that investigated food consumption in humans show that, compared to palmitic acid, stearic acid reduced plasma concentrations of TC and LDLc in a similar way to oleic acid. In addition, stearic acid increased oleic acid concentrations in plasma CE and TG,^383^which is explained by the fact that stearic acid is rapidly converted to oleic acid in the liver by the action of SCD1.^48^More recent data from the EPIC study showed a positive association between stearic acid concentrations in plasma phospholipids and risk of both coronary heart disease^108^and T2D.^14^However, it is important to note that stearic acid is also endogenously produced by de novo lipogenesis.

Stearic acid intake appears to have a neutral effect on cholesterolemia; however, it must be taken into account that chocolate is also a source of calories and simple sugars, which may contribute to weight gain and increased cardiovascular risk.

### 11.4. Butter

Butter derives from the cream obtained from milk that was skimmed; therefore, its fat comes exclusively from dairy fat. In a portion of butter, about 51.5% of fatty acids are SFAs, including palmitic (24%), stearic (10%), myristic (8%), and lauric (2%) acids, while the rest is composed of MUFAs (22%) and PUFAs (1.5%).^25^

A randomized study evaluating the impact of butter SFAs compared to isocaloric diets rich in UFAs on cardiometabolic risk showed that butter consumption increased the concentrations of TC, LDLc, and ApoB.^384^In a prospective cohort study of more than 26 000 individuals, consumption of butter, together with milk and milk products, was inversely associated with incidence of T2D.^385^In two other cohorts followed up for 10 and 20 years, no association was found between butter consumption and CVD.^386 , 387^However, it should be noted that in the MESA study cohort,^387^even in the highest quintile, the median consumption of butter was less than 5 g/day per person.

A systematic review of cohort studies with a high degree of evidence found no association between butter consumption and risk of CVD, CAD, and stroke. Conversely, there was an inverse association with risk of T2D.^388^

The results of the studies should be interpreted with caution, as the actions of SFAs in plasma lipids and cardiovascular health have been well consolidated. The use of butter should be part of a healthy, individualized dietary pattern that considers the added energy value and promotes weight management when required.

### 11.5. Dairy

Milk and milk products are an important source of calcium and high biological value protein. Conversely, whole-fat dairy consumption may increase the intake of SFAs, especially myristic acid, which has a strong correlation with increased cardiovascular risk. Skim dairy consumption is part of the DASH diet recommendations, a dietary pattern that was originally developed for the treatment of hypertension and, because of its cardiometabolic benefits,^389^is recommended as a healthy dietary pattern for all adults.^390^

More recently, studies have shown that dairy consumption is inversely associated with risk of T2D^14 , 391^stroke,^392^and CVD.^110^In those studies, plasma concentrations of pentadecanoic acid (15:0) and heptadecanoic acid (17:0) were used as markers of dairy consumption, as, because they are not endogenously synthesized, they must be obtained from the diet, and dairy is their main source.

It is important to note that the food matrix is a determining factor in cardiovascular risk, as, in addition to macronutrients, food provides micronutrients and fibers that contribute to a favorable cardiovascular outcome within the context of healthy dietary patterns. In contrast, the inclusion of processed foods rich in simple, refined sugars and additives such as food coloring agents, preservatives, and thickeners, may negatively impact cardiovascular risk. Additionally, the use of lipid-lowering drugs such as statins may mitigate or even mask the effects of SFA consumption on cardiovascular risk.^106^

### 11.6. Meat

The most consumed types of meat are beef, chicken, and pork, which are important nutritional sources of high biological value proteins, providing all essential amino acids, vitamins, and minerals. The amount of fat and the distribution of fatty acids will vary according to the source and the type of meat cut. Overall, meats contain mostly MUFAs and SFAs (especially palmitic and stearic acids) and a small amount of PUFAs.^25 , 28^

A positive association between meat consumption and cardiovascular risk has been observed in some studies^110^but not in others.^393^A study of more than 780 individuals found that consumption of red and processed meats correlated with a less healthy dietary pattern but not with CVD and T2D risk markers.^394^A prospective cohort study of more than 74 thousand individuals showed an association between greater consumption of (processed and unprocessed) meat and increased risk of CVD mortality (such association was found even in individuals with greater consumption of fruits and vegetables).^395^

An increased risk of all-cause and CVD mortality was associated with greater consumption of red and processed meats but not with consumption of unprocessed meats alone in two meta-analyses.^396 , 397^Processed meats are also rich in sodium and nitrogen compounds such as nitrates, which may contribute to a deleterious effect on cardiovascular risk because of their effects on blood pressure and endothelial function.

It is well established that high consumption of red and processed meats is associated with an increased cardiovascular risk, which is why their intake should be moderate and consistent with the total SFA intake recommended in the diet.

## 12. Gut Microbiota

High-fat diets, especially those rich in SFAs, are able to change the composition of gut microbiota,^398 - 400^induce decreased bacterial diversity, increased intestinal permeability, metabolic endotoxemia, and low-grade systemic inflammation,^401 - 407^and influence the development of several chronic diseases such as obesity, diabetes, and atherosclerosis.^408^Loss of intestinal epithelium integrity allows LPS from the cell membrane of gram-negative bacteria to translocate into plasma, culminating in metabolic endotoxemia.^401 , 403^

A greater consumption of high-SFA diets has been shown to increase intestinal paracellular permeability by interfering in tight-junction proteins, and thereby plasma concentrations of LPS are elevated.^409 , 410^Changes in intestinal permeability are related to the regulation of tight-junction proteins, a protein complex that maintains cell-cell junctions in the intestinal epithelium, forming a barrier against the passage of macromolecules.^411^

A study in mice found that a high-SFA diet induced greater formation of taurocholic acid, which allowed the expansion of sulfate-reducing bacteria such as *Bilophila wadsworthia* , an effect that was not observed in a high-PUFA diet. That study shows that changes in the composition of bile acids due to the type of dietary fat may cause dysbiosis, compromising host homeostasis.^400^

An increase in intestinal permeability induced by a high-fat diet, consisting mainly of SFAs, leads to changes in gut microbiota and increased inflammatory response, triggered by TLR4 activation by LPS.^412^Another mechanism may be associated with decreased secretion of the enzyme intestinal alkaline phosphatase by the duodenal brush border, which is responsible for detoxifying LPS, thus protecting against endotoxemia.^413^

An experimental study showed that a high-fat diet, especially when combined with a high-sugar diet, induces dysbiosis and inflammation in the intestinal epithelium and changes the activation of the vagal afferent pathway, actions that may impair the regulation of food intake, contributing to hyperphagia and development of obesity.^414^

### 12.1. Dietary Patterns and Gut Microbiota

Diet components have an important impact on the profile of gut microbiota. Therefore, different dietary patterns can modulate gut microbiota in distinct ways.

A study that investigated the association of dietary variables with gut microbiota identified 97 nutrients associated with relative abundance data or with presence/absence of microbiomes. The nutrients were divided into four groups: amino acids and choline; carbohydrates; fats; and fibers and vegetables. The study showed that the fat versus fiber groups were antagonistically associated with bacterial abundance,^415^i.e., bacteria that were positively associated with fat tended to be negatively associated with fibers. The same pattern of association was seen for the amino acid and protein versus carbohydrate groups and the fat versus carbohydrate groups. In addition, microbial rates that correlated with BMI also correlated with higher consumption of fats and SFAs.^415^

A recent randomized study of 217 healthy individuals compared the effect of isocaloric diets containing increasing concentrations of fat (20%, 30%, and 40%) and the same amount of fiber (14 g/day).^416^The high-fat diet increased fecal concentrations of palmitic, stearic, and arachidonic acids. The latter was positively associated with increased plasma concentrations of inflammatory mediators such as CRP as well as PGE2 and thromboxane B2, both derived from arachidonic acid. An important result of that study was that, even with adequate amounts of fiber in the diet, a high fat consumption prevented the formation of short-chain fatty acids (SCFAs) by bacteria.^416^Additionally, increased fat consumption reduced bacterial diversity.

### 12.2. Importance of Dietary Pattern in Short-chain Fatty Acid Synthesis

The production of glycoside hydrolases, which are responsible for the breakdown of some saccharides, is very limited in the human body. Conversely, some intestinal bacteria encode enzymes capable of digesting a wide range of polysaccharides, such as fibers.^417^The fermentation of soluble fibers promotes the formation of SCFAs, especially propionate (C3), acetate (C4), and butyrate (C5), which, in addition to serving as an energy substrate for colonocytes, perform systemic actions such as favoring glucose homeostasis.^418 , 419^

The presence of SCFAs induces secretion of intestinal incretins, such as GLP-1 and PYY, which act on the central nervous system by promoting satiety and reducing food consumption, decreasing gastric emptying time, increasing intestinal transit, in addition to stimulating insulin synthesis and secretion by the pancreas.^418^

A reduction in fiber consumption may impact the composition of the gut microbiota and the production of SCFAs. A prospective study of 17 obese individuals evaluated the impact of two high-protein/high-fat/low-fiber diets. The results show that both diets decreased fecal production of SCFAs and increased the concentration of branched-chain fatty acids, phenylacetic acid, and nitrogenous compounds, which are detrimental to colonic health.^420^

## 13. Dietary Cholesterol

### 13.1. Plasma Concentration of Lipids and Lipoproteins

The relationship between dietary cholesterol and plasma TC has been shown to be linear in observational cohort studies.^421 , 422^However, observational studies have limitations such as the presence of confounding variables, which may increase the magnitude of correlations, both positive and negative, and selection biases.^423^Furthermore, dietary cholesterol consumption is generally associated with increased consumption of SFAs, which are known to increase LDLc and cardiovascular risk.^424^

In recent years, there has been an intense discussion about the role of dietary cholesterol in the incidence of atherosclerotic complications. In response to that, the AHA no longer limits egg consumption as a way of protecting against CVDs. Thus, the Dietary Guidelines for Americans withdrew a recommendation for restricting cholesterol intake to no more than 300 mg per day.^7^However, the guidelines suggest that dietary cholesterol remains important and should be considered for developing healthy dietary patterns. They also highlight that dietary cholesterol consumption should be as low as possible, as recommended by the Institute of Medicine.^425^As noted, food sources containing high amounts of cholesterol are usually also rich in SFAs, such as fatty meats and high-fat dairy products. Therefore, the recommendation focuses on restricting SFAs to less than 10% per day, which should be sufficient to control dietary cholesterol.^7^

It is worth mentioning that not all people respond the same way to dietary cholesterol consumption, as the response is highly variable depending on genetic and metabolic factors^426 , 427^Lipid profile responses to dietary cholesterol were examined in 19 intervention studies. Cholesterol intake, mainly from eggs, led to an increase in both LDLc and HDLc, resulting in a slight increase in the LDLc/HDLc ratio. However, the analysis of this ratio can be very simplistic, as, while LDLc is an excellent marker of cardiovascular risk and changes in its value show a marked relationship with cardiovascular risk, changes in HDLc do not express possible changes in the functionality of HDL particles, which extends far beyond reverse cholesterol transport.^428^

Cholesterol consumption up to 400 mg/day from eggs is not associated with increased plasma TG concentrations in overweight individuals with diabetes or prediabetes.^429^

### 13.2. Risk of Developing Type 2 Diabetes

Observational and randomized studies have shown conflicting results regarding the association between dietary cholesterol consumption and risk of T2D. A case-control study demonstrated a 2-fold increase in the risk of T2D in individuals who consumed 3 to 4.9 eggs per week and a 3-fold increase in those who consumed more than 5 eggs per week, after adjusting for confounding factors such as BMI, family history of diabetes, smoking, physical activity, and plasma TG concentration.^430^An investigation that used data from two prospective randomized studies, Physicians’ Health Study I (1982-2007) and Women’s Health Study (1992-2007), demonstrated that during follow-up (20 years in men and 11.7 years in women) the development of diabetes was higher in those who consumed more than 5 eggs per week in men and more than 7 eggs per week in women, after multivariate adjustments.^431^However, other studies of populations from different regions have not shown the same association. A prospective study of the Japanese population (Japan Public Health Center-based Prospective Study) with a 5-year follow-up concluded that high intake of dietary cholesterol or eggs was not associated with a higher risk of T2D.^432^Opposite results were observed in the male population of the KuopioI Schaemic Heart Disease Risk Factor Study, which found that a higher egg consumption was associated with a lower risk of T2D in a 19.3-year follow-up.^433^

In the Jackson Heart Study, in an African American population, a higher prevalence of T2D was observed in those who consumed more eggs (> 5 eggs/week vs < 1 egg/month); however, a prospective analysis showed no association between egg consumption and incidence of T2D.^434^

In systematic reviews and meta-analyses with healthy individuals, there was also no consensus on the association between egg consumption and increased risk of CVD and T2D.^435 , 436^The results can be explained in part by confounding factors such as SFA intake and dietary energy intake, which favor weight gain and development of metabolic syndrome.^437^

### 13.3. Risk of Cardiovascular Diseases in Type 2 Diabetes

Another issue under discussion is the role of dietary cholesterol in cardiovascular risk in individuals with T2D or metabolic syndrome.

Observational and prospective studies associate egg consumption with a higher risk of CVD in the general population, while others only found association in individuals with T2D.^438^A meta-analysis concluded that the consumption of > 1 egg per day increased by 1.69 times the risk of developing CVD compared to the consumption of no eggs or < 1 egg per week. However, egg consumption was not associated with mortality.^439^

A randomized study of individuals with prediabetes or T2D (DIADEGG Study) who were assigned a diet with high (2 eggs/day for 6 days/week) or low (< 2 eggs/week) egg consumption for 3 months concluded that greater consumption of dietary cholesterol did not change plasma concentrations of HDLc, LDLc, and TC. The study also showed that there was no increase in risk factors for CVD in patients with T2D.^429^

In the NHS population, lower consumption of dietary cholesterol (assessed by the intake of eggs and meat) in patients with T2D was associated with healthier quality of life and thus lower risk of CVD. When quality-of-life factors were controlled for, the association between cholesterol consumption and risk of CVD was attenuated, suggesting that the improvement in quality of life is also associated with cardiovascular risk, and not only with dietary cholesterol.^440^

Results based on the Framingham Offspring Study population, which was followed up for 20 years, demonstrated no association of dietary cholesterol consumption with fasting lipid profile or risk of CVD in individuals with altered fasting blood glucose or T2D.^441^

An analysis of the prospective PREDIMED study population, which included participants with no previous cardiovascular events who were followed up for an average of 5.8 years, concluded that low or moderate egg consumption did not increase the risk of CVD in individuals either with or without T2D.^442^

Results of prospective randomized and observational studies, as well as systematic reviews and meta-analyses, are inconclusive regarding the association between greater consumption of dietary cholesterol and greater risk of CVD in individuals with T2D because of the high heterogeneity of the populations evaluated and methods used.

### 13.4. Impact on Cardiovascular Diseases

The available scientific evidence is conflicting regarding the impact of cholesterol intake on cardiovascular risk. Several studies suggest lack of association between dietary cholesterol and CAD or stroke, although there are limitations to be considered in the results.^427 , 443 , 444^In Asians, the highest quartile of dietary cholesterol consumption did not correlate with increased subclinical atherosclerosis assessed by calcium scoring.^445^In Finns, consumption of more than 400 mg of cholesterol per day was not associated with increased intima-media thickness or incidence of CAD.^446^However, in Americans, adding 300 mg of cholesterol to a baseline diet containing an average of 300 mg of cholesterol per day was associated with a 17% increase in CVD risk.^447^

Because high cholesterol consumption may be associated with an increased risk of developing CVD, and such risk may be dose-dependent, monitoring cholesterol intake is recommended.^447^

## 14. EGG

Egg is a low-SFA source of dietary cholesterol with high nutrient density and low cost. A chicken egg (50 g) contains high biological value protein (7.5 g), SFAs (1.6 g), MUFAs (1.8 g), PUFAs (0.9 g), and cholesterol (approximately 200 mg). Egg yolk is also rich in choline (147 mg), an essential nutrient for liver and muscle functions.^25 , 448^

The impact of egg consumption on lipid profile is quite variable.^449^In healthy adolescents, the consumption of more than 3 eggs per week is not associated with changes in lipid profile.^450^Similarly, in normolipidemic and physically active adults, the consumption of 2 eggs per day did not change plasma concentrations of lipoproteins after 12 weeks of study.^451^Conversely, a meta-analysis of 28 studies evaluating the consumption of from 5 eggs per week to 3 eggs per day showed that egg consumption in hyper-responsive individuals increases the concentration of TC by 5.60 mg/dL (95% CI: 3.11-8.09; P < 0.0001), LDLc by 5.55 mg/dL (95% CI: 3.14-7.69; P < 0.0001), and HDLc by 2.13 mg/dL (95% CI: 1.10-3.16; P < 0.0001), having a neutral effect on TG concentration compared to no egg consumption.^452^Nonetheless, there is evidence that egg consumption is associated with larger LDLc particles, which are less susceptible to oxidation and penetration into the endothelium.^449^

Findings on the impact of egg consumption on CVD risk, remain conflicting. A meta-analysis assessing the impact of consuming 1 egg per day versus < 2 eggs per week on the risk of CAD and stroke found no association between egg consumption and coronary risk in 7 studies of low heterogeneity.^453^Conversely, there was a 12% reduction in the risk of stroke with increased egg consumption and no dose-response relationship in the risk trend for stroke with increased egg consumption.^453^

In a cohort study of the Chinese population, high egg consumption (7 or more eggs per week) compared to low egg consumption (< 1 egg per week) was not associated with cardiovascular mortality, CAD, or stroke.^454^A study evaluating American population cohorts, considering an average consumption of 0.5 eggs per day (3 to 4 eggs per week), concluded that each additional 0.5 eggs consumed per day is associated with a 6% increase in risk of CVD (95% CI: 1.03-1.10) and an 8% increase in all-cause mortality (95% CI: 1.04-1.11). However, after statistical adjustment for cholesterol consumption, both associations were no longer significant, with an adjusted hazard ratio of 0.99 (95% CI: 0.93-1.05) for CVD incidence and an adjusted hazard ratio of 1.03 (95% CI: 0.97-1.09) for all-cause mortality.^447^A recent analysis of the results of 3 prospective cohort studies that included 177 000 individuals showed that moderate egg consumption (1 egg/day) was not associated with an increased risk of mortality or CVD.^455^

In high cardiovascular risk individuals, the degree of atherosclerosis (assessed by coronary angiography) was lower among those who consumed > 1 egg per week compared to those who consumed < 1 egg per week.^456^Similarly, the consumption of 2 eggs per day for 6 weeks did not affect endothelial function in individuals with CAD.^457^

A systematic review of cohort studies evaluating patients with T2D concluded that the consumption of at least 1 egg per day increased the risk of developing CVD by 69% (AMI, CAD, stroke, and ischemic heart disease) when compared to the consumption of < 1 egg per week, with no association with increased mortality.^439^

With regard to HF, a Swiss study assessing the results of two prospective cohorts concluded that daily consumption of 1 egg did not increase the risk of HF among men and women, but the consumption of > 1 egg per day increased the risk of HF by 30% in men, and the causal effect remains unclear.^444^

A review of current evidence is not able to establish a causal relationship between egg consumption and CVD. However, divergent results of observational studies suggest caution in egg consumption, especially among patients with T2D and those who are hyper-responsive to dietary cholesterol. Because eggs have high nutrient and protein density, they may be included in the diet as long as being part of a healthy dietary pattern.

### 14.1. Trimethylamine N-oxide in Cardiovascular Diseases

Studies have shown that the gut microbiota is involved in the development of CAD,^458^and trimethylamine N-oxide (TMAO) is an emerging research focus on the study of atherosclerosis progression. TMAO is an amine oxide that can be naturally found in the diet but also be metabolized from choline (abundant in eggs), carnitine (red meat), betaine, and phosphatidylcholine. These precursors are converted to trimethylamine (TMA) in the small intestine by specific bacteria such as Firmicutes, proteobacteria, and actinobacteria found in the gut microbiota.^459 , 460^TMA is absorbed and oxidized to TMAO through a reversible reaction in the liver, then catalyzed by the enzyme flavin-containing monooxygenase 3.^461^

Fish seems to be the largest food source of TMAO. Studies assessing fish intake show an increase in plasma TMAO concentrations (50 times higher) when compared to other food sources of carnitine or choline. Nevertheless, urinary excretion of TMAO and dimethylamine (derived from TMA) following fish consumption is higher compared to that of meat, dairy, fruits, vegetables, or grains.^462 - 464^

Elevated plasma TMAO concentrations correlated with increased risk of major cardiovascular events, prevalence of CVD, poorer prognosis, and increased risk of death.^461^This is because TMAO can exacerbate the inflammatory response in the vascular wall and induce the production of ROS. More recently, the role of TMAO in modulating cholesterol and bile acid metabolism and promoting atherosclerosis progression has been demonstrated.^463^

A mechanism by which TMAO may contribute to the progression of CVD is through an increased expression of scavenger receptors, which are responsible for the uptake of oxidized LDL, including class A scavenger receptors and surface protein CD36 in macrophages, both involved in cholesterol absorption. Some studies also suggest that TMAO prevents reverse cholesterol transport, which may contribute to the pathogenesis of CVD, promoting cholesterol accumulation in macrophages.^464^

Vascular events such as AMI and stroke in individuals with high plasma TMAO concentrations may be related to increased platelet activity due to cytoplasmic release of calcium, which may predispose the person to hypercoagulation and increased thrombotic events.^465 , 466^

A meta-analysis of studies recruiting over 26 000 participants followed up for about 4 years showed an increased relative risk (7.6%) of all-cause mortality for each increment of TMAO.^467^

A recent study evaluated the relationship of consumption of different protein sources (red meat, white meat, or vegetable protein) in TMAO metabolism. Long-term red meat consumption increased plasma TMAO concentrations by more than 3 times, as well as urinary excretion, compared to the other groups.^468^Studies on egg consumption have not found an association between egg consumption and increased TMAO. A study of 50 healthy participants showed that the consumption of 2 eggs (400 mg choline) per day did not change plasma TMAO concentrations.^460 , 468^

### 14.2. Hepatic Steatosis

Animal experiments suggest that high-cholesterol diets induce the progression of NASH, especially if combined with high-fat and high-energy diets.^469 - 472^However, there are no human studies showing the effect of dietary cholesterol on the development of hepatic steatosis. The current guideline for the treatment of NAFLD makes no reference to cholesterol consumption and etiology or treatment of this disease.^297^

## 15. Interesterified Fats

Interesterified fats have been used as a substitute for trans fatty acids, which are prepared using partial hydrogenation of vegetable oils. Interesterified fats are prepared using a fully hydrogenated solid base that is blended with a vegetable oil. Blended solid fractions such as palm stearin or lauric acid (found in coconut oil) and palm olein are used to prepare this solid base.^26^

The main characteristic of interesterified fats is the lack of trans fatty acids; however, they have a high concentration of SFAs. Interesterification is carried out through a chemical process that uses sodium methoxide as a catalyst, which promotes rearrangement of fatty acids in the glycerol molecule.^26^This forms TGs with new physical, organoleptic, and chemical properties, with enriched SFAs in the sn-2 position of glycerol, which is normally occupied by PUFAs in vegetable oils.^473^In this process, a large amount of TGs consisting of 3 SFAs are formed. Palmitic acid (more frequently) and stearic acid are the fatty acids most used in the food industry to replace trans fat.^473^

### 15.1. Studies in Animals

The consumption of a normolipidic diet containing interesterified fat produced from soybean oil, compared to a diet with soybean oil, by Wistar rats for 8 weeks resulted in higher expression of ATF3, an ER stress marker, and a higher concentration of the inflammatory cytokine TNF-α, with no difference in weight gain and glucose tolerance. However, greater weight gain was observed after 16 weeks of treatment, together with increased retroperitoneal adipose tissue mass and impaired glucose tolerance in the group that consumed interesterified fat.^474^

The effect of coconut oil, rice bran oil or sesame oil blended or subjected to enzymatic interesterification, with SFA/MUFA/PUFA ratio of 1:1:1 and PUFA/SFA ratio of 0.8:1, consumed for 60 days, was also evaluated in Wistar rats.^475^In animals fed interesterified oils, concentrations of TC, LDLc, and TG were reduced compared to animals fed blended oils. This was due to an increased expression of hepatic LDL receptor and the protein SREBP2, which induces cholesterol synthesis, compared to the same fat that had not undergone interesterification.^476^

Long-term consumption of a high-fat diet enriched with interesterified fat containing palmitic acid by LDL receptor knockout mice did not increase plasma cholesterol concentrations. However, there an increased concentration of cholesterol in LDL particles, a condition that resulted in higher atherosclerotic lesion, together with greater arterial macrophage infiltration.^477^Another study by the same research group demonstrated that long-term consumption of those diets in the same animal model led to greater weight gain, expanded adipose tissue, and adipocyte hypertrophy with greater inflammation, evidenced by increased pIKK and TNF-α levels.^478^

Other studies have evaluated the effect of a normolipidic diet containing interesterified fat rich in palmitic acid by female animals during pregnancy and lactation on the offspring. The results show that interesterified fat consumption predisposes the offspring to the development of obesity in adulthood,^479 , 480^suggesting a negative epigenetic influence. In addition, a study conducted by Misan et al. (2015)^480^found that, after 90 days of life, the offspring showed greater weight gain as well as lower EPA concentration and greater leukocyte circulation in the brain, with no increase in TLR4.

### 15.2. Studies in Humans

In humans, both partially hydrogenated and interesterified soybean oil provided an increase in the LDLc/HDLc ratio when compared to palm oil. In addition to the change in plasma lipid concentrations, interesterified fat had an adverse effect on glucose metabolism, reducing plasma insulin concentration and increasing fasting glucose.^481^However, a more recent study showed no changes in fasting glucose and insulin following interesterified fat consumption.^482^However, when compared to margarine containing high levels of linoleic acid and moderate levels of trans fat, the consumption of margarines containing palm oil (lauric, myristic, palmitic, oleic, and linoleic acids) or interesterified palm oil favored an increase in LDLc concentrations in hypercholesterolemic men.^483^A likely explanation to those different results is that Sundram et al.^481^used interesterified fat composed of stearic acid, while Filippou et al.^482^used palmitic acid. Both studies compared interesterified fat with palm oil.

Additionally, interesterification has been shown to transfer significant amounts of palmitic acid to the sn-2 position and UFAs to the sn-1 and sn-3 positions, which had an effect on plasma chylomicrons.^484^

Studies also showed that interesterified fat induced a lower postprandial plasma TG concentration in healthy menopausal women,^485^in healthy young adults,^486^and in hypertriglyceridemic adults^487^compared to palm oil.

Regarding the influence of nutritional status and the intake of interesterified fat consumption on lipoprotein profile,^488^interesterification was found to increase postprandial TG concentration (85%) in obese individuals. This was not observed in healthy individuals, suggesting that interesterification may affect them differently from those who are at risk of developing CVD and T2D.

In healthy individuals, interesterification did not change plasma lipid concentrations but favored a lower concentration of D-dimer, a fibrin degradation product associated with risk of CVD.^489^

To date, there is no scientific evidence for reaching a conclusion on the effect of the interesterification process on metabolic parameters, development of atherosclerosis, and cardiovascular outcome. However, it is important to note the high content of SFAs in interesterified fat that is currently used by the food industry.

## 16. Medium-chain Triglycerides

Medium-chain TGs are defined as structured lipids composed of a mixture of saturated-chain fatty acids, containing from 6 to 12 carbons, formed by caproic acid (C6: 1 to 2%), caprylic acid (C8: 65 to 75%), capric acid (C10: 25 to 35%), and lauric acid (C12: 1 to 2%).^367 , 490^The fatty acids of medium-chain TGs are obtained by fractionation of coconut or palm oils.^491^Except for lauric acid, the other fatty acids are absorbed via the portal system and, because they are not incorporated into chylomicrons, they do not induce an increase in plasma TG levels.^491 , 492^Lauric acid is preferably transported via the lymphatic system by chylomicrons.^38 , 493^For this reason, for the management of familial hyperchylomicronemia, when LPL is absent, the use of medium-chain TGs composed mostly of caproic, caprylic, and capric acids is indicated.^491^

## 17. Familial Chylomicronemia Syndrome

Familial chylomicronemia syndrome (FCS) is a rare autosomal recessive disease that affects 1 to 2 people per million.^494 , 495^It is characterized by severe hypertriglyceridemia, even when fasting, due to a deficiency in the enzyme LPL or in other proteins required for normal lipase activity. The most common homozygous mutations in FCS are found in the genes LPL, APOA5, GPBIHBP1 (glycosylphosphatidylinositol-anchored high-density lipoprotein-binding protein 1), APOC2, and LMF1 (lipase maturation factor 1), but compound heterozgous mutations may also appear in different genes that cause FCS.^496 - 498^TG concentrations are often 10 to 100 times higher than those found in normal individuals (< 150 mg/dL), ranging from 1500 to 15 000 mg/dL or higher.^499 , 500^Hypertriglyceridemia in FCS stems from the inability to metabolize TGs and other fats. TGs are normally metabolized via an LPL-dependent pathway.^500^Although an LPL-independent pathway exists, it is not sufficient to compensate for the loss of LPL function. In FCS, accumulation of chylomicrons and their remnants cannot be metabolized, and they build up in the plasma. In the pancreas, there is impairment of blood flow and activation of the inflammatory process, resulting in pancreatitis,^501 - 503^and this condition accounts for 10% of all causes of pancreatitis^501^. Patients with elevated TG-induced pancreatitis have more severe conditions, longer hospitalizations, required stay in the intensive care unit, high rates of progression to pancreatic necrosis, and a higher frequency of organ failure and mortality.^504^Pancreatitis may also progress to a chronic condition, with exocrine and endocrine pancreatic insufficiency, including pancreatic diabetes (type 3c), which can be fatal. Recurrent abdominal pain, lipemia retinalis, hepatosplenomegaly, lipemic plasma, eruptive xanthoma, and poor quality of life are other common findings.^505 - 510^Because those patients are not able to metabolize TGs, the current nutritional guidance consists of a very-low-fat diet (< 10-15% of total energy, or about 15-20 g of fat per day), restriction of refined carbohydrates, and alcohol withdrawal.^511^Additionally, individuals with FCS of all ages should be regularly monitored for the consumption of micronutrients, particularly fat-soluble vitamins.^511^Depending on individual tolerability, medium-chain TGs may be indicated for energy intake in the diet.^491^Medications that are known to elevate TGs should also be used with caution, such as diuretics, beta-blockers, systemic corticosteroids, retinoids, bile acid sequestrants, protease inhibitors, and antidepressants (sertraline). Supplementation with ω3 fatty acids and other drugs used to treat hypertriglyceridemia has been inconsistent in reducing TGs.^512 - 514^

## 18. Practical Aspects of Nutritional Intervention

The nutritional composition of the diet must be adjusted to the objectives proposed for each individual, considering the individual’s energy needs and cultural preferences. Several nutritional strategies can contribute to cardiovascular prevention provided they are based on the exclusion of trans fats, adequate SFA intake, and proportionally greater UFA intake, in addition to encouraging the consumption of fruits, vegetables, and whole grains.^9 , 515^

Foods of animal origin – such as meat, milk, and dairy products – naturally have a higher SFA content, while vegetable oils have a higher UFA content, except for coconut and palm oils, which are rich in SFAs. Among vegetable oils ( Table 1 ), soybean, canola and corn oils are most used, which have a good distribution of fatty acids. Soybean and canola oils have an additional advantage over corn oil: they have lower SFA content and higher ALA (ω3) content, which is essential for humans and is a precursor to EPA and DHA, also found in fish (Figures 1 and 2 ).

The amount of fat from meat varies according to the type of cut. Therefore, lean meat cuts, such as pork loin and pork tenderloin, have a SFA content similar to that of commonly recommended beef cuts, such as knuckle and rump steak ( Figure 3 ), making it possible to expand the options of protein-source foods with a cardioprotective focus.

Whole-milk dairy products have higher amounts of SFA than those produced with skimmed or semi-skimmed milk. Regarding cheese, those with lower water content and harder, such as parmesan cheese, proportionally have a higher SFA concentration than Brazilian cream cheese, Minas cheese, and ricotta cheese ( Figure 4 ). The choice between product types should consider the serving size, since even dairy products with less fat content may be important sources of SFAs if consumed in large amounts.

Nutritional guidance should enable consumers to understand the composition of foods, especially processed foods, since the amount and type of nutrients, especially fats, may vary within the same product type depending on the manufacturer (Table S1,Supplementary Material). In this context, adequate food labeling becomes essential for the processes of nutritional education and consumer choice. Another important aspect to be considered is food preparation. Deep frying, for example, can add a large amount of fat to food items, thus considerably increasing the energy intake. It is important to note that vegetable oils, which are sources of ω3 and ω6, should not be substituted for tropical oils (palm and coconut oils) or animal fats (lard and butter), as they are rich in SFAs and do not provide adequate amounts of essential dietary fatty acids. This guidance is in line with the latest AHA recommendation for cardiovascular risk prevention^8 , 9^and with the ESC/EAS guidelines, which recommend occasional use of tropical oils in small amounts.^10^

Finally, care should be taken in recommending the use of dietary supplements that have not been scientifically proven to provide health benefits. Therefore, non-pharmacological strategies to reduce cardiovascular risk should consider the available evidence that points to benefits, safety, costs, and tolerability, in addition to possible effects of drug-nutrient interactions. Another important aspect is that the misuse of supplements may compromise adherence to both pharmacological and nutritional treatment.^516^

## 19. Labeling and Trans Fatty Acids

The use of trans fats brings a number of advantages to the food industry, such as cost reduction, longer shelf life, high melting point, and wide possibilities of use. However, their association with increased cardiovascular risk is clearly established, so that several international and national guidelines recommend their exclusion from the diet. Reducing NCDs is one of the goals of the WHO Global Strategy on Diet, Physical Activity and Health,^517^which, in line with international guidelines,^9 , 10 , 518^recommends eliminating trans fats from the diet.^517^

In Brazil, the National Health Surveillance Agency (ANVISA), which is responsible for food labeling regulation, established in 2003 that food labels must state the amount per serving of trans fats present in the product.^519^However, despite the mandatory requirement, ANVISA resolution allows foods that contain an amount less than or equal to 0.2 g per serving to be declared as trans fat-free (labeled as “zero trans fat” or “does not contain trans fats”). It is important to note that this tolerance may lead to increased trans fat intake through the high intake of foods declared as trans fat-free, but which contain values close to 0.2 g per serving.^520^In addition, the serving declared on the label and considered trans fat-free is often smaller than the average amount of the product consumed.^520^Therefore, it is important that consumers receive guidance on how to identify the presence \of trans fats in the list of ingredients in order to avoid the intake of foods containing trans fats.

## 20. Final Considerations

This position statement shows that recent findings regarding the effects of fatty acids on intracellular signaling pathways and the results of clinical and epidemiological studies support the current nutritional guidelines for the prevention and treatment of cardiometabolic diseases. The grade of recommendation and level of evidence in regard of the effect of fatty acids on cardiovascular diseases are shown in table 2 and 3. International guidelines recommend eliminating trans fatty acids from the diet, reducing SFA intake, and including, in appropriate amounts, foods that are sources of UFAs. Epidemiological studies show that both excessive SFA intake and insufficient PUFA intake are associated with increased cardiovascular risk. In addition, the effects of fatty acid intake still depend on the dietary pattern in which they are consumed, since the replacement of SFAs with refined carbohydrates can increase cardiovascular risk. However, when isocalorically replaced with UFAs or even with complex carbohydrates, cardiovascular outcomes tend to be favorable. The benefits attributed to an adequate fatty acid profile are only observed in the presence of healthy eating patterns.

**Table 2:** – Dietary Fatty Acids and Cardiovascular Risk

Recommendation	Grade of recommendation	Level of evidence
*Trans* fatty acids must be excluded from the diet	III	A
Limit SFA consumption to < 7% of total energy Intake for individuals with high cardiovascular risk, such as people living with Diabetes Mellitus and familial hypercholesterolemia	I	A
Partially replacement of SFA with PUFA, should be recommended to intensify the reduction of plasma LDLc concentrations	I	A
Partially replacement of SFA with omega-6 PUFA can be recommended to improve insulin sensitivity	IIa	B
Replacement of SFA with PUFA can be recommended to reduce cardiovascular risk	IIa	A
Dietary recommendations should be based on total PUFA consumption and not on Omega-6/Omega-3 ratio	IIa	C
Stimulating the consumption of Omega-3 PUFA from vegetal sources, as part of a healthy diet, can be recommended to reduce cardiovascular risk	IIb	B
Stimulating the consumption of fish, as part of a healthy diet, should be recommended to reduce cardiovascular risk	I	B
Tropical oils (palm and coconut) should be used occasionally in limited amounts, because of their high SFA content	III	B

## 21. Nutritional Amounts of Fatty Acids and Cholesterol in Foods

**Table 1:** – Nutritional table with amounts of fatty acids and cholesterol in oils and fats. Food composition per 100 g of edible portion: fatty acids and cholesterol

Food	Total	Saturated fatty acids (g/100 g)					Monounsaturated fatty acids (g/100 g)		Polyunsaturated fatty acids (g/100 g)					Trans fats (g/100 g)	Cholesterol (mg)
		Total	Lauric acid 12:0	Myristic acid 14:0	Palmitic acid 16:0	Stearic acid 18:0	Total	Oleic acid 18:1	Total	ALA 18:3	EPA 20:5	DHA 22:6	Linoleic acid 18:2	Elaidic acid 18:1t	
Palm oil	100	43.1	0.28	0.79	36.77	4.61	40.1	39.86	16.1	0.83	0	0	15.69	0	NA
Extra-virgin olive oil	100	14.9	0	0	11.30	2.96	75.5	74.01	9.5	0.75	0	0	8.74	0	NA
Lard	100	39.2	0.2	1.3	23.8	13.5	45.1	41.2	11.2		0	0	10.2	0	95
Spray whipped cream with vegetable fat	27.3	25.9	10.70	3.64	2.63	7.46	0.1	0.05	0.1		0	0	0.08	0	tr.
Commercial mayonnaise made with eggs	30.5	4.1	0	0.02	2.84	0.37	6.4	6.24	15.4	1.43	0	0	13.86	0	42
Cocoa butter	100	59.7	0	0.1	25.5	33.2	32.9	32.6	3	0.1	0	0	2.8	0	0
Unsalted butter	86	51.5	2.11	7.96	23.87	9.64	21.9	19.80	1.5	0.27	0	0	1.22	2.31	214
Unsalted margarine with interesterified oil (65% lipids)	67.1	20.9	2.35	0.94	12.41	4.15	14.4	14.07	26.5	2.58	0	0	23.79	0.12	NA
Avocado oil	100	11.5	0	0	10.9	0.66	70.5	67.88	13.48	0.95	0	0	12.53	0	0
Cottonseed oil	100	25.9	0	0.8	22.7	2.3	17.8	17.0	51.9	0.2	0	0	51.5	0	0
Canola oil	100	7.9	0	0.06	4.59	2.21	62.6	61.14	28.4	6.78	0	0	20.87	0	NA
Coconut oil	99	82.4	41.8	16.6	8.63	2.5	6.3	6.25	1.7	0.019	0	0	1.67	0.02	0
Sesame oil	100	14.2	0		8.9	4.8	39.7	39.3	41.7	0.3	0	0	41.3	0	0
Sunflower oil	100	10.8	0	0.07	6.10	3.42	25.4	25.15	62.6	0.39	0	0	62.22	0	NA
Corn oil	100	15.2	0		12.12	2.18	33.4	33.04	50.9	0.96	0	0	49.44	0	NA
Soybean oil	100	15.2	0	0.08	10.83	3.36	23.3	22.98	60.0	5.72	0	0	53.85	0	NA

*Source: Núcleo de Estudos e Pesquisas em Alimentação – NEPA/Universidade Estadual de Campinas (UNICAMP). Tabela brasileira de composição de alimentos/NEPA-UNICAMP. Versão II. 2. ed. Campinas, SP: NEPA-UNICAMP, 2006. Available at: www.unicamp.br/nepa.25 USDA Food Composition Databases. United States Department of Agriculture. Agricultural Research Service USDA National Nutrient Database for Standard Reference Legacy Release, April 2018. USDA Branded Food Products Database. Available at: https://ndb.nal.usda.gov/ndb/search/list?home=true.520 ALA: alpha-linolenic acid; DHA: docosahexaenoic acid; EPA: eicosapentaenoic acid; NA: not applicable; tr.: trace.*

**Figure 1:** – Content of eicosapentaenoic acid (EPA) and docosahexaenoic acid (DHA) in fish (g/100 g)

**Figure 2:** – Content of monounsaturated fatty acids (MUFAs), polyunsaturated fatty acids (PUFAs), and saturated fatty acids (SFAs) in vegetable oils (g/100 g)

**Figure 3:** – Content of monounsaturated fatty acids (MUFAs), polyunsaturated fatty acids (PUFAs), saturated fatty acids (SFAs), and trans fatty acids in meats and eggs (g/100 g)

**Figure 4:** – Total content of saturated fatty acids in dairy products (g/100 g)

## 22. Grade of Recommendations and Level of Evidence: Fatty Acids and Cardiovascular Disease

**Table 3:** – Supplementation of omega-3 and cardiovascular risk

Supplementation of marine Omega-3 (2-4 g/dia) can be recommended in severe hypertriglyceridemia (> 500 mg/dL), as part of the treatment at the physician’s discretion	I	B
Purified Omega-3: Supplementation with formulation containing EPA (icosapent ethyl, 4 g/day) in patients with high cardiovascular risk and high levels of plasma triglycerides, on statin treatment, can be recommended since it seems to reduce the risk of major adverse cardiovascular events, including cardiovascular mortality, as part of the treatment at the physician’s discretion. This product is not locally accessible	I	A

## *Supplemental Materials

For additional information, please click here
